# Synthetic Lethality in Pancreatic Cancer: Discovery of a New RAD51-BRCA2 Small Molecule Disruptor That Inhibits Homologous Recombination and Synergizes with Olaparib

**DOI:** 10.1021/acs.jmedchem.9b01526

**Published:** 2020-02-10

**Authors:** Greta Bagnolini, Domenico Milano, Marcella Manerba, Fabrizio Schipani, Jose Antonio Ortega, Dario Gioia, Federico Falchi, Andrea Balboni, Fulvia Farabegoli, Francesca De Franco, Janet Robertson, Roberto Pellicciari, Isabella Pallavicini, Sebastiano Peri, Saverio Minucci, Stefania Girotto, Giuseppina Di Stefano, Marinella Roberti, Andrea Cavalli

**Affiliations:** † Computational and Chemical Biology, Istituto Italiano di Tecnologia, Via Morego 30, 16163 Genoa, Italy; ‡ Department of Pharmacy and Biotechnology, University of Bologna, Via Belmeloro 6, 40126 Bologna, Italy; § TES Pharma S.r.l., Via Palmiro Togliatti 22bis, I-06073 Corciano, Perugia, Italy; ∥ Department of Biosciences, University of Milan, Via Celoria 26, 20100 Milan, Italy; ⊥ Department of Experimental Oncology at the IEO, European Institute of Oncology IRCCS, IFOM-IEO Campus, Via Adamello 16, 20100 Milan, Italy; # Department of Experimental, Diagnostic and Specialty Medicine, University of Bologna, Via S. Giacomo 14, 40126 Bologna, Italy

## Abstract

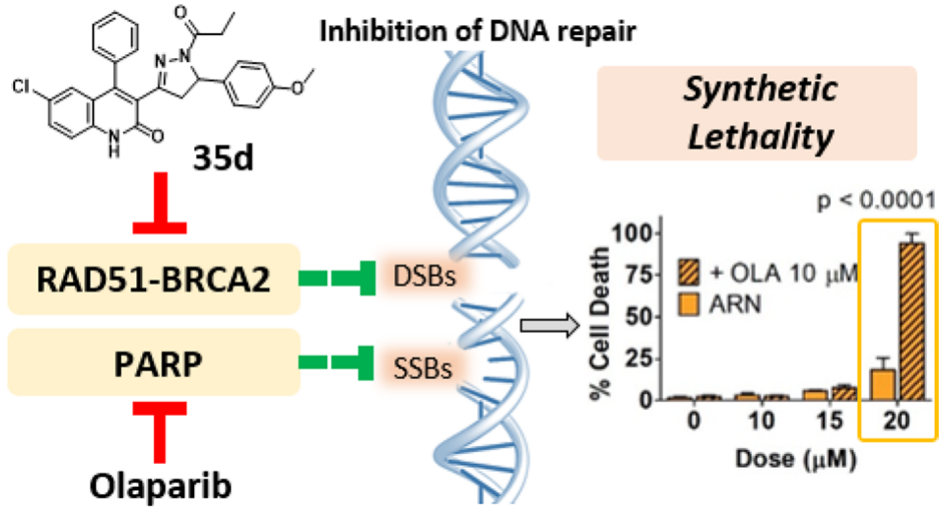

Synthetic lethality is an innovative framework for discovering novel anticancer drug candidates. One example is the use of PARP inhibitors (PARPi) in oncology patients with *BRCA* mutations. Here, we exploit a new paradigm based on the possibility of triggering synthetic lethality using only small organic molecules (dubbed “fully small-molecule-induced synthetic lethality”). We exploited this paradigm to target pancreatic cancer, one of the major unmet needs in oncology. We discovered a dihydroquinolone pyrazoline-based molecule (**35d**) that disrupts the RAD51-BRCA2 protein–protein interaction, thus mimicking the effect of *BRCA2* mutation. **35d** inhibits the homologous recombination in a human pancreatic adenocarcinoma cell line. In addition, it synergizes with olaparib (a PARPi) to trigger synthetic lethality. This strategy aims to widen the use of PARPi in *BRCA*-competent and olaparib-resistant cancers, making fully small-molecule-induced synthetic lethality an innovative approach toward unmet oncological needs.

## Introduction

Synthetic lethality is a new opportunity for discovering new anticancer molecules for personalized targeted therapies. The concept derives from genetic studies in model organisms.^1−5^ Two genes are synthetically lethal if the perturbation of either gene alone has no effect on cell viability, but the simultaneous impairment of both genes results in cell death. In principle, small organic molecules can target the synthetically lethal partner of an altered gene in cancer cells but not in normal cells. This creates opportunities to selectively kill cancer cells while sparing normal cells.^6−12^

The DNA repair and DNA damage response (DDR) pathways are suitable for the application of synthetic lethality as a novel anticancer therapeutic strategy.^13−15^ Genome instability is a hallmark of cancer.^16^ DNA damage occurs constantly in cells due to the continuous exposition to endogenous and exogenous stressors. Consequently, cells have evolved a complex coordinated DDR, which orchestrates a network of cellular processes to repair DNA damage and preserve genome integrity. DDR thus prevents the transmission of altered genetic material to daughter cells and acts as a tumor-suppressive barrier. Defects in DDR are associated with the accumulation of oncogenic mutations and genome instability, and they contribute to cancer initiation and progression. However, cancer cells with defects in one DDR pathway can become reliant on other pathways for their survival. Targeting these other DDR pathways can potentially cause selective cancer cell death through synthetic lethality. The classic example is the clinical application of poly (ADP-ribose)polymerase (PARP) inhibitors in oncology patients with *BRCA1/2* mutations. PARP is crucial for repairing DNA single-strand breaks (SSBs), whereas BRCA1/2 are important for repairing DNA double-strand breaks (DSBs) by homologous recombination (HR). The simultaneous impairment of both repair mechanisms results in cell-cycle arrest and apoptosis of cancer cells through synthetic lethality. In 2014, olaparib was the first PARP inhibitor (PARPi) approved to treat advanced ovarian cancer associated with defective *BRCA* genes.^17^ In 2018, olaparib was approved to treat metastatic breast tumors associated with germline *BRCA* mutations.^18^ In 2019, olaparib gained the FDA approval as first-line maintenance treatment of germline BRCA-mutated metastatic pancreatic cancer. It appears to be a new treatment option for this disease, which is one of the major unmet needs in oncology.^19^

One of BRCA2’s key mechanisms in DDR is to recruit RAD51, an evolutionarily conserved recombinase, at the site of DSBs where it performs DSB repair through HR.^20^ Additionally, the expression of RAD51 and the rate of RAD51-mediated HR are both elevated in a wide variety of cancers (e.g., breast, pancreatic).^21^ Moreover, the cellular amount of RAD51 is positively correlated with resistance to radiotherapies or chemotherapies that induce DNA damage.^22,23^

The RAD51-BRCA2 interaction is mediated by eight well-conserved motifs, known as BRC repeats.^24−27^ The X-ray crystallographic structure of the fourth BRC repeat (BRC4) is available in complex with the catalytic domain of RAD51, making the RAD51-BRCA2 interaction suitable for the structure-based design of small molecule inhibitors of protein–protein interactions (PPIs). Indeed, Lee et al. recently identified a small molecule inhibitor of RAD51-BRCA2 for potential cancer treatment.^28,29^

**Figure 1:**
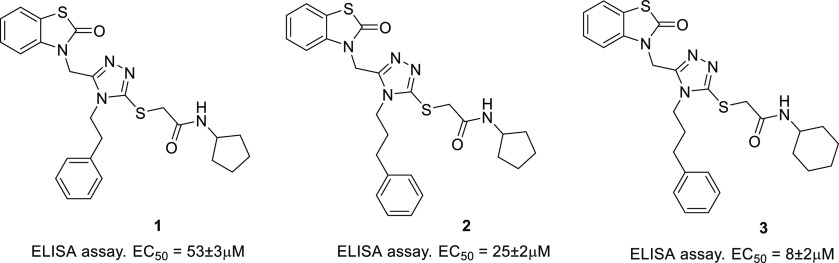
Structures of the previously identified triazoles **1**–**3**.

In this context, we recently proposed a new anticancer drug discovery concept, dubbed “fully small-molecules-induced synthetic lethality”.^30,31^ This concept combines RAD51-BRCA2 disruptors with olaparib to simultaneously impair two DNA repair pathways, thus mimicking the synthetic lethality described above. We carried out a successful virtual screening campaign at the FxxA pocket (i.e., zone I), one of the two RAD51 pockets responsible for BRC4 binding. This allowed us to discover a series of triazole-based compounds. Compounds **1** and **2** were selected as initial hit candidates (Figure 1). In line with our hypothesis, **2** increased the sensitivity to olaparib in pancreatic cancer cells (BxPC-3) with fully functional *BRCA2*. Notably, this synergistic effect was not observed in Capan-1, pancreas adenocarcinoma cells that lack *BRCA2*.^30^ Furthermore, to discover more effective compounds, we conducted a chemical modification campaign around the triazole moiety. We also improved the biological screening cascade with experiments to characterize how the new compounds disrupt the RAD51-BRCA2 interaction and inhibit DSB repair. We obtained compound **3** (Figure 1) with an improved profile relative to the initial hits (according to a biochemical ELISA assay) and a clear mechanism of action, allowing synergy with olaparib in cancer cells BxPC3, where olaparib is normally inactive. However, with **3**, we could not fully reproduce the paradigm of synthetic lethality.^31^ This could be due to its low-level potency, which did not cause HR inhibition greater than 40%. Additionally, the inherent resistance to apoptosis of BxPC3 cells, which bear a mutant p53, could have further prevented apoptosis. Therefore, further biological experiments and new classes of RAD51-BRCA2 are needed to confirm this paradigm and to assess its potential as an innovative anticancer strategy.

To this end, we report here on the identification of a new class of RAD51-BRCA2 disruptors based on the dihydroquinolone pyrazoline moiety (**4d**–**57d**, Table 1, Scheme 2). We attempt to depict general structure–activity relationships (SARs) of this new class of compounds as RAD51-BRCA2 inhibitors and outline the biological profile of the most promising derivative **35d** (Table 1, Scheme 2).

**Table 1:**
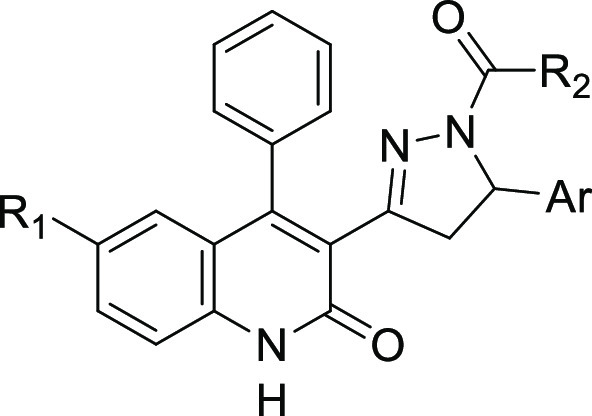

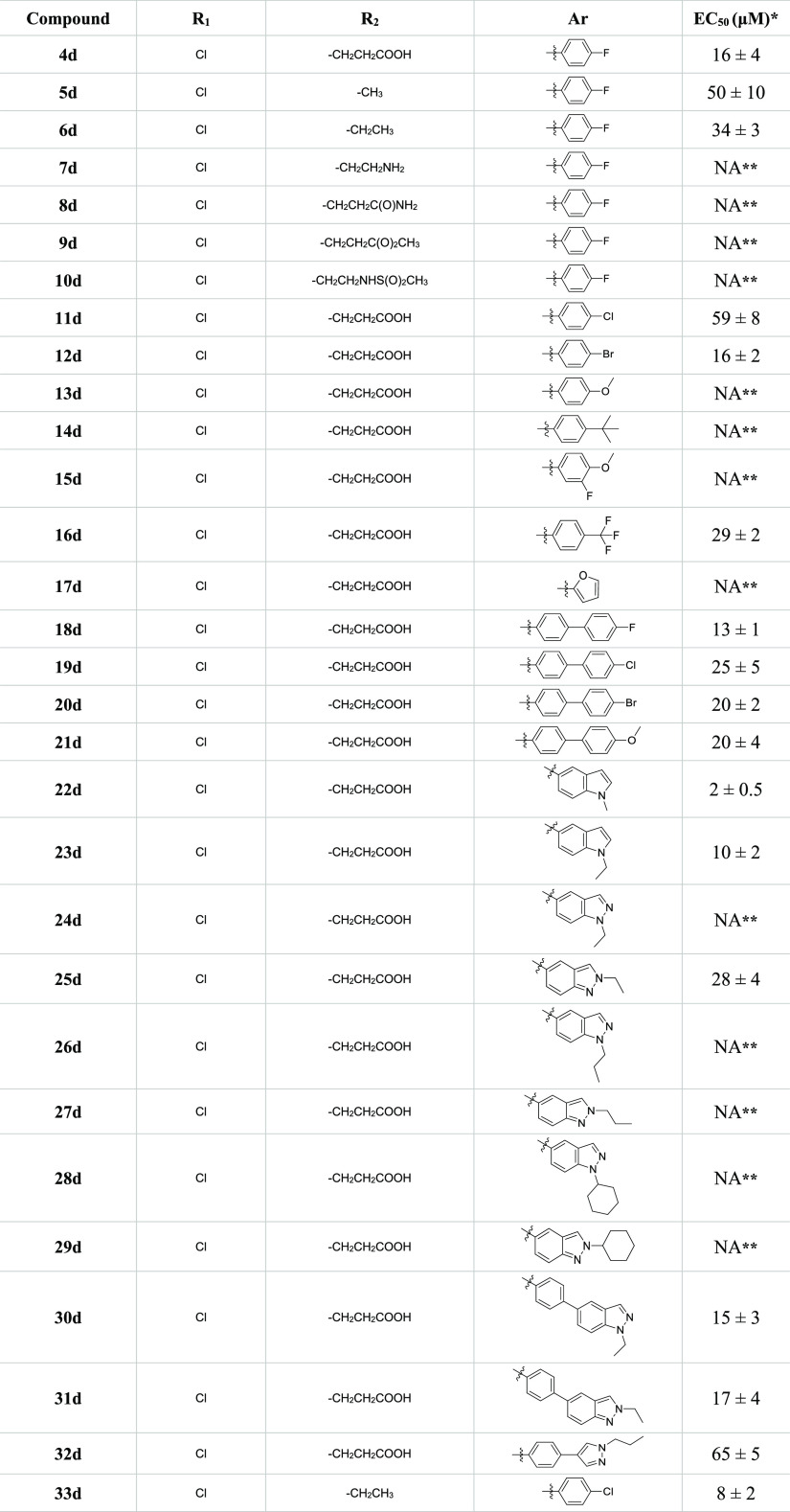

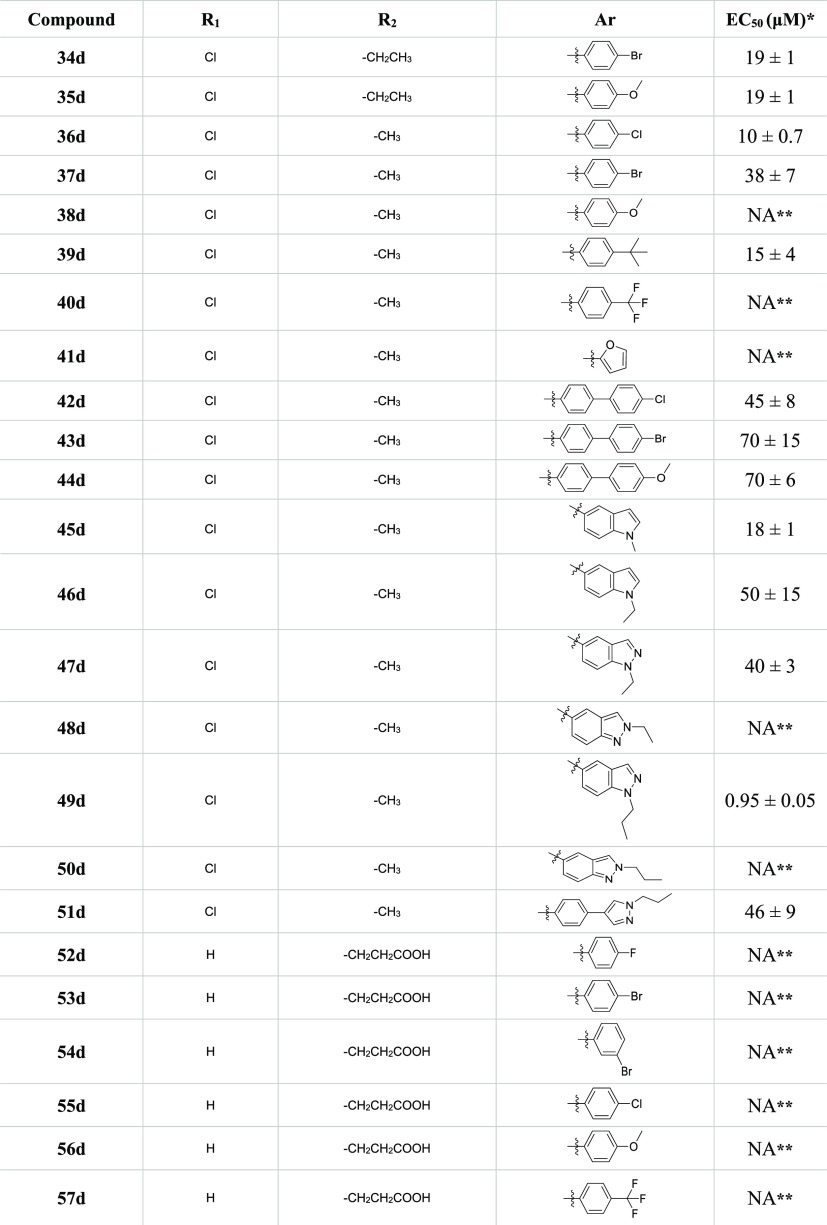
Structures and EC_50_ of Compounds **4d**–**57d** on ELISA Assay^a^

a Footnotes: *All points were tested in triplicate with error bars indicating the standard deviation. **NA: not active.

## Results and Discussion

### Hit Identification and Optimization

Targeting protein–protein interactions (PPIs) is an attractive strategy for designing innovative drugs for complex diseases such as cancer. Indeed, the first PPI inhibitors for cancer are now in clinical development.^32^

**Figure 2:**
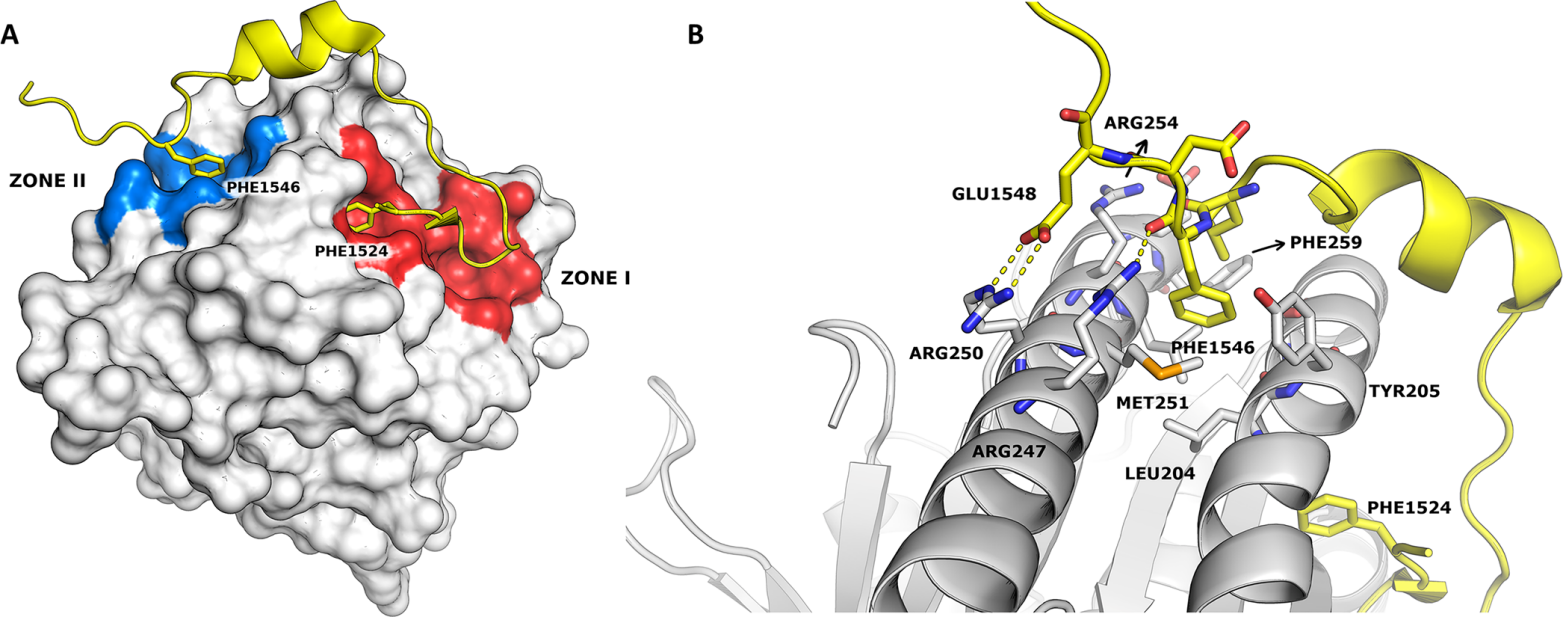
(A) RAD51-BRCA2 BRC repeat complex (PDB code 1N0W). RAD51 is represented as a surface, BRC4 as a cartoon. The two hot spots of the interaction between the proteins (Phe1524 and Phe1546) are highlighted in sticks. (B) Zone II magnification showing the interacting residues of BRC4 (yellow) and RAD51 (white).

In this context, to identify RAD51-BRCA2 disruptors, we used the available X-ray crystallographic structure of the fourth BRC repeat (BRC4) in complex with the catalytic domain of RAD51.^24^ BRC4 binds RAD51 in two different hydrophobic pockets (zone I and zone II, respectively). One pocket (named zone I) can lodge BRC4’s FxxA motif (residues 1524–1527 of BRCA2) and is critical for RAD51 multimerization. The other pocket (named zone II) can lodge the BRC4’s LFDE motif (residues 1545–1548 of BRCA2) far from the oligomerization interface (Figure 2).^24,33−35^ Recently, we ran a successful virtual screening campaign based on high-throughput docking at the FxxA pocket to identify the first RAD51-BRCA2 disruptors.^30^ To increase the chemical diversity and identify a novel class of RAD51-BRCA2 disruptors, we performed a second virtual screening campaign targeting the LFDE binding pocket (see the Supporting Information). This binding pocket is more evolutionarily conserved than the FxxA. Furthermore, mutation at the LFDE causes cellular lethality and failure of RAD51 assembly in nuclear foci at the site of DNA breaks in vivo. This further suggests this pocket as a critical site for RAD51’s mechanism of action.^33^ To the best of our knowledge, no inhibitor that binds the LFDE binding pocket has been reported so far in the literature. This may open up new possibilities for combining molecules targeting zone I and zone II toward a more in depth understanding of the mechanism of inhibition of RAD51-BRCA2 interaction.

**Figure 3:**
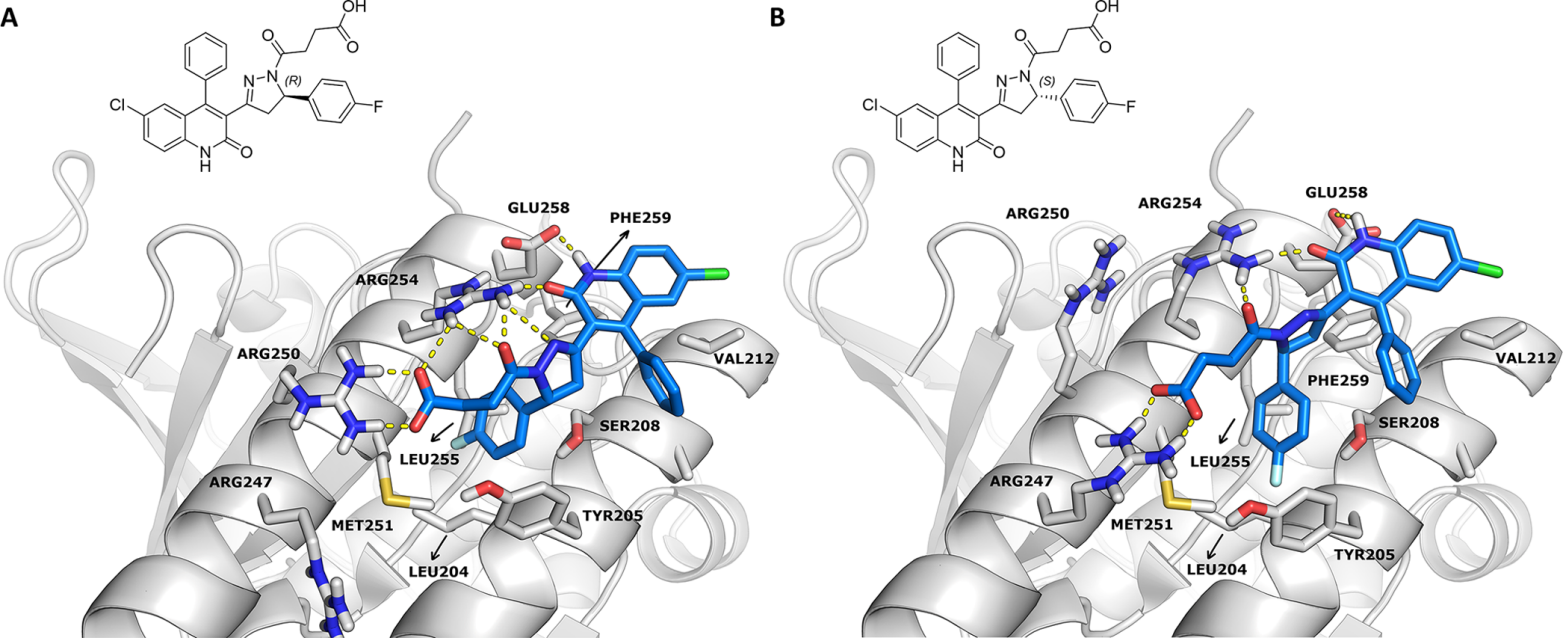
Both enantiomers of compound **4d** docked into the LFDE binding site (site II) of RAD51 (PDB code 1N0W).

**Figure 4:**
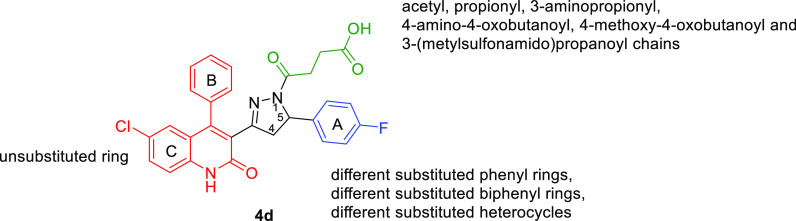
Overview of the optimization strategy of **4d** for SAR exploration.

Here, 42 small molecules were selected, purchased, and tested for their inhibitory activity using a competitive biochemical ELISA assay, as previously described by Rajendra et al.^33^ Among the tested compounds, the commercially available dihydroquinolone pyrazoline derivative **4d** (Figures 3 and 4) was the best candidate in terms of EC_50_ and chemical tractability. Its activity was confirmed by retesting the newly synthesized compound **4d** (Scheme 2). Indeed, the dihydroquinolone pyrazoline moiety is a core structure of compounds with different biological targets.^36,37^ The binding mode to RAD51 of both enantiomers of **4d** (Figure 3), as obtained by induced-fit docking simulations, displays some points of interaction similar to those of the crystallographic BRC4-RAD51 complex. Specifically, the docking model suggests that (i) the fluorophenyl ring in position 5 of the pyrazoline lies (similar to the Phe1546 of BRC4) in a hydrophobic pocket outlined by the side chains of Leu204, Tyr205, Met251, Leu255, and Phe259 of RAD51 and (ii) the carboxyl group of the pyrazoline side chain forms an ionic interaction with the Arg250 (or Arg247) of RAD51, as does the side chain Glu1548 of BRC4. In addition, the model suggests that the carbonyl and the nitrogen of the dihydroquinolone moiety, together with the carbonyl group of the pyrazoline side chain, establish hydrogen bonds with Arg254 and Glu258. Notably, both enantiomers show the same global pattern of interactions.

To improve the RAD51-BRCA2 inhibitory activity of **4d**, we conducted a chemical modification campaign around the dihydroquinolone pyrazoline core. We synthesized a chemical library that contained a variety of aromatic substitutions (red and blue regions) in combination with modifications of the acyl chain moiety (green region) (Figure 4). All compounds were synthesized and tested as racemic mixtures, after verifying that the enantiomers of the hit compound **4d** showed the same biochemical activity and the same binding mode (Figure 3, details in the Biological Evaluation section). First, a series of different acyl chains (namely, acetyl, propionyl, 3-aminopropionyl, 4-amino-4-oxobutanoyl, 4-methoxy-4-oxobutanoyl, 3-(methylsulfonamido)propanoyl) was introduced on the pyrazoline nitrogen (**5d**–**10d**, Table 1). Next, the aromatic ring A was modified by replacing the fluorine atom with different substituents, including chlorine and bromine atoms and methoxy, *tert*-butyl, and trifluoromethyl groups, leaving the succinate acyl chain unchanged (**11d**–**16d**, Table 1). In addition, the aromatic ring A was replaced by different substituted biphenyl or heterocycle groups in order to probe its role (**17d**–**32d**, Table 1). The ring A was also modified in combination with the propionyl (**33d**–**35d**, Table 1) or acetyl substitution on the pyrazoline nitrogen (**36d**–**51d**, Table 1). Regarding the dihydroquinolone core, the chlorine atom in the C-ring was removed, leaving the acyl chain unchanged and introducing some different substituents in the phenyl ring A (**52d**–**57d**, Table 1).

### Chemistry

**Scheme 1:**
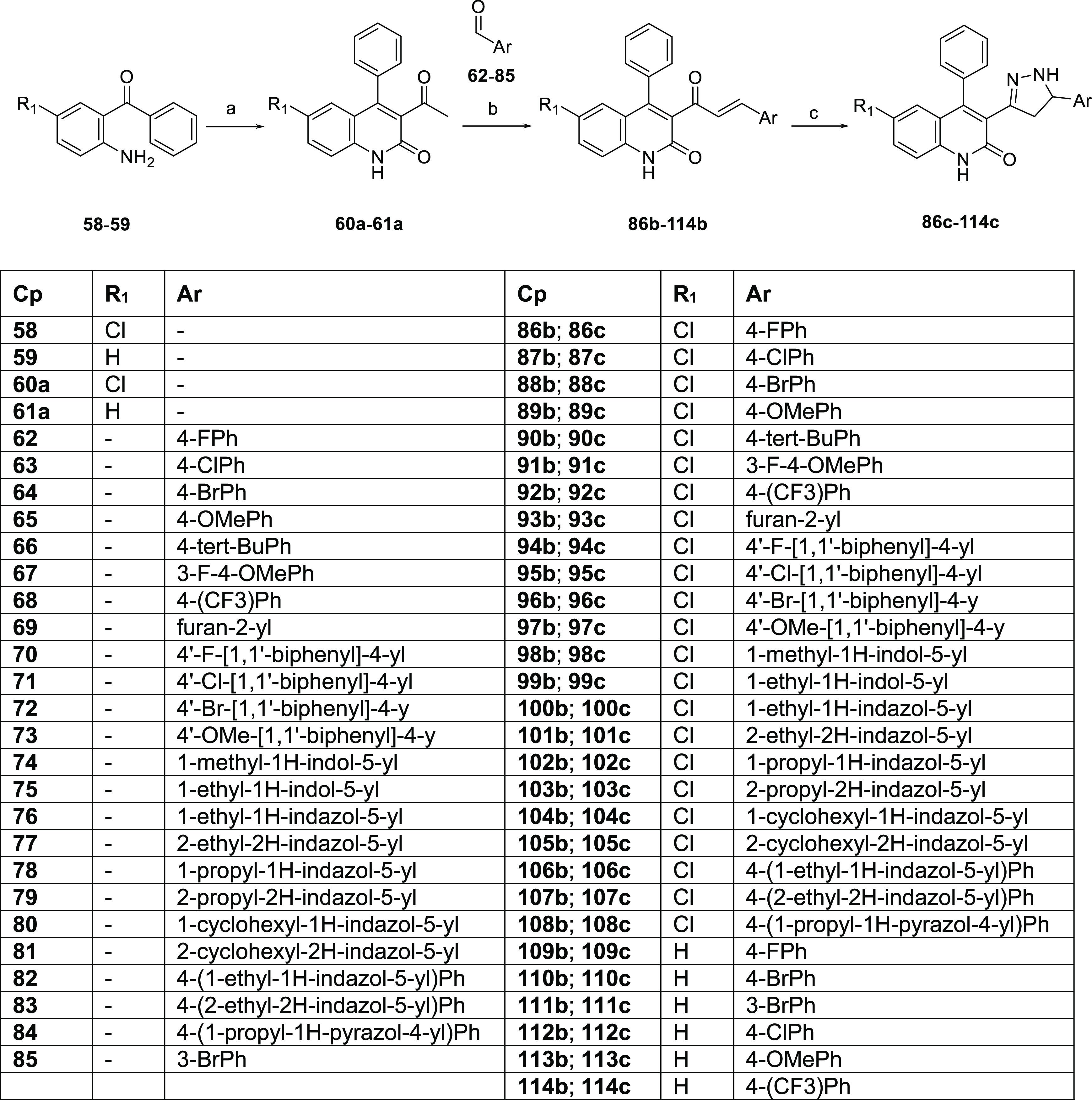
Synthesis of Dihydroquinolone Pyrazoline Intermediates **86c**–**114c** Reagents and conditions: (a) ethyl acetoacetate, DMF, 120 °C μWave or reflux, **60a** quantitative, **61a** 53%; (b) KOH, EtOH/H_2_O 4:3 v/v (0.05 M), 0 °C to rt, 53%, quantitative; (c) hydrazine monohydrate, EtOH, 110 °C μWave, 45 min, 64%, quantitative.

**Scheme 2:**
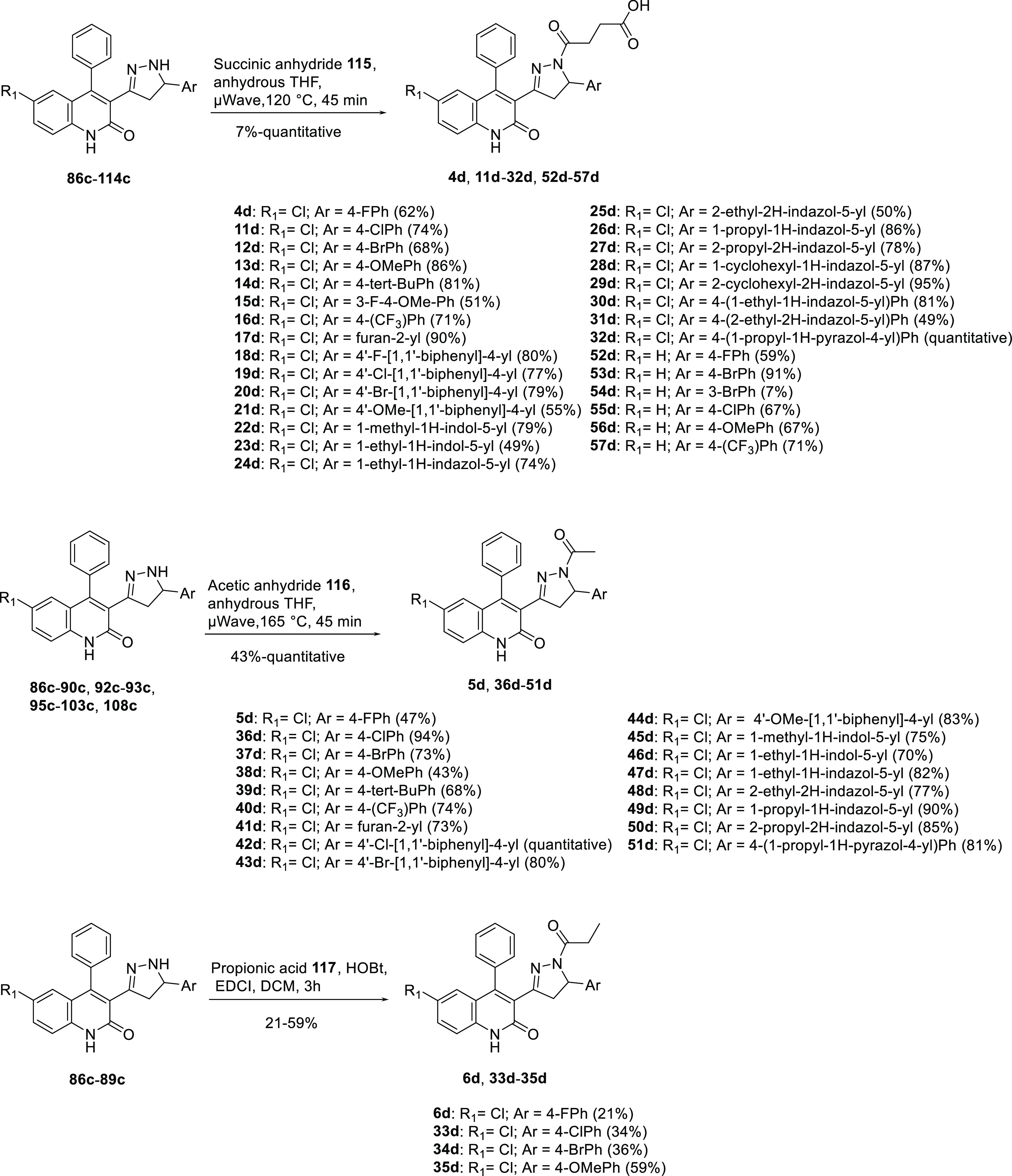
Synthesis of Final Dihydroquinolone Pyrazolines **4d**–**57d**

**Scheme 3:**
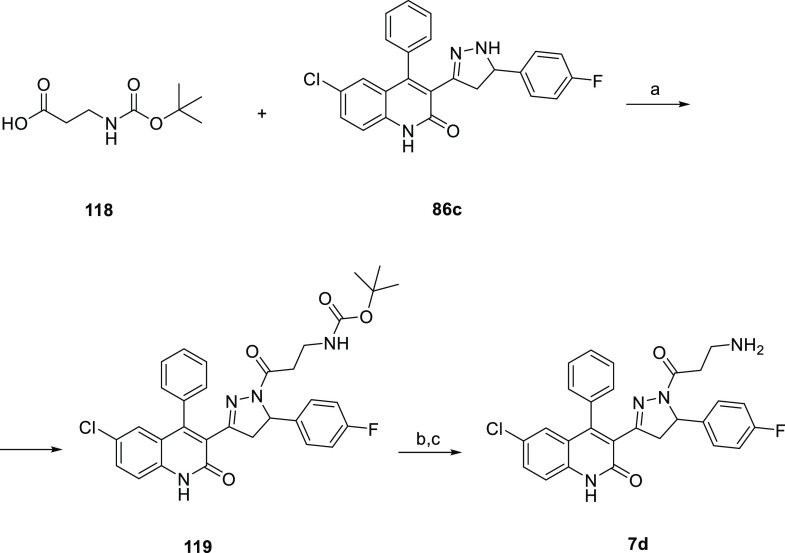
Synthesis of Compound **7d** Reagents and conditions: (a) HOBt, EDCI, DCM, overnight, yield 25%; (b) HCl 4 M in dioxane, rt, 15 min; (c) NaOH 0.5 M in EtOAc, rt, 15 min, yield 50%.

**Scheme 4:**
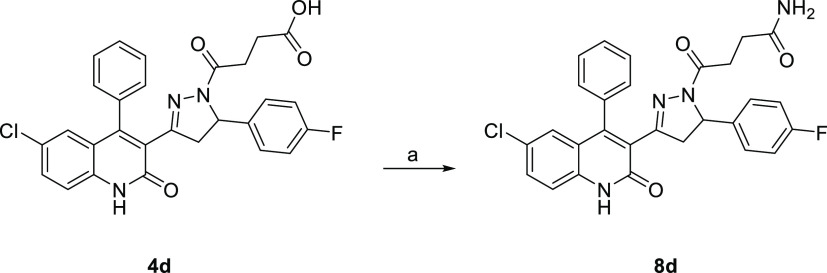
Synthesis of Compound **8d** Reagents and conditions: (a) HATU (1.5 equiv), EDC (1.5 equiv), DCM, DMF, ammonium chloride (5.0 equiv), DIPEA (4.0 equiv), rt, 26 h, yield 46%.

**Scheme 5:**
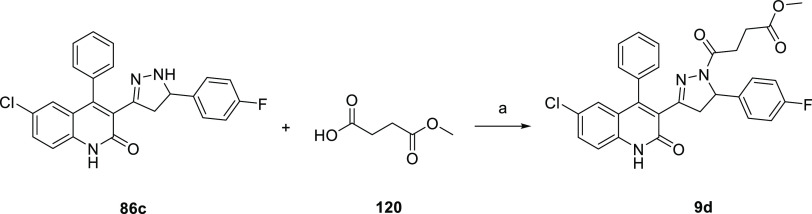
Synthesis of Compound **9d** Reagents and conditions: (a) HOBt (1.1 equiv), EDC (1.1 equiv), TEA (2.2. equiv) DCM, rt, 16 h, yield 42%.

**Scheme 6:**
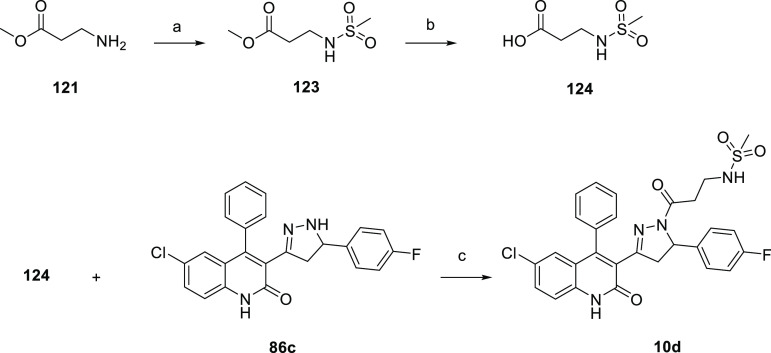
Synthesis of Compound **10d** Reagents and conditions: (a) TEA (5.0 equiv), anhydrous DCM, methanesulfonyl chloride **122** (2.0 equiv), rt, 48 h, yield 82%; (b) MeOH/THF (1:1 v/v), 2 M LiOH, rt, 16 h, yield 82%; (c) HOBt (1.1 equiv), EDC (1.1 equiv), TEA (2.2 equiv), DCM, rt, 16 h, yield 15%.

The desired dihydroquinolone pyrazoline derivatives **4d**–**6d** and **11d**–**57d** were achieved, taking advantage of a common synthetic strategy previously reported by Acker et al.^36^ The commercially available 2-aminobenzophenones **58** and **59** treated with ethyl acetoacetate afforded the corresponding methyl ketones **60a** and **61a**, which underwent base-catalyzed condensation with the appropriate aryl aldehydes **62**–**85** yielding the α,β-unsaturated aryl ketones **86b**–**114b**. In turn, **86b**–**114b** were treated with hydrazine monohydrate to yield the pyrazoline derivatives **86c**–**114c** (Scheme 1). The pyrazoline amines **86c**–**114c** functionalized with succinic anhydride **115**, acetic anhydride **116**, or propionic acid **117** afforded the corresponding desired dihydroquinolone pyrazoline derivatives **4d**–**6d** and **11d**–**57d** (Scheme 2). **7d** (Scheme 3) was obtained by coupling **86c** with the commercially available 3-((*tert*-butoxycarbonyl)amino)propanoic acid **118** to achieve the Boc-aminopyrazoline derivative **119**. In turn, **119** was Boc-deprotected to afford the desired **7d**. **8d** (Scheme 4) was obtained by HATU-mediated coupling of **4d** with ammonium chloride. **9d** (Scheme 5) was afforded by coupling **86c** with the commercially available 4-methoxy-4-oxobutanoic acid **120**. The synthesis of **10d** (Scheme 6) began with the commercially available methyl 3-aminopropanoate **121**, which was treated with methanesulfonyl chloride **122** to afford the corresponding methyl 3-(methylsulfonamido)propanoate **123**, which underwent basic hydrolysis to give 3-(methylsulfonamido)propanoic acid **124**. The coupling reaction of **86c** with **124** afforded the desired **10d**.

The aldehydes **67**, **70**, **74**–**84**, not commercially available, were prepared following standard procedures as reported in Supporting Information (Schemes S1–S9).

### Biological Evaluation

To investigate the mechanism of action of the new dihydroquinolone pyrazoline derivatives, different biological assays were performed. As a primary screening, the ability of compounds **5d**–**57d** to inhibit RAD51-BRCA2 interaction was investigated with a competitive biochemical ELISA assay against the parent compound **4d** (Table 1). This assay is effective in evaluating the ability of new molecules to compete with BRC4 to bind to RAD51.^30^ Replacing the acyl chain of the pyrazoline nitrogen yielded compounds (**5d**–**10d**, Table 1) that had reduced inhibitory activity (**5d**, EC_50_ = 50 ± 10 μM; **6d**, EC_50_ = 34 ± 3 μM) or were completely inactive (**7d**–**10d**). These data indicate that no improvement in RAD51-BRCA2 inhibitory activity was achieved with this subset of compounds relative to the parent **4d** (EC_50_ = 16 ± 4 μM). Replacing the fluorine atom on ring A with bromine led to **12d** (EC_50_ = 16 ± 2 μM), which shows the same activity as the initial hit **4d**. The activity was affected when the fluorine was replaced with chlorine (**11d**, EC_50_ = 59 ± 8 μM) and trifluoromethyl groups (**16d**, EC_50_ = 29 ± 2 μM). The replacement of fluorine with electron-donating groups yielded the inactive compounds (**13d**–**15d**). For the subset of dihydroquinolone pyrazolines, in which the aromatic ring A was replaced by different substituted biphenyl or heterocycle groups (**17d**–**32d**, Table 1), the *N*-methylindole derivative **22d** showed a fairly good potency with an EC_50_ = 2 ± 0.5 μM, 8 times higher than that of the parent **4d** (EC_50_ = 16 ± 4 μM). **18d**–**21d**, **23d**, **25d**, **30d**, and **31d** were active at micromolar range, all very similar to the initial hit. A drop in activity was observed with compound **32d**, while derivatives **24d**, **26d**–**29d** were inactive. The replacement of the aromatic ring A in combination with the substitution of the pyrazoline nitrogen with either propionyl or acetyl chain yielded compounds **33d**–**51d**. The 1-*N*-acetyl-5-(1-*N*-propyl)indazolylpyrazoline **49d** showed the best activity of the series with EC_50_ = 0.95 ± 0.05 μM, while **33d**–**36d**, **39d**, **45d** showed an activity very similar to that of the initial hit. A drop in potency was observed with compounds **37d**, **42d**–**44d**, **46d**–**47d**, and **51d**, while **38d**, **40d**–**41d**, **48d**, and **50d** were inactive. Finally, removing the chlorine atom on the dihydroquinolone core led to the completely inactive compounds **52d**–**57d**, suggesting an active role for the halogen. The enantiomers (**4d-I** and **4d-II**) of the racemic hit compound **4d**, separated via reverse phase chiral chromatography, showed a very similar inhibitory activity (**4d-I**, EC_50_ = 4 ± 0.5 μM; **4d-II**, 10 ± 1 μM) to that of the parent **4d**, suggesting no stereochemical preference of these compounds for the hypothesized molecular target RAD51 (see Experimental Section). As expected for PPI disruptors, the SARs of the new series of dihydroquinolone pyrazoline were rather complex to rationalize, with many cliffs and spikes that were difficult to understand. Nonetheless, the SAR campaign allowed us to identify several compounds with interesting EC_50_ values ranging from 0.95 to 20 μM. Accordingly, **4d**, **12d**, **18d**, **20d**–**23d**, **30d**, **31d**, **33d**–**36d**, **39d**, **45d**, and **49d** were submitted to cell-based study (Table 2).

**Table 2:** Preliminary Biological Screening of Compounds Showing EC_50_ on ELISA Assay Ranging from 0.95 to 20 μM

compd	EC_50_ ELISA (μM)	preliminary biological screening
**4d**	16 ± 4	HR inhibition = 10%, 40 μM
**12d**	16 ± 2	HR inhibition = not present
		olaparib association = not present
**18d**	13 ± 1	olaparib association = not present
**20d**	20 ± 2	olaparib association = not present
**21d**	20 ± 4	NE^a^
**22d**	2 ± 0.5	HR inhibition = not present
		olaparib association = not present at 5 μM
**23d**	10 ± 2	olaparib association = not present
**30d**	15 ± 3	NE^a^
**31d**	17 ± 4	HR inhibition = NDD^b^
		olaparib association = present at 20 μM
**33d**	8 ± 2	HR inhibition = not present
		olaparib association = not present
**34d**	19 ± 1	HR inhibition = not present
		olaparib association = NE^a^
**35d**	19 ± 1	HR inhibition = 54%, 20 μM
		olaparib association = present at 15 μM
**36d**	10 ± 0.7	HR inhibition = 24%, 10 μM
		olaparib association = not present
**39d**	15 ± 4	HR inhibition = NDD^b^
**45d**	18 ± 1	HR inhibition = not present
**49d**	0.95 ± 0.05	HR inhibition = not present
		olaparib association = not present

a NE, not evaluable.

b NDD, not dose-dependent.

Our working hypothesis is that compounds disrupting RAD51-BRCA2 interaction should affect HR repair and increase the efficacy of PARPi in treating breast, ovarian, and pancreatic cancer. For its clinical relevance, pancreatic adenocarcinoma was selected as our final model for cell-based experiments, and BxPC3 cells were selected for a straightforward comparison with the previously reported triazole derivatives.^30,31^ BxPC3 is derived from a human adenocarcinoma that expresses fully functional BRCA2.^38^ As shown in Table 2 and according to the rationale of our hypothesis, our preliminary screening consisted of verifying the efficacy of compounds in inhibiting cell HR and/or in increasing the antiproliferative effect of olaparib. For each compound, both parameters were verified using only one or two doses, in the range of the EC_50_ obtained with the ELISA test. HR activity was assessed by evaluating the recombination rate between two transfected plasmids, using a commercially available assay. This preliminary investigation allowed us to rapidly exclude molecules showing (i) low or no activity (**4d**, **12d**, **18d**, **20d**, **22d**, **23d**, **33d**, **45d**, **49d**), (ii) poor solubility in cell culture media (**21d**, **30d**, **34d**), (iii) discrepancy between the data obtained in the two different screening procedures such as HR inhibition not confirmed by increased olaparib efficacy in the viability assay or, on the contrary, increased olaparib efficacy without HR inhibition (**31d**, **36d**), (iv) a non-dose-dependent effect (**39d**). As for the emissucinic acid-containing compounds (**4d**, **12d**, **18d**, **20d**–**23d**, **30d**, **31d**), one of the reasons for their lower potency in cells might be the general poor permeability, likely related to the ionizable acid moiety. The data reported in Table 2 show that **35d** was the most promising compound in the HR activity test. We then characterized **35d** in additional biophysical and cell-based experiments.

**Figure 5:**
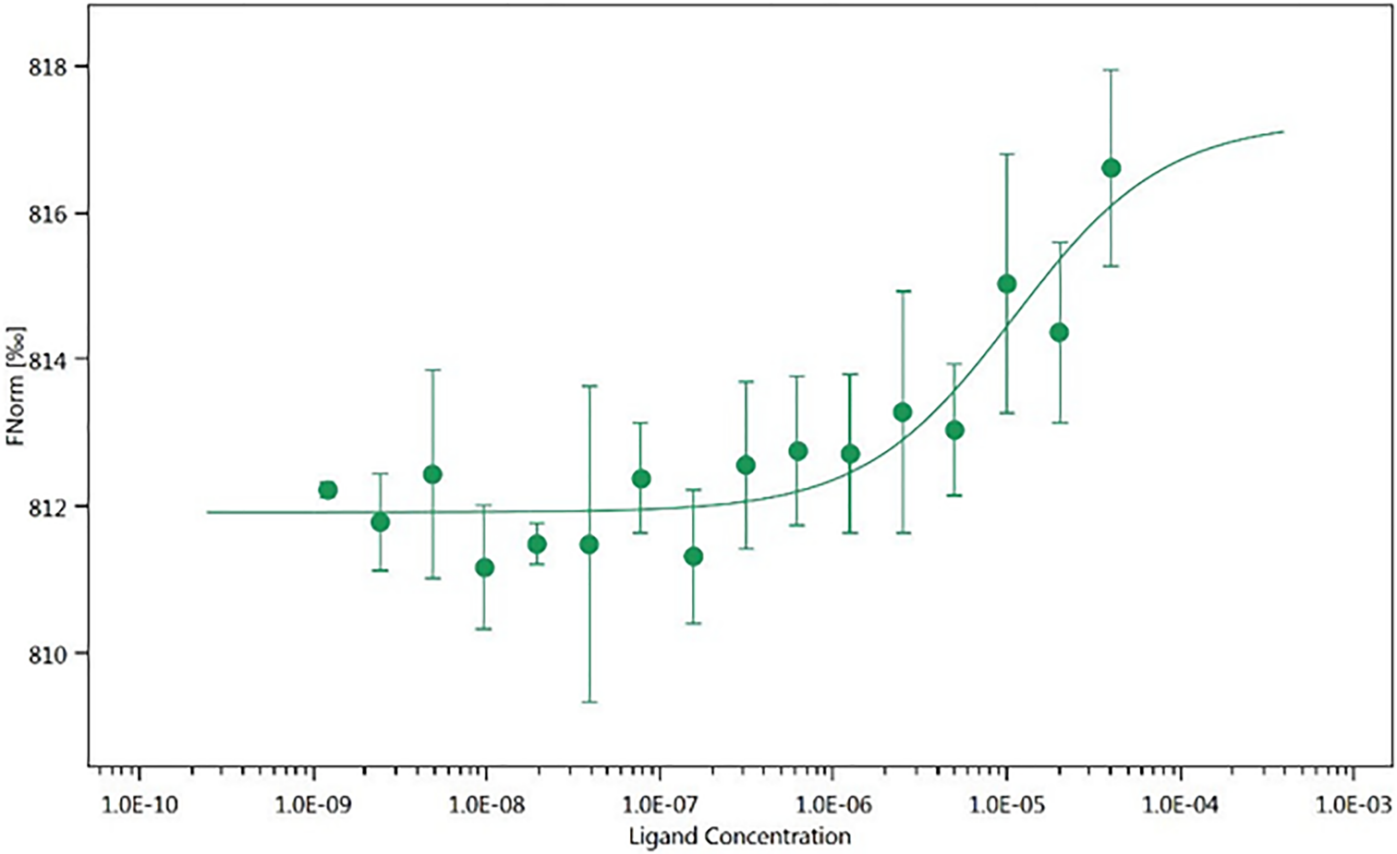
MST analysis of His-hRAD51-**35d** binding. Titration curve of (RED-tris-NTA 2nd Generation)-His-hRAD51 (80 nM) with increasing concentrations of **35d**. Sigmoidal fitting curve was obtained using the Affinity Analysis software of NanoTemper Technologies. MST data are the average of three replicates.

To further assess the physical interaction between **35d** and RAD51, a microscale thermophoresis (MST) assay was performed on the recombinant human RAD51 (see Experimental Section). The binding assay allowed us to determine the dissociation constant (*K*_d_) for the RAD51-**35d** interaction and binding. The final binding curve (Figure 5) shows that **35d** binds to RAD51 with a *K*_d_ value of 11 ± 6 μM. This is in agreement with the ELISA assay, supporting the initial hypothesis that **35d** could act as a RAD51-BRCA2 disruptor.

**Figure 6:**
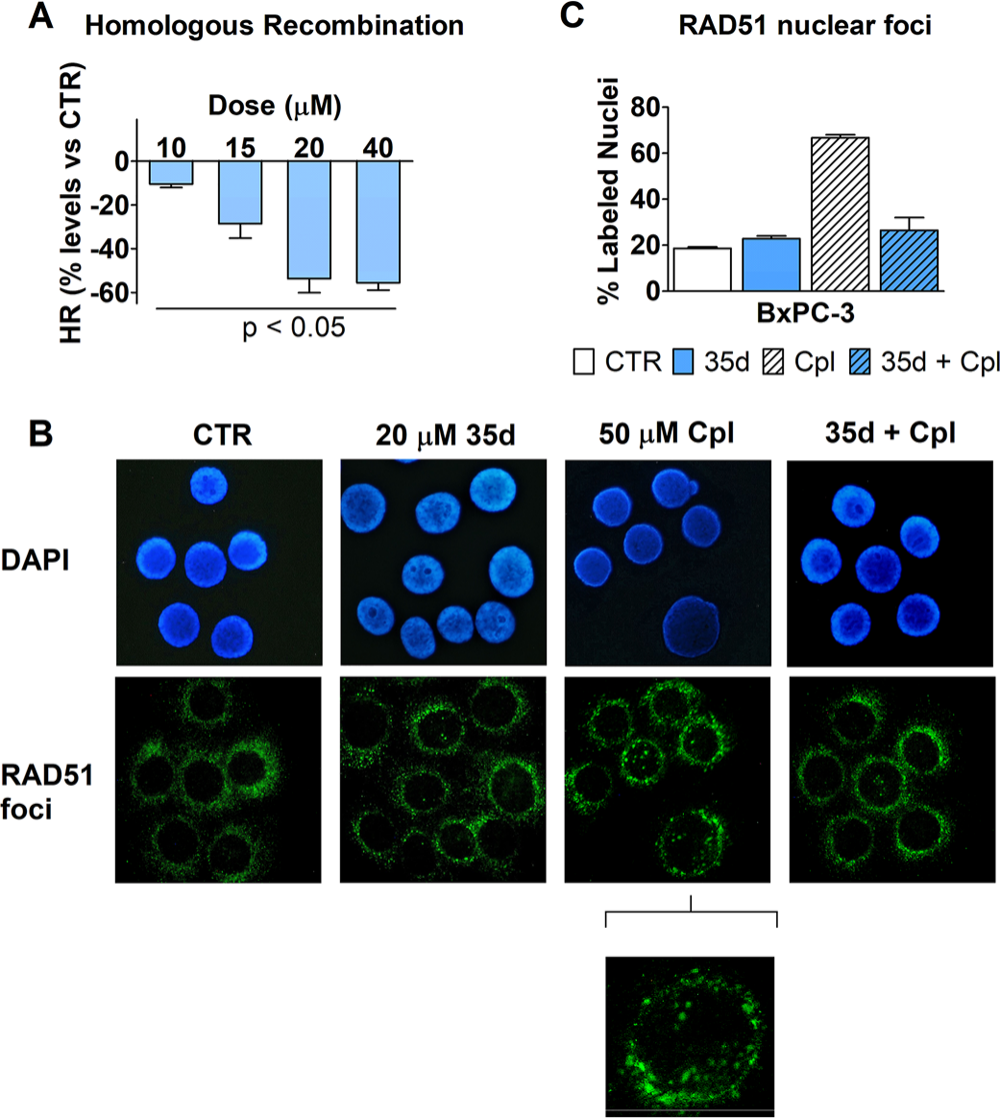
(A) Effect on HR caused by **35d** administered to BxPC3 cells during plasmid transfection (5 h). HR was evaluated by real-time PCR, as described in the Experimental Section. Data were statistically analyzed using the column statistics of Prism 5 software, which applies the inferences analysis and the one-sample *t* test. The observed inhibitory effect was significantly different from 0 (the level of untreated cultures) for all tested doses, with *p* < 0.05. (B, C) Immunofluorescence detection of RAD51 in BxPC-3 nuclei after a treatment with 50 μM cisplatin (Cpl) given separately or in combination with 20 μM **35d**. Experimental details are reported in the Experimental Section. (B) Representative pictures showing DAPI-stained cell nuclei and the corresponding immune-labeling of RAD51 localization. In untreated cells (CTR), RAD51 labeling is clearly evident in cytoplasm and does not appear in cell nuclei. In the pictures of Cpl-exposed cells, nuclear localization of the protein is clearly evident in 3 out of the 6 shown nuclei. A higher magnification detail was included for this sample. (C) The bar graph shows the percentage of RAD51-labeled nuclei counted by two independent observers who analyzed the treated cultures. Data were statistically evaluated by applying the one-way ANOVA, which indicated a significantly increased nuclear RAD51 labeling caused by Cpl (*p* < 0.05) and no statistically significant difference between cells treated with **35d** and those exposed to **35d** + Cpl.

To further characterize the effect of **35d** on HR, the compound was administered at different doses to BxPC3 cells for 5 h simultaneously with plasmid transfection; as shown in Figure 6A, it produced a statistically significant dose–response effect in reducing cell HR. The compound was tested up to 40 μM (an upper limit in terms of solubility), and we estimated the dose causing a 50% inhibition of HR by applying the polynomial regression to the collected data. The EC_50_ was 18.4 μM.

An additional evidence of compromised HR was obtained by assessing the localization of RAD51 in BxPC-3 nuclei after DNA damage. The results of this experiment are reported in Figure 6B,C. To obtain massive DNA damage, BxPC-3 cultures were exposed for 1 h to 50 μM cisplatin. The immunohistochemical staining of RAD51 shown in Figure 6B revealed evident nuclear foci in cisplatin-treated cells, which appeared significantly reduced when the drug was administered in association with 20 μM **35d**. Furthermore, the percentage of RAD51-labeled nuclei measured in cultures exposed to 20 μM **35d** was superimposable to that observed in cells exposed to the association of cisplatin with 20 μM **35d** (Figure 6C).

These results were in good agreement with the ELISA and MST outcomes, ultimately pointing to **35d** as a novel RAD51-BRCA2 disruptor with a clear capability to interfere with HR.

**Figure 7:**
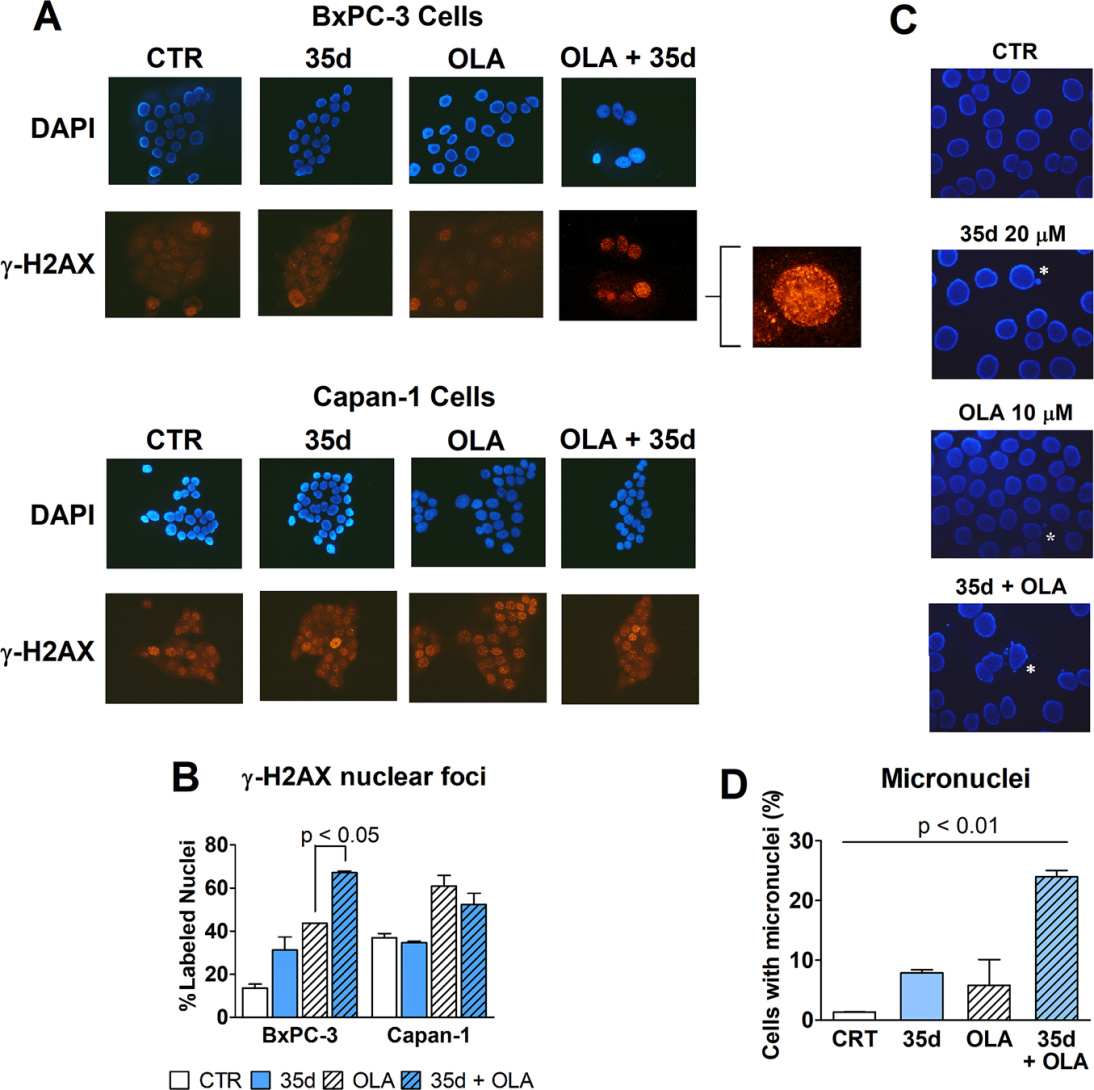
(A, B) Evaluation of DNA damage through immune detection of nuclear γ-H2AX foci in BxPC-3 and Capan-1 cells exposed for 48 h to olaparib (10 μM) or **35d** (20 μM), given alone or in combination. (A) Representative pictures showing DAPI-stained cell nuclei and the corresponding immune-labeling of γ-H2AX. In BxPC-3 cells, coadministration of **35d** and olaparib produced increased γ-H2AX labeling. A higher magnification detail was included for this sample. As expected, Capan-1 cells showed a constitutive γ-H2AX labeling that was highly increased by olaparib but was unaffected by **35d** coadministration. (B) The bar graph shows the percentage of γ-H2AX -labeled nuclei counted by two independent observers who analyzed the treated cultures. Data obtained in bxPC-3 cells were statistically evaluated by applying the one-way ANOVA, which indicated a statistically significant difference between the cultures treated with olaparib and those exposed to olaparib + **35d**. (C, D) Evaluation of micronuclei generation in BxPC3 cells treated (72 h) with **35d** and olaparib, given alone or in combination. (C) Representative pictures showing DAPI-stained cell nuclei. White asterisks indicate the presence of micronuclei. (D) The percentage of cells bearing micronuclei was estimated by two independent observers, by analyzing 100–250 cells for each treatment sample. The obtained results were statistically analyzed by applying the one-way ANOVA, which indicated a *p* value of <0.01.

The sustained inhibition of HR in cells should result in increased DNA damage, ultimately leading to mutations and chromosome aberrations; these effects are expected to be further amplified by PARP inhibition. The extent of DNA damage produced in cells treated for 48 h with 20 μM **35d**, administered singularly or in association with 10 μM olaparib, was studied by evidencing nuclear γ-H2AX foci by immunofluorescence. The experiment was performed on both BxPC-3 and Capan-1 cultures. Capan-1 cells are derived from a human pancreatic adenocarcinoma (very similar to BxPC3cells) and are BRCA2-defective.^38^ As a consequence, they do not operate RAD51-BRCA2-dependent HR. The olaparib dose was selected on the basis of previously obtained results with the same cell cultures.^30,31^ Results are reported in Figure 7A,B.

The microscope pictures shown in Figure 7A showed increased evidence of γ-H2AX labeling in nuclei of BxPC-3 cells exposed to the compounds’ association, compared to the labeling observed in cultures treated with olaparib. In Capan-1 cultures, the constitutive γ-H2AX labeling in nuclei appeared more evident than that observed in BxPC-3 cells.

Moreover, in agreement with data showing higher PARPi sensitivity for cells lacking functional HR,^17^ these cultures showed increased nuclear labeling when exposed to olaparib. Notably, this labeling was not further enhanced by **35d** coadministration. The percentage of γ-H2AX-labeled nuclei measured in all treated cultures is shown in the bar graphs of Figure 7B.

The sustained and increased DNA damage produced in BxPC-3 nuclei generates chromosomal aberrations which can be visualized through the presence of small DNA-staining bodies outside the main nucleus (micronuclei). The microscope pictures of Figure 7C show the appearance of this feature in BxPC3 cells exposed for 72 h to 20 μM **35d**, administered alone or in combination with 10 μM olaparib. The percentage of cells bearing micronuclei is reported in the graph of Figure 7D. Notably, cells bearing micronuclei were markedly more frequent in cultures treated with the **35d**/olaparib combination.

Taken together, the results reported in Figures 6 and 7 significantly support the requested mechanism of action for **35d**. Therefore, we conducted further experiments to test whether the **35d**/olaparib combination would induce synthetic lethality.

**Figure 8:**
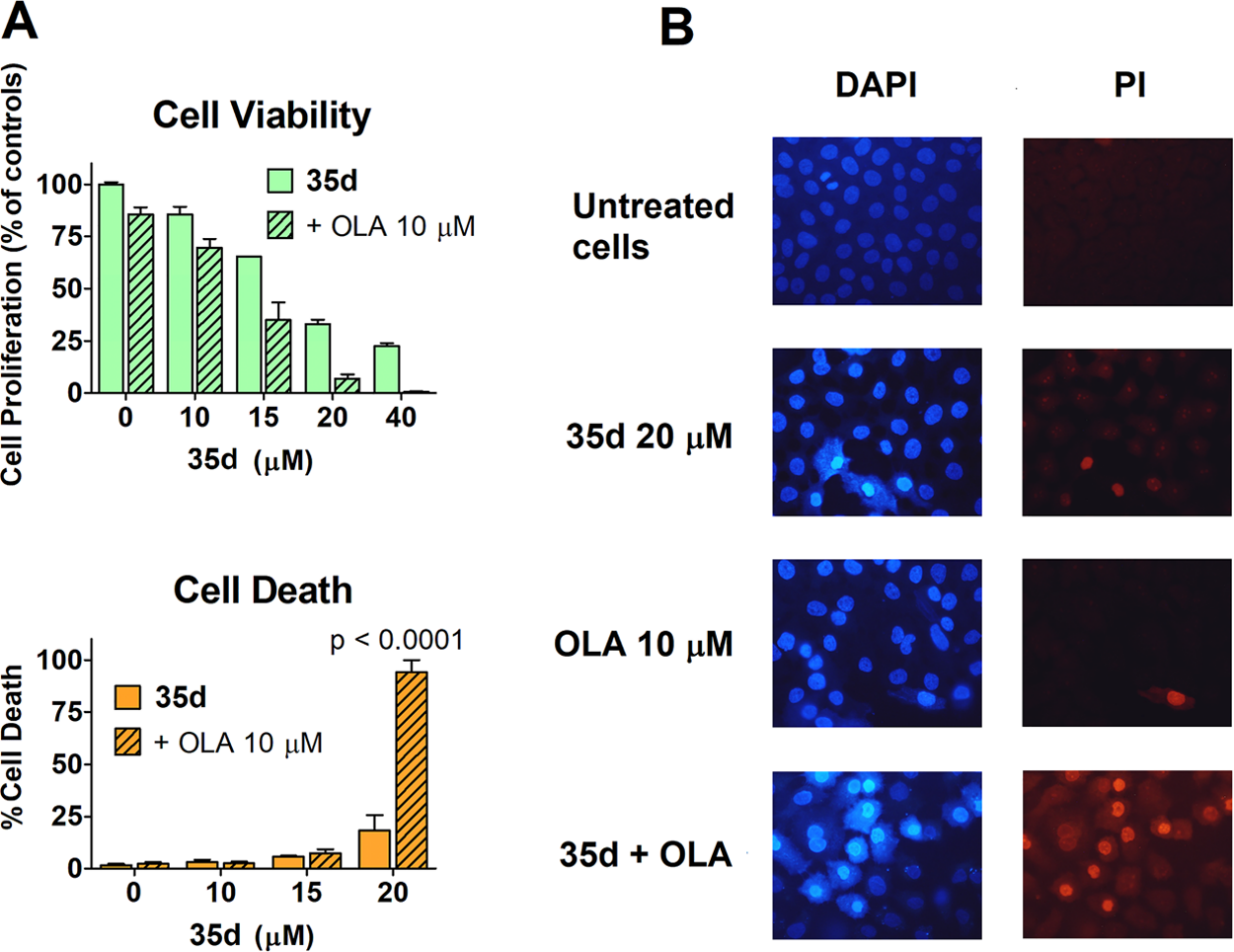
(A) BxPC3 cell viability and death measured after 72 h exposure to **35d** and 10 μM olaparib, given alone or in combination. Data were analyzed by two-way ANOVA using the two treatments (**35d** and olaparib) as variables. In the cell viability experiment, Bonferroni post-test indicated a statistically significant difference produced by olaparib coadministration in all BxPC3 cultures treated with the different **35d** doses, with *p* values ranging from 0.01 (10 μM **35d**) to 0.0001 (15 and 20 μM **35d**). The same analysis was applied to the data from the cell death experiment and indicated that no statistically significant increase in cell death was produced by olaparib when coadministered with 10–15 μM **35d**. In cultures exposed to olaparib + 20 μM **35d** the evidence for cell death was markedly increased and statistically significant, with *p* < 0.0001. (B) After the 72 h treatment, BxPC3 cells were stained with vital dyes. As shown in the microscope pictures, the only culture displaying sharp evidence of cell death was that exposed to the combination of olaparib/20 μM **35d**, as demonstrated by PI nuclear staining.

We simultaneously evaluated cell viability and cell death, measured at 72 h in BxPC3 cells exposed to **35d** alone or in combination with 10 μM olaparib (Figure 8). The statistical analysis (see legend of Figure 8) compared the results for cultures treated with different doses of **35d** or the **35d**/olaparib combination (Figure 8A, upper panel). When applied to the data of the cell viability experiment, this analysis indicated a statistically significant difference produced by **35d** in combination with olaparib in all the treated BxPC3 cultures, with *p* values ranging from 0.01 (10 μM **35d**) to 0.0001 (15 and 20 μM **35d**). When we applied the same analysis to the data of the cell death experiment, we found no statistically significant increase in cell death for olaparib when coadministered with 10–15 μM **35d**. Notably, in cultures exposed to olaparib + 20 μM **35d** (the concentration producing the highest inhibition of HR), the evidence for cell death was markedly increased and statistically significant, with *p* < 0.0001 (Figure 8A, lower panel).

When cell death is a consequence of progressive DNA damage accumulation induced by simultaneous PARP and HR inhibition, we would expect it to emerge gradually over time. To confirm and better characterize the lethality observed in cultures treated with the **35d**/olaparib combination, we therefore considered it inappropriate to conduct a simple evaluation of the commonly used markers (e.g., caspase activation), since these can show very transient changes. Instead, we observed cell morphology and reaction to vital dyes after the 72 h treatment, when the experiment of Figure 8A indicated a significant level of cell death.

Figure 8B shows microscope pictures of BxPC3 cells stained with mixed DAPI and PI. The simultaneous use of these two dyes can demonstrate cell death and indicate the death pattern.^39^ DAPI is cell-permeable and shows nuclear morphology; healthy cells appear to display normal nuclear morphology in the absence of PI staining, since this dye is not cell-permeable. Cells undergoing apoptosis display nuclear condensation, which is indicated by increased DAPI staining. PI staining indicates compromised membrane integrity, which characterizes necrotic cells and late-apoptotic cells maintained in culture.

The microscope pictures show that untreated BxPC3 cells display only a moderate DAPI staining of their nucleus. Nuclear DAPI staining is slightly increased in cells treated with **35d** alone but is strikingly bright only in cells treated with the **35d**/olaparib combination. Furthermore, PI staining appeared only in these cultures, confirming the manifestation of synthetic lethality. The simultaneous marked staining of these cells with both dyes could indicate an apoptotic phenomenon followed by compromised membrane integrity because of cell persistence in culture.

As expected, the sustained and increased DNA damage observed in BxPC3 cells treated with the **35d**/olaparib combination (Figure 7) reproduced the desired mechanism of synthetic lethality (Figure 8). These results are also relevant given the mutated p53 status of BxPC3 cells, which should make them more resistant to mechanisms that induce cell death.

**Figure 9:**
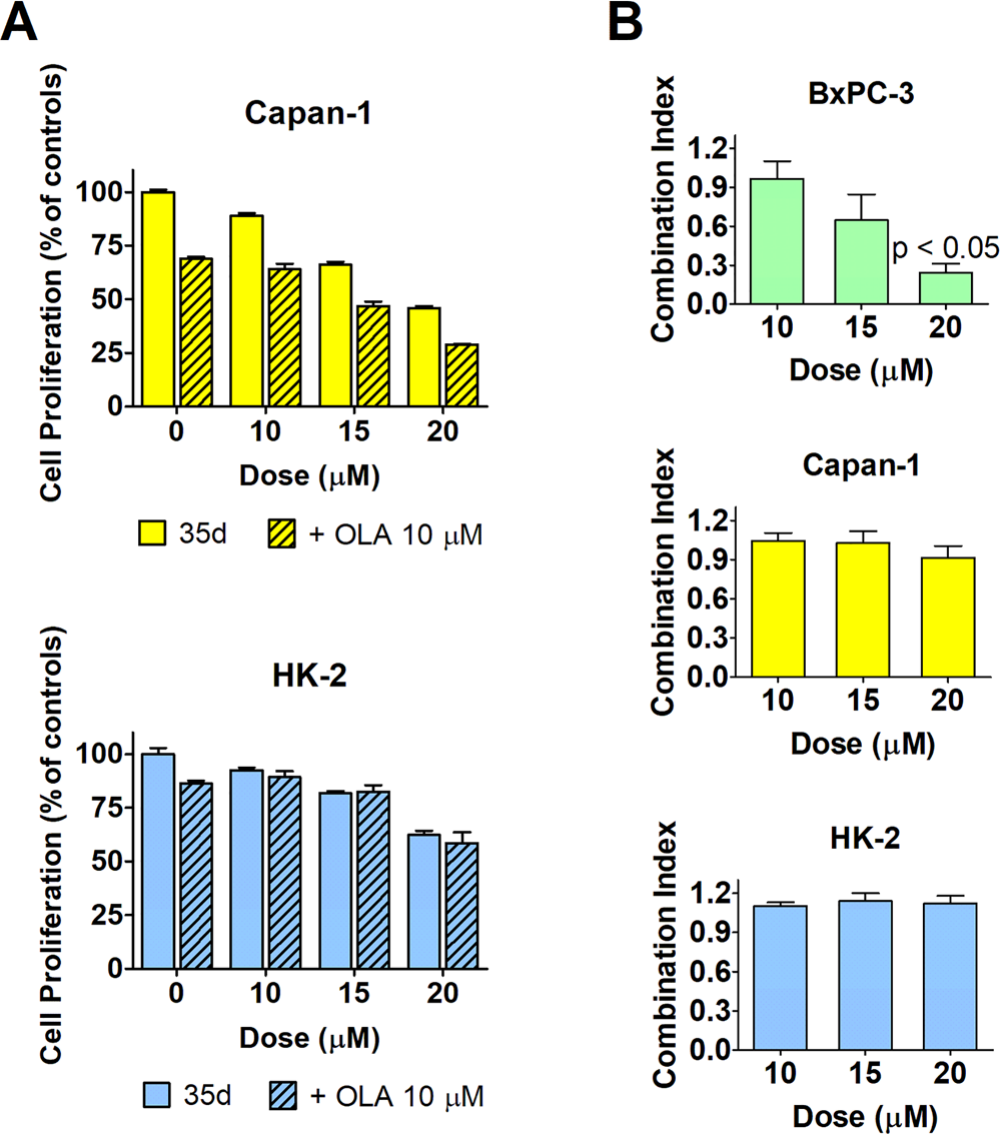
(A) Antiproliferative effect caused by **35d** and its association with olaparib, measured in Capan-1 cells (which do not operate RAD51-BRCA2-dependent HR) and in normal immortalized human renal cells (HK-2). The same procedures described for BxPC3 cells were used here. (B) Combination indexes of the olaparib/**35d** association measured in the three used cell lines, calculated according to the method previously described.^30,31^ For BxPC-3 cells, the data reported in Figure 8A (cell proliferation) were used. Data were analyzed using the column statistics and the one-sample *t* test of Prism 5 software. For BxPC-3 cells, this test showed a statistically significant difference from 0.8 for the combination of olaparib and 20 μΜ **35d**. Values of <0.8 indicate synergism between the two compounds.

Finally, the antiproliferative effect of the **35d**/olaparib combination was also studied on the HR-defective Capan-1 culture and on a non-neoplastic cell line derived from human kidney (HK-2). Moreover, to evaluate the antineoplastic potency of the **35d**/olaparib association, we also calculated the combination index (CI) of the two compounds, using the procedure previously described^30,31^ (Figure 9).

According to this method, CI < 0.8 indicates synergism while a result ranging from 0.8 to 1.2 indicates additive effects. This evaluation (Figure 9) was performed for all the three studied cell cultures; for BxPC-3 cells, the data reported in Figure 8A (cell viability experiment) were used for the calculation of CI. Interestingly, in these cells the potency of the compound combination increased in parallel with the **35d** dose, reaching a statistically significant difference from 0.8 at the 20 μM concentration. The effects observed when 20 μM **35d** is combined with 10 μM olaparib may thus indicate a synergism between the two compounds.

In HK-2 cultures, no dose of **35d** appeared to significantly increase the antiproliferative power of olaparib (two-way ANOVA). In Capan-1 cells, the same statistical evaluation showed significantly increased antiproliferative effects for **35d/**olaparib. However, in these cells, the CI did not show increasing potency of the compound combination with dose escalation. Moreover, at all tested doses, the value of CI was not significantly different from 1, which is the level measured in BxPC3 cells exposed to 10 μM **35d**, a dose that did not relevantly affect (≈10%) HR (Figure 6A). This result further supports the idea that the overadditive effects in the **35d**/olaparib combination arise from and are strictly related to HR inhibition.

Overall, these data supported our working hypothesis that combining a RAD51-BRCA2 small molecule disruptor with olaparib could be a valuable strategy for inducing synthetic lethality in cancer cell lines with fully functional *BRCA* genes and homologous recombination. This includes pancreatic cancer, which is a major unmet need in oncology. We believe that this paradigm could be used to discover innovative anticancer therapies based on other lethal gene pairs using a similar medicinal chemistry strategy.

## Conclusions

Continuing our research line, we described a series of dihydroquinolone pyrazoline derivatives as a new class of RAD51-BRCA2 disruptors. Compound **4d** was identified as a promising hit, and subsequent SAR efforts yielded **35d** with the desired biological profile. As expected, **35d** bound to its target (RAD51) and inhibited the protein–protein interaction between RAD51 and BRCA2. Importantly, it synergized and reproduced the paradigm of synthetic lethality in combination with olaparib in pancreatic cancer cells (BxPC3). These effects were strictly related to the extent of HR inhibition in a dose-dependent trend. This is the most promising achievement of the current investigation and supports our working hypothesis that one can trigger synthetic lethality using only small organic molecules. Interestingly, the observed synthetic lethality was triggered by tackling two biochemically different mechanisms: enzyme inhibition (PARP) and protein–protein disruption (RAD51-BRCA2). This highlights how complex and diverse mechanisms of action can synergistically contribute to the same physiological and, in turn, pharmacological activity. We note, however, that **35d**’s low solubility may affect its metabolic and pharmacokinetic profile (DM/PK), preventing it from being studied further in in vivo cancer models. Structural tuning is therefore required (and currently ongoing) to discover more drug-like dihydroquinolone pyrazoline derivatives.

In conclusion, we have further shown that synthetic lethality may be a suitable framework for discovering innovative anticancer therapies. We are confident that this novel concept will open up several new avenues based on other lethal gene pairs to meet the medical needs in oncology.

## Experimental Section

### Chemistry. General Chemical Methods

Solvents and reagents were obtained from commercial suppliers and used without further purification. If required, solvents were distilled prior to use. Automated column chromatography purifications were conducted using a Teledyne ISCO apparatus (CombiFlash Rf) with prepacked silica gel columns of different sizes (from 4 to 120 g). Mixtures of increasing polarity of cyclohexane and ethyl acetate or dichloromethane and methanol/ethanol were used as eluents. Preparative TLCs were performed using Macherey-Nagel precoated 0.05 mm TLC plates (SIL G-50 UV254). Microwave heating was performed using Explorer-48 positions instrument (CEM). NMR experiments were run on a Bruker Avance III 400 MHz spectrometer (400.13 MHz for ^1^H and 100.62 MHz for ^13^C), equipped with a BBI probe and Z-gradients, or on a Bruker FT NMR Avance III 600-MHz spectrometer (600.130 MHz for ^1^H and 150.903 MHz for ^13^C) equipped with a 5 mm CryoProbe QCI quadruple resonance, a shielded Z-gradient coil, and the automatic sample changer SampleJet NMR system. Spectra were acquired at 300 K, using deuterated dimethylsulfoxide (DMSO-*d*_6_) or deuterated chloroform (CDCl_3_) as solvents. Chemical shifts for ^1^H and ^13^C spectra were recorded in parts per million using the residual nondeuterated solvent as the internal standard (for CDCl_3_, ^1^H 7.26 ppm, ^13^C 77.16 ppm; for DMSO-*d*_6_, ^1^H 2.50 ppm, ^13^C 39.52 ppm). UPLC–MS analyses were run on a Waters ACQUITY UPLC/MS system consisting of an SQD (single quadrupole detector) mass spectrometer equipped with an electrospray ionization interface and a photodiode array detector. The PDA range was 210–400 nm. The analyses were performed on either an ACQUITY UPLC HSS T3 C_18_ column (50 × 2.1 mm i.d., particle size 1.8 μm) with a VanGuard HSS T3 C_18_ precolumn (5 mm × 2.1 mm i.d., particle size 1.8 μm) (log *D* < 1) or an ACQUITY UPLC BEH C18 column (50 mm × 2.1 mm i.d., particle size 1.7 μm) with a VanGuard BEH C_18_ precolumn (5 mm × 2.1 mm i.d., particle size 1.7 μm) (log *D* > 1). The mobile phase was 10 mM NH_4_OAc in H_2_O at pH 5 adjusted with AcOH (A) and 10 mM NH_4_OAc in MeCN–H_2_O (95:5) at pH 5 (B). Electrospray ionization in positive and negative mode was applied in the mass scan range 100–500 Da. Methods and gradients used were the following: *Polar method.* Column: Waters ACQUITY UPLC HSS T3 C_18_, 1.8 μm, 50 mm × 2.1 mm i.d. Precolumn: VanGuard HSS T3 C_18_, 1.8 μm, 5 mm × 2.1 mm i.d. Linear gradient: 0–0.2 min, 0% B; 0.2–2.7 min, 0–50% B; 2.7–2.8 min, 50–100% B; 2.8–3.0 min, 100% B. Flow rate: 0.5 mL/min. *Generic method.* Column: Waters ACQUITY UPLC BEH C_18_, 1.7 μm, 50 mm × 2.1 mm i.d. Linear gradient: 0–0.2 min, 5% B; 0.2–2.7 min, 5–95% B; 2.7–2.8 min, 95–100% B; 2.8–3.0 min, 100% B. Flow rate: 0.5 mL/min. *Apolar method.* Column: Waters ACQUITY UPLC BEH C_18_, 1.7 μm, 50 mm × 2.1 mm i.d. Precolumn: VanGuard BEH C_18_, 1.7 μm, 5 mm × 2.1 mm i.d. Gradient: 0–0.2 min, 50% B; 0.2–2.7 min, 50–100% B; 2.7–3.0 min, 100% B. Flow rate: 0.5 mL/min. Compounds were named using the naming algorithm developed by CambridgeSoft Corporation and used in ChemBioDraw Ultra 15.0. All final compounds displayed ≥95% purity as determined by UPLC/MS analysis. All final synthesized compounds were checked for PAINS compliance.

### General Procedure A for the Synthesis of Quinolin-2(1*H*)-one Acrolyl Intermediates

This procedure has been applied to the preparation of **86b**–**114b** (Scheme 1). In a round-bottomed flask, the appropriate quinolin-2(1*H*)-one (**60a**–**61a**, 1.00 equiv) and potassium hydroxide (25.00 equiv) were stirred in EtOH/H_2_O (4:3 v/v, 0.05 M) at 0 °C for 45 min prior to the addition of an appropriately substituted aryl aldehyde (**62**–**85**, 1.00 equiv). The reaction mixture was stirred overnight as it gradually reached room temperature. The reaction was quenched by slow addition of acetic acid (25.00 equiv). The crude was extracted with DCM/H_2_O (3 × 50 mL), the organic layer was then dried over Na_2_SO_4_, and the solvent was removed under reduced pressure. The desired compound was obtained after purification over silica gel unless otherwise noted.

### General Procedure B for Synthesis of Pyrazol-3-yl-quinolin-2(1*H*)-one Intermediates (Scheme 1)

This procedure has been applied to the preparation of **86c**–**114c**. In a microwaveable vessel, the appropriate quinolin-2(1*H*)-one acrolyl intermediate (**86b**–**114b**, 1.00 equiv) was dissolved in EtOH absolute (0.2 M), and hydrazine monohydrate (2.00 equiv) was added. The mixture was microwaved with stirring for 45 min at 110 °C (200 W). The EtOH was removed under reduced pressure. Crude was purified over silica gel, unless otherwise noted, to afford desired compounds.

### General Procedures C_1_ to Obtain the Final Desired Dihydroquinolone Pyrazoline Derivatives (Scheme 2)

This procedure has been applied to synthesize **4d**, **11d**–**32d**, **52d**–**57d**. In a microwaveable vessel, the appropriate pyrazol-3-ylquinolin-2(1*H*)-one intermediate (**86c**−**90c**, **92c**−**93c**, **95c**−**103c**, **108c**, 1.00 equiv) was dissolved in anhydrous THF (0.5 M). The succinic anhydride **115** (2.00 equiv) was added. The solution was microwaved (200 W) with stirring for 45 min at the appropriate temperature. The THF was removed under reduced pressure, and the organic layers were dissolved in DCM, washed with HCl_aq_ pH 2 (3 × 30 mL), and dried over Na_2_SO_4_. The solvent was removed under reduced pressure, and the crude was purified over silica gel.

### General Procedures C_2_ to Obtain the Final Desired Dihydroquinolone Pyrazoline Derivatives (Scheme 2)

This procedure has been applied to synthesize **5d**, **36d**–**51d**. In a microwaveable vessel, the appropriate pyrazol-3-ylquinolin-2(1*H*)-one intermediate (**86c**−**90c**, **92c**−**93c**, **95c**−**103c**, **108c**, 1.00 equiv) was dissolved in anhydrous THF (0.5 M). The acetic anhydride **116** (2.00 equiv) was added. The solution was microwaved (200 W) with stirring for 45 min at the appropriate temperature. The THF was removed under reduced pressure, and the organic layers were dissolved in DCM, washed with HCl_aq_ pH 2 (3 × 30 mL), and dried over Na_2_SO_4_. The solvent was removed under reduced pressure, and the crude was purified over silica gel.

### General Procedures C_3_ to Obtain the Final Dihydroquinolone Pyrazoline Derivatives (Scheme 2)

This procedure has been applied to synthesize **6d**, **33d**–**35d**. In a round-bottomed flask, propionic acid **117** (1.80 equiv), HOBt (1.80 equiv), and EDCI (1.80 equiv) were stirred in DCM (0.50 M) at room temperature for 1 h. Then a solution of the pyrazol-3-ylquinolin-2(1*H*)-one intermediate **86c**–**89c** (1.00 equiv) and Et_3_N (2.50 equiv) in DCM (0.50 M) was added. The reaction was stirred at room temperature for 3 h. The organic layer was washed with NaHCO_3(aq)_ 1 M (1 × 50 mL), citric acid 10% (1 × 50 mL), and H_2_O (1 × 50 mL), dried over Na_2_SO_4_, and the solvent was removed under reduced pressure. The desired compound was obtained after purification over silica gel unless otherwise noted.

### 4-(3-(6-Chloro-2-oxo-4-phenyl-1,2-dihydroquinolin-3-yl)-5-(4-fluorophenyl)-4,5-dihydro-1*H*-pyrazol-1-yl)-4-oxobutanoic Acid (**4d**)

6-Chloro-3-(5-(4-fluorophenyl)-4,5-dihydro-1*H*-pyrazol-3-yl)-4-phenylquinolin-2(1*H*)-one **86c** (270 mg, 0.65 mmol) and succinic anhydride **115** (130 mg, 1.30 mmol) were microwaved (120 °C, 200 W) according to general procedure C_1_. The crude was purified by flash column chromatography (SiO_2_ gold 24 g; 0–50% EtOH/DCM) to afford the desired **4d** (208 mg, 62% yield). ^1^H NMR (400 MHz, DMSO-*d*_6_) δ 12.38 (s, 1H), 12.02 (s, 1H), 7.64 (dd, *J* = 8.8, 2.4 Hz, 1H), 7.60–7.37 (m, 5H), 7.28 (dt, *J* = 6.8, 2.0 Hz, 1H), 7.04 (t, *J* = 8.9 Hz, 2H), 6.94 (d, *J* = 2.3 Hz, 1H), 6.88–6.76 (m, 2H), 5.32 (dd, *J* = 12.0, 4.5 Hz, 1H), 3.73 (dd, *J* = 18.5, 12.0 Hz, 1H), 2.79 (dd, *J* = 18.4, 4.5 Hz, 1H), 2.48–2.40 (m, 2H), 2.32–2.26 (m, 2H). ^13^C NMR (101 MHz, DMSO-*d*_6_) δ 173.95, 169.09, 160.56, 152.85, 150.38, 137.78, 135.00, 131.68, 129.88, 128.97, 128.88, 128.81, 127.99, 127.90, 126.57, 126.51, 125.07, 121.14, 118.09, 115.67, 115.46, 95.62, 58.69, 45.66, 29.04, 28.69. In agreement with that previously reported by Acker et al.^36^*t*_R_ = 2.04 min (generic method). ESI-MS for C_28_H_21_ClFN_3_O_4_: calculated 517.1, found *m*/*z* 518.4, 520.4 [M + H] ^+^; 516.4, 518.4 [M – H]^−^. UPLC–MS purity (UV at 215 nm) 98%.

### 3-(1-Acetyl-5-(4-fluorophenyl)-4,5-dihydro-1*H*-pyrazol-3-yl)-6-chloro-4-phenylquinolin-2(1*H*)-one (**5d**)

6-Chloro-3-(5-(4-fluorophenyl)-4,5-dihydro-1*H*-pyrazol-3-yl)-4-phenylquinolin-2(1*H*)-one **86c** (200 mg, 0.48 mmol) and acetic anhydride **116** (91 μL, 0.96 mmol) were microwaved (120 °C, 200 W) according to general procedure C_2_. The crude was purified by direct phase flash column chromatography (SiO_2_ gold 24 g, 0–20% MeOH/DCM) to afford the desired **5d** (207 mg, yield 47%). ^1^H NMR (400 MHz, DMSO-*d*_6_) δ 12.38 (s, 1H), 7.64 (dd, *J* = 8.8, 2.4 Hz, 1H), 7.61–7.50 (m, 3H), 7.46 (d, *J* = 8.8 Hz, 1H), 7.41 (dt, *J* = 6.6, 1.9 Hz, 1H), 7.29 (dt, *J* = 6.4, 1.9 Hz, 1H), 7.06 (t, *J* = 8.9 Hz, 2H), 6.94 (d, *J* = 2.3 Hz, 1H), 6.84 (dd, *J* = 8.6, 5.6 Hz, 2H), 5.32 (dd, *J* = 12.0, 4.5 Hz, 1H), 3.73 (dd, *J* = 18.4, 12.1 Hz, 1H), 2.84 (dd, *J* = 18.5, 4.5 Hz, 1H), 1.88 (s, 3H). ^13^C NMR (101 MHz, DMSO-*d*_6_) δ 167.53, 162.80, 160.57, 160.39, 152.67, 150.35, 138.87, 138.84, 137.79, 135.12, 131.64, 129.84, 129.04, 128.96, 128.80, 128.76, 128.00, 127.93, 126.53, 126.49, 125.02, 121.11, 118.09, 115.67, 115.45, 58.49, 45.71, 21.85. *t*_R_ = 2.38 min (generic method). ESI-MS for C_26_H_19_ClFN_3_O_2_: calculated 459.1, found *m*/*z* 460.1, 462.1 [M + H]^+^; 458.1, 460.1 [M – H]^−^. UPLC–MS purity (UV at 215 nm) >99.5%.

### 6-Chloro-3-(5-(4-fluorophenyl)-1-propionyl-4,5-dihydro-1*H*-pyrazol-3-yl)-4-phenylquinolin-2(1*H*)-one (**6d**)

Compound **6d** was synthesized via general procedure C_3_ using **86c** (250 mg, 0.60 mmol) and propionic acid **117** (80.8 μL, 1.08 mmol). Purification was performed by direct phase flash chromatography (SiO_2_ gold 24 g, 0–1% MeOH/DCM) to afford **6d** (60 mg, yield 21%). ^1^H NMR (400 MHz, DMSO-*d*_6_) δ 12.38 (s, 1H), 7.64 (dd, *J* = 2.0, 8.0 Hz, 1H), 7.58–7.50 (m, 3H), 7.45 (d, *J* = 8.8 Hz, 1H), 7.41–7.39 (m, 1H), 7.28 (d, *J* = 5.6 Hz, 1H), 7.06 (t, *J* = 8.8 Hz, 2H), 6.93 (d, *J* = 2.0 Hz, 1H), 6.84 (dd, *J* = 4.0, 8.0, Hz, 2H), 5.32 (dd, *J* = 8.0, 12.0 Hz, 1H), 3.73 (dd, *J* = 12.0, 16.0 Hz, 1H), 2.81 (dd, *J* = 4.0, 16.0 Hz, 1H), 2.30–2.16 (m, 2H), 0.83 (t, *J* = 7.6 Hz, 3H). ^13^C NMR (101 MHz, DMSO-*d*_6_) δ 206.89, 170.93, 162.79, 160.57, 160.38, 152.63, 150.30, 139.00, 138.98, 137.75, 135.12, 131.63, 129.79, 128.97, 128.95, 128.86, 128.80, 127.96, 127.88, 126.53, 126.47, 125.10, 121.11, 118.07, 115.67, 115.46, 58.54, 45.54, 29.44, 9.27. ESI-MS for C_27_H_21_ClFN_3_O_2_: calculated 473.1, found *m*/*z* 474.2, 476.2 [M + H]^+^; 472.3, 474.3 [M – H]^−^. UPLC–MS purity (UV at 215 nm) 99%.

### 3-(1-(3-Aminopropanoyl)-5-(4-fluorophenyl)-4,5-dihydro-1*H*-pyrazol-3-yl)-6-chloro-4-phenylquinolin-2(1*H*)-one (**7d**) (Scheme 3)

In round-bottom flask **119** (200 mg, 0.34 mmol) was treated with 4 M HCl in dioxane (4 mL) and stirred at rt for 15 min. The solvent was removed under reduced pressure. In a round-bottom flask the solid residue (110 mg, 0.21 mmol) was treated with NaOH 0.5 M (420 μL) in EtOAc (5 mL) stirring at rt for 30 min. The mixture was then diluted with further EtOAc and washed twice with H_2_O. The organic layer was dried over Na_2_SO_4_, filtered, and evaporated to dryness. Purification was performed by direct phase flash chromatography (0–10% MeOH/DCM, 0–0.1% NH_4_OH). Yield 50 mg, 50%. ^1^H NMR (400 MHz, DMSO-*d*_6_) δ 7.63 (dd, *J* = 12.0, 4.0 Hz, 1H), 7.57–7.50 (m, 3H), 7.54 (d, *J* = 4.8 Hz, 1H), 7.42–7.40 (m, 1H), 7.26 (d, *J* = 7.2 Hz, 1H), 7.03 (t, *J* = 8.8 Hz, 2H), 6.92 (d, *J* = 2.0 Hz, 1H), 6.81 (dd, *J* = 2.0, 8.0 Hz, 2H), 5.32 (dd, *J* = 2.0, 12.0 Hz, 1H), 3.72 (dd, *J* = 12.0, 20.0 Hz, 1H), 2.76 (dd, *J* = 4.0, 16.0 Hz, 1H), 2.57 (t, *J* = 6.6 Hz, 2H), 2.42–2.28 (m, 2H). ^13^C NMR (101 MHz, DMSO-*d*_6_) δ 169.42, 162.77, 160.56, 160.36, 152.76, 150.29, 138.96, 138.93, 137.78, 135.02, 131.63, 129.86, 128.99, 128.92, 128.89, 128.80, 127.95, 127.86, 126.52, 126.45, 125.09, 121.11, 118.10, 115.66, 115.45, 110.00, 58.53, 45.61, 38.06, 38.04. ESI-MS for C_27_H_22_ClFN_4_O_2_: calculated 488.1, found *m*/*z* 489.4, 491.4 [M + H]^+^, 487.3, 489.4 [M – H]^−^. UPLC–MS purity (UV at 215 nm) >99.5%.

### 4-(3-(6-Chloro-2-oxo-4-phenyl-1,2-dihydroquinolin-3-yl)-5-(4-fluorophenyl)-4,5-dihydro-1*H*-pyrazol-1-yl)-4-oxobutanamide (**8d**) (Scheme 4)

In a round bottomed flask 4-(3-(6-chloro-2-oxo-4-phenyl-1,2-dihydroquinolin-3-yl)-5-(4-fluorophenyl)-4,5-dihydro-1*H*-pyrazol-1-yl)-4-oxobutanoic acid **4d** (258 mg, 0.50 mmol) was dissolved with 3.2 mL of anhydrous DCM, then HATU (285 mg, 0.75 mmol), EDC (144 mg, 0.75 mmol), and 1.8 mL of anhydrous DMF were added. The mixture was stirred at rt for 10 min. Ammonium chloride (144 mg 2.50 mmol) and soon after DIPEA (348 μL, 2.00 mmol) were added. The reaction mixture was thus stirred at rt for 26 h. Purification was performed by direct phase chromatography (SiO_2_ gold 24 g, 2.5–50% EtOH/DCM). Yield 120 mg, 46%. ^1^H NMR (400 MHz, DMSO-*d*_6_) δ 12.38 (s, 1H), 7.64 (dd, *J* = 8.8, 2.4 Hz, 1H), 7.60–7.38 (m, 5H), 7.28 (d, *J* = 7.4 Hz, 1H), 7.19 (s, 1H), 7.04 (dd, *J* = 9.9, 7.7 Hz, 2H), 6.93 (d, *J* = 2.3 Hz, 1H), 6.84–6.78 (m, 2H), 6.68 (s, 1H), 5.31 (dd, *J* = 12.0, 4.5 Hz, 1H), 3.71 (dd, *J* = 18.5, 12.1 Hz, 1H), 2.76 (dd, *J* = 18.4, 4.5 Hz, 1H), 2.54–2.45 (m, 2H), 2.15 (t, *J* = 7.4 Hz, 2H). ^13^C NMR (101 MHz, DMSO-*d*_6_) δ 173.51, 169.56, 160.65, 160.38, 152.73, 150.28, 138.90, 137.94, 135.00, 131.61, 129.94, 129.94, 129.02, 128.99, 128.87, 128.79, 127.99, 127.90, 126.48, 125.10, 121.14, 118.19, 115.66, 115.45, 58.66, 45.64, 29.82, 29.38. *t*_R_ = 2.09 min (generic method). ESI-MS for C_28_H_22_ClFN_4_O_3_: calculated 516.1, found *m*/*z* 517.4, 519.3 [M + H]^+^; 515.4, 517.4 [M – H]^−^. UPLC–MS purity (UV at 215 nm) 99%.

### Methyl 4-(3-(6-Chloro-2-oxo-4-phenyl-1,2-dihydroquinolin-3-yl)-5-(4-fluorophenyl)-4,5-dihydro-1*H*-pyrazol-1-yl)-4-oxobutanoate (**9d**) (Scheme 5)

In a round-bottomed flask, commercially available 4-methoxy-4-oxobutanoic acid **120** (153 mg, 1.16 mmol), 6-chloro-3-(5-(4-fluorophenyl)-4,5-dihydro-1*H*-pyrazol-3-yl)-4-phenylquinolin-2(1*H*)-one **86c** (486 mg, 1.16 mmol), HOBT (173 mg, 1.28 mmol) were dissolved in DCM. Then TEA (355.8 μL, 2.55 mmol) was added, followed by EDCI (245 mg, 1.28 mmol) suspended in DCM. The mixture was stirred overnight at rt. The solvent was removed under vacuum, the residue was dissolved in ethyl acetate and washed with H_2_O, NaHCO_3_ sat. solution and 5% citric acid. The organic phase was dried over Na_2_SO_4_ and evaporated to dryness. The title compound was obtained after purification by direct phase flash column chromatography (0–30% EtOAc/DCM). Yield 256 mg, 42%. ^1^H NMR (400 MHz, DMSO-*d*_6_) δ 12.36 (s, 1H), 7.60 (dd, *J* = 2.2, 9.0 Hz, 1H), 7.53–7.48 (m, 3 H), 7.41 (d, *J* = 8.8 Hz, 1H), 7.37 (dd, *J* = 2.2, 4.6 Hz, 1H), 7.23 (d, *J* = 6.8 Hz, 1H), 7.01 (t, *J* = 8.8 Hz, 2H), 6.89 (d, *J* = 2.0, 1H), 6.77 (dd, *J* = 5.4, 8.6 Hz, 2H), 5.28 (dd, *J* = 12.0, 4.0 Hz, 1H), 3.50 (s, 3H), 3.67 (dd, *J* = 12.0, 18.0 Hz, 1H), 2.75 (dd, *J* = 4.0, 18.0 Hz, 1H). ^13^C NMR (101 MHz, DMSO-*d*_6_) 172.9, 168.8, 160.0, 153.1, 150.1, 139.0, 135.0, 131.9, 129.8, 128.9, 127.9, 126.5, 125.0, 120.9, 118.0, 115.6, 115.4, 58.7, 21.7, 45.0, 28.9, 28.4. UPLC–MS purity (UV at 215 nm) 98%.

### *N*-(3-(3-(6-Chloro-2-oxo-4-phenyl-1,2-dihydroquinolin-3-yl)-5-(4-fluorophenyl)-4,5-dihydro-1*H*-pyrazol-1-yl)-3-oxopropyl)methanesulfonamide (**10d**) (Scheme 6)

In a round-bottom flask, 6-chloro-3-(5-(4-fluorophenyl)-4,5-dihydro-1*H*-pyrazol-3-yl)-4-phenylquinolin-2(1*H*)-one **86c** (500 mg, 1.20 mmol), 3-(methylsulfonamido)propanoic acid **124** (200 mg, 1.20 mmol), HOBT (178 mg, 1.32 mmol) were stirred in DCM prior to the addition of triethylamine (367.7 mL, 2.64 mmol) and EDCI (253 mg, 1.32 mmol) at 0 °C. The reaction was stirred overnight while the mixture gradually reached rt. The solvent was removed under vacuum, and the residue was dissolved in DCM. The organic layer was washed with H_2_O, NaHCO_3_ sat. solution, and 5% citric acid, dried over Na_2_SO_4_, filtered, and evaporated to dryness. The title compound was obtained after purification over direct phase flash column chromatography (0–60% EtOAc/DCM). Yield 100 mg, 15%). ^1^H NMR (400 MHz, DMSO-*d*_6_) δ 12.35 (s, 1H), 7.60 (dd, *J* = 2.0, 8.0 Hz, 1H), 7.55–7.49 (m, 3H), 7.41 (d, *J* = 8.0 Hz, 1H), 7.38 (d, *J* = 6.0 Hz, 1H), 7.24 (d, J = 6.0 Hz, 1H), 7.00 (t, *J* = 8.0 Hz, 2H), 6.89 (d, *J* = 2.0 Hz, 2H), 6.77 (dd, *J* = 4.0, 8.0 Hz, 2H), 5.29 (dd, *J* = 4.0, 12.0 Hz, 1H), 3.69 (dd, *J* = 12.0, 20.0 Hz, 1H), 3.03–2.98 (m, 2H), 2.80 (s, 3H), 2.72 (dd, *J* = 4.0, 20.0 Hz, 1H), 2.51–2.47 (m, 2H). ^13^C NMR (101 MHz, DMSO-*d*_6_) δ 168.01, 162.81, 160.49, 160.40, 153.25, 150.39, 138.71, 137.78, 134.86, 131.68, 129.94, 129.10, 128.94, 128.91, 128.83, 128.02, 127.94, 126.57, 125.01, 121.07, 118.09, 115.69, 115.48, 58.63, 45.67, 38.61, 34.73. UPLC–MS purity (UV at 215 nm) 99%.

### 4-(3-(6-Chloro-2-oxo-4-phenyl-1,2-dihydroquinolin-3-yl)-5-(4-chlorophenyl)-4,5-dihydro-1*H*-pyrazol-1-yl)-4-oxobutanoic Acid (**11d**)

Compound **11d** was synthesized via general procedure C_1_ using **87c** (253 mg, 0.58 mmol) with succinic anhydride **115** (116 mg, 1.16 mmol) (120 °C, 200 W). Purification was performed by direct phase flash chromatography (SiO_2_ gold 24 g, 0–60% EtOH/DCM) to afford **11d** (231 mg, yield 74%). ^1^H NMR (400 MHz, DMSO-*d*_6_) δ 12.38 (s, 1H), 12.03 (s, 1H), 7.64 (dd, *J* = 8.8, 2.4 Hz, 1H), 7.59–7.48 (m, 3H), 7.45 (d, *J* = 8.8 Hz, 1H), 7.41 (dt, *J* = 6.3, 2.0 Hz, 1H), 7.31–7.23 (m, 3H), 6.93 (d, *J* = 2.4 Hz, 1H), 6.83–6.76 (m, 2H), 5.32 (dd, *J* = 12.0, 4.6 Hz, 1H), 3.74 (dd, *J* = 18.5, 12.1 Hz, 1H), 2.78 (dd, *J* = 18.5, 4.6 Hz, 1H), 2.48–2.41 (m, 2H), 2.29 (t, *J* = 7.1 Hz, 2H). In agreement with that previously reported by Acker et al.^36^*t*_R_ = 2.16 min (generic method). ESI-MS for C_28_H_21_Cl_2_N_3_O_4_: calculated 533.1, found *m*/*z* 534.4, 536.4, 538.3 [M + H]^+^; 532.4, 534.4, 536.4 [M – H]^−^. UPLC–MS purity (UV at 215 nm) 99%.

### 4-(5-(4-Bromophenyl)-3-(6-chloro-2-oxo-4-phenyl-1,2-dihydroquinolin-3-yl)-4,5-dihydro-1*H*-pyrazol-1-yl)-4-oxobutanoic Acid (**12d**)

Compound **12d** was synthesized via general procedure C_1_ using **88c** (330 mg, 0.69 mmol) with succinic anhydride **115** (138 mg, 1.38 mmol) (120 °C, 200 W). Purification was performed by direct phase flash chromatography (SiO_2_ gold 24 g, 0–50% EtOH/DCM) to afford **12d** (275 mg, yield 68%). ^1^H NMR (400 MHz, DMSO-*d*_6_) δ 12.38 (s, 1H), 12.03 (s, 1H), 7.63 (dd, *J* = 8.8, 2.3 Hz, 1H), 7.59–7.47 (m, 3H), 7.45 (d, *J* = 8.8 Hz, 1H), 7.40 (d, *J* = 8.4 Hz, 2H), 7.27 (dd, *J* = 6.5, 1.8 Hz, 1H), 6.93 (d, *J* = 2.3 Hz, 1H), 6.73 (d, *J* = 8.5 Hz, 2H), 5.30 (dd, *J* = 12.0, 4.6 Hz, 1H), 3.74 (dd, *J* = 18.5, 12.1 Hz, 1H), 2.77 (dd, *J* = 18.5, 4.6 Hz, 1H), 2.48–2.36 (m, 2H), 2.28 (t, *J* = 7.1 Hz, 2H). In agreement with that previously reported by Acker et al.^36^*t*_R_ = 2.18 min (generic method). ESI-MS for C_28_H_21_BrClN_3_O_4_: calculated 577.0, found *m*/*z* 578.2, 580.2, 582.3 [M + H]^+^; 576.1, 578.1, 580.0 [M – H]^−^. UPLC–MS purity (UV at 215 nm) >99.5%.

### 4-(3-(6-Chloro-2-oxo-4-phenyl-1,2-dihydroquinolin-3-yl)-5-(4-methoxyphenyl)-4,5-dihydro-1*H*-pyrazol-1-yl)-4-oxobutanoic Acid (**13d**)

Compound **13d** was synthesized via general procedure C_1_ using **89c** (320 mg, 0.74 mmol) with succinic anhydride **115** (148 mg, 1.48 mmol) (120 °C, 200 W). Purification was performed by direct phase flash chromatography (SiO_2_ gold 24 g, 0–15% EtOH/DCM) to afford **13d** (339 mg, yield 86%). ^1^H NMR (400 MHz, DMSO-*d*_6_) δ 12.19 (s, 1H), 7.57 (dd, *J* = 8.8, 2.3 Hz, 1H), 7.51 (m, 3H), 7.42 (d, *J* = 8.8 Hz, 1H), 7.36–7.31 (m, 1H), 7.27–7.21 (m, 1H), 7.02 (dd, *J* = 8.9, 5.9 Hz, 3H), 6.90 (d, *J* = 2.3 Hz, 1H), 6.80 (d, *J* = 8.7 Hz, 2H), 4.52 (td, *J* = 10.4, 2.9 Hz, 1H), 3.73 (s, 3H), 3.18 (dd, *J* = 16.4, 10.9 Hz, 1H), 2.58–2.52 (m, 5H). In agreement with that previously reported by Acker et al.^36^*t*_R_ = 1.99 min (generic method). ESI-MS for C_29_H_24_ClN_3_O_5_: calculated 529.1, found *m*/*z* 530.5, 532.4 [M + H]^+^; 528.4, 530.4 [M – H]^−^. UPLC–MS purity (UV at 215 nm) >99.5%.

### 4-(5-(4-(*tert*-Butyl)phenyl)-3-(6-chloro-2-oxo-4-phenyl-1,2-dihydroquinolin-3-yl)-4,5-dihydro-1*H*-pyrazol-1-yl)-4-oxobutanoic Acid (**14d**)

Compound **14d** was synthesized via general procedure C_1_ using **90c** (124 mg, 0.27 mmol) with succinic anhydride **115** (54 mg, 0.54 mmol) (120 °C, 200 W). Purification was performed by direct phase flash chromatography (SiO_2_ gold 24 g, 0–20% EtOH/DCM) to afford **14d** (121 mg, yield 81%). ^1^H NMR (400 MHz, DMSO-*d*_6_) δ 12.27 (d, *J* = 78.0 Hz, 1H), 12.08 (s, 1H), 7.64 (dd, *J* = 8.8, 2.3 Hz, 1H), 7.59–7.47 (m, 3H), 7.47–7.43 (m, 1H), 7.40 (dd, *J* = 4.6, 2.1 Hz, 1H), 7.28 (d, *J* = 7.5 Hz, 1H), 7.23 (d, *J* = 8.3 Hz, 2H), 6.93 (d, *J* = 2.3 Hz, 1H), 6.73 (d, *J* = 8.3 Hz, 2H), 5.26 (dd, *J* = 11.9, 4.5 Hz, 1H), 3.69 (dt, *J* = 24.9, 12.5 Hz, 1H), 2.85 (ddd, *J* = 23.0, 18.2, 4.3 Hz, 1H), 2.47 (dd, *J* = 6.7, 4.0 Hz, 2H), 2.36–2.22 (m, 2H), 1.27 (s, 9H). ^13^C NMR (101 MHz, DMSO-*d*_6_) δ 173.52, 168.56, 160.09, 152.41, 149.87, 149.19, 139.24, 137.30, 134.59, 131.15, 129.30, 128.57, 128.47, 128.33, 126.06, 126.03, 125.13, 125.09, 124.67, 120.69, 117.60, 58.62, 45.26, 34.12, 31.11, 28.57, 28.21, 26.32. *t*_R_ = 2.39 min (generic method). ESI-MS for C_32_H_30_ClN_3_O_4_: calculated 555.2, found *m*/*z* 556.5, 558.4 [M + H]^+^; 554.4, 556.4 [M – H]^−^. UPLC–MS purity (UV at 215 nm) 97%.

### 4-(3-(6-Chloro-2-oxo-4-phenyl-1,2-dihydroquinolin-3-yl)-5-(3-fluoro-4-methoxyphenyl)-4,5-dihydro-1*H*-pyrazol-1-yl)-4-oxobutanoic Acid (**15d**)

Compound **15d** was synthesized via general procedure C_1_ using **91c** (184 mg, 0.41 mmol) with succinic anhydride **115** (82 mg, 0.82 mmol) (120 °C, 200 W). Purification was performed by direct phase flash chromatography (SiO_2_ gold 24 g, 0–10% EtOH/DCM) to afford **15d** (115 mg, yield 51%). ^1^H NMR (400 MHz, DMSO-*d*_6_) δ 12.38 (s, 1H), 12.06 (s, 1H), 7.64 (dd, *J* = 8.8, 2.4 Hz, 1H), 7.59–7.43 (m, 4H), 7.43–7.38 (m, 1H), 7.29 (dd, *J* = 7.1, 1.9 Hz, 1H), 7.01 (t, *J* = 8.8 Hz, 1H), 6.94 (d, *J* = 2.4 Hz, 1H), 6.69–6.61 (m, 2H), 5.27 (dd, *J* = 12.0, 4.5 Hz, 1H), 3.82 (s, 3H), 3.70 (dd, *J* = 18.5, 12.0 Hz, 1H), 2.82 (dd, *J* = 18.5, 4.5 Hz, 1H), 2.46 (dd, *J* = 14.3, 7.0 Hz, 2H), 2.29 (t, *J* = 7.2 Hz, 2H). ^13^C NMR (101 MHz, DMSO-*d*_6_) δ 173.96, 169.09, 160.59, 152.89, 150.37, 146.62, 137.79, 135.43, 135.05, 131.68, 129.76, 129.01, 128.94, 128.84, 128.74, 126.57, 125.06, 123.56, 122.25, 121.13, 118.10, 114.22, 113.68, 113.50, 58.52, 56.48, 45.52, 29.02, 28.67. *t*_R_ = 2.00 min (generic method). ESI-MS for C_29_H_23_ClFN_3_O_5_: calculated 547.1, found *m*/*z* 548.3, 550.3 [M + H]^+^; 546.3, 548.2 [M – H]^−^. UPLC–MS purity (UV at 215 nm) >99.5%.

### 4-(3-(6-Chloro-2-oxo-4-phenyl-1,2-dihydroquinolin-3-yl)-5-(4-(trifluoromethyl)phenyl)-4,5-dihydro-1*H*-pyrazol-1-yl)-4-oxobutanoic Acid (**16d**)

Compound **16d** was synthesized via general procedure C_1_ using **92c** (309 mg, 0.66 mmol) with succinic anhydride **115** (132 mg, 1.32 mmol) (120 °C, 200 W). Purification was performed by direct phase flash chromatography (SiO_2_ gold 40 g, 0–10% EtOH/DCM) to afford **16d** (266 mg, yield 71%). ^1^H NMR (400 MHz, DMSO-*d*_6_) δ 12.38 (s, 1H), 12.05 (s, 1H), 7.63 (dd, *J* = 8.8, 2.4 Hz, 1H), 7.60–7.39 (m, 7H), 7.31–7.19 (m, 1H), 7.00 (d, *J* = 8.1 Hz, 2H), 6.93 (d, *J* = 2.3 Hz, 1H), 5.42 (dd, *J* = 12.1, 4.7 Hz, 1H), 3.78 (dd, *J* = 18.5, 12.2 Hz, 1H), 2.80 (dd, *J* = 18.5, 4.7 Hz, 1H), 2.59–2.38 (m, 2H), 2.36–2.26 (m, 2H). ^13^C NMR (101 MHz, DMSO-*d*_6_) δ 173.45, 168.78, 160.05, 152.42, 150.01, 146.65, 137.33, 134.55, 131.24, 129.42, 128.54, 128.44, 128.38, 128.35, 127.85, 127.53, 127.22, 126.20, 126.12, 126.04, 125.54, 125.39, 125.35, 124.45, 122.83, 120.63, 117.63, 58.51, 45.05, 28.49, 28.18, 26.32. In agreement with that previously reported by Acker et al.^36^*t*_R_ = 2.22 min (generic method). ESI-MS for C_29_H_21_ClF_3_N_3_O_4_: calculated 567.1, found *m*/*z* 568.5, 570.4, [M + H]^+^; 566.4, 568.4 [M – H]^−^. UPLC–MS purity (UV at 215 nm) 99%.

### 4-(3-(6-Chloro-2-oxo-4-phenyl-1,2-dihydroquinolin-3-yl)-5-(furan-2-yl)-4,5-dihydro-1*H*-pyrazol-1-yl)-4-oxobutanoic Acid (**17d**)

Compound **17d** was synthesized via general procedure C_1_ using **93c** (201 mg, 0.51 mmol) with succinic anhydride **115** (102 mg, 1.02 mmol) (120 °C, 200 W). Purification was performed by direct phase flash chromatography (SiO_2_ gold 24 g, 0–10% EtOH/DCM) to afford **17d** (226 mg, yield 90%). ^1^H NMR (400 MHz, DMSO-*d*_6_) δ 12.38 (s, 1H), 12.06 (s, 1H), 7.64 (dd, *J* = 8.8, 2.3 Hz, 1H), 7.54–7.43 (m, 5H), 7.32 (t, *J* = 6.4 Hz, 2H), 6.96 (d, *J* = 2.3 Hz, 1H), 6.33 (dd, *J* = 3.1, 1.9 Hz, 1H), 5.99 (d, *J* = 3.2 Hz, 1H), 5.39 (dd, *J* = 11.8, 4.5 Hz, 1H), 3.58 (dd, *J* = 18.2, 11.9 Hz, 1H), 3.17 (dd, *J* = 18.2, 4.6 Hz, 1H), 2.41–2.18 (m, 4H). ^13^C NMR (101 MHz, DMSO-*d*_6_) δ 173.58, 173.49, 168.82, 160.10, 152.84, 152.49, 150.04, 142.05, 137.37, 134.77, 131.19, 128.81, 128.78, 128.29, 128.26, 128.24, 126.12, 126.07, 124.33, 120.63, 117.60, 110.33, 106.27, 56.01, 52.62, 41.72, 28.84, 28.52, 28.15. In agreement with that previously reported by Acker et al.^36^*t*_R_ = 1.90 min (generic method). ESI-MS for C_26_H_20_ClN_3_O_5_: calculated 489.1, found *m*/*z* 490.4, 492.4 [M + H]^+^; 488.4, 490.4 [M – H]^−^. UPLC–MS purity (UV at 215 nm) >99.5%.

### 4-(3-(6-Chloro-2-oxo-4-phenyl-1,2-dihydroquinolin-3-yl)-5-(4′-fluoro-[1,1′-biphenyl]-4-yl)-4,5-dihydro-1*H*-pyrazol-1-yl)-4-oxobutanoic Acid (**18d**)

Compound **18d** was synthesized via general procedure C_1_ using **94c** (171 mg, 0.35 mmol) with succinic anhydride **115** (70 mg, 0.70 mmol) (120 °C, 200 W). Purification was performed by direct phase flash chromatography (SiO_2_ gold 24 g, 0–10% EtOH/DCM) to afford **18d** (167 mg, yield 80%). ^1^H NMR (400 MHz, DMSO-*d*_6_) δ 12.38 (s, 1H), 12.08 (s, 1H), 7.71–7.61 (m, 3H), 7.55 (ddt, *J* = 10.6, 9.5, 4.0 Hz, 3H), 7.50–7.40 (m, 4H), 7.33–7.25 (m, 3H), 6.94 (d, *J* = 2.3 Hz, 1H), 6.87 (d, *J* = 8.3 Hz, 2H), 5.35 (dd, *J* = 12.0, 4.6 Hz, 1H), 3.77 (dd, *J* = 18.5, 12.1 Hz, 1H), 2.84 (dd, *J* = 18.4, 4.7 Hz, 1H), 2.57–2.44 (m, 2H), 2.30 (t, *J* = 6.8 Hz, 2H). ^13^C NMR (101 MHz, DMSO-*d*_6_) δ 173.63, 173.50, 168.63, 163.05, 160.62, 160.11, 152.46, 149.94, 141.37, 137.96, 137.31, 136.34, 136.31, 134.60, 131.19, 129.41, 128.64, 128.56, 128.47, 128.33, 126.73, 126.08, 124.64, 120.69, 117.62, 115.78, 115.57, 58.66, 45.22, 28.96, 28.60, 28.23. *t*_R_ = 2.36 min (generic method). ESI-MS for C_34_H_25_ClFN_3_O_4_: calculated 593.1, found *m*/*z* 594.1, 596.1 [M + H]^+^, 592.2, 594.2 [M – H]^−^. UPLC–MS purity (UV at 215 nm) >99.5%.

### 4-(3-(6-Chloro-2-oxo-4-phenyl-1,2-dihydroquinolin-3-yl)-5-(4′-chloro-[1,1′-biphenyl]-4-yl)-4,5-dihydro-1*H*-pyrazol-1-yl)-4-oxobutanoic Acid (**19d**)

Compound **19d** was synthesized via general procedure C_1_ using **95c** (227 mg, 0.44 mmol) with succinic anhydride **115** (88 mg, 0.88 mmol) (120 °C, 200 W). Purification was performed by direct phase flash chromatography (SiO_2_ gold 24 g, 0–10% EtOH/DCM) to afford **19d** (205 mg, yield 77%). ^1^H NMR (400 MHz, DMSO-*d*_6_) δ 12.38 (s, 1H), 12.04 (s, 1H), 7.67 (d, *J* = 8.6 Hz, 2H), 7.63 (dd, *J* = 8.8, 2.4 Hz, 1H), 7.59–7.48 (m, 7H), 7.47–7.39 (m, 2H), 7.29 (dd, *J* = 6.5, 2.0 Hz, 1H), 6.94 (d, *J* = 2.4 Hz, 1H), 6.88 (d, *J* = 8.3 Hz, 2H), 5.35 (dd, *J* = 12.0, 4.6 Hz, 1H), 3.77 (dd, *J* = 18.5, 12.1 Hz, 1H), 2.84 (dd, *J* = 18.5, 4.6 Hz, 1H), 2.50 (m, 2H), 2.29 (t, *J* = 6.8 Hz, 2H). ^13^C NMR (101 MHz, DMSO-*d*_6_) δ 173.58, 173.49, 168.64, 160.10, 152.44, 149.94, 141.77, 138.63, 137.62, 137.31, 134.60, 132.26, 131.18, 129.40, 128.84, 128.52, 128.45, 128.37, 128.32, 126.72, 126.14, 126.10, 126.05, 124.62, 120.68, 117.61, 58.66, 45.21, 28.60, 28.23. *t*_R_ = 2.51 min (generic method). ESI-MS for C_34_H_25_Cl_2_N_3_O_4_: calculated 609.1, found *m*/*z* 610.0, 612.0, 614.2 [M + H]^+^; 608.1, 610.1, 612.0 [M – H]^−^. UPLC–MS purity (UV at 215 nm) >99.5%.

### 4-[3-[4-(4-4-(5-(4′-Bromo-[1,1′-biphenyl]-4-yl)-3-(6-chloro-2-oxo-4-phenyl-1,2-dihydroquinolin-3-yl)-4,5-dihydro-1*H*-pyrazol-1-yl)-4-oxobutanoic Acid (**20d**)

Compound **20d** was synthesized via general procedure C_1_ using **96c** (214 mg, 0.38 mmol) with succinic anhydride **115** (76 mg, 0.76 mmol) (120 °C, 200 W). Purification was performed by direct phase flash chromatography (SiO_2_ gold 24 g, 0–20% EtOH/DCM) to afford **20d** (197 mg, yield 79%). ^1^H NMR (400 MHz, DMSO-*d*_6_) δ 12.37 (s, 1H), 12.08 (s, 1H), 7.68–7.38 (m, 12H), 7.30–7.26 (m, 1H), 6.94 (d, *J* = 2.4 Hz, 1H), 6.89 (d, *J* = 8.4 Hz, 2H), 5.35 (dd, *J* = 12.0, 4.6 Hz, 1H), 3.78 (dd, *J* = 18.5, 12.1 Hz, 1H), 2.84 (dd, *J* = 18.5, 4.7 Hz, 1H), 2.58–2.43 (m, *J* = 3.7 Hz, 2H), 2.30 (t, *J* = 6.9 Hz, 2H). ^13^C NMR (101 MHz, DMSO-*d*_6_) δ 173.50, 168.66, 160.11, 152.44, 149.95, 141.81, 139.00, 137.67, 137.31, 134.60, 131.76, 131.18, 129.40, 128.71, 128.52, 128.45, 128.33, 126.68, 126.16, 126.11, 126.05, 124.62, 120.84, 120.68, 117.61, 58.67, 45.20, 28.61, 28.24. *t*_R_ = 2.52 min (generic method). ESI-MS for C_34_H_25_BrClN_3_O_4_: calculated 653.1, found *m*/*z* 653.9, 655.9, 657.9 [M + H]^+^; 652.0, 654.0, 655.8 [M – H]^−^. UPLC–MS purity (UV at 215 nm) >99.5%.

### 4-(3-(6-Chloro-2-oxo-4-phenyl-1,2-dihydroquinolin-3-yl)-5-(4′-methoxy-[1,1′-biphenyl]-4-yl)-4,5-dihydro-1*H*-pyrazol-1-yl)-4-oxobutanoic Acid (**21d**)

Compound **21d** was synthesized via general procedure C_1_ using **97c** (545 mg, 1.10 mmol) with succinic anhydride **115** (220 mg, 2.20 mmol) (120 °C, 200 W). Purification was performed by direct phase flash chromatography (SiO_2_ gold 40 g, 0–10% EtOH/DCM) to afford **21d** (359 mg, yield 55%). ^1^H NMR (400 MHz, DMSO-*d*_6_) δ 12.38 (s, 1H), 12.03 (s, 1H), 7.67–7.50 (m, 6H), 7.44 (dt, *J* = 7.7, 5.9 Hz, 4H), 7.29 (dd, *J* = 6.7, 2.1 Hz, 1H), 7.02 (d, *J* = 8.8 Hz, 2H), 6.94 (d, *J* = 2.3 Hz, 1H), 6.84 (d, *J* = 8.3 Hz, 2H), 5.33 (dd, *J* = 12.0, 4.6 Hz, 1H), 3.79 (s, 4H), 2.84 (dd, *J* = 18.4, 4.6 Hz, 1H), 2.30 (t, *J* = 6.9 Hz, 2H). ^13^C NMR (101 MHz, DMSO-*d*_6_) δ 173.51, 168.62, 160.12, 158.85, 152.44, 149.92, 140.63, 138.67, 137.31, 134.60, 132.23, 131.17, 129.40, 128.54, 128.44, 128.32, 127.68, 126.23, 126.10, 126.00, 124.66, 120.69, 117.61, 114.34, 58.70, 55.14, 45.24, 28.62, 28.25. *t*_R_ = 2.30 min (generic method). ESI-MS for C_35_H_28_ClN_3_O_5_: calculated 605.2, found *m*/*z* 606.0, 608.0 [M + H]^+^; 604.0, 606.0 [M – H]^−^. UPLC–MS purity (UV at 215 nm) >99.5%.

### 4-(3-(6-Chloro-2-oxo-4-phenyl-1,2-dihydroquinolin-3-yl)-5-(1-methyl-1*H*-indol-5-yl)-4,5-dihydro-1*H*-pyrazol-1-yl)-4-oxobutanoic Acid (**22d**)

Compound **22d** was synthesized via general procedure C_1_ using **98c** (153 mg, 0.34 mmol) with succinic anhydride **115** (68 mg, 0.68 mmol) (120 °C, 200 W). Purification was performed by direct phase flash chromatography (SiO_2_ gold 24 g, 0–20% EtOH/DCM) to afford **22d** (150 mg, yield 79%). ^1^H NMR (400 MHz, DMSO-*d*_6_) δ 12.37 (s, 1H), 12.18 (s, 1H), 7.63 (dd, *J* = 8.8, 2.3 Hz, 1H), 7.60–7.55 (m, 2H), 7.55–7.49 (m, 1H), 7.44 (dd, *J* = 7.7, 4.3 Hz, 2H), 7.31–7.20 (m, 3H), 6.98 (d, *J* = 1.7 Hz, 1H), 6.93 (d, *J* = 2.3 Hz, 1H), 6.61 (dd, *J* = 8.5, 1.7 Hz, 1H), 6.34 (dd, *J* = 3.0, 0.8 Hz, 1H), 5.35 (dd, *J* = 12.0, 4.6 Hz, 1H), 3.84–3.69 (m, 4H), 2.85 (dd, *J* = 18.4, 4.6 Hz, 1H), 2.53–2.45 (m, 2H), 2.26 (t, *J* = 6.9 Hz, 2H). ^13^C NMR (101 MHz, DMSO-*d*_6_) δ 173.72, 173.52, 168.43, 160.17, 152.27, 149.80, 137.29, 135.60, 134.66, 133.04, 131.11, 129.90, 129.47, 128.52, 128.43, 128.25, 127.73, 126.06, 124.84, 120.75, 118.98, 117.58, 117.22, 109.64, 100.28, 59.51, 45.76, 32.46, 29.17, 28.70, 28.27. *t*_R_ = 2.09 min (generic method). ESI-MS for C_31_H_25_ClN_4_O_4_: calculated 552.2, found *m*/*z* 553.1, 555.2 [M + H]^+^; 551.2, 553.2 [M – H]^−^. UPLC–MS purity (UV at 215 nm) >99.5%.

### 4-(3-(6-Chloro-2-oxo-4-phenyl-1,2-dihydroquinolin-3-yl)-5-(1-ethyl-1*H*-indol-5-yl)-4,5-dihydro-1*H*-pyrazol-1-yl)-4-oxobutanoic Acid (**23d**)

Compound **23d** was synthesized via general procedure C_1_ using **99c** (153 mg, 0.33 mmol) with succinic anhydride **115** (66 mg, 0.66 mmol) (120 °C, 200 W). Purification was performed by direct phase flash chromatography (SiO_2_ gold 24 g, 0–10% EtOH/DCM) to afford **23d** (92 mg, yield 49%). ^1^H NMR (400 MHz, DMSO-*d*_6_) δ 12.37 (s, 1H), 12.02 (s, 1H), 7.63 (dd, *J* = 8.8, 2.4 Hz, 1H), 7.58 (dd, *J* = 5.5, 3.3 Hz, 2H), 7.52 (ddd, *J* = 8.9, 5.2, 3.1 Hz, 1H), 7.44 (dd, *J* = 8.7, 4.4 Hz, 2H), 7.35 (d, *J* = 3.1 Hz, 1H), 7.29 (dd, *J* = 8.5, 2.2 Hz, 2H), 6.95 (dd, *J* = 15.5, 2.0 Hz, 2H), 6.60 (dd, *J* = 8.5, 1.7 Hz, 1H), 6.34 (d, *J* = 3.1 Hz, 1H), 5.34 (dd, *J* = 12.0, 4.7 Hz, 1H), 4.17 (q, *J* = 7.2 Hz, 2H), 3.77 (dd, *J* = 18.4, 12.1 Hz, 1H), 2.86 (dd, *J* = 18.4, 4.7 Hz, 1H), 2.48–2.42 (m, 2H), 2.26 (t, *J* = 6.9 Hz, 2H), 1.34 (t, *J* = 7.2 Hz, 3H). ^13^C NMR (101 MHz, DMSO-*d*_6_) δ 173.53, 168.45, 160.17, 152.28, 149.80, 137.29, 134.66, 134.56, 133.01, 131.11, 129.45, 128.53, 128.42, 128.28, 127.89, 126.05, 124.84, 120.74, 118.93, 117.58, 117.32, 109.62, 100.49, 59.51, 45.75, 40.26, 28.72, 28.30, 15.53. *t*_R_ = 2.20 min (generic method). ESI-MS for C_32_H_27_ClN_4_O_4_: calculated 566.2, found *m*/*z* 567.2, 569.2 [M + H]^+^; 565.3, 567.3 [M – H]^−^. UPLC–MS purity (UV at 215 nm) >99.5%.

### 4-(3-(6-Chloro-2-oxo-4-phenyl-1,2-dihydroquinolin-3-yl)-5-(1-ethyl-1*H*-indazol-5-yl)-4,5-dihydro-1*H*-pyrazol-1-yl)-4-oxobutanoic Acid (**24d**)

Compound **24d** was synthesized via general procedure C_1_ using **100c** (196 mg, 0.42 mmol) with succinic anhydride **115** (84 mg, 0.84 mmol) (120 °C, 200 W). Purification was performed by direct phase flash chromatography (SiO_2_ gold 24 g, 0–10% EtOH/DCM) to afford **24d** (177 mg, yield 74%). ^1^H NMR (400 MHz, DMSO-*d*_6_) δ 12.39 (s, 1H), 12.03 (s, 1H), 7.98 (s, 1H), 7.64 (dd, *J* = 8.8, 2.4 Hz, 1H), 7.59 (dd, *J* = 5.4, 3.5 Hz, 2H), 7.52 (dd, *J* = 10.0, 6.6 Hz, 2H), 7.48–7.43 (m, 2H), 7.29 (d, *J* = 7.5 Hz, 1H), 7.14 (d, *J* = 1.5 Hz, 1H), 6.94 (d, *J* = 2.3 Hz, 1H), 6.83 (dd, *J* = 8.7, 1.6 Hz, 1H), 5.42 (dd, *J* = 12.0, 4.6 Hz, 1H), 4.42 (q, *J* = 7.2 Hz, 2H), 3.80 (dd, *J* = 18.5, 12.1 Hz, 1H), 2.85 (dd, *J* = 18.5, 4.6 Hz, 1H), 2.54–2.48 (m, 2H), 2.28 (t, *J* = 6.9 Hz, 2H), 1.38 (t, *J* = 7.2 Hz, 3H). ^13^C NMR (101 MHz, DMSO-*d*_6_) δ 173.99, 169.05, 160.62, 152.86, 150.36, 138.51, 137.79, 135.07, 134.85, 132.81, 131.65, 130.00, 129.09, 128.97, 128.93, 128.78, 126.57, 126.54, 125.22, 124.57, 123.78, 121.19, 118.09, 117.79, 110.22, 59.57, 45.96, 43.53, 29.13, 28.72, 15.43. *t*_R_ = 1.95 min (generic method). ESI-MS for C_31_H_26_ClN_5_O_4_: calculated 567.2, found *m*/*z* 568.5, 570.4 [M + H]^+^, 566.4, 568.4 [M – H]^−^. UPLC–MS purity (UV at 215 nm) >99.5%.

### 4-(3-(6-Chloro-2-oxo-4-phenyl-1,2-dihydroquinolin-3-yl)-5-(2-ethyl-2*H*-indazol-5-yl)-4,5-dihydro-1*H*-pyrazol-1-yl)-4-oxobutanoic Acid (**25d**)

Compound **25d** was synthesized via general procedure C_1_ using **101c** (198 mg, 0.42 mmol) with succinic anhydride **115** (84 mg, 0.84 mmol) (120 °C, 200 W). Purification was performed by direct phase flash chromatography (SiO_2_ gold 24 g, 0–20% EtOH/DCM) to afford **25d** (120 mg, yield 50%). ^1^H NMR (400 MHz, DMSO-*d*_6_) δ 12.37 (s, 1H), 12.04 (s, 1H), 8.29 (s, 1H), 7.66–7.55 (m, 3H), 7.51 (td, *J* = 6.6, 5.5, 3.3 Hz, 1H), 7.44 (dd, *J* = 10.0, 6.8 Hz, 3H), 7.28 (d, *J* = 7.5 Hz, 1H), 7.07 (d, *J* = 1.6 Hz, 1H), 6.93 (d, *J* = 2.4 Hz, 1H), 6.61 (dd, *J* = 8.9, 1.7 Hz, 1H), 5.35 (dd, *J* = 12.0, 4.6 Hz, 1H), 4.43 (q, *J* = 7.3 Hz, 2H), 3.77 (dd, *J* = 18.4, 12.1 Hz, 1H), 2.82 (dd, *J* = 18.4, 4.6 Hz, 1H), 2.54–2.44 (m, 2H), 2.28 (t, *J* = 6.9 Hz, 2H), 1.49 (t, *J* = 7.2 Hz, 3H). ^13^C NMR (101 MHz, DMSO-*d*_6_) δ 173.50, 168.55, 160.12, 152.37, 149.84, 147.32, 137.29, 134.57, 134.39, 131.15, 129.53, 128.57, 128.49, 128.42, 128.28, 126.07, 126.04, 124.75, 123.57, 123.00, 120.94, 120.72, 117.60, 117.32, 116.68, 59.31, 47.68, 45.24, 28.67, 28.26, 15.84. *t*_R_ = 1.85 min (generic method). ESI-MS for C_31_H_26_ClN_5_O_4_: calculated 567.2, found *m*/*z* 568.4, 570.4 [M + H]^+^, 566.5, 568.4 [M – H]^−^. UPLC–MS purity (UV at 215 nm) >99.5%.

### 4-(3-(6-Chloro-2-oxo-4-phenyl-1,2-dihydroquinolin-3-yl)-5-(1-propyl-1*H*-indazol-5-yl)-4,5-dihydro-1*H*-pyrazol-1-yl)-4-oxobutanoic Acid (**26d**)

Compound **26d** was synthesized via general procedure C_1_ using **102c** (176 mg, 0.36 mmol) with succinic anhydride **115** (72 mg, 0.72 mmol) (120 °C, 200 W). Purification was performed by direct phase flash chromatography (SiO_2_ gold 24 g, 0–25% EtOH/DCM) to afford **26d** (178 mg, yield 86%). ^1^H NMR (400 MHz, DMSO-*d*_6_) δ 12.36 (s, 1H), 12.05 (s, 1H), 7.97 (d, *J* = 0.9 Hz, 1H), 7.62 (dd, *J* = 8.8, 2.4 Hz, 1H), 7.57 (qd, *J* = 4.0, 1.0 Hz, 2H), 7.53–7.47 (m, 2H), 7.47–7.41 (m, 2H), 7.30–7.25 (m, 1H), 7.16–7.11 (m, 1H), 6.94 (d, *J* = 2.3 Hz, 1H), 6.84 (dd, *J* = 8.8, 1.6 Hz, 1H), 5.42 (dd, *J* = 12.0, 4.6 Hz, 1H), 4.33 (t, *J* = 6.9 Hz, 2H), 3.78 (dd, *J* = 18.5, 12.0 Hz, 1H), 2.85 (dd, *J* = 18.5, 4.6 Hz, 1H), 2.54–2.48 (m, 2H), 2.28 (t, *J* = 6.9 Hz, 2H), 1.83 (h, *J* = 7.2 Hz, 2H), 0.83 (t, *J* = 7.4 Hz, 3H). ^13^C NMR (101 MHz, DMSO-*d*_6_) δ 173.53, 168.61, 160.15, 152.41, 149.89, 138.59, 137.31, 134.60, 134.31, 132.32, 131.17, 129.50, 128.60, 128.49, 128.43, 128.32, 126.11, 126.06, 124.73, 124.13, 123.19, 120.71, 117.62, 117.28, 109.80, 59.09, 49.64, 45.49, 28.67, 28.26, 22.83, 11.17. *t*_R_ = 2.06 min (generic method). ESI-MS for C_32_H_28_ClN_5_O_4_: calculated 581.2, found *m*/*z* 582.1, 584.1 [M + H]^+^, 580.4, 582.0 [M – H]^−^. UPLC–MS purity (UV at 215 nm) >99.5%.

### 4-(3-(6-Chloro-2-oxo-4-phenyl-1,2-dihydroquinolin-3-yl)-5-(2-propyl-2*H*-indazol-5-yl)-4,5-dihydro-1*H*-pyrazol-1-yl)-4-oxobutanoic Acid (**27d**)

Compound **27d** was synthesized via general procedure C_1_ using **103c** (173 mg, 0.36 mmol) with succinic anhydride **115** (72 mg, 0.72 mmol) (120 °C, 200 W). Purification was performed by direct phase flash chromatography (SiO_2_ gold 24 g, 0–20% EtOH/DCM) to afford **27d** (162 mg, yield 78%). ^1^H NMR (400 MHz, DMSO-*d*_6_) δ 12.37 (s, 2H), 8.28 (d, *J* = 0.9 Hz, 1H), 7.63–7.54 (m, 3H), 7.50 (td, *J* = 6.6, 5.5, 3.3 Hz, 1H), 7.47–7.40 (m, 3H), 7.30–7.24 (m, 1H), 7.08 (t, *J* = 1.2 Hz, 1H), 6.93 (d, *J* = 2.4 Hz, 1H), 6.62 (dd, *J* = 8.9, 1.7 Hz, 1H), 5.36 (dd, *J* = 12.0, 4.6 Hz, 1H), 4.35 (t, *J* = 6.9 Hz, 2H), 3.77 (dd, *J* = 18.5, 12.0 Hz, 1H), 2.83 (dd, *J* = 18.5, 4.6 Hz, 1H), 2.54–2.50 (m, 2H), 2.29 (t, *J* = 6.9 Hz, 2H), 1.96–1.87 (m, 2H), 0.83 (t, *J* = 7.4 Hz, 3H). ^13^C NMR (101 MHz, DMSO-*d*_6_) δ 173.57, 168.64, 160.16, 152.44, 149.89, 147.41, 137.31, 134.59, 134.40, 131.16, 129.56, 128.60, 128.52, 128.43, 128.31, 126.13, 126.07, 124.77, 123.75, 123.60, 120.86, 120.74, 117.62, 117.39, 116.71, 59.36, 54.27, 45.27, 28.73, 28.32, 23.44, 10.91. *t*_R_ = 2.00 min (generic method). ESI-MS for C_32_H_28_ClN_5_O_4_: calculated 581.2, found *m*/*z* 582.2, 584.3 [M + H]^+^, 580.3, 582.4 [M – H]^−^. UPLC–MS purity (UV at 215 nm) >99.5%.

### 4-(3-(6-Chloro-2-oxo-4-phenyl-1,2-dihydroquinolin-3-yl)-5-(1-cyclohexyl-1*H*-indazol-5-yl)-4,5-dihydro-1*H*-pyrazol-1-yl)-4-oxobutanoic Acid (**28d**)

Compound **28d** was synthesized via general procedure C_1_ using **104c** (164 mg, 0.31 mmol) with succinic anhydride **115** (62 mg, 0.62 mmol) (120 °C, 200 W). Purification was performed by direct phase flash chromatography (SiO_2_ gold 24 g, 0–12% EtOH/DCM) to afford **28d** (169 mg, yield 87%). ^1^H NMR (400 MHz, DMSO-*d*_6_) δ 12.38 (s, 1H), 12.07 (s, 1H), 7.96 (s, 1H), 7.65–7.49 (m, 5H), 7.48–7.41 (m, 2H), 7.28 (dd, *J* = 7.5, 1.8 Hz, 1H), 7.11 (d, *J* = 1.6 Hz, 1H), 6.94 (d, *J* = 2.3 Hz, 1H), 6.84 (dd, *J* = 8.8, 1.6 Hz, 1H), 5.41 (dd, *J* = 12.0, 4.6 Hz, 1H), 4.54 (tt, *J* = 10.0, 5.5 Hz, 1H), 3.80 (dd, *J* = 18.5, 12.1 Hz, 1H), 2.85 (dd, *J* = 18.5, 4.7 Hz, 1H), 2.53–2.46 (m, 2H), 2.28 (t, *J* = 6.9 Hz, 2H), 2.01–1.81 (m, 6H), 1.70 (dt, *J* = 12.8, 3.4 Hz, 1H), 1.58–1.43 (m, 2H), 1.26 (qt, *J* = 12.7, 3.5 Hz, 1H). ^13^C NMR (101 MHz, DMSO-*d*_6_) δ 173.74, 173.51, 168.59, 160.16, 152.36, 149.90, 137.70, 137.31, 134.64, 134.41, 132.08, 131.16, 129.51, 128.59, 128.48, 128.45, 128.30, 126.12, 126.07, 124.73, 123.92, 123.18, 120.71, 117.61, 117.24, 109.77, 59.12, 56.63, 45.50, 32.34, 28.67, 28.26, 25.08. *t*_R_ = 2.33 min (generic method). ESI-MS for C_35_H_32_ClN_5_O_4_: calculated 621.2, found *m*/*z* 622.3, 624.2 [M + H]^+^, 620.3, 622.4 [M – H]^−^. UPLC–MS purity (UV at 215 nm) >99.5%.

### 4-(3-(6-Chloro-2-oxo-4-phenyl-1,2-dihydroquinolin-3-yl)-5-(2-cyclohexyl-2*H*-indazol-5-yl)-4,5-dihydro-1*H*-pyrazol-1-yl)-4-oxobutanoic Acid (**29d**)

Compound **29d** was synthesized via general procedure C_1_ using **105c** (100 mg, 0.19 mmol) with succinic anhydride **115** (38 mg, 0.38 mmol) (120 °C, 200 W). Purification was performed by direct phase flash chromatography (SiO_2_ gold 24 g, 0–20% EtOH/DCM) to afford **29d** (113 mg, yield 95%). ^1^H NMR (400 MHz, DMSO-*d*_6_) δ 12.37 (s, 1H), 12.10 (s, 1H), 8.29 (d, *J* = 1.0 Hz, 1H), 7.65–7.55 (m, 3H), 7.53–7.48 (m, 1H), 7.44 (t, *J* = 8.7 Hz, 3H), 7.30–7.24 (m, 1H), 7.10–7.05 (m, 1H), 6.93 (d, *J* = 2.4 Hz, 1H), 6.60 (dd, *J* = 9.0, 1.7 Hz, 1H), 5.35 (dd, *J* = 12.0, 4.6 Hz, 1H), 4.44 (tt, *J* = 11.2, 3.8 Hz, 1H), 3.77 (dd, *J* = 18.4, 12.1 Hz, 1H), 2.82 (dd, *J* = 18.4, 4.7 Hz, 1H), 2.55–2.46 (m, 2H), 2.28 (t, *J* = 6.9 Hz, 2H), 2.14–2.02 (m, 2H), 1.95–1.81 (m, 4H), 1.70 (dd, *J* = 12.6, 3.7 Hz, 1H), 1.45 (qt, *J* = 12.5, 3.4 Hz, 2H), 1.25 (tdd, *J* = 15.6, 11.7, 7.8 Hz, 1H). ^13^C NMR (101 MHz, DMSO-*d*_6_) δ 173.72, 173.52, 168.56, 160.14, 152.38, 149.87, 146.88, 137.30, 134.58, 134.35, 131.15, 129.52, 128.59, 128.51, 128.44, 128.30, 126.09, 126.05, 124.76, 123.49, 121.57, 120.73, 120.62, 117.61, 117.47, 116.76, 61.63, 59.36, 45.25, 33.29, 29.13, 28.69, 28.28, 24.92, 24.89. *t*_R_ = 2.20 min (generic method). ESI-MS for C_35_H_32_ClN_5_O_4_: calculated 621.2, found *m*/*z* 622.2, 624.2 [M + H]^+^, 620.3, 622.3 [M – H]^−^. UPLC–MS purity (UV at 215 nm) 99%.

### 4-(3-(6-Chloro-2-oxo-4-phenyl-1,2-dihydroquinolin-3-yl)-5-(4-(1-ethyl-1*H*-indazol-5-yl)phenyl)-4,5-dihydro-1*H*-pyrazol-1-yl)-4-oxobutanoic Acid (**30d**)

Compound **30d** was synthesized via general procedure C_1_ using **106c** (170 mg, 0.31 mmol) with succinic anhydride **115** (62 mg, 0.62 mmol) (120 °C, 200 W). Purification was performed by direct phase flash chromatography (SiO_2_ gold 24 g, 0–20% EtOH/DCM) to afford **30d** (163 mg, yield 81%). ^1^H NMR (400 MHz, DMSO-*d*_6_) δ 12.38 (s, 1H), 12.07 (s, 1H), 8.10 (d, *J* = 0.8 Hz, 1H), 7.99 (d, *J* = 1.6 Hz, 1H), 7.74 (d, *J* = 8.8 Hz, 1H), 7.67 (dd, *J* = 8.8, 1.7 Hz, 1H), 7.63 (dd, *J* = 8.8, 2.4 Hz, 1H), 7.60–7.51 (m, 5H), 7.48–7.40 (m, 2H), 7.30 (dd, *J* = 5.9, 1.8 Hz, 1H), 6.94 (d, *J* = 2.3 Hz, 1H), 6.89 (d, *J* = 8.3 Hz, 2H), 5.35 (dd, *J* = 12.0, 4.6 Hz, 1H), 4.46 (q, *J* = 7.2 Hz, 2H), 3.78 (dd, *J* = 18.5, 12.1 Hz, 1H), 2.86 (dd, *J* = 18.5, 4.6 Hz, 1H), 2.59–2.44 (m, 2H), 2.31 (t, *J* = 6.8 Hz, 2H), 1.41 (t, *J* = 7.2 Hz, 3H). ^13^C NMR (101 MHz, DMSO-*d*_6_) δ 173.53, 168.65, 160.13, 152.47, 149.95, 140.77, 139.50, 138.23, 137.32, 134.63, 132.92, 132.54, 131.18, 129.43, 128.55, 128.47, 128.34, 126.90, 126.11, 126.07, 125.52, 124.67, 124.25, 120.70, 118.46, 117.62, 110.02, 58.72, 45.26, 43.15, 28.64, 28.26, 14.94. *t*_R_ = 2.24 min (generic method). ESI-MS for C_37_H_30_ClN_5_O_4_: calculated 643.2, found *m*/*z* 644.1, 646.1 [M + H]^+^, 642.2, 644.1 [M – H]^−^. UPLC–MS purity (UV at 215 nm) >99.5%.

### 4-(3-(6-Chloro-2-oxo-4-phenyl-1,2-dihydroquinolin-3-yl)-5-(4-(2-ethyl-2*H*-indazol-5-yl)phenyl)-4,5-dihydro-1*H*-pyrazol-1-yl)-4-oxobutanoic Acid (**31d**)

Compound **31d** was synthesized via general procedure C_1_ using **107c** (117 mg, 0.21 mmol) with succinic anhydride **115** (42 mg, 0.42 mmol) (120 °C, 200 W). Purification was performed by direct phase flash chromatography (SiO_2_ gold 24 g, 0–20% EtOH/DCM) to afford **31d** (66 mg, yield 49%). ^1^H NMR (400 MHz, DMSO-*d*_6_) δ 12.38 (s, 1H), 12.15 (s, 1H), 8.42 (d, *J* = 0.9 Hz, 1H), 7.92 (t, *J* = 1.3 Hz, 1H), 7.68 (d, *J* = 8.9 Hz, 1H), 7.63 (dd, *J* = 8.8, 2.4 Hz, 1H), 7.60–7.50 (m, 6H), 7.48–7.41 (m, 2H), 7.30 (dd, *J* = 6.1, 1.9 Hz, 1H), 6.94 (d, *J* = 2.3 Hz, 1H), 6.87 (d, *J* = 8.4 Hz, 2H), 5.35 (dd, *J* = 12.0, 4.6 Hz, 1H), 4.47 (q, *J* = 7.3 Hz, 2H), 3.78 (dd, *J* = 18.5, 12.1 Hz, 1H), 2.86 (dd, *J* = 18.5, 4.7 Hz, 1H), 2.57–2.43 (m, 2H), 2.30 (t, *J* = 6.9 Hz, 2H), 1.52 (t, *J* = 7.3 Hz, 3H). ^13^C NMR (101 MHz, DMSO-*d*_6_) δ 173.63, 173.52, 168.64, 160.13, 152.47, 149.95, 147.41, 140.72, 137.33, 132.68, 128.56, 128.47, 128.35, 126.74, 126.11, 126.06, 126.02, 125.23, 124.68, 123.68, 121.91, 120.71, 117.89, 117.63, 117.45, 58.72, 47.80, 28.93, 28.63, 28.26, 15.76. *t*_R_ = 2.10 min (generic method). ESI-MS for C_37_H_30_ClN_5_O_4_: calculated 643.2, found *m*/*z* 644.2, 646.2 [M + H]^+^, 642.3, 644.3 [M – H]^−^. UPLC–MS purity (UV at 215 nm) >99.5%.

### 4-(3-(6-Chloro-2-oxo-4-phenyl-1,2-dihydroquinolin-3-yl)-5-(4-(1-propyl-1*H*-pyrazol-4-yl)phenyl)-4,5-dihydro-1*H*-pyrazol-1-yl)-4-oxobutanoic Acid (**32d**)

Compound **32d** was synthesized via general procedure C_1_ using **108c** (155 mg, 0.30 mmol) with succinic anhydride **115** (60 mg, 0.60 mmol) (120 °C, 200 W). Purification was performed by direct phase flash chromatography (SiO_2_ gold 24 g, 0–30% EtOH/DCM) to afford **32d** (189 mg, quantitative yield). ^1^H NMR (400 MHz, DMSO-*d*_6_) δ 12.37 (s, 1H), 12.10 (s, 1H), 8.14 (s, 1H), 7.84 (s, 1H), 7.62 (dd, *J* = 8.8, 2.4 Hz, 1H), 7.59–7.50 (m, 3H), 7.48–7.36 (m, 4H), 7.31–7.25 (m, 1H), 6.94 (d, *J* = 2.3 Hz, 1H), 6.77 (d, *J* = 8.0 Hz, 2H), 5.28 (dd, *J* = 12.0, 4.6 Hz, 1H), 4.07 (t, *J* = 7.0 Hz, 2H), 3.75 (dd, *J* = 18.4, 12.1 Hz, 1H), 2.82 (dd, *J* = 18.5, 4.6 Hz, 1H), 2.48–2.41 (m, 2H), 2.28 (t, *J* = 6.9 Hz, 2H), 1.96–1.87 (m, 2H), 0.85 (t, *J* = 7.3 Hz, 3H). ^13^C NMR (101 MHz, DMSO-*d*_6_) δ 173.50, 168.56, 160.12, 152.39, 149.89, 139.75, 137.30, 135.88, 134.62, 131.56, 131.16, 129.42, 128.53, 128.44, 128.29, 126.88, 126.09, 125.98, 124.96, 124.68, 121.24, 120.70, 117.60, 58.75, 53.00, 45.20, 28.63, 28.26, 23.17, 10.93. *t*_R_ = 2.09 min (generic method). ESI-MS for C_34_H_30_ClN_5_O_4_: calculated 607.2, found *m*/*z* 608.0, 610.0 [M + H]^+^, 606.1, 608.0 [M – H]^−^. UPLC–MS purity (UV at 215 nm) 99%.

### 6-Chloro-3-(5-(4-chlorophenyl)-1-propionyl-4,5-dihydro-1*H*-pyrazol-3-yl)-4-phenylquinolin-2(1*H*)-one (**33d**)

Compound **33d** was synthesized via general procedure C_3_ using **87c** (200 mg, 0.46 mmol) and propionic acid **117** (60.5 μL, 0.83 mmol). Purification was performed by direct phase flash chromatography (SiO_2_ gold 24 g, 0–2% MeOH/DCM) to afford **33d** (76 mg, yield 34%). ^1^H NMR (400 MHz, DMSO-*d*_6_) δ 12.38 (s, 1H), 7.63 (dd, *J* = 9.0, 2.4 Hz, 1H), 7.54–7.49 (m, 3H), 7.44 (d, *J* = 8.8 Hz, 1H), 7.41–7.39 (m, 1H), 7.30–7.27 (m, 3H), 6.92 (d, *J* = 2.4 Hz, 1H), 6.80 (d, *J* = 8.8 Hz, 2H), 5.31 (dd, *J* = 12.2, 4.6 Hz, 1H), 3.74 (dd, *J* = 18.4, 12.0 Hz, 1H), 2.80 (dd, *J* = 18.4, 4.0 Hz, 1H), 2.31–2.14 (m, 2H), 0.82 (t, *J* = 7.2 Hz, 3H). ^13^C NMR (101 MHz, DMSO-*d*_6_) δ 206.88, 170.96, 160.55, 152.65, 150.33, 141.76, 137.75, 135.13, 132.03, 131.64, 129.80, 128.95, 128.87, 128.82, 128.79, 127.81, 126.55, 126.48, 125.04, 121.10, 118.07, 110.00, 58.61, 45.45, 31.11, 27.06, 9.26. ESI-MS for C_27_H_21_Cl_2_N_3_O_2_: calculated 489.1, found *m*/*z* 490.2, 492.1, 494.1 [M + H]^+^; 488.3, 490.3, 492.1 [M – H]^−^. UPLC–MS purity (UV at 215 nm) 98%.

### 3-(5-(4-Bromophenyl)-1-propionyl-4,5-dihydro-1*H*-pyrazol-3-yl)-6-chloro-4-phenylquinolin-2(1*H*)-one (**34d**)

Compound **34d** was synthesized via general procedure C_3_ using **88c** (150 mg, 0.31 mmol) and propionic acid **117** (41.8 μL, 0.56 mmol). Purification was performed by direct phase flash chromatography (SiO_2_ gold 24 g, 0–0.5% MeOH/DCM) to afford **34d** (60 mg, yield 36%). ^1^H NMR (400 MHz, DMSO-*d*_6_) δ 12.41 (s, 1H), 7.64 (dd, *J* = 12.8, 1.6 Hz, 1H), 7.55–7.50 (m, 3H), 7.46–7.41 (m, 4H), 7.28 (d, *J* = 6.8 Hz, 1H), 6.93 (d, *J* = 2.0 Hz, 1H), 6.75 (d, *J* = 8.0 Hz, 2H), 5.30 (dd, *J* = 12.4, 4.0 Hz, 1H), 3.75 (dd, *J* = 18.4, 12.0 Hz, 1H), 2.80 (dd, *J* = 18.2, 4.6 Hz, 1H), 2.31–2.14 (m, 2H), 0.83 (t, *J* = 7.6 Hz, 3H). ^13^C NMR (101 MHz, DMSO-*d*_6_) δ 206.78, 171.02, 160.54, 152.60, 150.33, 142.18, 137.77, 135.16, 131.72, 131.61, 129.79, 128.97, 128.92, 128.84, 128.78, 128.18, 126.56, 126.49, 125.03, 121.13, 120.51, 118.06, 58.73, 45.40, 31.08, 27.05, 9.23. ESI-MS for C_27_H_21_BrClN_3_O_2_: calculated 533.0, found *m*/*z* 534.2, 536.1, 538.1 [M + H]^+^; 532.2, 534.1, 536.2 [M – H]^−^. UPLC–MS purity (UV at 215 nm) 97%.

### 6-Chloro-3-(5-(4-methoxyphenyl)-1-propionyl-4,5-dihydro-1*H*-pyrazol-3-yl)-4-phenylquinolin-2(1*H*)-one (**35d**)

Compound **35d** was synthesized via general procedure C_3_ using **89c** (150 mg, 0.36 mmol) and propionic acid **117** (48.5 μL, 0.65 mmol). The crude was purified by direct phase flash column chromatography (SiO_2_ gold 24 g; 0–2% MeOH/DCM), to afford **35d** (100 mg, yield 59%). ^1^H NMR (400 MHz, DMSO*-d*_*6*_) δ 12.36 (s, 1H), 7.64 (dd, *J* = 8.6, 2.4 Hz, 1H), 7.56–7.52 (m, 3H), 7.45 (d, *J* = 8.4 Hz, 1H), 7.40–7.38 (m, 1H), 7.29–7.27 (m, 1H), 6.92 (d, *J* = 2.0 Hz, 1H), 6.75 (dd, *J* = 15.2, 9.2 Hz, 4H), 5.24 (dd, *J* = 12.0, 4.8 Hz, 1H), 3.72 (s, 3H), 3.70 (dd, *J* = 16.0, 12.0 Hz, 1H), 2.81 (dd, *J* = 20.0, 4.4 Hz, 1H), 2.29–2.12 (m, 2H), 0.82 (t, *J* = 8.0 Hz, 3H). ^13^C NMR (101 MHz, DMSO*-d*_*6*_) δ 170.80, 160.60, 158.67, 152.56, 150.23, 137.74, 135.16, 134.94, 131.58, 129.76, 129.03, 128.89, 128.84, 128.77, 127.20, 126.51, 126.47, 125.22, 121.15, 118.05, 114.15, 58.73, 55.49, 45.59, 27.12, 9.31. ESI-MS for C_28_H_24_ClN_3_O_3_: calculated 485.1, found *m*/*z* 486.4, 488.4 [M + H]+; 484.3, 486.3 [M – H]-. UPLC–MS purity (UV at 215 nm) 98%.

### 3-(1-Acetyl-5-(4-chlorophenyl)-4,5-dihydro-1*H*-pyrazol-3-yl)-6-chloro-4-phenylquinolin-2(1*H*)-one (**36d**)

Compound **36d** was synthesized via general procedure C_2_ using **87c** (210 mg, 0.48 mmol) with acetic anhydride **116** (888 μL, 0.96 mmol) (165 °C, 200 W). Purification was performed by direct phase flash chromatography (SiO_2_ gold 24 g, 0–1.5% MeOH/DCM) to afford **36d** (200 mg, yield 94%). ^1^H NMR (400 MHz, DMSO-*d*_6_) δ 12.41 (s, 1H), 7.64 (dt, *J* = 8.0, 1.2 Hz, 1H), 7.58–7.53 (m, 3H), 7.44 (d, *J* = 8.0 Hz, 1H), 7.41–7.39 (m, 1H), 7.30–7.28 (m, 3H), 6.93 (d, *J* = 0.8 Hz, 1H), 6.80 (d, *J* = 7.6 Hz, 2H), 5.32 (dd, *J* = 12.0, 8.0 Hz, 1H), 3.74 (dd, *J* = 20.0, 12.0 Hz, 1H), 2.82 (dd, *J* = 16.0, 4.0 Hz, 1H), 1.87 (s, 3H). ^13^C NMR (101 MHz, DMSO-*d*_6_) δ 167.56, 160.54, 152.67, 150.39, 141.60, 137.75, 135.11, 132.05, 131.65, 129.86, 129.01, 128.96, 128.81, 128.75, 127.83, 126.55, 126.49, 124.96, 121.10, 118.06, 58.56, 45.62, 21.82. ESI-MS for C_26_H_19_Cl_2_N_3_O_2_: calculated 475.1, found *m*/*z* 476.3, 478.4, 480.4 [M + H]^+^; 474.3, 476.3, 478.1 [M – H]^−^. UPLC–MS purity (UV at 215 nm) 99%.

### 3-(1-Acetyl-5-(4-bromophenyl)-4,5-dihydro-1*H*-pyrazol-3-yl)-6-chloro-4-phenylquinolin-2(1*H*)-one (**37d**)

Compound **37d** was synthesized via general procedure C_2_ using **88c** (100 mg, 0.21 mmol) with acetic anhydride **116** (40 μL, 0.42 mmol) (165 °C, 200 W). Purification was performed by direct phase flash chromatography (SiO_2_ gold 12 g, 0–2% MeOH/DCM) to afford **37d** (80 mg, yield 73%). ^1^H NMR (400 MHz, DMSO-*d*_6_) δ 12.39 (s, 1H), 7.64 (dd, *J* = 8.0, 4.0 Hz, 1H), 7.57–7.53 (m, 3H), 7.45–7.39 (m, 4H), 7.28 (dd, *J* = 6.0, 1.6 Hz, 1H), 6.93 (d, *J* = 2.0 Hz, 1H), 6.75 (d, *J* = 8.8 Hz, 2H), 5.30 (dd, *J* = 12.0, 4.4 Hz, 1H), 3.74 (dd, *J* = 18.5, 12.0 Hz, 1H), 2.82 (dd, *J* = 4.0, 20.0 Hz, 1H), 1.86 (s, 3H). ^13^C NMR (101 MHz, DMSO-*d*_6_) δ 167.58, 160.54, 152.64, 150.39, 142.02, 137.78, 135.15, 131.72, 131.63, 129.83, 129.03, 128.94, 128.78, 128.74, 128.20, 126.56, 126.50, 124.96, 121.13, 120.54, 118.06, 58.67, 45.58, 21.78. ESI-MS for C_26_H_19_BrClN_3_O_2_: calculated 519.0, found *m*/*z* 520.1, 522.1, 524.1 [M + H]^+^; 518.1, 520.1, 522.2 [M – H]^−^. UPLC–MS purity (UV at 215 nm) 99%.

### 3-(1-Acetyl-5-(4-methoxyphenyl)-4,5-dihydro-1*H*-pyrazol-3-yl)-6-chloro-4-phenylquinolin-2(1*H*)-one (**38d**)

Compound **38d** was synthesized via general procedure C_2_ using **89c** (65 mg, 0.15 mmol) with acetic anhydride **116** (28 μL, 0.30 mmol) (165 °C, 200 W). Purification was performed by direct phase flash chromatography (SiO_2_ gold 12 g, 0–1.5% MeOH/DCM) to afford **38d** (30 mg, yield 43%). ^1^H NMR (400 MHz, DMSO-*d*_6_) δ 12.37 (s, 1H), 7.63 (dd, *J* = 8.8, 2.0 Hz, 1H), 7.55–7.52 (m, 3H), 7.45 (d, *J* = 8.8 Hz, 1H), 7.40–7.39 (m, 1H), 7.29 (d, *J* = 6.8 Hz, 1H), 6.93 (d, *J* = 2.0 Hz, 1H), 6.75 (dd, *J* = 8.8, 16.0 Hz, 4H), 5.24 (dd, *J* = 12.0, 4.0 Hz, 1H), 3.72 (s, 3H), 3.70–3.65 (m,1H), 2.83 (dd, *J* = 4.4, 18.4 Hz, 2H), 1.84 (s, 3H). ^13^C NMR (101 MHz, DMSO-*d*_6_) δ 167.42, 160.60, 158.70, 152.63, 150.29, 137.75, 135.14, 134.79, 131.60, 129.80, 129.09, 128.92, 128.78, 128.75, 127.24, 126.52, 126.49, 125.13, 121.14, 118.07, 114.15, 58.67, 55.49, 45.74, 21.90. ESI-MS for C_27_H_22_ClN_3_O_3_: calculated 471.1, found *m*/*z* 472.2, 474.2, [M + H]^+^; 470.3, 472.3 [M – H]^−^. UPLC–MS purity (UV at 215 nm) 99%.

### 3-(1-Acetyl-5-(4-(*tert*-butyl)phenyl)-4,5-dihydro-1*H*-pyrazol-3-yl)-6-chloro-4-phenylquinolin-2(1*H*)-one (**39d**)

Compound **39d** was synthesized via general procedure C_2_ using **90c** (141 mg, 0.31 mmol) with acetic anhydride **116** (59 μL, 0.62 mmol) (165 °C, 200 W). Purification was performed by direct phase flash chromatography (SiO_2_ gold 24 g, 0–5% EtOH/DCM) to afford **39d** (105 mg, yield 68%). ^1^H NMR (400 MHz, DMSO-*d*_6_) δ 12.36 (s, 1H), 7.63 (dt, *J* = 9.8, 4.9 Hz, 1H), 7.60–7.48 (m, 3H), 7.44 (t, *J* = 9.5 Hz, 1H), 7.41–7.36 (m, 1H), 7.30 (d, *J* = 7.1 Hz, 1H), 7.24 (d, *J* = 8.3 Hz, 2H), 6.94 (d, *J* = 2.3 Hz, 1H), 6.75 (d, *J* = 8.3 Hz, 2H), 5.32–5.17 (m, 1H), 3.69 (dt, *J* = 28.5, 14.3 Hz, 1H), 2.86 (dd, *J* = 18.4, 4.6 Hz, 1H), 1.86 (s, 3H), 1.27 (s, 9H). ^13^C NMR (101 MHz, DMSO-*d*_6_) δ 167.01, 160.12, 152.20, 149.87, 149.25, 139.30, 137.30, 134.72, 131.14, 129.30, 128.66, 128.44, 128.31, 128.29, 126.07, 126.04, 125.16, 125.13, 124.65, 120.69, 117.60, 58.45, 45.30, 34.12, 31.10, 21.42. *t*_R_ = 1.83 min (apolar method). ESI-MS for C_30_H_28_ClN_3_O_2_: calculated 497.2, found *m*/*z* 498.5, 500.5 [M + H]^+^; 494.4, 496.4 [M – H]^−^. UPLC–MS purity (UV at 215 nm) >99.5%.

### 3-(1-Acetyl-5-(4-(trifluoromethyl)phenyl)-4,5-dihydro-1*H*-pyrazol-3-yl)-6-chloro-4-phenylquinolin-2(1*H*)-one (**40d**)

Compound **40d** was synthesized via general procedure C_2_ using **92c** (326 mg, 0.70 mmol) with acetic anhydride **116** (132 μL, 1.40 mmol) (165 °C, 200 W). Purification was performed by direct phase flash chromatography (SiO_2_ gold 24 g, 0–5% EtOH/DCM) to afford **40d** (268 mg, yield 74%). ^1^H NMR (400 MHz, DMSO-*d*_6_) δ 12.39 (s, 1H), 7.67–7.49 (m, 6H), 7.46 (d, *J* = 8.8 Hz, 1H), 7.44–7.39 (m, 1H), 7.32–7.25 (m, 1H), 7.03 (d, *J* = 8.0 Hz, 2H), 6.95 (d, *J* = 2.4 Hz, 1H), 5.42 (dd, *J* = 12.1, 4.7 Hz, 1H), 3.79 (dd, *J* = 18.5, 12.1 Hz, 1H), 2.87 (dd, *J* = 18.5, 4.8 Hz, 1H), 1.89 (s, 3H). ^13^C NMR (101 MHz, DMSO-*d*_6_) δ 167.21, 160.08, 152.27, 150.01, 146.70, 137.34, 134.69, 131.23, 129.39, 128.54, 128.51, 128.33, 127.90, 127.58, 126.24, 126.11, 126.05, 125.52, 125.43, 125.39, 125.35, 124.41, 122.82, 120.62, 117.63, 58.36, 45.14, 21.30. *t*_R_ = 2.55 min (generic method). ESI-MS for C_27_H_19_ClF_3_N_3_O_2_: calculated 509.1, found *m*/*z* 510.5, 513.5 [M + H]^+^; 508.4, 510.4 [M – H]^−^. UPLC–MS purity (UV at 215 nm) 99%.

### 3-(1-Acetyl-5-(furan-2-yl)-4,5-dihydro-1*H*-pyrazol-3-yl)-6-chloro-4-phenylquinolin-2(1*H*)-one (**41d**)

Compound **41d** was synthesized via general procedure C_2_ using **93c** (202 mg, 0.52 mmol) with acetic anhydride **116** (98 μL, 1.04 mmol) (165 °C, 200 W). Purification was performed by direct phase flash chromatography (SiO_2_ gold 24 g, 0–5% EtOH/DCM) to afford **41d** (162 mg, yield 73%). ^1^H NMR (400 MHz, DMSO-*d*_6_) δ 12.36 (s, 1H), 7.63 (dd, *J* = 8.8, 2.3 Hz, 1H), 7.52–7.42 (m, 5H), 7.35–7.29 (m, 2H), 6.96 (d, *J* = 2.3 Hz, 1H), 6.33 (dd, *J* = 3.3, 1.9 Hz, 1H), 6.03 (d, *J* = 3.2 Hz, 1H), 5.38 (dd, *J* = 11.9, 4.6 Hz, 1H), 3.57 (dd, *J* = 18.2, 11.9 Hz, 1H), 3.20 (dd, *J* = 18.2, 4.7 Hz, 1H), 1.75 (s, 3H). ^13^C NMR (101 MHz, DMSO-*d*_6_) δ 167.26, 160.12, 152.98, 152.28, 150.04, 142.02, 137.37, 134.92, 131.18, 128.87, 128.80, 128.25, 128.19, 126.12, 126.07, 124.31, 120.62, 117.59, 110.33, 106.33, 52.35, 41.84, 21.33. *t*_R_ = 0.95 min (apolar method). ESI-MS for C_24_H_18_ClN_3_O_3_: calculated 431.1, found *m*/*z* 432.4, 434.4 [M + H]^+^; 430.4, 432.5 [M – H]^−^. UPLC–MS purity (UV at 215 nm) >99.5%.

### 3-(1-Acetyl-5-(4′-chloro-[1,1′-biphenyl]-4-yl)-4,5-dihydro-1*H*-pyrazol-3-yl)-6-chloro-4-phenylquinolin-2(1*H*)-one (**42d**)

Compound **42d** was synthesized via general procedure C_2_ using **95c** (224 mg, 0.44 mmol) with acetic anhydride **116** (83 μL, 0.88 mmol) (165 °C, 200 W). Purification was performed by direct phase flash chromatography (SiO_2_ gold 24 g, 0–10% EtOH/DCM) to afford **42d** (245 mg, quantitative yield). ^1^H NMR (400 MHz, DMSO-*d*_6_) δ 12.37 (s, 1H), 7.71–7.48 (m, 10H), 7.48–7.39 (m, 2H), 7.29 (dt, *J* = 5.2, 2.0 Hz, 1H), 6.94 (d, *J* = 2.4 Hz, 1H), 6.90 (d, *J* = 8.3 Hz, 2H), 5.35 (dd, *J* = 12.1, 4.6 Hz, 1H), 3.78 (dd, *J* = 18.4, 12.1 Hz, 1H), 2.88 (dd, *J* = 18.5, 4.6 Hz, 1H), 1.88 (s, 3H). ^13^C NMR (101 MHz, DMSO-*d*_6_) δ 167.09, 160.12, 152.23, 149.93, 141.80, 138.63, 137.66, 137.30, 134.73, 132.26, 131.16, 129.39, 128.82, 128.59, 128.49, 128.40, 128.37, 128.29, 126.74, 126.15, 126.10, 126.05, 124.60, 120.67, 117.60, 58.48, 45.29, 21.41. *t*_R_ = 2.00 min (apolar method). ESI-MS for C_32_H_23_Cl_2_N_3_O_2_: calculated 551.1, found *m*/*z* 552.0, 554.0, 556.1 [M + H]^+^; 550.1, 552.1, 554.0 [M – H]^−^. UPLC–MS purity (UV at 215 nm) 99%.

### 3-(1-Acetyl-5-(4′-bromo-[1,1′-biphenyl]-4-yl)-4,5-dihydro-1*H*-pyrazol-3-yl)-6-chloro-4-phenylquinolin-2(1*H*)-one (**43d**)

Compound **43d** was synthesized via general procedure C_2_ using **96c** (203 mg, 0.36 mmol) with acetic anhydride **116** (68 μL, 0.72 mmol) (165 °C, 200 W). Purification was performed by direct phase flash chromatography (SiO_2_ gold 24 g, 0–10% EtOH/DCM) to afford **43d** (175 mg, yield 80%). ^1^H NMR (400 MHz, DMSO-*d*_6_) δ 12.37 (s, 1H), 7.66–7.36 (m, 12H), 7.32–7.24 (m, 1H), 6.98–6.85 (m, 3H), 5.35 (dd, *J* = 12.0, 4.6 Hz, 1H), 3.78 (dd, *J* = 18.4, 12.1 Hz, 1H), 2.90 (dd, *J* = 18.4, 4.6 Hz, 1H), 1.88 (s, 3H). ^13^C NMR (101 MHz, DMSO-*d*_6_) δ 167.10, 160.12, 152.20, 149.92, 141.81, 138.97, 137.70, 137.30, 134.73, 131.71, 131.12, 129.36, 128.67, 128.58, 128.46, 128.36, 128.27, 126.67, 126.17, 126.12, 126.04, 124.57, 120.84, 120.65, 117.58, 79.15, 58.51, 45.28, 21.39. *t*_R_ = 2.52 min (apolar method). ESI-MS for C_32_H_23_BrClN_3_O_2_: calculated 595.1, found *m*/*z* 595.9, 597.9, 600.0 [M + H]^+^; 594.0, 596.0, 597.9 [M – H]^−^. UPLC–MS purity (UV at 215 nm) 99%.

### 3-(1-Acetyl-5-(4′-methoxy-[1,1′-biphenyl]-4-yl)-4,5-dihydro-1*H*-pyrazol-3-yl)-6-chloro-4-phenylquinolin-2(1*H*)-one (**44d**)

Compound **44d** was synthesized via general procedure C_2_ using **97c** (179 mg, 0.35 mmol) with acetic anhydride **116** (66 μL, 0.70 mmol) (165 °C, 200 W). Purification was performed by direct phase flash chromatography (SiO_2_ gold 24 g, 0–8% EtOH/DCM) to afford **44d** (158 mg, yield 83%). ^1^H NMR (600 MHz, DMSO-*d*_6_) δ 12.39 (s, 1H), 7.63 (dd, *J* = 8.8, 2.4 Hz, 1H), 7.60–7.53 (m, 5H), 7.45 (d, *J* = 8.5 Hz, 3H), 7.43–7.39 (m, 1H), 7.31–7.27 (m, 1H), 7.03–6.99 (m, 2H), 6.94 (d, *J* = 2.4 Hz, 1H), 6.86 (d, *J* = 8.3 Hz, 2H), 5.33 (dd, *J* = 12.1, 4.6 Hz, 1H), 3.79 (s, 4H), 2.88 (dd, *J* = 18.5, 4.6 Hz, 1H), 1.88 (s, 3H). ^13^C NMR (151 MHz, DMSO-*d*_6_) δ 167.09, 160.17, 158.87, 152.28, 149.95, 140.70, 138.74, 137.33, 134.75, 132.24, 131.20, 129.43, 128.64, 128.53, 128.43, 128.33, 127.72, 126.28, 126.12, 126.08, 126.04, 124.66, 120.71, 117.64, 114.35, 58.53, 55.16, 45.34, 21.47. *t*_R_ = 1.65 min (apolar method). ESI-MS for C_33_H_26_ClN_3_O_3_: calculated 547.2, found *m*/*z* 548.0, 550.0 [M + H]^+^; 546.0, 548.0 [M – H]^−^. UPLC–MS purity (UV at 215 nm) >99.5%.

### 3-(1-Acetyl-5-(1-methyl-1*H*-indol-5-yl)-4,5-dihydro-1*H*-pyrazol-3-yl)-6-chloro-4-phenylquinolin-2(1*H*)-one (**45d**)

Compound **45d** was synthesized via general procedure C_2_ using **98c** (127 mg, 0.28 mmol) with acetic anhydride **116** (53 μL, 0.56 mmol) (165 °C, 200 W). Purification was performed by direct phase flash chromatography (SiO_2_ gold 24 g, 0–10% EtOH/DCM) to afford **45d** (107 mg, yield 75%). ^1^H NMR (400 MHz, DMSO-*d*_6_) δ 12.36 (s, 1H), 7.64–7.56 (m, 3H), 7.53 (ddd, *J* = 8.9, 5.6, 3.3 Hz, 1H), 7.47–7.40 (m, 2H), 7.31–7.23 (m, 3H), 6.99 (d, *J* = 1.7 Hz, 1H), 6.94 (d, *J* = 2.3 Hz, 1H), 6.65 (dd, *J* = 8.5, 1.7 Hz, 1H), 6.35 (dd, *J* = 3.1, 0.8 Hz, 1H), 5.36 (dd, *J* = 12.0, 4.5 Hz, 1H), 3.75 (s, 4H), 2.90 (dd, *J* = 18.4, 4.6 Hz, 1H), 1.86 (s, 3H). ^13^C NMR (101 MHz, DMSO-*d*_6_) δ 166.93, 160.20, 152.06, 149.80, 137.30, 135.61, 134.79, 133.10, 131.09, 129.92, 129.46, 128.59, 128.47, 128.37, 128.23, 127.74, 126.06, 124.81, 120.74, 119.02, 117.58, 117.16, 109.63, 100.26, 59.32, 45.86, 32.45, 21.54. *t*_R_ = 2.41 min (generic method). ESI-MS for C_29_H_23_ClN_4_O_2_: calculated 494.1, found *m*/*z* 495.2, 497.2 [M + H]^+^; 493.3, 495.3 [M – H]^−^. UPLC–MS purity (UV at 215 nm) >99.5%.

### 3-(1-Acetyl-5-(1-ethyl-1*H*-indol-5-yl)-4,5-dihydro-1*H*-pyrazol-3-yl)-6-chloro-4-phenylquinolin-2(1*H*)-one (**46d**)

Compound **46d** was synthesized via general procedure C_2_ using **99c** (168 mg, 0.36 mmol) with acetic anhydride **116** (68 μL, 0.72 mmol) (165 °C, 200 W). Purification was performed by direct phase flash chromatography (SiO_2_ gold 24 g, 0–10% EtOH/DCM) to afford **46d** (128 mg, yield 70%). ^1^H NMR (400 MHz, DMSO-*d*_6_) δ 12.36 (s, 1H), 7.64–7.50 (m, 4H), 7.47–7.40 (m, 2H), 7.35 (d, *J* = 3.1 Hz, 1H), 7.32–7.27 (m, 2H), 6.97 (dd, *J* = 22.0, 2.0 Hz, 2H), 6.64 (dd, *J* = 8.5, 1.7 Hz, 1H), 6.35 (dd, *J* = 3.1, 0.8 Hz, 1H), 5.35 (dd, *J* = 12.0, 4.6 Hz, 1H), 4.16 (q, *J* = 7.2 Hz, 2H), 3.78 (dd, *J* = 18.4, 12.0 Hz, 1H), 2.91 (dd, *J* = 18.4, 4.6 Hz, 1H), 1.86 (s, 3H), 1.33 (t, *J* = 7.2 Hz, 3H). ^13^C NMR (101 MHz, DMSO-*d*_6_) δ 166.95, 160.20, 152.07, 149.81, 137.29, 134.80, 134.57, 133.07, 131.09, 129.43, 128.61, 128.47, 128.36, 128.31, 128.24, 127.90, 126.07, 124.82, 120.74, 118.97, 117.58, 117.29, 109.62, 100.48, 59.33, 45.85, 40.28, 21.55, 21.18, 15.52. *t*_R_ = 2.51 min (generic method). ESI-MS for C_30_H_25_ClN_4_O_2_: calculated 508.2, found *m*/*z* 509.3, 511.2 [M + H]^+^; 507.3, 509.3 [M – H]^−^. UPLC–MS purity (UV at 215 nm) >99.5%.

### 3-(1-Acetyl-5-(1-ethyl-1*H*-indazol-5-yl)-4,5-dihydro-1*H*-pyrazol-3-yl)-6-chloro-4-phenylquinolin-2(1*H*)-one (**47d**)

Compound **47d** was synthesized via general procedure C_2_ using **100c** (131 mg, 0.28 mmol) with acetic anhydride **116** (53 μL, 0.56 mmol) (165 °C, 200 W). Purification was performed by direct phase flash chromatography (SiO_2_ gold 12 g, 0–5% EtOH/DCM) to afford **47d** (118 mg, yield 82%). ^1^H NMR (400 MHz, DMSO-*d*_6_) δ 12.37 (s, 1H), 7.98 (d, *J* = 0.9 Hz, 1H), 7.66–7.57 (m, 3H), 7.57–7.49 (m, 2H), 7.48–7.40 (m, 2H), 7.29 (ddt, *J* = 7.6, 2.0, 1.0 Hz, 1H), 7.15 (t, *J* = 1.1 Hz, 1H), 6.94 (d, *J* = 2.3 Hz, 1H), 6.84 (dd, *J* = 8.7, 1.6 Hz, 1H), 5.41 (dd, *J* = 12.0, 4.5 Hz, 1H), 4.41 (q, *J* = 7.2 Hz, 2H), 3.79 (dd, *J* = 18.5, 12.1 Hz, 1H), 2.88 (dd, *J* = 18.4, 4.6 Hz, 1H), 1.88 (s, 3H), 1.37 (t, *J* = 7.2 Hz, 3H). ^13^C NMR (101 MHz, DMSO-*d*_6_) δ 167.05, 160.18, 152.21, 149.88, 138.03, 137.33, 134.72, 134.44, 132.33, 131.16, 129.51, 128.57, 128.41, 128.29, 126.08, 124.71, 124.11, 123.28, 120.72, 117.63, 117.30, 109.78, 58.90, 45.57, 43.06, 21.50, 14.95. *t*_R_ = 2.29 min (generic method). ESI-MS for C_29_H_24_ClN_5_O_2_: calculated 509.2, found *m*/*z* 510.2, 512.2 [M + H]^+^, 508.3, 510.4 [M – H]^−^. UPLC–MS purity (UV at 215 nm) 99%.

### 3-(1-Acetyl-5-(2-ethyl-2*H*-indazol-5-yl)-4,5-dihydro-1*H*-pyrazol-3-yl)-6-chloro-4-phenylquinolin-2(1*H*)-one (**48d**)

Compound **48d** was synthesized via general procedure C_2_ using **101c** (80 mg, 0.17 mmol) with acetic anhydride **116** (32 μL, 0.34 mmol) (165 °C, 200 W). Purification was performed by direct phase flash chromatography (SiO_2_ gold 24 g, 0–10% EtOH/DCM) to afford **48d** (67 mg, yield 77%). ^1^H NMR (400 MHz, DMSO-*d*_6_) δ 12.37 (s, 1H), 8.31 (d, *J* = 0.9 Hz, 1H), 7.67–7.50 (m, 4H), 7.48–7.42 (m, 3H), 7.36–7.26 (m, 1H), 7.13–7.05 (m, 1H), 6.94 (d, *J* = 2.4 Hz, 1H), 6.65 (dd, *J* = 9.0, 1.7 Hz, 1H), 5.36 (dd, *J* = 12.0, 4.6 Hz, 1H), 4.44 (q, *J* = 7.3 Hz, 2H), 3.78 (dd, *J* = 18.4, 12.0 Hz, 1H), 2.87 (dd, *J* = 18.4, 4.6 Hz, 1H), 1.89 (s, 3H), 1.50 (t, *J* = 7.2 Hz, 3H). ^13^C NMR (101 MHz, DMSO-*d*_6_) δ 167.04, 160.18, 152.20, 149.83, 147.32, 137.35, 134.71, 134.46, 131.13, 129.52, 128.57, 128.54, 128.37, 128.27, 126.04, 124.73, 123.59, 123.01, 120.93, 120.72, 117.63, 117.34, 116.65, 59.13, 47.69, 45.35, 21.52, 15.84. *t*_R_ = 2.14 min (generic method). ESI-MS for C_29_H_24_ClN_5_O_2_: calculated 509.2, found *m*/*z* 510.2, 512.2 [M + H]^+^, 508.2, 510.3 [M – H]^−^. UPLC–MS purity (UV at 215 nm) 99%.

### 3-(1-Acetyl-5-(1-propyl-1*H*-indazol-5-yl)-4,5-dihydro-1*H*-pyrazol-3-yl)-6-chloro-4-phenylquinolin-2(1*H*)-one (**49d**)

Compound **49d** was synthesized via general procedure C_2_ using **102c** (188 mg, 0.39 mmol) with acetic anhydride **116** (74 μL, 0.78 mmol) (165 °C, 200 W). Purification was performed by direct phase flash chromatography (SiO_2_ gold 24 g, 0–10% EtOH/DCM) to afford **49d** (184 mg, yield 90%. ^1^H NMR (400 MHz, DMSO-*d*_6_) δ 12.37 (s, 1H), 7.99 (d, *J* = 0.9 Hz, 1H), 7.65–7.57 (m, 3H), 7.56–7.50 (m, 2H), 7.47–7.41 (m, 2H), 7.36–7.25 (m, 1H), 7.17–7.09 (m, 1H), 6.94 (d, *J* = 2.3 Hz, 1H), 6.85 (dd, *J* = 8.8, 1.7 Hz, 1H), 5.41 (dd, *J* = 12.0, 4.6 Hz, 1H), 4.33 (t, *J* = 6.9 Hz, 2H), 3.78 (dd, *J* = 18.5, 12.0 Hz, 1H), 2.89 (dd, *J* = 18.5, 4.6 Hz, 1H), 1.90–1.77 (m, 5H), 0.82 (t, *J* = 7.4 Hz, 3H). ^13^C NMR (101 MHz, DMSO-*d*_6_) δ 167.06, 160.14, 152.20, 149.85, 147.37, 137.29, 134.69, 134.43, 131.13, 129.52, 128.53, 128.35, 128.26, 126.07, 124.73, 123.73, 123.58, 120.81, 120.71, 117.59, 117.37, 116.65, 59.13, 54.23, 45.34, 23.40, 21.51, 10.86. *t*_R_ = 1.21 min (generic method). ESI-MS for C_30_H_26_ClN_5_O_2_: calculated 523.2, found *m*/*z* 524.1, 526.2 [M + H]^+^, 522.3, 524.2 [M – H]^−^. UPLC–MS purity (UV at 215 nm) 94%.

### 3-(1-Acetyl-5-(2-propyl-2*H*-indazol-5-yl)-4,5-dihydro-1*H*-pyrazol-3-yl)-6-chloro-4-phenylquinolin-2(1*H*)-one (**50d**)

Compound **50d** was synthesized via general procedure C_2_ using **103c** (132 mg, 0.27 mmol) with acetic anhydride **116** (51 μL, 0.54 mmol) (165 °C, 200 W). Purification was performed by direct phase flash chromatography (SiO_2_ gold 24 g, 0–10% EtOH/DCM) to afford **50d** (120 mg, yield 85%). ^1^H NMR (400 MHz, DMSO-*d*_6_) δ 12.37 (s, 1H), 8.29 (d, *J* = 1.0 Hz, 1H), 7.65–7.56 (m, 3H), 7.56–7.49 (m, 1H), 7.48–7.40 (m, 3H), 7.32–7.26 (m, 1H), 7.08 (t, *J* = 1.2 Hz, 1H), 6.93 (d, *J* = 2.3 Hz, 1H), 6.64 (dd, *J* = 8.9, 1.7 Hz, 1H), 5.35 (dd, *J* = 12.0, 4.5 Hz, 1H), 4.36 (t, *J* = 6.9 Hz, 2H), 3.77 (dd, *J* = 18.4, 12.0 Hz, 1H), 2.87 (dd, *J* = 18.4, 4.6 Hz, 1H), 1.97–1.83 (m, 5H), 0.83 (t, *J* = 7.4 Hz, 3H). ^13^C NMR (101 MHz, DMSO-*d*_6_) δ 167.07, 160.15, 152.21, 149.85, 147.38, 137.31, 134.69, 134.43, 131.13, 129.52, 128.57, 128.53, 128.36, 128.26, 126.07, 126.04, 124.74, 123.74, 123.58, 120.81, 120.72, 117.61, 117.38, 116.65, 59.13, 54.24, 45.35, 23.41, 21.52, 10.87. *t*_R_ = 1.01 min (apolar method). ESI-MS for C_30_H_26_ClN_5_O_2_: calculated 523.2, found *m*/*z* 524.2, 526.2 [M + H]^+^, 522.3, 524.3 [M – H]^−^. UPLC–MS purity (UV at 215 nm) 99%.

### 3-(1-Acetyl-5-(4-(1-propyl-1*H*-pyrazol-4-yl)phenyl)-4,5-dihydro-1*H*-pyrazol-3-yl)-6-chloro-4-phenylquinolin-2(1*H*)-one (**51d**)

Compound **51d** was synthesized via general procedure C_2_ using **108c** (146 mg, 0.31 mmol) with acetic anhydride **116** (59 μL, 0.62 mmol) (165 °C, 200 W). Purification was performed by direct phase flash chromatography (SiO_2_ gold 24 g, 0–8% EtOH/DCM) to afford **51d** (138 mg, yield 81%). ^1^H NMR (400 MHz, DMSO-*d*_6_) δ 12.36 (s, 1H), 8.13 (s, 1H), 7.83 (d, *J* = 0.7 Hz, 1H), 7.62 (dd, *J* = 8.8, 2.4 Hz, 1H), 7.60–7.52 (m, 3H), 7.45 (d, *J* = 8.8 Hz, 1H), 7.43–7.38 (m, 3H), 7.30 (dt, *J* = 6.2, 1.7 Hz, 1H), 6.94 (d, *J* = 2.3 Hz, 1H), 6.79 (d, *J* = 8.3 Hz, 2H), 5.28 (dd, *J* = 12.0, 4.5 Hz, 1H), 4.07 (t, *J* = 7.0 Hz, 2H), 3.75 (dd, *J* = 18.4, 12.0 Hz, 1H), 2.86 (dd, *J* = 18.4, 4.6 Hz, 1H), 1.91–1.75 (m, 5H), 0.85 (t, *J* = 7.4 Hz, 3H). ^13^C NMR (101 MHz, DMSO-*d*_6_) δ 167.01, 160.14, 152.19, 149.89, 139.79, 137.30, 135.89, 134.74, 131.59, 131.15, 129.41, 128.60, 128.49, 128.38, 128.26, 126.88, 126.08, 126.00, 124.96, 124.66, 121.22, 120.69, 117.59, 58.57, 52.99, 45.27, 23.17, 21.42, 10.92. *t*_R_ = 1.22 min (apolar method). ESI-MS for C_32_H_28_ClN_5_O_2_: calculated 549.2, found *m*/*z* 550.0, 552.0 [M + H]^+^, 548.1, 550.0 [M – H]^−^. UPLC–MS purity (UV at 215 nm) 99%.

### 4-(5-(4-Fluorophenyl)-3-(2-oxo-4-phenyl-1,2-dihydroquinolin-3-yl)-4,5-dihydro-1*H*-pyrazol-1-yl)-4-oxobutanoic Acid (**52d**)

Compound **52d** was synthesized via general procedure C_1_ using **109c** (150 mg, 0.39 mmol) with succinic anhydride **115** (78 mg, 0.78 mmol) (120 °C, 200 W). Purification was performed by direct phase flash chromatography (SiO_2_ gold 24 g, 20–50% EtOH/DCM) to afford **52d** (114 mg, yield 59%). ^1^H NMR (400 MHz, DMSO-*d*_6_) δ 12.22 (s, 1H), 12.01 (s, 1H), 7.63–7.34 (m, 6H), 7.25 (dt, *J* = 6.8, 1.9 Hz, 1H), 7.14 (ddd, *J* = 8.2, 7.0, 1.2 Hz, 1H), 7.07–6.98 (m, 3H), 6.86–6.78 (m, 2H), 5.31 (dd, *J* = 12.0, 4.5 Hz, 1H), 3.72 (dd, *J* = 18.4, 12.0 Hz, 1H), 2.78 (dd, *J* = 18.4, 4.6 Hz, 1H), 2.47 (m, 2H), 2.32–2.25 (m, 2H). ^13^C NMR (101 MHz, DMSO-*d*_6_) δ 173.48, 168.56, 160.25, 152.77, 151.05, 141.11, 138.53, 138.42, 135.16, 131.27, 129.38, 128.53, 128.22, 128.20, 128.13, 127.54, 127.45, 127.38, 123.30, 122.25, 119.35, 115.51, 115.16, 114.95, 58.16, 45.27, 28.58, 28.23. *t*_R_ = 1.90 min (generic method). ESI-MS for C_28_H_22_FN_3_O_4_: calculated 483.2, found *m*/*z* 484.5 [M + H]^+^, 482.5 [M – H]^−^. UPLC–MS purity (UV at 215 nm) >99.5%.

### 4-(5-(4-Bromophenyl)-3-(2-oxo-4-phenyl-1,2-dihydroquinolin-3-yl)-4,5-dihydropyrazol-1-yl)-4-oxobutanoic Acid (**53d**)

Compound **53d** was synthesized via general procedure C_1_ using **110c** (41 mg, 0.09 mmol) with succinic anhydride **115** (18 mg, 0.18 mmol) (120 °C, 200 W). Title compound **53d** was obtained after the acidic workup (46 mg, yield 91%). ^1^H NMR (400 MHz, DMSO-*d*_6_) δ 12.22 (s, 1H), 12.01 (s, 1H), 7.61–7.45 (m, 4H), 7.45–7.36 (m, 4H), 7.25 (dt, *J* = 6.8, 1.9 Hz, 1H), 7.14 (ddd, *J* = 8.3, 7.0, 1.2 Hz, 1H), 7.04 (dd, *J* = 8.2, 1.4 Hz, 1H), 6.74 (d, *J* = 8.4 Hz, 2H), 5.29 (dd, *J* = 12.0, 4.6 Hz, 1H), 3.74 (dd, *J* = 18.4, 12.1 Hz, 1H), 2.77 (dd, *J* = 18.5, 4.6 Hz, 1H), 2.50 (m, 2H), 2.28 (t, *J* = 7.1 Hz, 2H). ^13^C NMR (101 MHz, DMSO-*d*_6_) δ 173.46, 168.60, 160.25, 152.78, 151.09, 141.60, 138.54, 135.17, 131.29, 131.23, 129.41, 128.51, 128.21, 128.14, 127.75, 127.39, 123.23, 122.27, 121.42, 120.03, 119.35, 115.53, 58.30, 45.12, 28.55, 28.22. *t*_R_ = 2.03 min (generic method). ESI-MS for C_28_H_22_BrN_3_O_4_: calculated 543.1, found *m*/*z* 544.5, 546.5 [M + H]^+^; 542.5, 544.4. UPLC–MS purity (UV at 215 nm) >99.5%.

### 4-(5-(3-Bromophenyl)-3-(2-oxo-4-phenyl-1,2-dihydroquinolin-3-yl)-4,5-dihydro-1*H*-pyrazol-1-yl)-4-oxobutanoic Acid (**54d**)

Compound **54d** was synthesized via general procedure C_1_ using **111c** (121 mg, 0.27 mmol) with succinic anhydride **115** (54 mg, 0.54 mmol) (120 °C, 200 W). Purification was performed by direct phase flash chromatography (SiO_2_ gold 24 g, 20–90% EtOH/DCM) to afford **54d** (11 mg, yield 7%). ^1^H NMR (400 MHz, DMSO-*d*_6_) δ 12.24 (s, 1H), 12.13 (s, 1H), 7.61–7.34 (m, 7H), 7.26 (dt, *J* = 7.8, 1.8 Hz, 1H), 7.23–7.11 (m, 3H), 7.06 (dd, *J* = 8.2, 1.4 Hz, 1H), 6.76 (dt, *J* = 7.8, 1.3 Hz, 1H), 5.32 (dd, *J* = 12.1, 4.8 Hz, 1H), 3.74 (dd, *J* = 18.5, 12.1 Hz, 1H), 2.84 (dd, *J* = 18.5, 4.8 Hz, 1H), 2.51 (m, 2H), 2.33–2.26 (m, 2H). ^13^C NMR (101 MHz, DMSO-*d*_6_) δ 174.08, 173.94, 169.19, 160.76, 153.36, 151.60, 145.30, 139.04, 135.72, 131.79, 131.18, 130.45, 129.75, 128.92, 128.71, 128.56, 127.90, 124.94, 123.65, 122.75, 122.09, 119.80, 116.01, 58.80, 45.64, 29.39, 29.01, 28.67. *t*_R_ = 2.00 min (generic method). ESI-MS for C_28_H_22_BrN_3_O_4_: calculated 543.1, found *m*/*z* 544.4, 546.4 [M + H]^+^; 542.4, 544.4 [M – H]^−^. UPLC–MS purity (UV at 215 nm) >99.5%.

### 4-(5-(4-Chlorophenyl)-3-(2-oxo-4-phenyl-1,2-dihydroquinolin-3-yl)-4,5-dihydro-1*H*-pyrazol-1-yl)-4-oxobutanoic Acid (**55d**)

Compound **55d** was synthesized via general procedure C_1_ using **112c** (121 mg, 0.27 mmol) with succinic anhydride **115** (54 mg, 054 mmol) (120 °C, 200 W). Purification was performed by direct phase flash chromatography (SiO_2_ gold 24 g, 4–20% EtOH/DCM) to afford **55d** (130 mg, yield 67%). ^1^H NMR (400 MHz, DMSO-*d*_6_) δ 12.23 (s, 1H), 12.05 (s, 1H), 7.61–7.46 (m, 4H), 7.43 (dd, *J* = 8.3, 1.1 Hz, 1H), 7.39 (dt, *J* = 6.0, 2.0 Hz, 1H), 7.30–7.23 (m, 3H), 7.15 (ddd, *J* = 8.3, 7.0, 1.2 Hz, 1H), 7.05 (dd, *J* = 8.2, 1.4 Hz, 1H), 6.81 (d, *J* = 8.5 Hz, 2H), 5.31 (dd, *J* = 12.0, 4.6 Hz, 1H), 3.75 (dd, *J* = 18.5, 12.1 Hz, 1H), 2.78 (dd, *J* = 18.5, 4.6 Hz, 1H), 2.58–2.44 (m, 2H), 2.30 (t, *J* = 7.1 Hz, 2H). ^13^C NMR (101 MHz, DMSO-*d*_6_) δ 173.95, 169.08, 160.73, 153.27, 151.57, 141.65, 139.02, 135.65, 132.01, 131.77, 129.89, 129.00, 128.79, 128.69, 128.62, 127.88, 123.73, 122.74, 119.83, 116.01, 58.72, 45.66, 29.04, 28.70. *t*_R_ = 1.97 min (generic method). ESI-MS for C_28_H_22_ClN_3_O_4_: calculated 499.1, found *m*/*z* 500.4, 502.4 [M + H]^+^; 498.4, 500.3 [M – H]^−^. UPLC–MS purity (UV at 215 nm) >99.5%.

### 4-(5-(4-Methoxyphenyl)-3-(2-oxo-4-phenyl-1,2-dihydroquinolin-3-yl)-4,5-dihydro-1*H*-pyrazol-1-yl)-4-oxobutanoic Acid (**56d**)

Compound **56d** was synthesized via general procedure C_1_ using **113c** (120 mg, 0.30 mmol) with succinic anhydride **115** (60 mg, 0.60 mmol) (120 °C, 200 W). Purification was performed by direct phase flash chromatography (SiO_2_ gold 24 g, 5–35% EtOH/DCM) to afford **56d** (99 mg, yield 67%). ^1^H NMR (400 MHz, DMSO-*d*_6_) δ 12.21 (s, 1H), 12.00 (s, 1H), 7.61–7.34 (m, 6H), 7.29–7.23 (m, 1H), 7.14 (ddd, *J* = 8.2, 7.0, 1.2 Hz, 1H), 7.04 (dd, *J* = 8.2, 1.4 Hz, 1H), 6.79–6.66 (m, 4H), 5.23 (dd, *J* = 11.9, 4.5 Hz, 1H), 3.72 (s, 4H), 2.79 (dd, *J* = 18.4, 4.5 Hz, 1H), 2.46 (dd, *J* = 6.8, 2.4 Hz, 2H), 2.27 (t, *J* = 6.9 Hz, 2H). ^13^C NMR (101 MHz, DMSO-*d*_6_) δ 173.99, 168.91, 160.78, 158.68, 153.22, 151.47, 139.00, 135.70, 134.85, 131.71, 129.84, 129.08, 128.66, 128.60, 127.87, 127.27, 123.91, 122.71, 119.88, 115.99, 114.16, 58.85, 55.51, 45.80, 29.11, 28.74. *t*_R_ = 1.83 min (generic method). ESI-MS for C_29_H_25_N_3_O_5_: calculated 495.1, found *m*/*z* 496.5 [M + H]^+^; 494.5 [M – H]^−^. UPLC–MS purity (UV at 215 nm) >99.5%.

### 4-Oxo-4-(3-(2-oxo-4-phenyl-1,2-dihydroquinolin-3-yl)-5-(4-(trifluoromethyl)phenyl)-4,5-dihydro-1*H*-pyrazol-1-yl)butanoic Acid (**57d**)

Compound **57d** was synthesized via general procedure C_1_ using **114c** (189 mg, 0.45 mmol) with succinic anhydride **115** (90 mg, 0.90 mmol) (120 °C, 200 W). Purification was performed by direct phase flash chromatography (SiO_2_ gold 24 g, 2–50% EtOH/DCM) to afford **57d** (173 mg, yield 71%). ^1^H NMR (400 MHz, DMSO-*d*_6_) δ 12.23 (s, 1H), 12.02 (s, 1H), 7.64–7.36 (m, 8H), 7.25 (d, *J* = 7.3 Hz, 1H), 7.15 (ddd, *J* = 8.3, 7.1, 1.2 Hz, 1H), 7.08–6.98 (m, 3H), 5.42 (dd, *J* = 12.1, 4.7 Hz, 1H), 3.79 (dd, *J* = 18.5, 12.1 Hz, 1H), 2.81 (dd, *J* = 18.5, 4.7 Hz, 1H), 2.59–2.48 (m, 2H), 2.36–2.26 (m, 2H). ^13^C NMR (101 MHz, DMSO-*d*_6_) δ 173.94, 169.21, 160.71, 153.30, 151.65, 147.20, 139.03, 135.67, 131.80, 129.88, 128.96, 128.70, 128.65, 128.29, 127.88, 126.70, 126.02, 125.84, 125.80, 123.63, 123.31, 122.76, 119.81, 116.02, 58.95, 45.63, 28.99, 28.68. *t*_R_ = 2.06 min (generic method). ESI-MS for C_29_H_22_F_3_N_3_O_4_: calculated 533.2, found *m*/*z* 534.5 [M + H]^+^; 532.5 [M – H]^−^. UPLC–MS purity (UV at 215 nm) >99.5%.

### 3-Acetyl-6-chloro-4-phenylquinolin-2(1*H*)-one (**60a**) (Scheme 1)

2-Amino-5-chlorophenyl)phenylmethanone **58** (1.460 g, 6.3 mmol) and ethyl acetoacetate (2.4 mL, 18.9 mmol) were dissolved in DMF (1.00 M) in an appropriately sized screw-capped pressure tube. The mixture was heated at 153 °C and stirred for 19 h. The solvent was removed under reduced pressure to afford **60a (**1.875 g, quantitative yield). ^1^H NMR (400 MHz, DMSO-*d*_6_) δ 12.38 (s, 1H), 7.64 (dd, *J* = 8.8, 2.4 Hz, 1H), 7.59–7.48 (m, 3H), 7.44 (d, *J* = 8.8 Hz, 1H), 7.39–7.28 (m, 2H), 6.95 (d, *J* = 2.3 Hz, 1H), 2.22 (s, 3H). *t*_R_ = 2.06 min (generic method). ESI-MS for C_17_H_12_ClNO_2_: calculated 297.1, found *m*/*z* 298.3, 300.3 [M + H]^+^; 296.3, 298.3 [M – H]^−^.

### 3-Acetyl-4-phenylquinolin-2(1*H*)-one (**61a**) (Scheme 1)

(2-Aminophenyl)phenylmethanone **59** (2.00 g, 10.1 mmol) and ethyl acetoacetate (1.9 mL, 15.2 mmol) were dissolved in DMF (1.00 M) in an appropriately sized microwaveable vessel and microwaved at 120 °C (200 W) for 1.5 h. The solvent was concentrated under reduced pressure, and the residue was diluted with DCM and washed (3 × 100 mL) with H_2_O. The organic layer was dried over Na_2_SO_4_, filtered and the solvent removed under reduced pressure. **61a** was obtained after precipitation from EtOAc (1.44 g, 53% yield). ^1^H NMR (400 MHz, DMSO-*d*_6_) δ 12.22 (s, 1H), 7.56 (ddd, *J* = 8.4, 7.1, 1.5 Hz, 1H), 7.54–7.47 (m, 3H), 7.41 (dd, *J* = 8.4, 1.2 Hz, 1H), 7.34–7.28 (m, 2H), 7.14 (ddd, *J* = 8.3, 7.1, 1.2 Hz, 1H), 7.05 (dd, *J* = 8.2, 1.4 Hz, 1H), 2.21 (s, 3H). ^13^C NMR (101 MHz, DMSO-*d*_6_) δ 201.60, 159.26, 146.96, 138.45, 134.19, 133.29, 131.20, 128.71, 128.64, 128.46, 127.03, 122.33, 118.89, 115.64, 31.47. *t*_R_ = 1.86 min (generic method). ESI-MS for C_17_H_13_NO_2_: calculated 263.1, found *m*/*z* 264.5 [M + H]^+^; 262.4 [M – H]^−^.

### (*E*)-6-Chloro-3-(3-(4-fluorophenyl)acryloyl)-4-phenylquinolin-2(1*H*)-one (**86b**)

3-Acetyl-6-chloro-4-phenylquinolin-2(1*H*)-one **60a** (2.517 g, 8.45 mmol) and 4-fluorobenzaldehyde **62** (906 μL, 8.45 mmol) were allowed to react overnight according to general procedure A. Purification was performed by direct phase flash chromatography (SiO_2_ gold 40 g; 0–20% EtOH/DCM) to afford **86b** (2.548 g, 75% yield). ^1^H NMR (400 MHz, DMSO-*d*_6_) δ 12.35 (s, 1H), 7.81–7.70 (m, 2H), 7.66 (dd, *J* = 8.8, 2.4 Hz, 1H), 7.54–7.41 (m, 5H), 7.36–7.30 (m, 2H), 7.28–7.19 (m, 2H), 6.98 (d, *J* = 2.4 Hz, 1H), 6.73 (d, *J* = 16.4 Hz, 1H). *t*_R_ = 2.42 min (generic method). ESI-MS for C_24_H_15_ClFNO_2_: calculated 403.1, found *m*/*z* 404.1, 406.1 [M + H]^+^; 402.1, 404.1 [M – H]^−^.

### 6-Chloro-3-(5-(4-fluorophenyl)-4,5-dihydro-1*H*-pyrazol-3-yl)-4-phenylquinolin-2(1*H*)-one (**86c**)

(*E*)-6-Chloro-3-(3-(4-fluorophenyl)acryloyl)-4-phenylquinolin-2(1*H*)-one **86b** (317 mg, 0.78 mmol) and hydrazine monohydrate (76 μL, 1.56 mmol) were allowed to react according to general procedure B. Crude compound was purified by flash column chromatography (SiO_2_ gold 40 g; 0–20% EtOH/DCM) to afford **86c** (303 mg, yield 92%). ^1^H NMR (400 MHz, DMSO-*d*_6_) δ 12.19 (s, 1H), 7.57 (dd, *J* = 8.8, 2.4 Hz, 1H), 7.50 (m, 2H), 7.41 (d, *J* = 8.8 Hz, 1H), 7.33 (dt, *J* = 6.9, 1.5 Hz, 1H), 7.23 (dt, *J* = 6.8, 2.0 Hz, 1H), 7.14 (d, *J* = 3.2 Hz, 1H), 7.13–7.02 (m, 4H), 6.89 (d, *J* = 2.3 Hz, 1H), 4.59 (td, *J* = 10.9, 10.2, 3.1 Hz, 1H), 3.23 (dd, *J* = 16.5, 11.0 Hz, 1H), 2.59–2.52 (m, 1H). *t*_R_ = 2.31 min (generic method). ESI-MS for C_24_H_17_ClFN_3_O: calculated 417.1, found *m*/*z* 418.4, 420.4 [M + H]^+^; 416.4, 418.3 [M – H]^−^.

### (*E*)-6-Chloro-3-(3-(4-chlorophenyl)acryloyl)-4-phenylquinolin-2(1*H*)-one (**87b**)

Compound **87b** was synthesized via general procedure A using **60a** (304 mg, 1.00 mmol) and 4-chlorobenzaldehyde **63** (140 mg, 1.00 mmol). Title compound **87b** was obtained by precipitation and filtration from the reaction crude (429 mg, quantitative yield). ^1^H NMR (400 MHz, DMSO-*d*_6_) δ 7.70 (d, *J* = 8.6 Hz, 2H), 7.63 (dd, *J* = 8.8, 2.4 Hz, 1H), 7.55–7.37 (m, 7H), 7.32 (dd, *J* = 7.6, 1.9 Hz, 2H), 6.96 (d, *J* = 2.4 Hz, 1H), 6.78 (d, *J* = 16.4 Hz, 1H). *t*_R_ = 2.55 min (generic method). ESI-MS for C_24_H_15_Cl_2_NO_2_: calculated 419.0, found *m*/*z* 420.4, 422.4, 424.4 [M + H]^+^; 418.5, 420.4, 422.3 [M – H]^−^.

### 6-Chloro-3-(5-(4-chlorophenyl)-4,5-dihydro-1*H*-pyrazol-3-yl)-4-phenylquinolin-2(1*H*)-one (**87c**)

Compound **87c** was synthesized via general procedure B using **87b** (429 mg, 1.00 mmol) with hydrazine hydrate (97 μL, 2.00 mmol). Purification was performed by direct phase flash chromatography (SiO_2_ gold 24 g; 0–10% EtOH/DCM) to afford **87c** (301 mg, yield 68%). ^1^H NMR (400 MHz, DMSO-*d*_6_) δ 12.20 (s, 1H), 7.58 (dd, *J* = 8.8, 2.4 Hz, 1H), 7.50 (tt, *J* = 7.5, 2.9 Hz, 3H), 7.42 (d, *J* = 8.8 Hz, 1H), 7.34 (dt, *J* = 7.0, 1.7 Hz, 1H), 7.30 (d, *J* = 8.4 Hz, 2H), 7.23 (dt, *J* = 7.3, 2.0 Hz, 1H), 7.18 (d, *J* = 3.2 Hz, 1H), 7.09 (d, *J* = 8.4 Hz, 2H), 6.90 (d, *J* = 2.3 Hz, 1H), 4.59 (td, *J* = 11.6, 9.3, 3.1 Hz, 1H), 3.25 (dd, *J* = 16.5, 11.1 Hz, 1H), 2.58–2.53 (m, 1H). *t*_R_ = 2.45 min (generic method). ESI-MS for C_24_H_17_Cl_2_N_3_O: calculated 433.1, found *m*/*z* 434.4, 436.4, 438.5 [M + H]^+^; 432.4, 434.4, 436.4 [M – H]^−^.

### (*E*)-3-(3-(4-Bromophenyl)acryloyl)-6-chloro-4-phenylquinolin-2(1*H*)-one (**88b**)

Compound **88b** was synthesized via general procedure A using **60a** (328 mg, 1.10 mmol) and 4-bromobenzaldehyde **64** (204 mg, 1.10 mmol). Title compound **88b** was obtained by precipitation and filtration from the reaction crude (511 mg, quantitative yield). ^1^H NMR (400 MHz, DMSO-*d*_6_) δ 7.67–7.47 (m, 6H), 7.46–7.35 (m, 4H), 7.30 (dd, *J* = 7.7, 1.8 Hz, 2H), 6.93 (d, *J* = 2.3 Hz, 1H), 6.80 (d, *J* = 16.4 Hz, 1H). *t*_R_ = 2.59 min (generic method). ESI-MS for C_24_H_15_BrClNO_2_: calculated 463.0, found *m*/*z* 464.3, 466.2, 468.2 [M + H]^+^; 462.2, 464.2, 466.2 [M – H]^−^.

### 3-(5-(4-Bromophenyl)-4,5-dihydro-1*H*-pyrazol-3-yl)-6-chloro-4-phenylquinolin-2(1*H*)-one (**88c**)

Compound **88c** was synthesized via general procedure B using **88b** (510 mg, 1.10 mmol) with hydrazine hydrate (110 μL, 2.20 mmol). Purification was performed by direct phase flash chromatography (0–8% EtOH/DCM) to afford **88c** (349 mg, yield 64%). ^1^H NMR (400 MHz, DMSO-*d*_6_) δ 12.20 (s, 1H), 7.58 (dd, *J* = 8.8, 2.4 Hz, 1H), 7.56–7.45 (m, 3H), 7.42 (dd, *J* = 8.6, 5.5 Hz, 3H), 7.34 (dt, *J* = 7.2, 1.6 Hz, 1H), 7.23 (dt, *J* = 7.2, 2.0 Hz, 1H), 7.18 (d, *J* = 3.2 Hz, 1H), 7.07–6.99 (m, 2H), 6.89 (d, *J* = 2.3 Hz, 1H), 4.58 (td, *J* = 11.6, 9.3, 3.2 Hz, 1H), 3.25 (dd, *J* = 16.5, 11.1 Hz, 1H), 2.57–2.53 (m, 1H). *t*_R_ = 2.50 min (generic method). ESI-MS for C_24_H_17_BrClN_3_O: calculated 477.0, found *m*/*z* 478.2, 480.2, 482.2 [M + H]^+^; 476.3, 478.2, 480.3 [M – H]^−^.

### (*E*)-6-Chloro-3-(3-(4-methoxyphenyl)acryloyl)-4-phenylquinolin-2(1*H*)-one (**89b**)

3-Acetyl-6-chloro-4-phenylquinolin-2(1*H*)-one **60a** (611 mg, 2.00 mmol) and *p*-anisaldehyde **65** (243 μL, 2.00 mmol) were allowed to react overnight according to general procedure A. Purification was performed by direct phase flash chromatography (SiO_2_ gold 40 g; 0–5% EtOH/DCM), affording the desired **89b** (867 mg, quantitative yield). ^1^H NMR (400 MHz, DMSO-*d*_6_) δ 12.31 (s, 1H), 7.68–7.58 (m, 3H), 7.49–7.38 (m, 5H), 7.34–7.29 (m, 2H), 6.99–6.89 (m, 3H), 6.60 (d, *J* = 16.3 Hz, 1H), 3.78 (s, 3H). *t*_R_ = 1.19 min (apolar method). ESI-MS for C_25_H_18_ClNO_3_: calculated 415.1, found *m*/*z* 416.4, 418.4 [M + H]^+^; 414.4, 416.4 [M – H]^−^.

### 6-Chloro-3-(5-(4-methoxyphenyl)-4,5-dihydro-1*H*-pyrazol-3-yl)-4-phenylquinolin-2(1*H*)-one (**89c**)

(*E*)-6-Chloro-3-(3-(4-methoxyphenyl)acryloyl)-4-phenylquinolin-2(1*H*)-one **89b** (780 mg, 1.9 mmol) and hydrazine monohydrate (185 μL, 3.8 mmol) were allowed to react according to general procedure B. Crude compound was purified by flash column chromatography (SiO_2_ gold 40 g; 0–10% EtOH/DCM) to afford **89c** (611 mg, yield 75%). ^1^H NMR (400 MHz, DMSO-*d*_6_) δ 12.19 (s, 1H), 7.57 (dd, *J* = 8.8, 2.3 Hz, 1H), 7.53–7.47 (m, 3H), 7.42 (d, *J* = 8.8 Hz, 1H), 7.34 (dd, *J* = 10.3, 3.8 Hz, 1H), 7.27–7.19 (m, 1H), 7.02 (dd, *J* = 8.9, 5.9 Hz, 3H), 6.90 (d, *J* = 2.3 Hz, 1H), 6.80 (d, *J* = 8.7 Hz, 2H), 4.52 (td, *J* = 10.4, 2.9 Hz, 1H), 3.73 (s, 3H), 3.18 (dd, *J* = 16.4, 10.9 Hz, 1H), 2.58–2.53 (m, 1H). ^13^C NMR (101 MHz, DMSO-*d*_6_) δ 166.84, 160.17, 152.10, 149.77, 149.57, 137.28, 134.76, 131.10, 129.96, 129.28, 128.71, 128.42, 128.30, 128.28, 126.41, 126.04, 124.80, 120.72, 117.58, 112.23, 58.37, 45.22, 40.19, 21.49. *t*_R_ = 2.24 min (generic method). ESI-MS for C_25_H_20_ClN_3_O_2_: calculated 429.1, found *m*/*z* 430.4, 432.4 [M + H]^+^; 428.4, 430.5 [M – H]^−^.

### (*E*)-3-(3-(4-(*tert*-Butyl)phenyl)acryloyl)-6-chloro-4-phenylquinolin-2(1*H*)-one (**90b**)

Compound **90b** was synthesized via general procedure A using **60a** (543 mg, 1.80 mmol) and 4-*tert*-butylbenzaldeyde **66** (301 μL, 1.80 mmol). Purification was performed by direct phase flash chromatography (SiO_2_ gold 40 g; 0–10% EtOH/DCM) to afford **90b** (441 mg, yield 55%). ^1^H NMR (400 MHz, DMSO-*d*_6_) δ 12.33 (s, 1H), 7.64 (dd, *J* = 8.8, 2.4 Hz, 1H), 7.58 (d, *J* = 8.5 Hz, 2H), 7.50–7.37 (m, 7H), 7.32 (dd, *J* = 7.7, 1.9 Hz, 2H), 6.97 (d, *J* = 2.4 Hz, 1H), 6.69 (d, *J* = 16.4 Hz, 1H), 1.26 (s, 9H). ^13^C NMR (101 MHz, DMSO-*d*_6_) δ 193.82, 159.40, 153.81, 146.61, 146.19, 137.50, 133.60, 132.56, 131.52, 130.89, 128.86, 128.80, 128.50, 128.43, 126.64, 126.05, 125.71, 125.56, 120.56, 117.68, 79.15, 34.62, 30.97, 30.82. Rt 2.18 min (generic method). ESI-MS for C28H24ClNO2: calculated 441.1, found *m*/*z* 442.4, 444.4 [M + H]+; 440.4, 442.4 [M – H]-.

### 3-[5-(4-*tert*-Butylphenyl)-4,5-dihydro-1*H*-pyrazol-3-yl]-6-chloro-4-phenylquinolin-2(1*H*)-one (**90c**)

Compound **90c** was synthesized via general procedure B using **90b** (369 mg, 0.83 mmol) with hydrazine hydrate (81 μL, 1.66 mmol). Purification was performed by direct phase flash chromatography (SiO_2_ gold 24 g; 0–90% EtOAc/CHX) to afford **90c** (351 mg, yield 93%). ^1^H NMR (400 MHz, DMSO-*d*_6_) δ 12.20 (s, 1H), 7.56 (dd, *J* = 8.8, 2.4 Hz, 1H), 7.49 (dtd, *J* = 9.6, 7.2, 5.3 Hz, 3H), 7.42 (d, *J* = 8.8 Hz, 1H), 7.33 (dt, *J* = 7.2, 1.7 Hz, 1H), 7.25 (d, *J* = 8.3 Hz, 3H), 7.06 (d, *J* = 3.0 Hz, 1H), 7.02 (d, *J* = 8.4 Hz, 2H), 6.90 (d, *J* = 2.3 Hz, 1H), 4.54 (t, *J* = 10.4 Hz, 1H), 3.19 (dd, *J* = 16.4, 10.9 Hz, 1H), 2.58 (dd, *J* = 16.4, 9.6 Hz, 1H), 1.26 (s, 9H). ^13^C NMR (101 MHz, DMSO-*d*_6_) δ 160.54, 149.21, 148.30, 145.49, 140.31, 136.97, 135.21, 130.32, 129.19, 128.72, 128.26, 128.21, 128.14, 127.79, 127.18, 126.88, 126.33, 126.18, 125.75, 125.62, 124.92, 120.92, 117.33, 79.16, 62.69, 44.34, 34.09, 31.13, 31.05, 30.74. *t*_R_ = 1.82 min (generic method). ESI-MS for C_28_H_26_ClN_3_O: calculated 455.2, found *m*/*z* 456.5, 458.4, [M + H]^+^; 454.5 456.5 [M – H]^−^.

### (*E*)-6-Chloro-3-(3-(3-fluoro-4-methoxyphenyl)acryloyl)-4-phenylquinolin-2(1*H*)-one (**91b**)

Compound **91b** was synthesized via general procedure A using **60a** (473 mg, 1.60 mmol) and 3-fluoro-4-methoxybenzaldehyde **67** (247 mg, 1.60 mmol). Title compound **91b** was obtained after precipitation and filtration from the reaction crude (547 mg, yield 79%). ^1^H NMR (600 MHz, DMSO-*d*_6_) δ 12.38 (s, 1H), 7.70–7.62 (m, 2H), 7.52–7.40 (m, 6H), 7.35–7.30 (m, 2H), 7.18 (t, *J* = 8.7 Hz, 1H), 6.97 (d, *J* = 2.4 Hz, 1H), 6.69 (d, *J* = 16.3 Hz, 1H), 3.88 (s, 3H). ^13^C NMR (151 MHz, DMSO-*d*_6_) δ 194.19, 159.89, 153.13, 150.69, 149.82, 149.71, 147.06, 145.83, 138.00, 134.10, 133.01, 131.35, 129.34, 129.27, 128.90, 127.92, 127.85, 127.10, 126.87, 126.52, 126.02, 121.07, 118.17, 115.70, 115.52, 114.26, 56.62. *t*_R_ = 2.35 min (generic method). ESI-MS for C_25_H_17_ClFNO_3_: calculated 433.1, found *m*/*z* 434.4, 436.4 [M + H]^+^; 432.3, 434.3 [M – H]^−^.

### 6-Chloro-3-(5-(3-fluoro-4-methoxyphenyl)-4,5-dihydro-1*H*-pyrazol-3-yl)-4-phenylquinolin-2(1*H*)-one (**91c**)

Compound **91c** was synthesized via general procedure B using **91b** (203 mg, 0.50 mmol) with hydrazine hydrate (49 μL, 1.00 mmol). Purification was performed by direct phase flash chromatography (SiO_2_ gold 24 g; 0–5% EtOH/DCM) to afford **91c** (213 mg, quantitative yield). ^1^H NMR (400 MHz, DMSO-*d*_6_) δ 12.20 (s, 1H), 7.63–7.39 (m, 5H), 7.35–7.29 (m, 1H), 7.23 (dt, *J* = 7.0, 2.0 Hz, 1H), 7.12 (d, *J* = 3.1 Hz, 1H), 7.03 (t, *J* = 8.6 Hz, 1H), 6.97–6.85 (m, 3H), 4.55 (ddd, *J* = 11.5, 9.0, 2.9 Hz, 1H), 3.81 (s, 3H), 3.19 (dd, *J* = 16.5, 11.0 Hz, 1H), 2.60–2.53 (m, 1H). ^13^C NMR (101 MHz, DMSO-*d*_6_) δ 161.01, 152.92, 150.50, 148.84, 146.52, 145.91, 137.45, 137.04, 135.64, 130.85, 129.61, 129.14, 128.70, 127.20, 126.24, 126.10, 123.00, 121.36, 117.82, 114.42, 114.23, 114.02, 62.31, 56.48, 44.83. *t*_R_ = 2.27 min (generic method). ESI-MS for C_25_H_19_ClFN_3_O_2_: calculated 447.1, found *m*/*z* 448.4, 450.3 [M + H]^+^; 446.3, 448.2 [M – H]^−^.

### (*E*)-6-Chloro-4-phenyl-3-(3-(4-(trifluoromethyl)phenyl)acryloyl)quinolin-2(1*H*)-one (**92b**)

Compound **92b** was synthesized via general procedure A using **60a** (760 mg, 2.50 mmol) and 4-(trifluoromethyl)benzaldehyde **68** (341 μL, 2.50 mmol). Purification was performed by direct phase flash chromatography (SiO_2_ gold 40 g; 0–5% EtOH/DCM) to afford **92b** (820 mg, yield 71%). ^1^H NMR (400 MHz, DMSO-*d*_6_) δ 12.38 (s, 1H), 7.89 (d, *J* = 8.2 Hz, 2H), 7.74 (d, *J* = 8.4 Hz, 2H), 7.67 (dd, *J* = 8.8, 2.4 Hz, 1H), 7.62–7.57 (m, 1H), 7.53–7.40 (m, 4H), 7.36–7.31 (m, 2H), 6.99 (d, *J* = 2.3 Hz, 1H), 6.91 (d, *J* = 16.5 Hz, 1H). ^13^C NMR (101 MHz, DMSO-*d*_6_) δ 193.90, 159.39, 147.09, 144.03, 138.29, 137.58, 133.52, 132.23, 131.05, 130.34, 130.07, 130.02, 129.59, 129.19, 128.88, 128.48, 126.12, 125.68, 125.65, 125.62, 125.30, 122.59, 120.55, 117.74. *t*_R_ = 1.65 min (apolar method). ESI-MS for C_25_H_15_ClF_3_NO_2_: calculated 453.1, found *m*/*z* 454.4, 456.4 [M + H]^+^; 452.4, 454.4 [M – H]^−^.

### 6-Chloro-4-phenyl-3-(5-(4-(trifluoromethyl)phenyl)-4,5-dihydro-1*H*-pyrazol-3-yl)quinolin-2(1*H*)-one (**92c**)

Compound **92c** was synthesized via general procedure B using **92b** (752 mg, 1.70 mmol) with hydrazine hydrate (165 μL, 3.40 mmol). Purification was performed by direct phase flash chromatography (SiO_2_ gold 40 g; 0–5% EtOH/DCM) to afford **92c** (696 mg, yield 90%). ^1^H NMR (400 MHz, DMSO-*d*_6_) δ 12.21 (s, 1H), 7.63–7.56 (m, 3H), 7.56–7.44 (m, 3H), 7.42 (d, *J* = 8.8 Hz, 1H), 7.37–7.32 (m, 1H), 7.29 (d, *J* = 8.5 Hz, 3H), 7.25–7.20 (m, 1H), 6.90 (d, *J* = 2.3 Hz, 1H), 4.75–4.64 (m, 1H), 3.39–3.25 (m, 1H), 2.63–2.54 (m, 1H). ^13^C NMR (101 MHz, DMSO-*d*_6_) δ 160.49, 148.49, 148.37, 145.45, 136.99, 135.13, 130.43, 129.25, 128.63, 128.29, 128.23, 128.20, 127.68, 127.22, 126.58, 125.79, 125.64, 125.11, 125.08, 122.95, 120.88, 117.37, 62.16, 44.50. *t*_R_ = 2.52 min (generic method). ESI-MS for C_25_H_17_ClF_3_N_3_O: calculated 467.1, found *m*/*z* 468.4, 469.5 [M + H]^+^, 466.4, 468.4 [M – H]^−^.

### (*E*)-6-Chloro-3-(3-(furan-2-yl)acryloyl)-4-phenylquinolin-2(1*H*)-one (**93b**)

Compound **93b** was synthesized via general procedure A using **60a** (503 mg, 1.70 mmol) and furan-2-carbaldehyde **69** (147 μL, 1.70 mmol). Purification was performed by direct phase flash chromatography (SiO_2_ gold 24 g; 0–5% EtOH/DCM) to afford **93b** (471 mg, yield 74%). ^1^H NMR (400 MHz, DMSO-*d*_6_) δ 12.34 (s, 1H), 7.85 (d, *J* = 1.7 Hz, 1H), 7.64 (dd, *J* = 8.8, 2.4 Hz, 1H), 7.51–7.40 (m, 4H), 7.35 (s, 1H), 7.33–7.26 (m, 2H), 6.96 (dd, *J* = 6.9, 2.9 Hz, 2H), 6.63 (dd, *J* = 3.5, 1.8 Hz, 1H), 6.37 (d, *J* = 16.1 Hz, 1H). ^13^C NMR (101 MHz, DMSO-*d*_6_) δ 193.04, 159.41, 150.27, 146.93, 146.60, 137.52, 133.55, 132.45, 132.30, 130.98, 128.84, 128.46, 126.08, 125.58, 123.96, 120.51, 117.69, 117.67, 113.14, 40.19, 39.98. *t*_R_ = 2.24 min (generic method). ESI-MS for C_22_H_14_ClNO_3_: calculated 375.1, found *m*/*z* 376.3, 378.3 [M + H]^+^; 374.3, 376.4 [M – H]^−^.

### 6-Chloro-3-(5-(furan-2-yl)-4,5-dihydro-1*H*-pyrazol-3-yl)-4-phenylquinolin-2(1*H*)-one (**93c**)

Compound **93c** was synthesized via general procedure B using **93b** (442 mg, 1.20 mmol) with hydrazine hydrate (117 μL, 2.40 mmol). Purification was performed by direct phase flash chromatography (SiO_2_ gold 40 g; 0–7.5% EtOH/DCM) to afford **93c** (422 mg, yield 91%). ^1^H NMR (400 MHz, DMSO-*d*_6_) δ 12.21 (s, 1H), 7.57 (dd, *J* = 8.8, 2.3 Hz, 1H), 7.54–7.51 (m, 1H), 7.50–7.38 (m, 4H), 7.31–7.23 (m, 2H), 7.07 (d, *J* = 2.8 Hz, 1H), 6.90 (d, *J* = 2.3 Hz, 1H), 6.34 (dd, *J* = 3.1, 1.9 Hz, 1H), 6.17 (d, *J* = 3.2 Hz, 1H), 4.61–4.53 (m, 1H), 3.04 (dd, *J* = 16.2, 10.6 Hz, 1H), 2.87 (dd, *J* = 16.2, 9.2 Hz, 1H). ^13^C NMR (151 MHz, DMSO-*d*_6_) δ 160.55, 155.04, 148.48, 146.34, 142.11, 137.05, 135.23, 130.50, 128.95, 128.90, 128.34, 128.26, 128.18, 126.54, 125.83, 125.74, 120.91, 117.42, 110.33, 105.94, 40.63, 40.06. *t*_R_ = 2.14 min (generic method). ESI-MS for C_22_H_16_ClN_3_O_2_: calculated 389.1, found *m*/*z* 390.4, 392.3 [M + H]^+^; 388.4, 390.4 [M – H]^−^.

### (*E*)-6-Chloro-3-(3-(4′-fluoro-[1,1′-biphenyl]-4-yl)acryloyl)-4-phenylquinolin-2(1*H*)-one (**94b**)

Compound **94b** was synthesized via general procedure A using **60a** (214 mg, 0.72 mmol) and 4′-fluoro-[1,1′-biphenyl]-4-carbaldehyde **70** (144 mg, 0.72 mmol). Purification was performed by direct phase flash chromatography (SiO_2_ gold 24 g; 0–7% EtOH/CHCl_3_) to afford **94b** (305 mg, yield 89%). ^1^H NMR (400 MHz, DMSO-*d*_6_) δ 12.35 (s, 1H), 7.82–7.62 (m, 7H), 7.57–7.40 (m, 5H), 7.36–7.26 (m, 4H), 6.98 (d, *J* = 2.3 Hz, 1H), 6.80 (d, *J* = 16.4 Hz, 1H). ^13^C NMR (101 MHz, DMSO-*d*_6_) δ 193.83, 163.40, 160.96, 159.45, 146.77, 145.64, 141.15, 137.55, 135.57, 135.54, 133.63, 133.34, 132.54, 131.52, 131.42, 130.93, 129.33, 128.90, 128.84, 128.80, 128.72, 128.46, 127.29, 126.98, 126.10, 125.60, 120.59, 117.71, 115.91, 115.70. *t*_R_ = 1.90 min (apolar method). ESI-MS for C_30_H_19_ClFNO_2_: calculated 479.1, found *m*/*z* 480.2, 482.2 [M + H]^+^, 478.2, 480.5 [M – H]^−^.

### 6-Chloro-3-(5-(4′-fluoro-[1,1′-biphenyl]-4-yl)-4,5-dihydro-1*H*-pyrazol-3-yl)-4-phenylquinolin-2(1*H*)-one (**94c**)

Compound **94c** was synthesized via general procedure B using **94b** (273 mg, 0.57 mmol) with hydrazine hydrate (55 μL, 1.14 mmol). Purification was performed by direct phase flash chromatography (SiO_2_ gold 24 g; 0–10% EtOH/DCM) to afford **94c** (188 mg, yield 67%). ^1^H NMR (400 MHz, DMSO-*d*_6_) δ 12.18 (s, 1H), 7.71–7.64 (m, 2H), 7.60–7.46 (m, 6H), 7.42 (d, *J* = 8.8 Hz, 1H), 7.37–7.32 (m, 1H), 7.31–7.21 (m, 3H), 7.19–7.13 (m, 3H), 6.90 (d, *J* = 2.3 Hz, 1H), 4.67–4.57 (m, 1H), 3.26 (dd, *J* = 16.6, 11.1 Hz, 1H), 2.60 (dd, *J* = 16.4, 9.5 Hz, 1H). ^13^C NMR (101 MHz, DMSO-*d*_6_) δ 162.98, 160.55, 160.52, 148.35, 145.48, 142.66, 137.78, 136.96, 136.44, 136.41, 135.19, 130.33, 129.22, 128.69, 128.51, 128.43, 128.25, 128.17, 127.06, 126.78, 126.48, 125.75, 125.62, 120.91, 117.32, 115.73, 115.52, 62.49, 44.43. *t*_R_ = 1.72 min (apolar method). ESI-MS for C_30_H_21_ClFN_3_O: calculated 493.1, found *m*/*z* 494.2, 496.1 [M + H]^+^, 492.2, 494.2 [M – H]^−^.

### (*E*)-6-Chloro-3-(3-(4′-chloro-[1,1′-biphenyl]-4-yl)acryloyl)-4-phenylquinolin-2(1*H*)-one (**95b**)

Compound **95b** was synthesized via general procedure A using **60a** (339 mg, 1.10 mmol) and 4′-chloro-[1,1′-biphenyl]-4-carbaldehyde **71** (238 mg, 1.10 mmol). Purification was performed by direct phase flash chromatography (SiO_2_ gold 24 g; 0–5% EtOH/DCM) to afford **95b** (567 mg, quantitative yield). ^1^H NMR (400 MHz, DMSO-*d*_6_) δ 12.35 (s, 1H), 7.78–7.68 (m, 6H), 7.65 (dd, *J* = 8.8, 2.4 Hz, 1H), 7.57–7.48 (m, 3H), 7.48–7.40 (m, 4H), 7.34 (dd, *J* = 7.8, 1.8 Hz, 2H), 6.99 (d, *J* = 2.3 Hz, 1H), 6.81 (d, *J* = 16.4 Hz, 1H). ^13^C NMR (101 MHz, DMSO-*d*_6_) δ 193.80, 159.41, 146.75, 145.51, 140.78, 137.87, 137.53, 133.70, 133.61, 132.90, 132.51, 130.92, 129.34, 128.93, 128.88, 128.82, 128.45, 127.42, 126.98, 126.07, 125.58, 120.57, 117.69, 79.15. *t*_R_ = 2.16 min (apolar method). ESI-MS for C_30_H_19_Cl_2_NO_2_: calculated 495.1, found *m*/*z* 496.0, 497.9, 499.9, [M + H]^+^; 494.1, 496.0, 498.0 [M – H]^−^.

### 6-Chloro-3-(5-(4′-chloro-[1,1′-biphenyl]-4-yl)-4,5-dihydro-1*H*-pyrazol-3-yl)-4-phenylquinolin-2(1*H*)-one (**95c**)

Compound **95c** was synthesized via general procedure B using **95b** (558 mg, 1.10 mmol) with hydrazine hydrate (107 μL, 2.20 mmol). Purification was performed by direct phase flash chromatography (SiO_2_ gold 40 g; 0–15% EtOH/DCM) to afford **95c** (501 mg, yield 87%). ^1^H NMR (400 MHz, DMSO-*d*_6_) δ 12.20 (s, 1H), 7.69–7.63 (m, 2H), 7.59–7.46 (m, 8H), 7.41 (d, *J* = 8.7 Hz, 1H), 7.37–7.31 (m, 1H), 7.26–7.20 (m, 1H), 7.17 (d, *J* = 8.3 Hz, 2H), 6.90 (d, *J* = 2.3 Hz, 1H), 4.63 (ddd, *J* = 11.3, 9.5, 3.0 Hz, 1H), 3.27 (dd, *J* = 16.4, 11.1 Hz, 1H), 2.60 (dd, *J* = 16.4, 9.4 Hz, 1H). ^13^C NMR (101 MHz, DMSO-*d*_6_) δ 160.53, 148.37, 145.49, 143.11, 138.74, 137.45, 136.97, 135.20, 132.16, 130.35, 129.23, 128.82, 128.69, 128.28, 128.18, 127.14, 126.77, 126.49, 125.77, 125.63, 120.91, 117.34, 62.49, 44.45. *t*_R_ = 1.99 min (apolar method). ESI-MS for C_30_H_21_Cl_2_N_3_O: calculated 509.1, found *m*/*z* 510.0, 511.9, 514.0 [M + H]^+^; 508.0, 510.0, 511.9 [M – H]^−^.

### (*E*)-3-(3-(4′-Bromo-[1,1′-biphenyl]-4-yl)acryloyl)-6-chloro-4-phenylquinolin-2(1*H*)-one (**96b**)

Compound **96b** was synthesized via general procedure A using **60a** (337 mg, 1.10 mmol) and 4′-bromo-[1,1′-biphenyl]-4-carbaldehyde **72** (287 mg, 1.10 mmol). Purification was performed by direct phase flash chromatography (SiO_2_ gold 24 g; 0–5% EtOH/DCM) to afford **96b** (576 mg, yield 89%). ^1^H NMR (400 MHz, DMSO-*d*_6_) δ 12.35 (s, 1H), 7.80–7.68 (m, 4H), 7.68–7.62 (m, 5H), 7.57–7.39 (m, 5H), 7.34 (dd, *J* = 7.7, 1.8 Hz, 2H), 6.99 (d, *J* = 2.3 Hz, 1H), 6.81 (d, *J* = 16.4 Hz, 1H). ^13^C NMR (101 MHz, DMSO-*d*_6_) δ 193.81, 159.41, 146.76, 145.52, 140.83, 138.24, 137.53, 133.74, 133.60, 132.51, 131.85, 130.92, 129.36, 128.88, 128.82, 128.77, 128.44, 127.43, 126.94, 126.07, 125.58, 121.53, 120.57, 117.69, 79.15. *t*_R_ = 2.20 min (apolar method). ESI-MS for C_30_H_19_BrClNO_2_: calculated 539.0, found *m*/*z* 539.9, 541.9, 543.9 [M + H]^+^; 538.0, 540.0, 542.0 [M – H]^−^.

### 3-(5-(4′-Bromo-[1,1′-biphenyl]-4-yl)-4,5-dihydro-1*H*-pyrazol-3-yl)-6-chloro-4-phenylquinolin-2(1*H*)-one (**96c**)

Compound **96c** was synthesized via general procedure B using **96b** (570 mg, 1.00 mmol) with hydrazine hydrate (97 μL, 2.00 mmol). Purification was performed by direct phase flash chromatography (SiO_2_ gold 24 g; 0–10% EtOH/DCM) to afford **96c** (472 mg, yield 81%). ^1^H NMR (400 MHz, DMSO-*d*_6_) δ 12.20 (s, 1H), 7.68–7.46 (m, 10H), 7.41 (d, *J* = 8.8 Hz, 1H), 7.37–7.33 (m, 1H), 7.26–7.21 (m, 1H), 7.19–7.15 (m, 3H), 6.90 (d, *J* = 2.4 Hz, 1H), 4.63 (ddd, *J* = 11.3, 9.5, 3.1 Hz, 1H), 3.27 (dd, *J* = 16.4, 11.0 Hz, 1H), 2.60 (dd, *J* = 16.4, 9.4 Hz, 1H). ^13^C NMR (101 MHz, DMSO-*d*_6_) δ 160.53, 148.37, 145.48, 143.17, 139.11, 137.48, 136.97, 135.20, 131.74, 130.36, 129.23, 128.69, 128.63, 128.27, 128.18, 127.16, 126.77, 126.45, 125.76, 125.63, 120.91, 120.72, 117.34, 79.15, 62.49, 44.45, 40.15, 39.99, 39.94, 39.73, 39.52, 39.31, 39.10, 38.89. *t*_R_ = 2.05 min (apolar method). ESI-MS for C_30_H_21_BrClN_3_O: calculated 553.0, found *m*/*z* 553.9, 555.9, 557.9 [M + H]^+^, 551.9, 554.0, 556.0 [M – H]^−^.

### (*E*)-6-Chloro-3-(3-(4′-methoxy-[1,1′-biphenyl]-4-yl)acryloyl)-4-phenylquinolin-2(1*H*)-one (**97b**)

Compound **97b** was synthesized via general procedure A using **60a** (430 mg, 1.40 mmol) and 4′-methoxy-[1,1′-biphenyl]-4-carbaldehyde **73** (297 mg, 1.40 mmol). Purification was performed by direct phase flash chromatography (SiO_2_ gold 24 g; 0–10% EtOH/DCM) to afford **97b** (528 mg, yield 74%). ^1^H NMR (400 MHz, DMSO-*d*_6_) δ 12.35 (s, 1H), 7.75–7.62 (m, 6H), 7.55–7.40 (m, 6H), 7.33 (ddt, *J* = 6.7, 3.2, 1.8 Hz, 2H), 7.05–7.00 (m, 2H), 6.98 (d, *J* = 2.4 Hz, 1H), 6.77 (d, *J* = 16.4 Hz, 1H), 3.79 (s, 3H). ^13^C NMR (101 MHz, DMSO-*d*_6_) δ 193.75, 159.42, 159.38, 159.07, 146.68, 145.85, 141.92, 137.52, 133.63, 132.58, 131.32, 131.13, 130.90, 129.30, 128.88, 128.81, 128.69, 128.65, 128.43, 127.84, 126.86, 126.37, 126.22, 126.06, 125.69, 125.57, 120.58, 117.69, 114.43, 55.19, 31.34. *t*_R_ = 1.81 min (apolar method). ESI-MS for C_31_H_22_ClNO_3_: calculated 491.1, found *m*/*z* 492.0, 494.0 [M + H]^+^; 490.0, 492.1 [M – H]^−^.

### 6-Chloro-3-(5-(4′-methoxy-[1,1′-biphenyl]-4-yl)-4,5-dihydro-1*H*-pyrazol-3-yl)-4-phenylquinolin-2(1*H*)-one (**97c**)

Compound **97c** was synthesized via general procedure B using **97b** (522 mg, 1.10 mmol) with hydrazine hydrate (107 μL, 2.20 mmol). Purification was performed by direct phase flash chromatography (SiO_2_ gold 40 g; 0–10% EtOH/DCM) to afford **97c** (412 mg, yield 77%). ^1^H NMR (400 MHz, DMSO-*d*_6_) δ 12.20 (s, 1H), 7.57 (dd, *J* = 8.9, 2.4 Hz, 3H), 7.54–7.44 (m, 5H), 7.41 (d, *J* = 8.8 Hz, 1H), 7.38–7.31 (m, 1H), 7.24 (td, *J* = 4.2, 3.7, 1.8 Hz, 1H), 7.16–7.11 (m, 3H), 7.01 (d, *J* = 8.8 Hz, 2H), 6.90 (d, *J* = 2.3 Hz, 1H), 4.60 (ddd, *J* = 11.0, 9.5, 3.1 Hz, 1H), 3.79 (s, 3H), 3.25 (dd, *J* = 16.4, 11.0 Hz, 1H), 2.60 (dd, *J* = 16.4, 9.5 Hz, 1H). ^13^C NMR (101 MHz, DMSO-*d*_6_) δ 160.54, 158.79, 148.34, 145.49, 141.88, 138.51, 136.97, 135.21, 132.34, 130.35, 129.24, 128.71, 128.27, 128.18, 127.60, 126.99, 126.82, 126.01, 125.75, 125.63, 120.93, 117.33, 114.32, 62.58, 55.14, 44.44. *t*_R_ = 2.60 min (generic method). ESI-MS for C_31_H_24_ClN_3_O_2_: calculated 505.2, found *m*/*z* 506.0, 508.0 [M + H]^+^; 504.1, 506.1 [M – H]^−^.

### (*E*)-6-Chloro-3-(3-(1-methyl-1*H*-indol-5-yl)acryloyl)-4-phenylquinolin-2(1*H*)-one (**98b**)

Compound **98b** was synthesized via general procedure A using **60a** (545 mg, 1.80 mmol) and 1-methyl-1*H*-indole-5-carbaldehyde **74** (287 mg, 1.80 mmol). Purification was performed by direct phase flash chromatography (SiO_2_ gold 24 g; 0–10% EtOH/DCM) to afford **98b** (427 mg, yield 53%). ^1^H NMR (400 MHz, DMSO-*d*_6_) δ 12.31 (s, 1H), 7.86 (d, *J* = 1.5 Hz, 1H), 7.65 (dd, *J* = 8.8, 2.3 Hz, 1H), 7.56 (d, *J* = 16.3 Hz, 1H), 7.51–7.30 (m, 9H), 6.97 (d, *J* = 2.3 Hz, 1H), 6.65 (d, *J* = 16.3 Hz, 1H), 6.44 (dd, *J* = 3.2, 0.7 Hz, 1H), 3.79 (s, 3H). ^13^C NMR (101 MHz, DMSO-*d*_6_) δ 193.46, 159.48, 148.78, 146.31, 137.77, 137.46, 133.77, 132.92, 131.03, 130.76, 128.89, 128.72, 128.37, 128.15, 125.99, 125.53, 125.36, 124.31, 123.22, 121.09, 120.65, 117.63, 110.33, 101.50, 32.61. *t*_R_ = 2.45 min (generic method). ESI-MS for C_27_H_19_ClN_2_O_2_: calculated 438.1, found *m*/*z* 439.2, 441.1 [M + H]^+^; 437.3, 439.3 [M – H]^−^.

### 6-Chloro-3-(5-(1-methyl-1*H*-indol-5-yl)-4,5-dihydro-1*H*-pyrazol-3-yl)-4-phenylquinolin-2(1*H*)-one (**98c**)

Compound **98c** was synthesized via general procedure B using **98b** (370 mg, 0.84 mmol) with hydrazine hydrate (82 μL, 1.68 mmol). Purification was performed by direct phase flash chromatography (SiO_2_ gold 24 g; 0–5% EtOH/DCM) to afford **98c** (325 mg, yield 85%). ^1^H NMR (400 MHz, DMSO-*d*_6_) δ 12.20 (s, 1H), 7.57–7.46 (m, 4H), 7.41 (d, *J* = 8.8 Hz, 1H), 7.35 (dt, *J* = 7.2, 1.7 Hz, 1H), 7.31–7.21 (m, 4H), 7.02 (s, 1H), 6.91 (td, *J* = 3.9, 1.7 Hz, 2H), 6.34 (dd, *J* = 3.0, 0.7 Hz, 1H), 4.66 (t, *J* = 10.5 Hz, 1H), 3.75 (s, 3H), 3.22 (dd, *J* = 16.4, 11.0 Hz, 1H), 2.63 (dd, *J* = 16.4, 9.9 Hz, 1H). ^13^C NMR (101 MHz, DMSO-*d*_6_) δ 160.60, 148.21, 145.51, 136.97, 135.70, 135.30, 133.87, 130.28, 129.72, 129.24, 128.74, 128.27, 128.17, 127.79, 127.03, 125.76, 125.62, 120.96, 120.01, 118.09, 117.32, 109.44, 100.21, 63.57, 44.92, 32.45. *t*_R_ = 2.38 min (generic method). ESI-MS for C_27_H_21_ClN_4_O: calculated 452.1, found *m*/*z* 453.2, 455.2 [M + H]^+^; 451.3, 453.3 [M – H]^−^.

### (*E*)-6-Chloro-3-(3-(1-ethyl-1*H*-indol-5-yl)acryloyl)-4-phenylquinolin-2(1*H*)-one (**99b**)

Compound **99b** was synthesized via general procedure A using **60a** (402 mg, 1.30 mmol) and 1-ethyl-1*H*-indole-5-carbaldehyde **75** (225 mg, 1.30 mmol). Purification was performed by direct phase flash chromatography (SiO_2_ gold 40 g; 0–5% EtOH/DCM) to afford **99b** (395 mg, yield 65%). ^1^H NMR (400 MHz, DMSO-*d*_6_) δ 12.31 (s, 1H), 7.85 (d, *J* = 1.4 Hz, 1H), 7.65 (dd, *J* = 8.8, 2.4 Hz, 1H), 7.56 (d, *J* = 16.3 Hz, 1H), 7.50–7.45 (m, 3H), 7.45–7.38 (m, 4H), 7.33 (dd, *J* = 7.7, 1.8 Hz, 2H), 6.97 (d, *J* = 2.3 Hz, 1H), 6.65 (d, *J* = 16.2 Hz, 1H), 6.45 (d, *J* = 3.1 Hz, 1H), 4.20 (q, *J* = 7.2 Hz, 2H), 1.34 (t, *J* = 7.2 Hz, 3H). ^13^C NMR (101 MHz, DMSO-*d*_6_) δ 193.47, 159.48, 148.79, 146.30, 137.46, 136.80, 133.77, 132.92, 130.76, 129.42, 128.98, 128.89, 128.72, 128.66, 128.37, 128.30, 125.99, 125.53, 125.35, 124.27, 123.35, 121.02, 120.65, 117.63, 110.32, 101.75, 79.15, 15.44. *t*_R_ = 2.56 min (generic method). ESI-MS for C_28_H_21_ClN_2_O_2_: calculated 452.1, found *m*/*z* 453.2, 455.2 [M + H]^+^; 451.3, 453.3 [M – H]^−^.

### 6-Chloro-3-(5-(1-ethyl-1*H*-indol-5-yl)-4,5-dihydro-1*H*-pyrazol-3-yl)-4-phenylquinolin-2(1*H*)-one (**99c**)

Compound **99c** was synthesized via general procedure B using **99b** (336 mg, 0.79 mmol) with hydrazine hydrate (77 μL, 1.58 mmol). Purification was performed by direct phase flash chromatography (SiO_2_ gold 24 g; 0–10% EtOH/DCM) to afford **99c** (322 mg, yield 87%). ^1^H NMR (400 MHz, DMSO-*d*_6_) δ 12.18 (s, 1H), 7.60–7.48 (m, 4H), 7.41 (d, *J* = 8.8 Hz, 1H), 7.38–7.30 (m, 3H), 7.25 (dt, *J* = 6.6, 2.1 Hz, 2H), 7.00 (d, *J* = 3.2 Hz, 1H), 6.89 (dd, *J* = 9.7, 2.0 Hz, 2H), 6.34 (dd, *J* = 3.2, 0.8 Hz, 1H), 4.64 (td, *J* = 10.4, 3.2 Hz, 1H), 4.17 (q, *J* = 7.2 Hz, 2H), 3.21 (dd, *J* = 16.4, 11.0 Hz, 1H), 2.63 (dd, *J* = 16.4, 9.9 Hz, 1H), 1.33 (t, *J* = 7.2 Hz, 3H). ^13^C NMR (101 MHz, DMSO-*d*_6_) δ 160.59, 148.22, 145.47, 136.97, 135.29, 134.66, 133.82, 130.30, 129.23, 128.75, 128.27, 128.18, 128.12, 127.94, 127.04, 125.74, 125.63, 120.97, 119.94, 118.22, 117.33, 109.48, 100.42, 63.56, 56.02, 44.87, 18.54, 15.49. *t*_R_ = 2.51 min (generic method). ESI-MS for C_28_H_23_ClN_4_O: calculated 466.2, found *m*/*z* 467.2, 469.2 [M + H]^+^; 465.3, 467.4 [M – H]^−^.

### (*E*)-6-Chloro-3-(3-(1-ethyl-1*H*-indazol-5-yl)acryloyl)-4-phenylquinolin-2(1*H*)-one (**100b**)

Compound **100b** was synthesized via general procedure A using **60a** (292 mg, 0.98 mmol) and 1-ethyl-1*H*-indazole-5-carbaldehyde **76** (171 mg, 0.98 mmol). Purification was performed by direct phase flash chromatography (SiO_2_ gold 24 g; 0–5% EtOH/DCM) to afford **100b** (275 mg, yield 61%). ^1^H NMR (400 MHz, DMSO-*d*_6_) δ 12.33 (s, 1H), 8.09 (s, 1H), 8.07 (d, *J* = 1.4 Hz, 1H), 7.72 (dd, *J* = 8.9, 1.6 Hz, 1H), 7.69–7.57 (m, 3H), 7.48 (d, *J* = 8.8 Hz, 1H), 7.46–7.36 (m, 3H), 7.34 (dd, *J* = 7.8, 1.8 Hz, 2H), 6.98 (d, *J* = 2.3 Hz, 1H), 6.74 (d, *J* = 16.3 Hz, 1H), 4.43 (q, *J* = 7.2 Hz, 2H), 1.38 (t, *J* = 7.2 Hz, 3H). ^13^C NMR (101 MHz, DMSO-*d*_6_) δ 193.64, 159.45, 147.35, 146.51, 139.48, 137.50, 133.76, 133.70, 132.72, 130.84, 128.89, 128.76, 128.40, 126.97, 126.03, 125.64, 125.55, 125.18, 123.97, 123.80, 120.63, 117.66, 110.26, 43.20, 14.85. *t*_R_ = 2.35 min (generic method). ESI-MS for C_27_H_20_ClN_3_O_2_: calculated 453.1, found *m*/*z* 454.4, 456.4 [M + H]^+^, 452.4, 454.4 [M – H]^−^.

### 6-Chloro-3-(5-(1-ethyl-1*H*-indazol-5-yl)-4,5-dihydro-1*H*-pyrazol-3-yl)-4-phenylquinolin-2(1*H*)-one (**100c**)

Compound **100c** was synthesized via general procedure B using **100b** (260 mg, 0.57 mmol) with hydrazine hydrate (55 μL, 1.14 mmol). Purification was performed by direct phase flash chromatography (SiO_2_ gold 24 g; 0–10% EtOH/DCM) to afford **100c** (228 mg, yield 86%). ^1^H NMR (400 MHz, DMSO-*d*_6_) δ 12.21 (s, 1H), 7.97 (d, *J* = 0.9 Hz, 1H), 7.59–7.46 (m, 5H), 7.42 (d, *J* = 8.8 Hz, 2H), 7.38–7.34 (m, 1H), 7.27–7.21 (m, 1H), 7.17–7.08 (m, 2H), 6.91 (d, *J* = 2.4 Hz, 1H), 4.71 (ddd, *J* = 11.3, 9.3, 2.5 Hz, 1H), 4.41 (q, *J* = 7.2 Hz, 2H), 3.27 (dd, *J* = 16.5, 11.1 Hz, 1H), 2.62 (dd, *J* = 16.5, 9.4 Hz, 1H), 1.38 (t, *J* = 7.2 Hz, 3H). ^13^C NMR (101 MHz, DMSO-*d*_6_) δ 161.06, 148.81, 145.93, 138.63, 137.46, 136.04, 135.71, 134.85, 132.74, 130.82, 129.75, 129.17, 128.76, 128.70, 127.36, 126.93, 126.25, 126.11, 125.62, 124.49, 123.84, 121.42, 118.50, 117.82, 110.00, 109.89, 63.43, 45.22, 43.52, 15.40. *t*_R_ = 2.19 min (generic method). ESI-MS for C_27_H_22_ClN_5_O: calculated 467.1, found *m*/*z* 468.5, 470.5 [M + H]^+^, 466.5, 468.5 [M – H]^−^.

### (*E*)-6-Chloro-3-(3-(2-ethyl-2*H*-indazol-5-yl)acryloyl)-4-phenylquinolin-2(1*H*)-one (**101b**)

Compound **101b** was synthesized via general procedure A using **60a** (307 mg, 1.00 mmol) and 2-ethyl-2*H*-indazole-5-carbaldehyde **77** (174 mg, 1.00 mmol). Purification was performed by direct phase flash chromatography (SiO_2_ gold 24 g; 0–5% EtOH/DCM) to afford **101b** (386 mg, yield 82%). ^1^H NMR (400 MHz, DMSO-*d*_6_) δ 12.33 (s, 1H), 8.48 (s, 1H), 8.02 (s, 1H), 7.65 (dd, *J* = 8.8, 2.4 Hz, 1H), 7.61–7.51 (m, 3H), 7.51–7.38 (m, 4H), 7.34 (dd, *J* = 7.7, 1.9 Hz, 2H), 6.98 (d, *J* = 2.3 Hz, 1H), 6.68 (d, *J* = 16.3 Hz, 1H), 4.44 (q, *J* = 7.3 Hz, 2H), 1.49 (t, *J* = 7.2 Hz, 3H). ^13^C NMR (101 MHz, DMSO-*d*_6_) δ 193.61, 159.46, 148.47, 147.59, 146.48, 137.49, 133.71, 132.78, 130.83, 128.88, 128.76, 128.40, 127.38, 126.02, 125.55, 125.30, 125.16, 123.56, 121.42, 120.63, 117.65, 117.56, 47.87, 15.57. *t*_R_ = 2.22 min (generic method). ESI-MS for C_27_H_20_ClN_3_O_2_: calculated 453.1, found *m*/*z* 454.4, 456.4 [M + H]^+^, 452.4, 454.4 [M – H]^−^.

### 6-Chloro-3-(5-(2-ethyl-2*H*-indazol-5-yl)-4,5-dihydro-1*H*-pyrazol-3-yl)-4-phenylquinolin-2(1*H*)-one (**101c**)

Compound **101c** was synthesized via general procedure B using **101b** (302 mg, 0.60 mmol) with hydrazine hydrate (58 μL, 1.20 mmol). Purification was performed by direct phase flash chromatography (SiO_2_ gold 24 g; 0–10% EtOH/DCM) to afford **101c** (236 mg, yield 76%). ^1^H NMR (400 MHz, DMSO-*d*_6_) δ 12.19 (s, 1H), 8.27 (d, *J* = 0.9 Hz, 1H), 7.59–7.38 (m, 6H), 7.35 (td, *J* = 3.1, 1.5 Hz, 2H), 7.28–7.20 (m, 1H), 7.08 (d, *J* = 3.2 Hz, 1H), 6.98–6.86 (m, 2H), 4.65 (td, *J* = 10.3, 3.1 Hz, 1H), 4.42 (q, *J* = 7.3 Hz, 2H), 3.24 (dd, *J* = 16.5, 11.1 Hz, 1H), 2.60 (dd, *J* = 16.5, 9.6 Hz, 1H), 1.48 (t, *J* = 7.3 Hz, 3H). ^13^C NMR (101 MHz, DMSO-*d*_6_) δ 160.56, 148.29, 147.50, 145.53, 136.97, 135.54, 135.22, 130.32, 129.28, 128.68, 128.27, 128.20, 126.91, 125.76, 125.62, 124.73, 122.79, 121.02, 120.94, 117.33, 117.28, 117.07, 63.27, 47.64, 44.41, 15.77. *t*_R_ = 2.06 min (generic method). ESI-MS for C_27_H_22_ClN_5_O: calculated 467.1, found *m*/*z* 468.5, 470.4 [M + H]^+^, 466.4, 468.5 [M – H]^−^.

### (*E*)-6-Chloro-4-phenyl-3-(3-(1-propyl-1*H*-indazol-5-yl)acryloyl)quinolin-2(1*H*)-one (**102b**)

Compound **102b** was synthesized via general procedure A using **60a** (402 mg, 1.35 mmol) and 1-propyl-1*H*-indazole-5-carbaldehyde **78** (254 mg, 1.35 mmol). Purification was performed by direct phase flash chromatography (SiO_2_ gold 24 g; 0–7% EtOH/DCM) to afford **102b** (401 mg, yield 64%). ^1^H NMR (400 MHz, DMSO-*d*_6_) δ 12.33 (s, 1H), 8.10 (d, *J* = 0.8 Hz, 1H), 8.07 (d, *J* = 1.4 Hz, 1H), 7.72 (dd, *J* = 9.0, 1.6 Hz, 1H), 7.70–7.57 (m, 3H), 7.48 (d, *J* = 8.8 Hz, 1H), 7.44–7.38 (m, 3H), 7.34 (dd, *J* = 7.7, 1.8 Hz, 2H), 6.98 (d, *J* = 2.4 Hz, 1H), 6.74 (d, *J* = 16.3 Hz, 1H), 4.36 (t, *J* = 6.9 Hz, 2H), 1.82 (h, *J* = 7.2 Hz, 2H), 0.80 (t, *J* = 7.4 Hz, 3H). ^13^C NMR (101 MHz, DMSO-*d*_6_) δ 193.66, 159.46, 147.36, 146.52, 140.12, 137.50, 133.80, 133.70, 132.74, 130.85, 128.89, 128.77, 128.41, 126.94, 126.03, 125.65, 125.55, 125.20, 123.98, 123.65, 120.64, 117.67, 110.36, 49.71, 22.82, 11.04. *t*_R_ = 2.51 min (generic method). ESI-MS for C_28_H_22_ClN_3_O_2_: calculated 467.1, found *m*/*z* 468.2, 470.2 [M + H]^+^, 466.3, 468.3 [M – H]^−^.

### 6-Chloro-4-phenyl-3-(5-(1-propyl-1*H*-indazol-5-yl)-4,5-dihydro-1*H*-pyrazol-3-yl)quinolin-2(1*H*)-one (**102c**)

Compound **102c** was synthesized via general procedure B using **102b** (395 mg, 0.84 mmol) with hydrazine hydrate (82 μL, 1.68 mmol). Purification was performed by direct phase flash chromatography (SiO_2_ gold 24 g; 0–10% EtOH/DCM) to afford **102c** (381 mg, yield 94%). ^1^H NMR (400 MHz, DMSO-*d*_6_) δ 12.19 (s, 1H), 8.00–7.91 (m, 1H), 7.59–7.45 (m, 6H), 7.42 (d, *J* = 8.7 Hz, 2H), 7.38–7.31 (m, 1H), 7.24 (dd, *J* = 6.8, 1.9 Hz, 1H), 7.12 (dd, *J* = 8.7, 1.6 Hz, 1H), 6.90 (d, *J* = 2.3 Hz, 1H), 4.70 (t, *J* = 10.3 Hz, 1H), 4.33 (t, *J* = 6.9 Hz, 2H), 3.25 (dd, *J* = 16.5, 11.1 Hz, 1H), 2.62 (dd, *J* = 16.5, 9.5 Hz, 1H), 1.88–1.77 (m, 2H), 0.81 (t, *J* = 7.4 Hz, 3H). ^13^C NMR (101 MHz, DMSO-*d*_6_) δ 160.54, 148.29, 145.42, 138.73, 136.96, 135.44, 135.21, 132.22, 130.31, 129.23, 128.68, 128.25, 128.22, 128.18, 126.87, 125.74, 125.60, 125.13, 123.18, 120.92, 117.98, 117.32, 109.56, 62.94, 49.59, 44.66, 22.81, 11.09. *t*_R_ = 2.34 min (generic method). ESI-MS for C_28_H_24_ClN_5_O: calculated 481.2, found *m*/*z* 482.1, 484.2 [M + H]^+^, 480.2, 482.3 [M – H]^−^.

### (*E*)-6-Chloro-4-phenyl-3-(3-(2-propyl-2*H*-indazol-5-yl)acryloyl)quinolin-2(1*H*)-one (**103b**)

Compound **103b** was synthesized via general procedure A using **60a** (292 mg, 0.98 mmol) and 2-propyl-2*H*-indazole-5-carbaldehyde **79** (184 mg, 0.98 mmol). Purification was performed by direct phase flash chromatography (SiO_2_ gold 24 g; 0–7% EtOH/DCM) to afford **103b** (372 mg, yield 81%). ^1^H NMR (400 MHz, DMSO-*d*_6_) δ 12.33 (s, 1H), 8.47 (s, 1H), 8.04–8.00 (m, 1H), 7.65 (dd, *J* = 8.8, 2.3 Hz, 1H), 7.60–7.51 (m, 3H), 7.47 (d, *J* = 8.8 Hz, 1H), 7.44–7.38 (m, 3H), 7.36–7.32 (m, 2H), 6.97 (d, *J* = 2.3 Hz, 1H), 6.68 (d, *J* = 16.2 Hz, 1H), 4.36 (t, *J* = 6.9 Hz, 2H), 1.96–1.87 (m, 2H), 0.83 (t, *J* = 7.4 Hz, 3H). ^13^C NMR (101 MHz, DMSO-*d*_6_) δ 193.63, 159.46, 148.52, 147.59, 146.47, 137.50, 133.71, 132.79, 130.83, 128.88, 128.77, 128.41, 127.39, 126.02, 125.87, 125.55, 125.31, 123.56, 121.32, 120.63, 117.66, 117.59, 54.36, 23.22, 10.84. *t*_R_ = 2.37 min (generic method). ESI-MS for C_28_H_22_ClN_3_O_2_: calculated 467.1, found *m*/*z* 468.2, 470.2 [M + H]^+^, 466.3, 468.3 [M – H]^−^.

### 6-Chloro-4-phenyl-3-(5-(2-propyl-2*H*-indazol-5-yl)-4,5-dihydro-1*H*-pyrazol-3-yl)quinolin-2(1*H*)-one (**103c**)

Compound **103c** was synthesized via general procedure B using **103b** (342 mg, 0.73 mmol) with hydrazine hydrate (71 μL, 1.46 mmol). Purification was performed by direct phase flash chromatography (SiO_2_ gold 24 g; 0–10% EtOH/DCM) to afford **103c** (316 mg, yield 89%). ^1^H NMR (400 MHz, DMSO-*d*_6_) δ 12.19 (s, 1H), 8.27 (d, *J* = 0.9 Hz, 1H), 7.57 (dd, *J* = 8.8, 2.3 Hz, 1H), 7.55–7.44 (m, 4H), 7.41 (d, *J* = 8.8 Hz, 1H), 7.38–7.33 (m, 2H), 7.24 (dt, *J* = 6.6, 1.9 Hz, 1H), 7.08 (d, *J* = 3.2 Hz, 1H), 6.92 (dd, *J* = 8.9, 1.6 Hz, 1H), 6.90 (d, *J* = 2.4 Hz, 1H), 4.70–4.59 (m, 1H), 4.35 (t, *J* = 6.9 Hz, 2H), 3.24 (dd, *J* = 16.5, 11.1 Hz, 1H), 2.60 (dd, *J* = 16.4, 9.6 Hz, 1H), 1.96–1.87 (m, 2H), 0.82 (t, *J* = 7.4 Hz, 3H). ^13^C NMR (101 MHz, DMSO-*d*_6_) δ 160.58, 148.31, 147.56, 145.50, 136.98, 135.55, 135.23, 130.37, 129.31, 128.70, 128.30, 128.28, 128.23, 126.93, 125.76, 125.64, 124.75, 123.59, 120.95, 120.89, 117.36, 117.31, 117.11, 63.26, 54.20, 44.40, 23.40, 10.89. *t*_R_ = 2.23 min (generic method). ESI-MS for C_28_H_24_ClN_5_O: calculated 481.2, found *m*/*z* 482.2, 484.2 [M + H]^+^, 480.3, 482.3 [M – H]^−^.

### (*E*)-6-Chloro-3-(3-(1-cyclohexyl-1*H*-indazol-5-yl)acryloyl)-4-phenylquinolin-2(1*H*)-one (**104b**)

Compound **104b** was synthesized via general procedure A using **60a** (134 mg, 0.46 mmol) and 1-cyclohexyl-1*H*-indazole-5-carbaldehyde **80** (105 mg, 0.46 mmol). Purification was performed by direct phase flash chromatography (SiO_2_ gold 24 g; 0–5% EtOH/DCM) to afford **104b** (193 mg, yield 83%). ^1^H NMR (400 MHz, DMSO-*d*_6_) δ 12.33 (s, 1H), 8.08 (s, 1H), 8.06 (d, *J* = 1.4 Hz, 1H), 7.72–7.69 (m, 2H), 7.65 (dd, *J* = 8.8, 2.4 Hz, 1H), 7.60 (d, *J* = 16.3 Hz, 1H), 7.48 (d, *J* = 8.8 Hz, 1H), 7.45–7.37 (m, 3H), 7.33 (dd, *J* = 7.7, 1.8 Hz, 2H), 6.98 (d, *J* = 2.4 Hz, 1H), 6.73 (d, *J* = 16.3 Hz, 1H), 4.60 (tt, *J* = 9.6, 4.8 Hz, 1H), 1.98–1.80 (m, 6H), 1.70 (d, *J* = 13.0 Hz, 1H), 1.48 (td, *J* = 12.4, 11.1, 4.6 Hz, 2H), 1.25 (dtd, *J* = 12.9, 9.7, 3.6 Hz, 1H). ^13^C NMR (101 MHz, DMSO-*d*_6_) δ 193.66, 159.47, 147.44, 146.53, 139.15, 137.51, 133.72, 133.57, 132.73, 130.86, 128.90, 128.79, 128.42, 127.00, 126.05, 125.59, 125.56, 125.00, 123.98, 123.66, 120.65, 117.68, 110.37, 56.77, 32.26, 24.98. *t*_R_ = 1.94 min (apolar method). ESI-MS for C_31_H_26_ClN_3_O_2_: calculated 507.2, found *m*/*z* 508.2, 510.2 [M + H]^+^, 506.3, 508.3 [M – H]^−^.

### 6-Chloro-3-(5-(1-cyclohexyl-1*H*-indazol-5-yl)-4,5-dihydro-1*H*-pyrazol-3-yl)-4-phenylquinolin-2(1*H*)-one (**104c**)

Compound **104c** was synthesized via general procedure B using **104b** (187 mg, 0.37 mmol) with hydrazine hydrate (36 μL, 0.74 mmol). Purification was performed by direct phase flash chromatography (SiO_2_ gold 24 g; 0–10% EtOH/DCM) to afford **104c** (172 mg, yield 86%). ^1^H NMR (400 MHz, DMSO-*d*_6_) δ 12.20 (s, 1H), 7.95 (d, *J* = 0.8 Hz, 1H), 7.62–7.55 (m, 2H), 7.50 (dddd, *J* = 13.9, 7.1, 5.1, 4.0 Hz, 3H), 7.44–7.39 (m, 2H), 7.38–7.32 (m, 1H), 7.28–7.20 (m, 1H), 7.15–7.08 (m, 2H), 6.90 (d, *J* = 2.3 Hz, 1H), 4.69 (ddd, *J* = 11.0, 9.5, 3.2 Hz, 1H), 4.55 (tt, *J* = 10.0, 5.3 Hz, 1H), 3.25 (dd, *J* = 16.5, 11.1 Hz, 1H), 2.61 (dd, *J* = 16.5, 9.5 Hz, 1H), 1.88 (ddd, *J* = 20.4, 10.2, 3.5 Hz, 6H), 1.71 (dt, *J* = 13.0, 3.2 Hz, 1H), 1.51 (qd, *J* = 12.1, 11.7, 5.9 Hz, 2H), 1.26 (tdd, *J* = 12.8, 9.5, 4.4 Hz, 1H). ^13^C NMR (101 MHz, DMSO-*d*_6_) δ 160.56, 148.32, 145.45, 137.82, 136.98, 135.55, 135.24, 132.00, 130.35, 129.24, 128.70, 128.29, 128.26, 128.21, 126.89, 125.76, 125.63, 124.92, 123.21, 120.93, 118.01, 117.35, 109.60, 62.97, 56.62, 44.74, 32.30, 25.06. *t*_R_ = 2.65 min (generic method). ESI-MS for C_31_H_28_ClN_5_O: calculated 521.2, found *m*/*z* 522.3, 524.3 [M + H]^+^, 520.3, 522.4 [M – H]^−^.

### (*E*)-6-Chloro-3-(3-(2-cyclohexyl-2*H*-indazol-5-yl)acryloyl)-4-phenylquinolin-2(1*H*)-one (**105b**)

Compound **105b** was synthesized via general procedure A using **60a** (89 mg, 0.30 mmol) and 2-cyclohexyl-2*H*-indazole-5-carbaldehyde **81** (68 mg, 0.30 mmol). Purification was performed by direct phase flash chromatography (SiO_2_ gold 12 g; 0–5% EtOH/DCM) to afford **105b** (136 mg, yield 90%). ^1^H NMR (400 MHz, DMSO-*d*_6_) δ 12.33 (s, 1H), 8.49 (s, 1H), 8.00 (d, *J* = 1.5 Hz, 1H), 7.65 (dd, *J* = 8.8, 2.3 Hz, 1H), 7.59–7.52 (m, 3H), 7.47 (d, *J* = 8.8 Hz, 1H), 7.45–7.38 (m, 3H), 7.35–7.30 (m, 2H), 6.97 (d, *J* = 2.3 Hz, 1H), 6.66 (d, *J* = 16.3 Hz, 1H), 4.45 (tt, *J* = 11.3, 3.8 Hz, 1H), 2.09 (dd, *J* = 13.0, 4.0 Hz, 2H), 1.86 (ddt, *J* = 14.4, 9.8, 5.6 Hz, 4H), 1.76–1.63 (m, 1H), 1.51–1.37 (m, 2H), 1.25 (qt, *J* = 13.1, 3.4 Hz, 1H). ^13^C NMR (101 MHz, DMSO-*d*_6_) δ 193.63, 159.47, 148.04, 147.67, 146.47, 137.49, 133.72, 132.78, 130.84, 128.89, 128.78, 128.41, 127.34, 126.03, 125.55, 125.42, 125.24, 123.78, 123.45, 121.13, 120.65, 117.70, 61.84, 33.12, 24.85, 24.82. *t*_R_ = 2.60 min (generic method). ESI-MS for C_31_H_26_ClN_3_O_2_: calculated 507.2, found *m*/*z* 508.3, 510.2 [M + H]^+^, 506.3, 508.4 [M – H]^−^.

### 6-Chloro-3-(5-(2-cyclohexyl-2*H*-indazol-5-yl)-4,5-dihydro-1*H*-pyrazol-3-yl)-4-phenylquinolin-2(1*H*)-one (**105c**)

Compound **105c** was synthesized via general procedure B using **105b** (130 mg, 0.26 mmol) with hydrazine hydrate (25 μL, 0.52 mmol). Purification was performed by direct phase flash chromatography (SiO_2_ gold 24 g; 0–10% EtOH/DCM) to afford **105c** (117 mg, yield 86%). ^1^H NMR (400 MHz, DMSO-*d*_6_) δ 12.19 (s, 1H), 8.28 (d, *J* = 0.9 Hz, 1H), 7.59–7.44 (m, 5H), 7.41 (d, *J* = 8.8 Hz, 1H), 7.38–7.31 (m, 2H), 7.23 (dt, *J* = 6.5, 2.0 Hz, 1H), 7.07 (d, *J* = 3.2 Hz, 1H), 6.94–6.87 (m, 2H), 4.64 (td, *J* = 9.9, 3.1 Hz, 1H), 4.43 (tt, *J* = 11.3, 3.8 Hz, 1H), 3.23 (dd, *J* = 16.5, 11.1 Hz, 1H), 2.59 (dd, *J* = 16.4, 9.6 Hz, 1H), 2.15–2.03 (m, 2H), 1.90–1.80 (m, 4H), 1.69 (dt, *J* = 12.5, 3.3 Hz, 1H), 1.44 (qt, *J* = 13.0, 3.5 Hz, 2H), 1.35–1.17 (m, 1H). ^13^C NMR (101 MHz, DMSO-*d*_6_) δ 160.58, 148.32, 147.05, 145.53, 136.99, 135.52, 135.24, 130.37, 129.30, 128.70, 128.31, 128.29, 128.24, 126.93, 125.77, 125.65, 124.65, 121.38, 120.96, 120.70, 117.37, 117.34, 117.22, 63.29, 61.58, 44.46, 33.30, 24.93, 24.90. *t*_R_ = 2.47 min (generic method). ESI-MS for C_31_H_28_ClN_5_O: calculated 521.2, found *m*/*z* 522.2, 524.2 [M + H]^+^, 520.2, 522.3 [M – H]^−^.

### (*E*)-6-Chloro-3-(3-(4-(1-ethyl-1*H*-indazol-5-yl)phenyl)acryloyl)-4-phenylquinolin-2(1*H*)-one (**106b**)

Compound **106b** was synthesized via general procedure A using **60a** (137 mg, 0.46 mmol) and 4-(1-ethyl-1*H*-indazol-5-yl)benzaldehyde **82** (115 mg, 0.46 mmol). Purification was performed by direct phase flash chromatography (SiO_2_ gold 24 g; 0–2% EtOH/DCM) to afford **106b** (244 mg, quantitative yield). ^1^H NMR (400 MHz, DMSO-*d*_6_) δ 12.35 (s, 1H), 8.10 (d, *J* = 7.3 Hz, 2H), 7.74 (s, 5H), 7.65 (dd, *J* = 8.8, 2.4 Hz, 1H), 7.59–7.39 (m, 6H), 7.34 (dd, *J* = 7.8, 1.8 Hz, 2H), 6.99 (d, *J* = 2.4 Hz, 1H), 6.80 (d, *J* = 16.3 Hz, 1H), 4.46 (q, *J* = 7.2 Hz, 2H), 1.40 (t, *J* = 7.2 Hz, 3H). ^13^C NMR (101 MHz, DMSO-*d*_6_) δ 193.80, 159.46, 146.73, 145.88, 142.84, 138.45, 137.54, 133.65, 133.16, 132.74, 132.59, 131.71, 130.93, 129.35, 128.91, 128.47, 127.09, 126.97, 126.09, 125.60, 125.35, 124.25, 120.61, 118.93, 117.71, 110.15, 43.17, 14.92. *t*_R_ = 2.64 min (generic method). ESI-MS for C_33_H_24_ClN_3_O_2_: calculated 529.2, found *m*/*z* 530.2, 532.2 [M + H]^+^, 528.3, 530.3 [M – H]^−^.

### 6-Chloro-3-(5-(4-(1-ethyl-1*H*-indazol-5-yl)phenyl)-4,5-dihydro-1*H*-pyrazol-3-yl)-4-phenylquinolin-2(1*H*)-one (**106c**)

Compound **106c** was synthesized via general procedure B using **106b** (233 mg, 0.44 mmol) with hydrazine hydrate (43 μL, 0.88 mmol). Purification was performed by direct phase flash chromatography (SiO_2_ gold 24 g; 0–8% EtOH/DCM) to afford **106c** (181 mg, yield 75%). ^1^H NMR (400 MHz, DMSO-*d*_6_) δ 12.20 (s, 1H), 8.10 (d, *J* = 0.9 Hz, 1H), 7.98 (t, *J* = 1.2 Hz, 1H), 7.74 (d, *J* = 8.8 Hz, 1H), 7.67 (dd, *J* = 8.8, 1.7 Hz, 1H), 7.60–7.47 (m, 6H), 7.42 (d, *J* = 8.8 Hz, 1H), 7.36 (dt, *J* = 6.4, 1.8 Hz, 1H), 7.28–7.24 (m, 1H), 7.20–7.14 (m, 3H), 6.90 (d, *J* = 2.4 Hz, 1H), 4.63 (td, *J* = 10.9, 10.3, 3.2 Hz, 1H), 4.46 (q, *J* = 7.2 Hz, 2H), 3.29–3.20 (m, 1H), 2.62 (dd, *J* = 16.4, 9.4 Hz, 1H), 1.41 (t, *J* = 7.2 Hz, 3H). ^13^C NMR (101 MHz, DMSO-*d*_6_) δ 160.55, 148.36, 145.50, 142.03, 139.35, 138.19, 136.98, 135.22, 132.90, 132.66, 130.37, 129.25, 128.73, 128.29, 128.20, 127.06, 126.83, 126.69, 125.76, 125.64, 125.48, 124.24, 120.94, 118.34, 117.35, 110.00, 62.58, 44.46, 43.14, 14.93. *t*_R_ = 1.49 min (apolar method). ESI-MS for C_33_H_26_ClN_5_O: calculated 543.2, found *m*/*z* 544.1, 546.1 [M + H]^+^, 542.2, 544.1 [M – H]^−^.

### (*E*)-6-Chloro-3-(3-(4-(2-ethyl-2*H*-indazol-5-yl)phenyl)acryloyl)-4-phenylquinolin-2(1*H*)-one (**107b**)

Compound **107b** was synthesized via general procedure A using **60a** (92 mg, 0.31 mmol) and 4-(2-ethyl-2*H*-indazol-5-yl)benzaldehyde **83** (78 mg, 0.31 mmol). Purification was performed by direct phase flash chromatography (SiO_2_ gold 12 g; 0–10% EtOH/DCM) to afford **107b** (150 mg, yield 91%). ^1^H NMR (400 MHz, DMSO-*d*_6_) δ 12.35 (s, 1H), 8.44 (d, *J* = 0.8 Hz, 1H), 8.04 (t, *J* = 1.3 Hz, 1H), 7.73 (s, 4H), 7.70–7.63 (m, 2H), 7.59 (dd, *J* = 9.1, 1.8 Hz, 1H), 7.57–7.42 (m, 5H), 7.34 (dd, *J* = 7.8, 1.8 Hz, 2H), 6.99 (d, *J* = 2.3 Hz, 1H), 6.79 (d, *J* = 16.4 Hz, 1H), 4.47 (q, *J* = 7.3 Hz, 2H), 1.52 (t, *J* = 7.3 Hz, 3H). ^13^C NMR (101 MHz, DMSO-*d*_6_) δ 193.79, 159.45, 147.56, 146.70, 145.88, 143.10, 137.54, 133.65, 132.67, 132.59, 131.84, 130.91, 129.31, 128.90, 128.83, 128.45, 126.92, 126.07, 125.58, 124.89, 124.04, 121.88, 120.60, 118.56, 117.70, 117.56, 47.83, 15.72. *t*_R_ = 2.48 min (generic method). ESI-MS for C_33_H_24_ClN_3_O_2_: calculated 529.2, found *m*/*z* 530.1, 532.1 [M + H]^+^, 528.2, 530.2 [M – H]^−^.

### 6-Chloro-3-(5-(4-(2-ethyl-2*H*-indazol-5-yl)phenyl)-4,5-dihydro-1*H*-pyrazol-3-yl)-4-phenylquinolin-2(1*H*)-one (**107c**)

Compound **107c** was synthesized via general procedure B using **107b** (141 mg, 0.27 mmol) with hydrazine hydrate (26 μL, 0.54 mmol). Purification was performed by direct phase flash chromatography (SiO_2_ gold 24 g; 0–15% EtOH/DCM) to afford **107c** (123 mg, yield 83%). ^1^H NMR (400 MHz, DMSO-*d*_6_) δ 8.41 (d, *J* = 0.9 Hz, 1H), 7.92–7.88 (m, 1H), 7.67 (d, *J* = 9.0 Hz, 1H), 7.59–7.48 (m, 7H), 7.42 (d, *J* = 8.8 Hz, 1H), 7.37–7.33 (m, 1H), 7.25 (dq, *J* = 4.9, 1.9 Hz, 1H), 7.16 (dd, *J* = 9.3, 2.7 Hz, 3H), 6.90 (d, *J* = 2.4 Hz, 1H), 4.62 (ddd, *J* = 10.9, 9.4, 3.1 Hz, 1H), 4.47 (q, *J* = 7.3 Hz, 2H), 3.27 (dd, *J* = 16.5, 11.1 Hz, 1H), 2.62 (dd, *J* = 16.4, 9.5 Hz, 1H), 1.52 (t, *J* = 7.3 Hz, 3H). ^13^C NMR (101 MHz, DMSO-*d*_6_) δ 160.67, 148.34, 147.41, 145.61, 141.99, 139.64, 137.15, 135.26, 132.81, 130.34, 129.28, 128.75, 128.31, 128.21, 127.04, 126.81, 126.55, 125.72, 125.64, 125.23, 123.65, 121.93, 120.96, 117.77, 117.44, 62.61, 47.80, 44.49, 15.76. *t*_R_ = 2.35 min (generic method). ESI-MS for C_33_H_26_ClN_5_O: calculated 543.2, found *m*/*z* 544.2, 546.2 [M + H]^+^, 542.3, 544.3 [M – H]^−^.

### (*E*)-6-Chloro-4-phenyl-3-(3-(4-(1-propyl-1*H*-pyrazol-4-yl)phenyl)acryloyl)quinolin-2(1*H*)-one (**108b**)

Compound **108b** was synthesized via general procedure A using **60a** (324 mg, 1.10 mmol) and 4-(1-propyl-1*H*-pyrazol-4-yl)benzaldehyde **84** (236 mg, 1.10 mmol). Purification was performed by direct phase flash chromatography (SiO_2_ gold 24 g; 0–8% EtOH/DCM) to afford **108b** (387 mg, yield 72%). ^1^H NMR (400 MHz, DMSO-*d*_6_) δ 12.33 (s, 1H), 8.26 (d, *J* = 0.8 Hz, 1H), 7.94 (d, *J* = 0.8 Hz, 1H), 7.68–7.55 (m, 5H), 7.50–7.38 (m, 5H), 7.33 (dd, *J* = 7.7, 1.8 Hz, 2H), 6.98 (d, *J* = 2.3 Hz, 1H), 6.73 (d, *J* = 16.4 Hz, 1H), 4.06 (t, *J* = 7.0 Hz, 2H), 1.96–1.87 (m, 2H), 0.84 (t, *J* = 7.4 Hz, 3H). ^13^C NMR (101 MHz, DMSO-*d*_6_) δ 193.72, 159.43, 146.62, 146.14, 137.50, 136.32, 135.30, 133.65, 132.61, 131.69, 130.87, 129.40, 128.88, 128.79, 128.42, 127.62, 126.26, 126.05, 125.56, 125.07, 120.85, 120.59, 117.67, 53.07, 23.07, 10.90. *t*_R_ = 2.46 min (generic method). ESI-MS for C_30_H_24_ClN_3_O_2_: calculated 493.2, found *m*/*z* 494.0, 496.0 [M + H]^+^, 492.0, 494.0 [M – H]^−^.

### 6-Chloro-4-phenyl-3-(5-(4-(1-propyl-1*H*-pyrazol-4-yl)phenyl)-4,5-dihydro-1*H*-pyrazol-3-yl)quinolin-2(1*H*)-one (**108c**)

Compound **108c** was synthesized via general procedure B using **108b** (358 mg, 0.72 mmol) with hydrazine hydrate (70 μL, 1.44 mmol). Purification was performed by direct phase flash chromatography (SiO_2_ gold 24 g; 0–10% EtOH/DCM) to afford **108c** (326 mg, yield 89%). ^1^H NMR (400 MHz, DMSO-*d*_6_) δ 12.18 (s, 1H), 8.13 (d, *J* = 0.8 Hz, 1H), 7.83 (d, *J* = 0.8 Hz, 1H), 7.57 (dd, *J* = 8.8, 2.4 Hz, 1H), 7.51 (tdd, *J* = 6.6, 3.8, 1.8 Hz, 3H), 7.42 (dd, *J* = 8.5, 6.2 Hz, 3H), 7.34 (dt, *J* = 6.8, 1.7 Hz, 1H), 7.26–7.22 (m, 1H), 7.09 (d, *J* = 3.2 Hz, 1H), 7.08–7.03 (m, 2H), 6.90 (d, *J* = 2.3 Hz, 1H), 4.61–4.49 (m, 1H), 4.06 (t, *J* = 7.0 Hz, 2H), 3.22 (dd, *J* = 16.4, 11.0 Hz, 1H), 2.56 (dd, *J* = 16.4, 9.6 Hz, 1H), 1.96–1.87 (m, 2H), 0.85 (t, *J* = 7.4 Hz, 3H). ^13^C NMR (101 MHz, DMSO-*d*_6_) δ 160.53, 148.31, 145.48, 140.99, 136.97, 135.81, 135.21, 131.39, 130.34, 129.22, 128.71, 128.27, 128.19, 126.92, 126.85, 126.79, 125.75, 125.62, 124.76, 121.34, 120.92, 117.33, 62.66, 52.98, 44.43, 23.16, 10.94. *t*_R_ = 2.34 min (generic method). ESI-MS for C_30_H_26_ClN_5_O: calculated 507.2, found *m*/*z* 508.0, 510.1 [M + H]^+^, 506.1, 508.1 [M – H]^−^.

### (*E*)-3-(3-(4-Fluorophenyl)acryloyl)-4-phenylquinolin-2(1*H*)-one (**109b**)

Compound **109b** was synthesized via general procedure A using **61a** (265 mg, 1.00 mmol) and 4-fluorobenzaldehyde **62** (107 μL, 1.00 mmol). Title compound **109b** was obtained after precipitation and filtration from the reaction crude (345 mg, yield 93%). ^1^H NMR (400 MHz, DMSO-*d*_6_) δ 12.30 (s, 1H), 7.73 (d, *J* = 7.2 Hz, 2H), 7.65–7.00 (m, 12H), 6.73 (d, *J* = 16.4 Hz, 1H). ^13^C NMR (101 MHz, DMSO-*d*_6_) δ 194.31, 159.77, 147.76, 144.37, 134.36, 131.36, 130.92, 130.92, 128.92, 128.46, 128.23, 127.52, 126.86, 126.86, 124.54, 122.05, 119.21, 116.00, 115.92, 115.90, 115.88, 115.79. *t*_R_ = 2.25 min (generic method). ESI-MS for C_24_H_16_FNO_2_: calculated 369.1, found *m*/*z* 370.5 [M + H]^+^; 368.4 [M – H]^−^.

### 3-(5-(4-Fluorophenyl)-4,5-dihydro-1*H*-pyrazol-3-yl)-4-phenylquinolin-2(1*H*)-one (**109c**)

Compound **109c** was synthesized via general procedure B using **109b** (300 mg, 0.81 mmol) with hydrazine hydrate (79 μL, 1.62 mmol). Title compound **109c** was obtained after precipitation from DCM (292 mg, yield 94%). ^1^H NMR (400 MHz, DMSO-*d*_6_) δ 12.11 (s, 1H), 7.54–7.44 (m, 4H), 7.40 (dd, *J* = 8.3, 1.2 Hz, 1H), 7.32 (dt, *J* = 7.4, 1.7 Hz, 1H), 7.21 (dt, *J* = 6.8, 1.9 Hz, 1H), 7.14–7.02 (m, 6H), 6.99 (dd, *J* = 8.1, 1.4 Hz, 1H), 4.58 (td, *J* = 10.9, 3.1 Hz, 1H), 3.23 (dd, *J* = 16.4, 11.0 Hz, 1H), 2.56–2.52 (m, 1H). *t*_R_ = 2.13 min (generic method). ESI-MS for C_24_H_18_FN_3_O: calculated 383.1, found *m*/*z* 384.5 [M + H]^+^, 382.5 [M – H]^−^.

### (*E*)-3-(3-(4-Bromophenyl)acryloyl)-4-phenylquinolin-2(1*H*)-one (**110b**)

Compound **110b** was synthesized via general procedure A using **61a** (265 mg, 1.00 mmol) and 4-bromobenzaldehyde **64** (185 mg, 1.00 mmol). Title compound **110b** was obtained after precipitation and filtration from the reaction crude (396 mg, yield 92%). ^1^H NMR (400 MHz, DMSO-*d*_6_) δ 7.67–7.50 (m, 5H), 7.45–7.36 (m, 5H), 7.29 (dd, *J* = 7.7, 1.8 Hz, 2H), 7.15–7.02 (m, 2H), 6.79 (d, *J* = 16.4 Hz, 1H). ^13^C NMR (101 MHz, DMSO-*d*_6_) δ 210.70, 133.70, 131.89, 131.23, 130.49, 129.00, 128.43, 128.25, 126.84, 124.03, 121.73, 119.29, 116.57, 99.54. *t*_R_ = 2.43 min (generic method). ESI-MS for C_24_H_16_BrNO_2_: calculated 429.0, found *m*/*z* 430.4, 432.4 [M + H]^+^; 428.4, 430.4 [M – H]^−^.

### 3-(5-(4-Bromophenyl)-4,5-dihydro-1*H*-pyrazol-3-yl)-4-phenylquinolin-2(1*H*)-one (**110c**)

Compound **110c** was synthesized via general procedure B using **110b** (300 mg, 0.70 mmol) with hydrazine hydrate (68 μL, 1.40 mmol). Purification was performed by direct phase flash chromatography (SiO_2_ gold 24 g; 0.2–3% EtOH/DCM) to afford **110c** (213 mg, yield 69%). ^1^H NMR (400 MHz, DMSO-*d*_6_) δ 12.04 (s, 1H), 7.58–7.36 (m, 7H), 7.31 (dt, *J* = 7.3, 1.6 Hz, 1H), 7.20 (dt, *J* = 7.3, 2.0 Hz, 1H), 7.13–6.95 (m, 5H), 4.56 (ddd, *J* = 11.0, 9.3, 3.3 Hz, 1H), 3.30–3.14 (m, 1H), 2.57–2.51 (m, 1H). ^13^C NMR (101 MHz, DMSO-*d*_6_) δ 160.71, 149.63, 145.96, 143.13, 138.24, 135.77, 131.05, 130.51, 129.28, 128.73, 128.69, 128.10, 128.06, 127.90, 126.94, 125.39, 121.92, 119.82, 119.57, 115.31, 62.01, 44.62. *t*_R_ = 2.31 min (generic method). ESI-MS for C_24_H_18_BrN_3_O: calculated 443.1, found *m*/*z* 444.4, 446.5 [M + H]^+^.

### (*E*)-3-(3-(3-Bromophenyl)acryloyl)-4-phenylquinolin-2(1*H*)-one (**111b**)

Compound **111b** was synthesized via general procedure A using **61a** (265 mg, 1.00 mmol) and 3-bromobenzaldehyde **85** (117 μL, 1.00 mmol). Title compound **111b** was obtained after precipitation and filtration from the reaction crude (380 mg, yield 88%). ^1^H NMR (400 MHz, DMSO-*d*_6_) δ 12.18 (s, 1H), 7.93 (s, 1H), 7.69 (d, *J* = 7.8 Hz, 1H), 7.59 (ddt, *J* = 7.2, 3.9, 1.6 Hz, 2H), 7.51–7.37 (m, 5H), 7.37–7.27 (m, 3H), 7.16 (td, *J* = 7.6, 7.1, 1.2 Hz, 1H), 7.08 (dd, *J* = 8.2, 1.5 Hz, 1H), 6.84 (d, *J* = 16.4 Hz, 1H). ^13^C NMR (101 MHz, DMSO-*d*_6_) δ 194.29, 159.58, 148.05, 143.93, 138.80, 136.83, 134.24, 133.07, 131.37, 131.26, 131.06, 130.87, 128.92, 128.85, 128.52, 128.26, 127.14, 126.93, 122.24, 122.19, 119.23, 115.66. *t*_R_ = 2.40 min (generic method). ESI-MS for C_24_H_16_BrNO_2_: calculated 429.0, found *m*/*z* 430.4, 432.4 [M + H]^+^; 428.3, 430.3 [M – H]^−^.

### 3-(5-(3-Bromophenyl)-4,5-dihydro-1*H*-pyrazol-3-yl)-4-phenylquinolin-2(1*H*)-one (**111c**)

Compound **111c** was synthesized via general procedure B using **111b** (300 mg, 0.70 mmol) with hydrazine hydrate (68 μL, 1.40 mmol). Purification was performed by direct phase flash chromatography (SiO_2_ gold 24 g; 1–5% EtOH/DCM) to afford **111c** (256 mg, yield 84%). ^1^H NMR (400 MHz, DMSO-*d*_6_) δ 12.06 (s, 1H), 7.56–7.36 (m, 7H), 7.36–7.29 (m, 1H), 7.26–7.18 (m, 2H), 7.18–7.06 (m, 3H), 7.01 (dd, *J* = 8.2, 1.4 Hz, 1H), 4.59 (ddd, *J* = 11.1, 9.3, 3.4 Hz, 1H), 3.24 (dd, *J* = 16.6, 11.2 Hz, 1H), 2.56 (dd, *J* = 16.6, 9.4 Hz, 1H). ^13^C NMR (101 MHz, DMSO-*d*_6_) δ 160.67, 149.59, 146.52, 145.91, 138.21, 135.73, 130.47, 130.39, 129.66, 129.25, 129.10, 128.64, 128.02, 128.00, 127.87, 126.89, 125.61, 125.30, 121.87, 121.52, 119.47, 115.27, 61.94, 44.57. *t*_R_ = 2.30 min (generic method). ESI-MS for C_24_H_18_BrN_3_O: calculated 443.0, found *m*/*z* 444.5, 446.4 [M + H]^+^; 442.6, 444.4 [M – H]^−^.

### (*E*)-3-(3-(4-Chlorophenyl)acryloyl)-4-phenylquinolin-2(1*H*)-one (**112b**)

Compound **112b** was synthesized via general procedure A using **61a** (169 mg, 0.64 mmol) and 4-chlorobenzaldehyde **63** (90 mg, 0.64 mmol). Title compound **112b** was obtained after precipitation and filtration from the reaction crude (246 mg, quantitative yield). ^1^H NMR (400 MHz, DMSO-*d*_6_) δ 12.18 (s, 1H), 7.71 (d, *J* = 8.3 Hz, 2H), 7.59 (td, *J* = 7.6, 7.0, 1.5 Hz, 1H), 7.52–7.37 (m, 7H), 7.34–7.29 (m, 2H), 7.17 (t, *J* = 7.6 Hz, 1H), 7.12–7.05 (m, 1H), 6.79 (d, *J* = 16.4 Hz, 1H). ^13^C NMR (101 MHz, DMSO-*d*_6_) δ 194.66, 160.05, 148.50, 144.66, 139.25, 135.67, 134.74, 133.75, 131.78, 131.55, 130.77, 129.39, 129.00, 128.73, 128.65, 127.42, 122.69, 119.69, 116.13. *t*_R_ = 2.37 min (generic method). ESI-MS for C_24_H_16_ClNO_2_: calculated 385.1, found *m*/*z* 386.4, 388.4 [M + H]^+^; 384.4, 386.4 [M – H]^−^.

### 3-(5-(4-Chlorophenyl)-4,5-dihydro-1*H*-pyrazol-3-yl)-4-phenylquinolin-2(1*H*)-one (**112c**)

Compound **112c** was synthesized via general procedure B using **112b** (225 mg, 0.58 mmol) with hydrazine hydrate (56 μL, 1.16 mmol). Purification was performed by direct phase flash chromatography (SiO_2_ gold 24 g; 1–10% EtOH/DCM) to afford **112c** (185 mg, yield 79%). ^1^H NMR (400 MHz, DMSO-*d*_6_) δ 12.05 (s, 1H), 7.56–7.43 (m, 4H), 7.40 (dd, *J* = 8.3, 1.2 Hz, 1H), 7.35–7.26 (m, 3H), 7.21 (dt, *J* = 7.3, 2.0 Hz, 1H), 7.13–7.06 (m, 4H), 7.00 (dd, *J* = 8.2, 1.4 Hz, 1H), 4.58 (ddd, *J* = 10.9, 9.2, 3.2 Hz, 1H), 3.25 (dd, *J* = 16.5, 11.1 Hz, 1H), 2.58–2.52 (m, 1H). ^13^C NMR (101 MHz, DMSO-*d*_6_) δ 161.18, 150.09, 146.43, 143.16, 138.71, 136.24, 131.79, 130.97, 129.76, 129.16, 128.82, 128.60, 128.56, 128.53, 128.36, 127.41, 125.88, 122.39, 120.04, 115.78, 62.43, 45.12. *t*_R_ = 2.26 min (generic method). ESI-MS for C_24_H_18_ClN_3_O: calculated 399.1, found *m*/*z* 400.4, 402.4 [M + H]^+^; 398.4, 400.5 [M – H]^−^.

### (*E*)-3-(3-(4-Methoxyphenyl)acryloyl)-4-phenylquinolin-2(1*H*)-one (**113b**)

Compound **113b** was synthesized via general procedure A using **61a** (265 mg, 1.00 mmol) and *p*-anisaldehyde **65** (122 μL, 1.00 mmol). Title compound **113b** was obtained after precipitation and filtration from the reaction crude (304 mg, yield 80%). ^1^H NMR (400 MHz, DMSO-*d*_6_) δ 12.16 (s, 1H), 7.66–7.51 (m, 3H), 7.47–7.34 (m, 5H), 7.32–7.27 (m, 2H), 7.14 (t, *J* = 7.7 Hz, 1H), 7.06 (dd, *J* = 8.2, 1.4 Hz, 1H), 6.93 (d, *J* = 8.8 Hz, 2H), 6.60 (d, *J* = 16.3 Hz, 1H), 3.78 (s, 3H). ^13^C NMR (101 MHz, DMSO-*d*_6_) δ 194.07, 161.37, 159.72, 147.51, 145.75, 138.92, 134.40, 131.59, 130.85, 130.48, 128.91, 128.41, 128.19, 126.83, 125.41, 122.04, 119.23, 115.76, 114.36, 55.33. *t*_R_ = 2.19 min (generic method). ESI-MS for C_25_H_19_NO_3_: calculated 381.1, found *m*/*z* 382.5 [M + H]^+^, 380.5 [M – H]^+^.

### 3-(5-(4-Methoxyphenyl)-4,5-dihydro-1*H*-pyrazol-3-yl)-4-phenylquinolin-2(1*H*)-one (**113c**)

Compound **113c** was synthesized via general procedure B using **113b** (300 mg, 0.79 mmol) with hydrazine hydrate (77 μL, 1.58 mmol). Purification was performed by direct phase flash chromatography (SiO_2_ gold 24 g; 0–5% EtOH/DCM) to afford **113c** (212 mg, yield 69%). ^1^H NMR (400 MHz, DMSO-*d*_6_) δ 12.02 (s, 1H), 7.54–7.43 (m, 4H), 7.39 (dd, *J* = 8.3, 1.2 Hz, 1H), 7.31 (dq, *J* = 7.6, 1.4 Hz, 1H), 7.21 (ddd, *J* = 5.3, 4.0, 2.0 Hz, 1H), 7.09 (ddd, *J* = 8.2, 7.0, 1.2 Hz, 1H), 7.00 (dt, *J* = 8.2, 2.2 Hz, 3H), 6.82–6.76 (m, 2H), 4.51 (t, *J* = 10.3 Hz, 1H), 3.72 (s, 3H), 3.16 (dd, *J* = 16.4, 10.9 Hz, 1H), 2.57–2.51 (m, 1H). ^13^C NMR (101 MHz, DMSO-*d*_6_) δ 160.73, 158.22, 149.48, 146.05, 138.21, 135.81, 135.45, 130.43, 129.25, 128.74, 128.08, 128.05, 127.86, 127.59, 126.91, 125.62, 121.88, 119.59, 115.28, 113.57, 62.36, 55.02, 44.63. *t*_R_ = 2.08 min (generic method). ESI-MS for C_25_H_21_N_3_O_2_: calculated 395.1, found *m*/*z* 396.5 [M + H]^+^; 394.6 [M – H]^−^.

### (*E*)-4-Phenyl-3-(3-(4-(trifluoromethyl)phenyl)acryloyl)quinolin-2(1*H*)-one (**114b**)

Compound **114b** was synthesized via general procedure A using **61a** (301 mg, 1.10 mmol) and 4-trifluoromethylbenzaldehyde **68** (150 μL, 1.10 mmol). Title compound **114b** was obtained after precipitation and filtration from the reaction crude (414 mg, yield 87%). ^1^H NMR (400 MHz, DMSO-*d*_6_) δ 12.23 (s, 1H), 7.81 (dd, *J* = 62.7, 7.9 Hz, 4H), 7.67–7.24 (m, 8H), 7.25–7.05 (m, 2H), 6.91 (d, *J* = 16.2 Hz, 1H). ^13^C NMR (101 MHz, DMSO-*d*_6_) δ 194.74, 160.08, 148.71, 143.97, 139.35, 138.85, 134.71, 131.63, 130.31, 129.64, 129.40, 129.04, 128.76, 127.45, 126.14, 122.71, 119.67, 116.20. *t*_R_ = 2.41 min (generic method). ESI-MS for C_25_H_16_F_3_NO_2_: calculated 419.1, found *m*/*z* 420.5 [M + H]^+^; 418.5 [M – H]^−^.

### 4-Phenyl-3-(5-(4-(trifluoromethyl)phenyl)-4,5-dihydro-1*H*-pyrazol-3-yl)quinolin-2(1*H*)-one (**114c**)

Compound **114c** was synthesized via general procedure B using **114b** (256 mg, 0.60 mmol) with hydrazine hydrate (58 μL, 1.20 mmol). Purification was performed by direct phase flash chromatography (SiO_2_ gold 24 g; 0.8–6% EtOH/DCM) to afford **114c** (214 mg, yield 80%). ^1^H NMR (400 MHz, DMSO-*d*_6_) δ 12.05 (s, 1H), 7.60 (d, *J* = 8.1 Hz, 2H), 7.55–7.37 (m, 5H), 7.32 (dd, *J* = 11.5, 7.6 Hz, 3H), 7.24–7.17 (m, 2H), 7.10 (ddd, *J* = 8.2, 7.0, 1.2 Hz, 1H), 7.00 (dd, *J* = 8.2, 1.4 Hz, 1H), 4.69 (ddd, *J* = 11.6, 9.1, 2.8 Hz, 1H), 3.33 (m, 1H), 2.63–2.52 (m, 1H). ^13^C NMR (101 MHz, DMSO-*d*_6_) δ 161.17, 150.18, 148.95, 146.44, 138.72, 136.22, 131.00, 129.76, 129.22, 129.13, 128.78, 128.57, 128.51, 128.37, 128.02, 127.72, 127.42, 126.14, 125.77, 125.62, 125.57, 125.53, 122.40, 120.02, 115.79, 62.59, 45.15. *t*_R_ = 2.30 min (generic method). ESI-MS for C_25_H_18_F_3_N_3_O: calculated 433.1, found *m*/*z* 434.4 [M + H]^+^; 432.5 [M – H]^−^.

### *tert*-Butyl (3-(3-(6-Chloro-2-oxo-4-phenyl-1,2-dihydroquinolin-3-yl)-5-(4-fluorophenyl)-4,5-dihydro-1*H*-pyrazol-1-yl)-3-oxopropyl)carbamate (**119**)

In round-bottom flask, the commercially available 3-((*tert*-butoxycarbonyl)amino)propanoic acid **118** (182 mg, 0.96 mmol), HOBt (156 mg, 1.16 mmol), and EDCI (222 mg, 1.16 mmol) were stirred in DCM (15 mL) at rt for 1 h. Then a solution of the **86c** (400 mg, 0.96 mmol) and Et_3_N (296 μL, 2.12 mmol) in DCM (5 mL) was added. The mixture was stirred at rt overnight. The solvent was removed under reduced pressure, the residue redissolved with EtOAc and then washed with H_2_O, 1 M NaHCO_3_, and finally 10% citric acid. The organic layer was dried over Na_2_SO_4_ and evaporated to dryness. Purification was performed by direct phase flash chromatography (0–30% EtOAc/DCM). Yield 72 mg, 25%. ^1^H NMR (400 MHz, DMSO-*d*_6_) δ 12.40 (s, 1H), 7.65 (dd, *J* = 4.0, 8.0 Hz, 1H), 7.59–7.50 (m, 4H), 7.46 (d, *J* = 8.0 Hz, 1H), 7.43–7.42 (m, 1H), 7.28 (d, *J* = 8.0 Hz, 1H), 7.04 (t, *J* = 8.0 Hz, 2H), 6.93 (d, *J* = 2.0 Hz, 1H), 6.80 (dd, *J* = 4.0, 8.0 Hz, 2H), 6.66 (t, *J* = 8.0 Hz, 1H), 5.32 (dd, *J* = 4.0, 12.0 Hz, 1H), 3.73 (dd, *J* = 12.0, 16.0 Hz, 1H), 2.99 (dd, *J* = 8.0, 12.0 Hz, 2H), 2.76 (dd, *J* = 4.0, 20.0 Hz, 1H) 2.41 (q, *J* = 8.0 Hz, 2H), 1.38 (s, 9H).

### Methyl 3-(Methylsulfonamido)propanoate (**123**)

In a dried round-bottom flask, commercially available methyl 3-aminopropanoate **121** (745 mg, 5.34 mmol) and triethylamine (3.7 mL, 26.68 mmol) were stirred in anhydrous DCM (5.6 mL) prior to the addition of methanesulfonyl chloride **122** (1.65 mL, 10.67 mmol). After stirring at rt for 2 days, the reaction was quenched with NaHCO_3_ sat. solution and extracted three times with CHCl_3_. The organic layer was dried over Na_2_SO_4_ and evaporated to dryness. The title compound was obtained after purification over direct phase flash column chromatography (0–40% EtOAc/petroleum ether). Yield 791 mg, 82%. ^1^H NMR (400 MHz, CDCl_3_-*d*) δ 4.94 (s, 1H), 3.72 (s, 3H), m (3.42–3.37, 2H), 2.97 (s, 3H), 2.64 (t, *J* = 4 Hz, 2H). ^13^C NMR (101 MHz, CDCl_3_-*d*) δ 172.63, 52.18, 40.60, 38.89, 34.58.

### 3-(Methylsulfonamido)propanoic Acid (**124**)

In round-bottom flask, methyl 3-(methylsulfonamido)propanoate **123** (791 mg, 4.36 mmol) was stirred in MeOH/THF (1:1 v/v) prior to the addition of 2 M LiOH (2.5 mL). The reaction was stirred at rt overnight. The reaction mixture was acidified with 1 M HCl_aq_ (pH 2) and extracted three times with ethyl acetate. The organic layer was dried over Na_2_SO_4_, filtered and evaporated to dryness to obtain the title compound. Yield 600 mg, 82%. ^1^H NMR (400 MHz, DMSO-*d*_6_) δ 12.29 (s, 1H), 7.02 (t, *J* = 4.0 Hz, 1H), 7.15 (dd, *J* = 7.0, 12.6, 2H), 2.43 (t, *J* = 7.2 Hz, 2H).

### Biology. ELISA Assay

Competitive ELISA screening assay using biotinylated BRC4 peptide to disrupt the BRC4–RAD51 interaction was performed by modifying the method described by Rajendra et al.^33^ BRC4-biotinylated peptide (N-term biotin-KEPTLLGFHTASGKKVKIAKESLDKVKNLFDEKEQ from Life Technologies) was used to coat 384-well plates (Nunc). After washing with PBS containing 0.05% Tween-20 (PBST) and blocking with the solution BSA 1% /PBST, overnight hybridization with human RAD51 protein (NP_002866 Creative Biomart, NY) was performed. Test compounds were added in dose response from 0.01 to 100 μM in triplicate with constant DMSO 1%. Antibody raised against RAD51 (Millipore) and HRP-secondary antibody staining to develop the 3,3′,5,5′-tetramethylbenzidine signal (Sigma) quenched with 1 M HCl was used as the assay readout. Colorimetric measure was read on a Victor5 (PerkinElmer) plate reader. BRC4 and Rad51 were included in the assay as positive control. Results were analyzed by using GraphPad software.

### Protocol for the Expression and Purification of His-hRAD51

hRAD51 was expressed in *E. coli* Rosetta2(DE3)pLysS cells. A saturated overnight culture of Rosetta2(DE3)pLysS/pET15b-His-hRAD51 was diluted (1:1000) into a fresh TB-5052 autoinduction medium containing ampicillin (100 μg/mL). The flasks were shaken at 200 rpm at 20 °C for 72 h. The pellet was subsequently resuspended in an appropriate volume of buffer A (20 mM Tris-HCl (pH 8.00), 500 mM NaCl, 10 mM imidazole, 5 mM DTT, 10% (v/v) glycerol) supplemented with protease inhibitor cocktail (SIGMAFAST protease inhibitor cocktail tablets, EDTA-50 free). The cell suspension was lysed on ice trough sonication (24 rounds of 30 in.; amplitude 85%; Tip MS72; Bandelin Sonoplus HD2070 sonicator). The disrupted cell suspension was centrifuged for 1 h at 13 000 rpm. The supernatant fraction was filtered with a 0.45 μm (MiniSart syringe filter 0.45 μm) membrane to remove residual particulates before chromatography. The supernatant was applied onto a His-Trap column (His-TrapTM FF 5 mL, GE Healthcare) equilibrated with buffer A. A wash step was performed using 10% of buffer B (20 mM Tris-HCl (pH 8.00), 500 mM NaCl, 500 mM imidazole, 10% (v/v) glycerol). The protein was then eluted with a linear gradient from 10% to 100% of buffer B over 10 column volumes. Fractions (0.5 mL) were collected and analyzed by SDS–PAGE. Collected fractions corresponding to the recombinant protein were dialyzed overnight at 4 °C against buffer C (50 mM Tris-HCl (pH 8.00), 200 mM KCl, 0.25 mM EDTA, 2 mM DTT, 10% (v/v) glycerol). Dialyzed protein was loaded onto an anion exchange column (ResQ, GE Healthcare) equilibrated in buffer C. The elution was performed with a linear gradient of buffer B (50 mM Tris-HCl (pH 8.00), 1 M KCl, 0.25 mM EDTA, 2 mM DTT, 10% (v/v) glycerol). Fractions (0.5 mL) were collected and analyzed by SDS–PAGE. Fractions containing His-hRAD51 were pooled and dialyzed against the storage buffer (20 mM Hepes (pH 8.00), 250 mM KCl, 0.1 mM EDTA, 2 mM DTT, 10% (v/v) glycerol). The protein yield was determined from the optical absorption at 280 nm (extinction coefficient 14 900 M^–1^ cm^–1^) of the final sample.

### Microscale Thermophoresis

The recombinant protein hRAD51 was labeled with the Monolith His-Tag labeling kit RED-tris-NTA 2nd Generation kit (NanoTemper Technologies). MST measurements were simultaneously performed on 16 capillaries containing a constant concentration (25 nM) of labeled RED-tris-NTA 2nd Generation His-hRAD51 protein and 16 different concentrations of **35d** in order to determine a concentration-dependent MST binding curve. The highest **35d** concentration tested was 40 μM. Measurements were carried out in MST buffer (20 mM Hepes (pH 8.00), 250 mM KCl, 0.1 mM EDTA, 5% (v/v) glycerol, 5% DMSO).

### Cell Culture and Treatments

BxPC-3 and Capan-1 cells were grown in RPMI 1640 supplemented with 10% FBS, 100 U/mL penicillin/streptomycin, 2 mM glutamine. All media and supplements were from Sigma-Aldrich. Non-neoplastic, immortalized cells from human kidney (HK-2, ATCC CRL2190) were grown in DMEM:F12 medium containing 40 ng/mL dexamethasone and supplemented as described above.

All cultures were routinely tested for Mycoplasma contamination. Treatments (olaparib and BRCA2-RAD51 disruptors) were administered in culture medium supplemented with 0.6% DMSO. The same amount of DMSO was added to the control, untreated cultures.

### Homologous Recombination Assay

Homologous recombination (HR) was assessed by using a commercially available assay (Norgen). This assay is based on cell transfection with two plasmids that, upon cell entry, recombine. The efficiency of HR can be assessed by real-time PCR, using primer mixtures included in the assay kit. Different primer mixtures allow one to discriminate between the original plasmid backbones and their recombination product.

BxPC3 cells (2 × 10^5^ per well) were seeded in a 24-well plate and allowed to adhere overnight. Co-transfection with the two plasmids was performed in Lipofectamine 2000 (Invitrogen) according to the manufacturer’s instructions. During transfection (5 h), cells were exposed to different doses of RAD51-BRCA2 disruptors, dissolved in RPMI in the presence of 0.6% DMSO. After washing with PBS, cells were harvested, and DNA was isolated using Illustra Tissue and Cell Genomic Prep Mini Spin kit (GE Healthcare). Sample concentration was measured using an ONDA Nano Genius photometer. The efficiency of HR was assessed by real-time PCR, using 25 ng of template, the primer mixtures included in the assay kit and following the protocol indicated by the manufacturer. Data analysis was based on the ΔΔCt method: [recombination product/backbone plasmids]^treated^ versus [recombination product/backbone plasmids]^control^.

### Immunofluorescence

Immunofluorescence was used for studying RAD51 nuclear translocation and for evaluating DNA damage through the detection of γH2AX nuclear foci. To visualize RAD51 in cell nuclei, BxPC-3 cells were seeded on glass coverslips placed in a 6-well culture plate (2 × 10^5^ cells/well) and allowed to adhere overnight. Cultures were then preincubated with 20 μM **35d** for 1 h and subsequently exposed to 50 μM cisplatin for an additional 1.5 h. Medium was removed, and cells were maintained in the presence of 20 μM **35d** for 4 h. After this time, cultures growing on coverslips were fixed in PBS containing 1% formalin for 20 min, permeabilized in 70% ethanol, air-dried, and washed twice with PBS. Samples were incubated in 10% bovine serum albumin (BSA) in PBS for 30 min at 37 °C and subsequently exposed to an anti-RAD51 mouse monoclonal antibody (Santa Cruz Biotechnology, 1:1000 in 5% BSA/PBS) overnight at 4 °C. After washing, coverslips were incubated with an anti-mouse FITC-conjugated secondary antibody (1:1000 in 1% BSA/PBS) for 1 h at 37 °C, washed, air-dried, and mounted with a solution of DAPI (2 μg/mL) and DABCO.

To evaluate DNA damage through γH2AX nuclear foci, BXPC3 and Capan1 cells were seeded on glass coverslips in 6-well tissue culture plate (2 × 10^5^ cells/well) and allowed to adhere overnight. After 48 h treatment with olaparib (10 μM) or **35d** (20 μM) given alone or in combination, cultures growing on coverslips were fixed and treated as described above. For this experiment, the used antibodies were a rabbit polyclonal anti-γH2AX (Abcam, 1:1000 in 5% BSA/PBS) and a secondary anti-rabbit rhodamine-labeled (Novus Biologicals, 1:1000 in 1% BSA/PBS). For both experiments, images were acquired using a Nikon fluorescent microscope equipped with filters for FITC, TRITC, and DAPI. The percentage of cells bearing nuclear foci was estimated by two independent observers, by analyzing 100–250 cells for each treatment sample.

### Cell Viability Assay

Cell viability was assessed with the CellTiter-Glo luminescent cell viability assay from Promega. For this experiment, 1 × 10^4^ cells in 200 μL of culture medium were seeded into each well of a 96-multiwell white body plate and allowed to adhere overnight. After 72 h incubation in the presence of olaparib (10 μM) and the RAD51-BRCA2 disruptors alone or in combination, the plate was allowed to equilibrate at room temperature for 30 min and the CellTiter-Glo reactive was directly added to each well. The plate was kept on a shaker for 10 min to induce cell lysis, and its luminescence was measured with a Fluoroskan Ascent FL reader (Labsystems).

### Cytotoxicity Assay

Cell death was assessed by applying the CellTox Green cytotoxicity assay (Promega). Briefly, BxPC-3 (1 × 10^4^/well) were plated in 96-well plates and treated for 72 h with olaparib (10 μM) and the BRCA2-RAD51 disruptors administered alone or in combination. At the end of treatment, the CellTox dye was added to cell cultures and the green fluorescence signal, which is produced by the binding interaction with dead cell DNA, was measured following the manufacturer’s instructions.

### Assessment of Cell Death with Vital Dyes

BxPC-3 cells were grown on coverslips placed in a 6-multiwell plate (5 × 10^5^ cells/well). After a 72 h treatment with olaparib (10 μM) or **35d** (20 μM) given alone or in combination, wells were rapidly washed with PBS and filled with 500 μL of a PBS solution containing DAPI (4.6 μg/mL) and PI (50 μg/mL). After a 10 min incubation at room temperature under light-shielded condition, they were washed with PBS, fixed (10 min) with 10% neutral buffered formalin solution, and washed again to eliminate the fixative. Coverslips were then applied on glass slides using two drops of mounting media (DABCO). Images were acquired on a Nikon fluorescent microscope equipped with filters for DAPI and PI.

### Micronuclei Visualization

For micronuclei visualization, cells were grown on coverslips placed in a 6-multiwell plate (5 × 10^5^ cells/well). After a 72 h treatment with olaparib (10 μM) or **35d** (20 μM) given alone or in combination, wells were rapidly washed with PBS and then fixed for 10 min with cold methanol. Coverslips were then air-dried and mounted on glass slides using a solution of DAPI (5 μg/mL)/DABCO. Images were acquired on a Nikon fluorescent microscope equipped with filters for DAPI. A cell was considered to contain micronuclei if the following criteria were met: (i) one or more round fluorescent bodies were present in the cytoplasm which did not touch the main nucleus; (ii) they were <^1^/_3_ of the main nucleus diameter; (iii) they were nonrefractile, to exclude foreign bodies. The percentage of cells bearing micronuclei was estimated by two independent observers, by analyzing 100–250 cells for each treatment sample.

## Supporting Information Available

Molecular formula strings and some data (CSV)
Structure representation of Figure 3A (PDB)
Structure representation of Figure 3B (PDB)
Computational methods; separation and ELISA assay results of enantiomers **4d-I** and **4d-II**; experimental procedure and characterization data of aldehydes **67**, **70**, **74**–**84**; ^1^H and ^13^C NMR spectra of final compounds **4d**–**10d**, **14d**–**15d**, **18d**–**57d**; HPLC–MS analysis of selected final compounds **4d**–**10d**, **14d**–**15d**, **18**–**25d**, **35d**, **49d**, **57d** (PDF)

## Supplementary Material

jm9b01526_si_001.csv

jm9b01526_si_002.pdb

jm9b01526_si_003.pdb

## Abbreviations Used

MeCN: acetonitrile
Boc_2_O: di-*tert*-butyl dicarbonate
CHX: cyclohexane
DCM: dichloromethane
DIPEA: *N*,*N*-diisopropylethylamine
DMSO: dimethylsulfoxide
EDC: *N*-(3-dimethylaminopropyl)-*N*′-ethylcarbodiimide hydrochloride
EDCI: 1-ethyl-3-(3-dimethylaminopropyl)carbodiimide
EtOH: ethanol
ESI: electrospray ionization
EtOAc: ethyl acetate
HATU: 1-[bis(dimethylamino)methylene]-1*H*-1,2,3-triazolo[4,5-*b*]pyridinium 3-oxide hexafluorophosphate
HOBt: hydroxybenzotriazole
MS: mass spectrometry
μWave: microwave
MeI: iodomethane
DMF: *N*,*N*-dimethylformamide
MeOH: methanol
NMR: nuclear magnetic resonance
rt: room temperature
THF: tetrahydrofuran
TLC: thin layer chromatography
Et_3_N: triethylamine
DAPI: 4′,6-diamidino-2-phenylindole
PI: propidium iodide
