# Supplementary material for: Synthetic Lethality in Pancreatic Cancer: Discovery of a New RAD51-BRCA2 Small Molecule Disruptor That Inhibits Homologous Recombination and Synergizes with Olaparib
Source: J Med Chem. 2020 Feb 10;63(5):2588–619. doi: 10.1021/acs.jmedchem.9b01526 (PMC7997579; doi:10.1021/acs.jmedchem.9b01526)
Supplement: Supplementary file 4 — jm9b01526_si_004.pdf [file jm9b01526_si_004.pdf]

SUPPORTING INFORMATION

Synthetic Lethality in Pancreatic Cancer: Discovery of a New RAD51-BRCA2 Small Molecule Disruptor That Inhibits Homologous Recombination and Synergizes with Olaparib

Greta Bagnolini,[§,†,+] *Domenico Milano,*[§,+] *Marcella Manerba,*[§,+] *Fabrizio Schipani,*[§] *Jose Antonio Ortega,*[§] *Dario Gioia,*[§] *Federico Falchi,*[§] *Andrea Balboni,*[§,†] *Fulvia Farabegoli,*[†] *Francesca De Franco,*[¦] *Janet Robertson,*[¦] *Roberto Pellicciari,*[¦] *Isabella Pallavicini,*[⊥] *Sebastiano Peri,*[⊥] *Saverio Minucci,*[ǂ,⊥] *Stefania Girotto,*[§] *Giuseppina Di Stefano,*[#] *Marinella Roberti**[†] *and Andrea Cavalli**[§,†]

[§]Computational & Chemical Biology, Istituto Italiano di Tecnologia, via Morego 30, 16163 Genoa, Italy.

[†]Department of Pharmacy and Biotechnology, University of Bologna, Via Belmeloro 6, 40126 Bologna, Italy.

[¦]TES Pharma S.r.l., Via Palmiro Togliatti 22bis, I-06073, Corciano, Perugia, Italy.

[⊥]Department of Biosciences, University of Milan, Via Celoria 26, 20100 Milan.

[ǂ]Department of Experimental Oncology at the IEO, European Institute of Oncology IRCCS, IFOM-IEO Campus, Via Adamello 16, 20100 Milan.

[#]Department of Experimental, Diagnostic and Specialty Medicine, University of Bologna, Via S. Giacomo 14, 40126 Bologna, Italy.

TABLE OF CONTENT

Computational methods.. S4

Separation and ELISA assay results of enantiomers **4d-I** and **4d-II**………………………………S5

Scheme S1. Synthesis of aldehyde **67** ..…………………………………………………….…...S6

Scheme S2. Synthesis of aldehyde **70** ..……………………………………………………....S6

Scheme S3. Synthesis of aldehyde **74** ..…………………………………………………….….. S7

Scheme S4. Synthesis of aldehyde **75** ..……………………………………………….….…….. S8

Scheme S5. Synthesis of aldehydes **76**, **77** ..………………………...…………...……….. S9

Scheme S6. Synthesis of aldehydes **78**, **79** ..……………………….………….…..…….. S10

Scheme S7. Synthesis of aldehydes **80**, **81** ..……………………….………….……....... S11

Scheme S8. Synthesis of aldehydes **82**, **83** ………………………..…………………….. S12

Scheme S9. Synthesis of aldehyde **84** ………………………..……………..……..…… S14

^{1}H-NMR spectrum, ^{13}C-NMR spectrum and HPLC-MS analysis of final compounds **4d-10d**, **14d-15d**, **18d-57d** and HPLC-MS analysis of selected final compounds **4d-10d**, **14d-15d**, **18-25d**, **35d**, **49d**, **57d** ....………………………………………………………………………S16

Computational methods

Protein Preparation

The crystal structure of a RAD51-BRCA2 BRC repeat complex was downloaded from the Protein Data Bank (PDB code 1N0W). The structure was then treated with the Schrödinger Suite 2014-4 Protein Preparation Wizard tool. All the selenomethionines were mutated to methionine, water molecules and ions were removed, and an exhaustive sampling of the orientations of groups, whose hydrogen bonding network needs to be optimized, was performed. Finally, the protein structure was refined to relieve steric clashes with a restrained minimization with the OPLS2005 force field until a final RMSD of 0.30 Å with respect to the input protein coordinates.

Database Preparation

A commercially available library of compounds composed of ASINEX and LifeChemicals databases collected from ZINC was prepared with the LigPrep tool of the Schrödinger Suite. The 2D (smi file) structures were converted to 3D structures and for each entry all stereoisomers were generated. The resulting molecules were submitted to Epik and all the tautomers and ionization states at pH 7.0 ± 2.0 were calculated. Finally, duplicates, compounds with more than 2 chiral centers, Pan-Assay Interference Compounds (PAINS), compounds with Michael acceptor groups, and frequent hitters were deleted. To enrich the database with potential Protein Protein Interaction Inhibitors, the database was filtered with the PPI-HitProfiler tool using the “soft” methods.

High Throughput Docking (Virtual Screening)

All filtered ligands (about 750K) were docked with Glide SP by centering the grid on the position of BRCA **Phe1546**. The 10K top-scoring compounds were re-docked with Glide XP and the 1K top-scoring compounds were selected. Both grid generation and docking calculations were performed with the default settings. The selected compounds were visually inspected to identify compounds able to match the interactions between RAD51 and BRCA and **42** compounds were selected and purchased.

Induced-Fit Docking

IFD (Induced-Fit Docking) calculations were performed with the previously prepared protein structure using both enantiomers of each ligand. All the ligands were prepared using Ligprep utility in Schrödinger 2019-2. The IFD protocol involves the use of the Glide docking program to generate a number of initial possible ligand poses followed by a protein side chain optimization using the

Prime protein structure modeling program. After a number of iterations, the process produces a list of final poses, ordered by a proprietary scoring function (that is a combination of the Prime Energy and Glide scoring function).

The Schrödinger Extended Sampling protocol was selected along with the OPLS3e force field. The grid box was centered on the centroid of residues Tyr205, Arg247, and Phe259. Residues within 5.0 Å of ligand poses were refined during the process. The other parameters were set to their default values. Resulting poses were evaluated by visual inspection.

Separation and ELISA assay results of enantiomers 4d-I and 4d-II

The semi-preparative chiral separations of the racemic **4d** by HPLC were performed on a Waters Alliance HPLC instrument consisting of a 1525 Binary HPLC Pump, Waters Fraction Collector III and a 2998 Photodiode Array Detector. The separations were run in isocratic mode on a Daicel ChiralPak AD column (250x10mmID, particle size 10µm) with a ChiralPak AD Semi-Prep. Guard pre-column (50x10mmID, particle size 10µm). The mobile phase was Heptane-2-Propanol (75:25) with a flowrate = 5mL/min.

To determine the enantiomeric excess (ee) of the enantiomers **4d-I** and **4d-II**, the analytical chiral separations by HPLC were run on a Waters Alliance HPLC instrument consisting of an e2695 Separation Module and a 2998 Photodiode Array Detector. The PDA range was 210-400nm. The analyses were performed in isocratic mode on a Daicel ChiralPak AD column (250x4.6mmID, particle size 10µm). The mobile phase was Heptane-2-Propanol (50:50) with a flow rate = 1mL/min; **4d-I**: t_R = 6.411min., >99.5% ee at 240nm; **4d-II**: t_R = 13.431min., 96.6% ee at 240nm. The ^{1}H NMR spectrum was identical to that of racemic **4d** for each enantiomer.

Table S1.

Compound	Retention time (t_R)	QC (UV) @215 nm	Enantiomeric excess (ee)	EC_{50} ELISA (µM)
**4d-I**	6.411 minutes	97%	>99.5%	4 ± 0.5
**4d-II**	13.431 minutes	99%	96.6%	10 ± 1

Scheme S1: Synthesis of aldehyde 67

The 3-fluoro-4-methoxybenzaldehyde **67** was achieved by the alkylation of the commercially available 3-fluoro-4-hydroxybenzaldehyde **125** with methyl iodide under basic conditions.

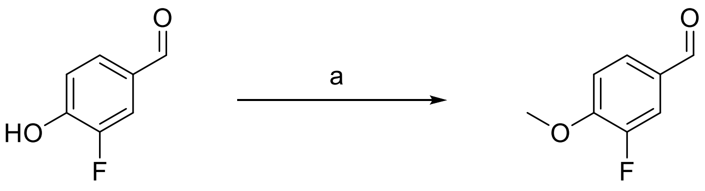

125 **67**

Reagents and conditions. Synthesis of **67**. Reagents and conditions: (a) Potassium carbonate (3.3 equiv), anhydrous DMF, methyl iodide **126** (1.2 equiv), 70 °C, 3.5 h.

3-Fluoro-4-methoxybenzaldehyde (**67**). In a screw capped pressure tube 3-fluoro-4-hydroxybenzaldehyde **125** (461 mg, 3.29 mmol) was dissolved with 3.0 ml of anhydrous DMF then potassium carbonate (1.49 g, 10.8 mmol) was added and the mixture kept under stirring for 5 minutes at rt. Methyl iodide **126** (559 mg, d = 2.28 g/mL, 245 µL, 3.95 mmol) was then added. The reaction was thus heated to 70 °C and stirred for 3.5 hours. The crude mixture was diluted with $CHCl_3$ and washed three times with water. The organic layer was dried through a phase separator and the solvent removed under reduced pressure. The crude was purified by normal phase flash column chromatography (SiO_2 24 g; 0-20% EtOAc/ cyclohexane) to afford **67**. Yield 440 mg, 87%. ^{1}H NMR (400 MHz, DMSO-d_6) δ 9.87 (d, J = 2.1 Hz, 1H), 7.79 (ddd, J = 8.4, 2.0, 1.0 Hz, 1H), 7.69 (dd, J = 11.4, 2.0 Hz, 1H), 7.39 (t, J = 8.4 Hz, 1H), 3.95 (s, 3H). R_t 1.59 min (generic method). ESI-MS for $C_8H_7FO_2$: calculated 154.0, found m/z 155.3 $[M+H]^+$.

Scheme S2: Synthesis of aldehyde 70

4'-Fluoro-[1,1'-biphenyl]-4-carbaldehyde **70** was synthesized taking advantage of Suzuki coupling reaction between the commercially available 4-iodobenzaldehyde **127** and (4-fluorophenyl) boronic acid **128** in presence of palladium(0) catalyst.

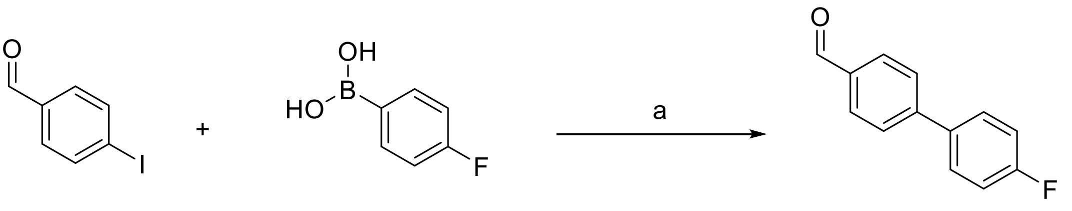

127 **128** **70**

Reagents and conditions: (a) i. Anhydrous sodium carbonate (3.0 equiv); ii. DMF, H_2O, rt, 15 minutes; iii. Tetrakis(triphenylphosphine)palladium(0) (0.15 equiv), 110 °C, 20 h.

4'-Fluoro-[1,1'-biphenyl]-4-carbaldehyde (**70**). In a screw capped pressure tube 4-iodobenzaldehyde **127** (255 mg, 1.1 mmol), (4-fluorophenyl) boronic acid **128** (231 mg, 1.5 mmol) and anhydrous sodium carbonate (350 mg, 3.3 mmol) were added. The tube was deoxygenated with three cycles vacuum/Ar, then DMF (2 mL) and H_2O (400 µL) were added. The solution was stirred at rt, under Ar flux, for 15 minutes. Tetrakis(triphenylphosphine)palladium(0) (185 mg, 0.16 mmol) was thus added, the mixture was heated to 110 °C and stirred for 20 hours. The crude was then diluted with DCM and washed with water (3 x 50 mL). The organic layer was dried over Na_2SO_4 and the solvent was then removed under reduced pressure. Purified by normal phase flash column chromatography (SiO_2 gold 24 g, 0-7% EtOAc/ cyclohexane). Yield 226 mg, 66%. ^{1}H NMR (400 MHz, DMSO-d_6) δ 10.06 (s, 1H), 8.03 – 7.97 (m, 2H), 7.94 – 7.88 (m, 2H), 7.87 – 7.81 (m, 2H), 7.40 – 7.32 (m, 2H). R_t 2.30 min (generic method).

Scheme S3: Synthesis of aldehyde 74

1-Methyl-1H-indole-5-carbaldehyde **74** was achieved by the alkylation of the commercially available 1*H*-indole-5-carboxaldehyde **129** with methyl iodide **126** under basic conditions.

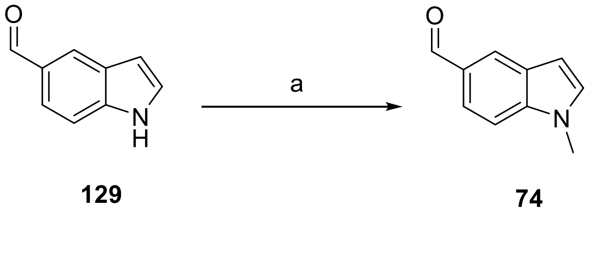

129 **74**

Reagents and conditions: (a) Potassium carbonate (2.0 equiv), anhydrous DMF, methyl iodide **126** (2.0 equiv), 35 °C, 18 h.

1-Methyl-1H-indole-5-carbaldehyde (**74**). In a screw capped pressure tube 1*H*-indole-5-carboxaldehyde **129** (333 mg, 2.3 mmol) was dissolved with 2.0 mL of anhydrous DMF then

potassium carbonate (630 mg, 4.6 mmol) was added and the mixture kept under stirring for 10 minutes at rt. Methyl iodide **126** (647 mg, d = 2.28 g/mL, 284 µL, 4.6 mmol) was then added. The reaction was thus heated to 35 °C and stirred for 18 hours. The crude mixture was diluted with DCM and washed three times with water. The organic layer was anhydrified through a phase separator and the solvent removed under reduced pressure. Purified by normal phase flash column chromatography (SiO_2 gold 24 g, 0- 7.5% EtOAc/ cyclohexane). Yield 286 mg, 81%. ^{1}H NMR (400 MHz, DMSO-d_6) δ 9.98 (s, 1H), 8.18 (d, J = 1.5 Hz, 1H), 7.74 – 7.56 (m, 2H), 7.49 (d, J = 3.2 Hz, 1H), 6.67 (dd, J = 3.1, 0.8 Hz, 1H), 3.85 (d, J = 1.3 Hz, 3H). R_t 1.75 min (generic method). ESI-MS for $C_{10}H_9NO$: calculated 159.1, found m/z 160.1 $[M+H]^+$.

Scheme S4: Synthesis of aldehyde 75

1-Ethyl-1H-indole-5-carbaldehyde **75** was achieved by the alkylation of the commercially available 1*H*-indole-5-carboxaldehyde **129** with iodoethane **130** under basic conditions.

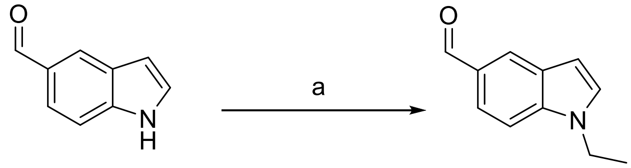

129 **75**

Reagents and conditions: (a) Potassium carbonate (2.0 equiv), anhydrous DMF, iodoethane **130** (2.0 equiv), 35 °C, 18 h.

1-Ethyl-1H-indole-5-carbaldehyde (**75**). In a screw capped pressure tube 1*H*-indole-5-carboxaldehyde **129** (338 mg, 2.3 mmol) was dissolved with 2.0 ml of anhydrous DMF then potassium carbonate (638 mg, 4.6 mmol) was added and the mixture kept under stirring for 10 minutes at rt. Iodoethane **130** (503 mg, d = 1.94 g/mL, 344 µL, 4.6 mmol) was then added. The reaction was thus heated to 35 °C and stirred for 18 hours. The crude mixture was diluted with DCM and washed three times with water. The organic layer was dried through a phase separator and the solvent removed under reduced pressure. Purified by normal phase flash column chromatography (SiO_2 gold 24 g, 0- 5%, EtOAc/ cyclohexane). Yield 363 mg, 91%. ^{1}H NMR (400 MHz, DMSO-d_6) δ 9.98 (s, 1H), 8.18 (t, J = 1.1 Hz, 1H), 7.71 – 7.62 (m, 2H), 7.57 (d, J = 3.2 Hz, 1H), 6.67 (dd, J = 3.2, 0.7 Hz, 1H), 4.27 (q, J = 7.2 Hz, 2H), 1.37 (t, J = 7.2 Hz, 3H). R_t 1.94 min (generic method). ESI-MS for $C_{11}H_{11}NO$: calculated 173.1, found m/z 174.0 $[M+H]^+$.

Scheme S5: Synthesis of aldehydes 76, 77

Ethylindazole-5-carbaldehydes **76** and **77** were synthesized by the alkylation of the commercially available 1*H*-indazole-5-carboxaldehyde **131** with ethyl bromide **132** under basic conditions.

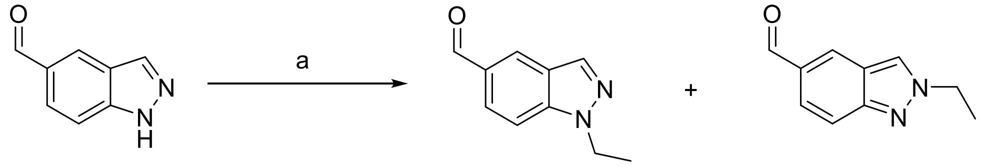

131 **76** **77**

Reagents and conditions: (a) Potassium carbonate (3.0 equiv), anhydrous DMF, ethyl bromide **132** (1.1 equiv), 40 °C, 5 h.

Ethylindazole-5-carbaldehydes (**76, 77**). In a screw capped pressure tube 1*H*-indazole-5-carboxaldehyde **131** (300 mg, 2.84 mmol) was dissolved with 3.0 mL of anhydrous DMF then potassium carbonate (1.13 g, 8.2 mmol) was added and the mixture kept under stirring for 10 minutes at rt. Ethyl bromide **132** (335 mg, d = 1.46 g/mL, 230 µL, 3.1 mmol) was then added. The reaction was thus heated to 40 °C and stirred for 5 hours. The crude mixture was diluted with DCM and washed with water (3x50 mL). The organic layer was anhydrified through a phase separator and the solvent removed under reduced pressure. Purified by normal phase flash column chromatography (SiO_2 gold 24 g, 0- 50% EtOH/ DMC). Mono- and bi-dimensional ^{1}H- and ^{13}C-NMR (HMBC) analyses confirmed the structure of the title compounds named **76** (1-ethyl-1H-indazole-5-carbaldehyde), which showed a positive ^{1}H-^{13}C correlation between C<u>H</u>$_2$ at 4.49 ppm and a quaternary C at 141.7 ppm, and **77** (2-ethyl-2H-indazole-5-carbaldehyde), which showed a positive ^{1}H-^{13}C correlation between C<u>H</u>$_2$ at 4.51 ppm and the C_3 at 127.5 ppm, as shown in the reaction scheme above according to their elution order. Yields: (**76**) 179 mg, 50%; (**77**) 121 mg, 34%.

76) ^{1}H NMR (400 MHz, DMSO-d_6) δ 10.04 (s, 1H), 8.43 (t, *J* = 1.1 Hz, 1H), 8.34 (d, *J* = 0.8 Hz, 1H), 7.92 – 7.81 (m, 2H), 4.50 (q, *J* = 7.2 Hz, 2H), 1.42 (t, *J* = 7.2 Hz, 3H). ^{13}C NMR (101 MHz, DMSO-d_6) δ 192.67, 141.43, 135.64, 130.49, 128.16, 124.80, 123.78, 110.94, 43.85, 15.32. R_t 1.56 min (generic method). ESI-MS for $C_{10}H_{10}N_2O$: calculated 174.1, found *m/z* 175.3 $[M+H]^+$;

77) ^{1}H NMR (400 MHz, DMSO-d_6) δ 9.98 (s, 1H), 8.75 (s, 1H), 8.45 (d, *J* = 1.2 Hz, 1H), 7.69 (qd, *J* = 9.0, 1.3 Hz, 2H), 4.52 (q, *J* = 7.3 Hz, 2H), 1.54 (t, *J* = 7.3 Hz, 3H). ^{13}C NMR (101 MHz,

DMSO-d_6) δ 192.69, 150.17, 130.99, 130.81, 127.49, 122.46, 121.23, 118.24, 48.63, 16.02. R_t 1.36 min (generic method). ESI-MS for $C_{10}H_{10}N_2O$: calculated 174.1, found *m/z* 175.3 $[M+H]^+$.

Scheme S6: Synthesis of aldehydes 78, 79

Propylindazole-5-carbaldehydes **78** and **79** were synthesized by the alkylation of the commercially available 1*H*-indazole-5-carboxaldehyde **131** with 1-bromopropane **133** under basic conditions.

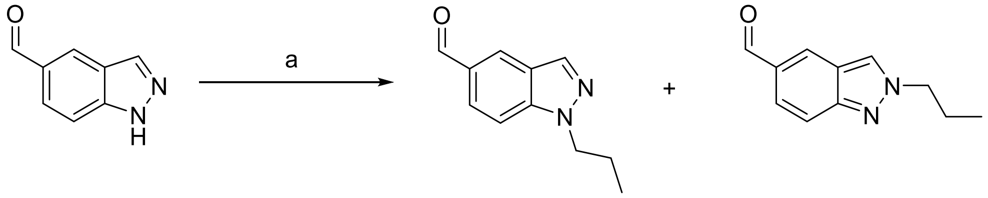

131 **78** **79**

Reagents and conditions: (a) Potassium carbonate (2.6 equiv), anhydrous DMF, 1-bromopropane **133** (2.0 equiv), rt, 14 h.

Propylindazole-5-carbaldehydes (78, 79). In a screw capped pressure tube 1*H*-indazole-5-carboxyaldehyde **131** (152 mg, 1.0 mmol) was dissolved with 1.1 mL of anhydrous DMF then potassium carbonate (359 mg, 2.6 mmol) was added and the mixture kept under stirring for 30 minutes at rt. 1-Bromopropane **133** (256 mg, d = 1.353 g/mL, 189 µL, 2.0 mmol) was then added. The reaction was thus stirred for 14 hours at rt. The crude mixture was diluted with DCM and washed with water (3x50 mL). The organic layer was dried through a phase separator and the solvent removed under reduced pressure. Purified by normal phase flash column chromatography (SiO_2 gold 24 g, 0-70% EtOH/ DCM). Mono- and bi-dimensional ^{1}H- and ^{13}C-NMR (HMBC) analyses confirmed the structure of the title compounds named **78** (1-propyl-1H-indazole-5-carbaldehyde), which showed a positive ^{1}H-^{13}C correlation between $C\underline{H}_2$ at 4.42 ppm and a quaternary C at 142.0 ppm, and **79** (2-propyl-2H-indazole-5-carbaldehyde), which showed a positive ^{1}H-^{13}C correlation between $C\underline{H}_2$ at 4.44 ppm and the C_3 at 128.2 ppm, as shown in the reaction scheme above according to their elution order. Yields: (**78**) 99 mg, 50%; (**79**) 66 mg, 34%.
78) ^{1}H NMR (400 MHz, DMSO-d_6) δ 10.02 (s, 1H), 8.42 (d, *J* = 1.2 Hz, 1H), 8.33 (s, 1H), 7.88 – 7.80 (m, 2H), 4.42 (t, *J* = 6.9 Hz, 2H), 1.85 (h, *J* = 7.2 Hz, 2H), 0.85 – 0.77 (m, 3H). ^{13}C NMR (101 MHz, DMSO-d_6) δ 192.18, 141.58, 135.19, 129.99, 127.69, 124.33, 123.15, 110.55, 49.84, 22.80, 11.02. R_t 1.82 min (generic method). ESI-MS for $C_{11}H_{12}N_2O$: calculated 188.1, found *m/z* 189.1 $[M+H]^+$.

79) ^{1}H NMR (400 MHz, DMSO-d_6) δ 9.97 (s, 1H), 8.73 (s, 1H), 8.44 (d, *J* = 1.4 Hz, 1H), 7.78 – 7.62 (m, 2H), 4.43 (t, *J* = 7.0 Hz, 2H), 1.95 (h, *J* = 7.2 Hz, 2H), 0.85 (t, *J* = 7.4 Hz, 3H). ^{13}C NMR (101 MHz, DMSO-d_6) δ 192.21, 149.74, 130.53, 130.36, 127.71, 121.97, 120.65, 117.78, 54.60, 23.20, 10.83. R_t 1.61 min (generic method). ESI-MS for $C_{11}H_{12}N_2O$: calculated 188.1, found *m*/*z* 189 $[M+H]^+$.

Scheme S7: Synthesis of aldehydes 80, 81

Cyclohexylindazole-5-carbaldehydes **80** and **81** were synthesized by the alkylation of the commercially available 1*H*-indazole-5-carboxaldehyde **131** with 1-bromocyclohexane **134** under basic conditions.

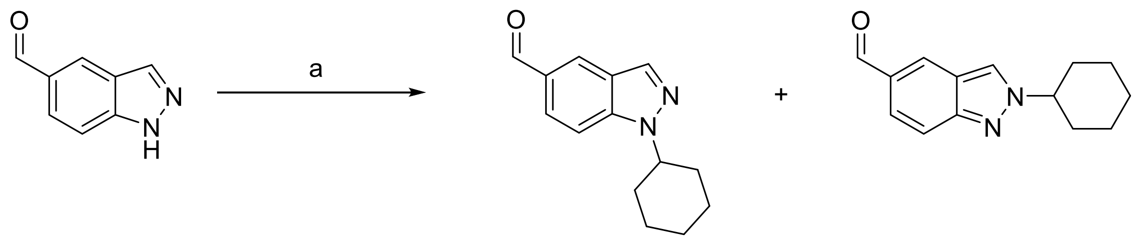

131 **80** **81**

Reagents and conditions: (a) Potassium carbonate (2.1 equiv), anhydrous DMF, 1-bromocyclohexane **134** (2.1 equiv), rt, 6 days.

Cyclohexylindazole-5-carbaldehydes (**80, 81**). In a screw capped pressure tube 1*H*-indazole-5-carboxaldehyde **131** (165 mg, 2.13 mmol) was dissolved with 1.0 mL of anhydrous DMF then potassium carbonate (624 mg, 4.52 mmol) was added and the mixture kept under stirring for 30 minutes at rt. Bromocyclohexane **134** (737 mg, d = 1.335 g/mL, 552 µL, 4.52 mmol) was then added. The reaction was thus stirred at rt for 6 days. The crude mixture was diluted with DCM and washed with water (3x50 mL). The organic layer was dried through a phase separator and the solvent removed under reduced pressure. Purified by normal phase flash column chromatography (SiO_2 gold 24 g, 0-50% EtOH/ DCM). Mono- and bi-dimensional ^{1}H- and ^{13}C-NMR (HMBC) analyses confirmed the structure of the title compounds named **80** (1-cyclohexyl-1H-indazole-5-carbaldehyde) and **81** (2-cyclohexyl-2H-indazole-5-carbaldehyde) according to their elution order, as shown in the reaction scheme above. **81** showed a positive ^{1}H-^{13}C correlation between C<u>H</u> at 4.54 ppm and the C_3 at 126.1 ppm. Yields: (**80**) 111 mg, 21%; (**81**) 75 mg, 14%.

80) ^{1}H NMR (400 MHz, DMSO-d_6) δ 10.02 (s, 1H), 8.41 (t, *J* = 1.0 Hz, 1H), 8.32 (s, 1H), 7.91 – 7.81 (m, 2H), 4.67 (tt, *J* = 10.0, 5.0 Hz, 1H), 1.89 (ddt, *J* = 24.4, 11.6, 3.7 Hz, 6H), 1.71 (dt, *J* = 12.7, 3.3 Hz, 1H), 1.50 (dtt, *J* = 16.7, 8.1, 4.4 Hz, 2H), 1.27 (qt, *J* = 12.8, 3.5 Hz, 1H). ^{13}C NMR (101 MHz, DMSO-d_6) δ 192.18, 140.59, 134.95, 130.06, 127.69, 124.09, 123.14, 110.54, 56.92, 32.23, 24.94. R_t 2.30 min (generic method). ESI-MS for $C_{14}H_{16}N_2O$: calculated 228.1, found *m*/*z* 229.0 $[M+H]^+$.

81) ^{1}H NMR (400 MHz, DMSO-d_6) δ 9.97 (s, 1H), 8.76 (d, *J* = 0.9 Hz, 1H), 8.43 (t, *J* = 1.2 Hz, 1H), 7.74 – 7.62 (m, 2H), 4.53 (tt, *J* = 11.4, 3.8 Hz, 1H), 2.18 – 2.09 (m, 2H), 1.97 – 1.81 (m, 4H), 1.78 – 1.65 (m, 1H), 1.46 (qt, *J* = 12.9, 3.5 Hz, 2H), 1.27 (qt, *J* = 12.8, 3.5 Hz, 1H). ^{13}C NMR (101 MHz, DMSO-d_6) δ 192.23, 149.27, 130.48, 130.40, 125.65, 121.89, 120.46, 117.88, 62.06, 33.08, 24.83, 24.77. R_t 2.05 min (generic method). ESI-MS for $C_{14}H_{16}N_2O$: calculated 228.1, found *m*/*z* 228.8 $[M+H]^+$.

Scheme S8: Synthesis of aldehydes 82, 83

The synthesis of the 4-(ethylindazol-5-yl)benzaldehydes **82** and **83** start from the Suzuki coupling reaction between the commercially available 4-iodobenzaldehyde **127** and 1*H*-indazole-5-boronic acid **135** in presence of palladium(0) catalyst to give the corresponding intermediate **136**. The alkylation of **136** with ethyl bromide **132** under basic conditions afforded the aldehydes **82** and **83**.

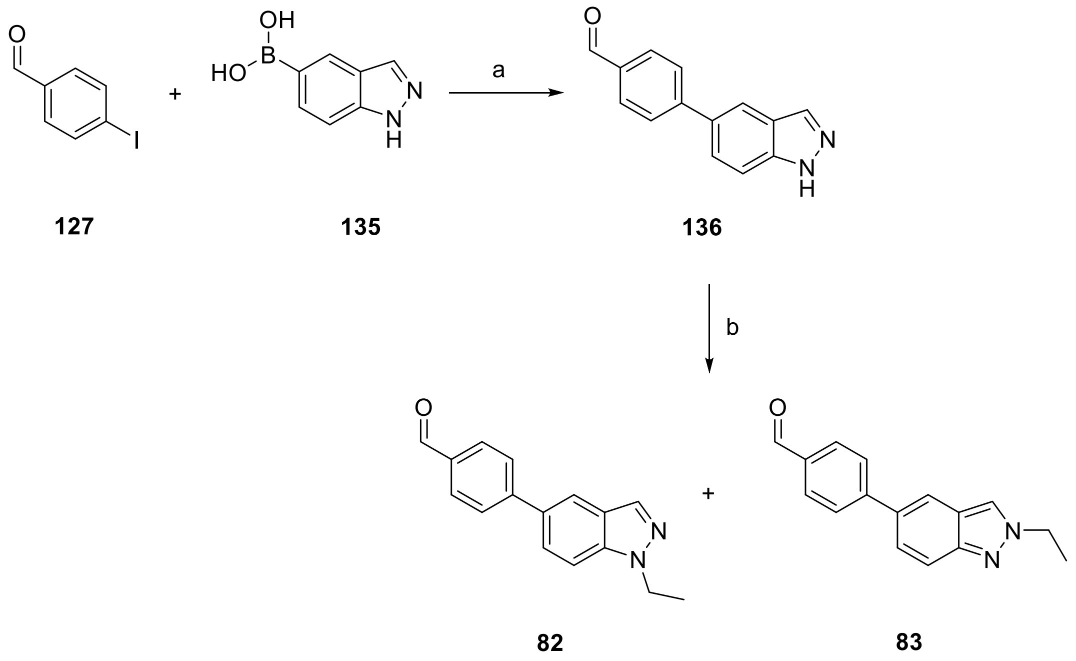

Reagents and conditions: (a) i. Anhydrous sodium carbonate (3.0 equiv); ii. DMF, H_2O, rt, 15 minutes; iii. Tetrakis(triphenylphosphine)palladium(0) (0.15 equiv), 110 °C, 22 h; (b) Potassium carbonate (2.0 equiv), anhydrous DMF, ethyl bromide **132** (2.5 equiv), rt, 40 h.

4-(1*H*-Indazol-5-yl)benzaldehyde (**136**). In a Schlenk tube 4-iodobenzaldehyde **127** (300 mg, 1.3 mmol), 1*H*-indazole-5-boronic acid **135** (316 mg, 1.9 mmol) and anhydrous sodium carbonate (413 mg, 3.9 mmol) were added. The tube was deoxygenated with three cycles vacuum/Ar, then DMF (2 mL) and H_2O (400 µL) were added. The solution was stirred at rt, under Ar flux, for 15 minutes. Tetrakis(triphenylphosphine)palladium(0) (220 mg, 0.19 mmol) was thus added, the mixture was heated to 110 °C and stirred for 22 hours. The crude was diluted with DCM and washed with water (3 x 50 mL), the solvent thus removed under reduced pressure. Purified by normal phase flash column chromatography (SiO_2 gold 24 g, 0-5% EtOH/ DCM). Yield 205 mg, 71%. ^{1}H NMR (400 MHz, DMSO-d_6) δ 13.20 (s, 1H), 10.05 (s, 1H), 8.22 – 8.12 (m, 2H), 7.98 (q, *J* = 8.5 Hz, 4H), 7.84 – 7.61 (m, 2H). R_t 1.77 min (generic method). ESI-MS for $C_{14}H_{10}N_2O$: calculated 222.1, found *m*/*z* 223.0 $[M+H]^+$, 221.2 $[M-H]^-$

4-(Ethylindazol-5-yl)benzaldehydes (**82, 83**). In a screw capped pressure tube 4-(1*H*-indazol-5-yl)benzaldehyde **136** (204 mg, 0.92 mmol) was dissolved with 1.0 mL of anhydrous DMF then potassium carbonate (254 mg, 1.8 mmol) was added and the mixture kept under stirring for 15 minutes at rt. Ethyl bromide **132** (251 mg, d = 1.46 g/mL, 172 µL, 2.3 mmol) was then added. The reaction was thus stirred at rt for 40 hours. The crude mixture was diluted with DCM and washed with water (3x50 mL). The organic layer was dried through a phase separator and the solvent

removed under reduced pressure. Purified by normal phase flash column chromatography (SiO_2 gold 24 g, 0- 5% EtOH/ DMC). Mono- and bi-dimensional ^{1}H- and ^{13}C-NMR analyses confirmed the structure of the title compounds named **82** (4-(1-ethyl-1H-indazol-5-yl)benzaldehyde) and **83** **(**4-(2-ethyl-2H-indazol-5-yl)benzaldehyde) according to their elution order, as shown in the reaction scheme above. Yields: (**82**) 125 mg, 54%; (**83**) 82 mg, 39%.

82) ^{1}H NMR (400 MHz, DMSO-d_6) δ 10.05 (s, 1H), 8.17 (t, J = 1.3 Hz, 1H), 8.15 (s, 1H), 8.02 – 7.94 (m, 4H), 7.80 (d, J = 1.3 Hz, 2H), 4.48 (q, J = 7.2 Hz, 2H), 1.42 (t, J = 7.2 Hz, 3H). ^{13}C NMR (101 MHz, DMSO-d_6) δ 192.65, 146.47, 138.64, 134.57, 133.34, 131.34, 130.17, 127.36, 125.54, 124.24, 119.64, 110.32, 43.20, 14.93. R_t 2.19 min (generic method). ESI-MS for $C_{16}H_{14}N_2O$: calculated 250.1, found m/z 251.1 $[M+H]^+$.

83) ^{1}H NMR (400 MHz, DMSO-d_6) δ 10.05 (s, 1H), 8.49 (d, J = 0.9 Hz, 1H), 8.13 (dd, J = 1.8, 0.9 Hz, 1H), 8.02 – 7.91 (m, 4H), 7.78 – 7.60 (m, 2H), 4.49 (q, J = 7.3 Hz, 2H), 1.53 (t, J = 7.3 Hz, 3H). ^{13}C NMR (101 MHz, DMSO-d_6) δ 192.63, 147.68, 146.74, 134.52, 131.49, 130.14, 127.19, 124.97, 124.34, 121.86, 119.45, 117.72, 47.88, 15.72. R_t 1.97 min (generic method). ESI-MS for $C_{16}H_{14}N_2O$: calculated 250.1, found m/z 251.1 $[M+H]^+$.

Scheme S9: Synthesis of aldehyde 84

4-(1-Propyl-1H-pyrazol-4-yl)benzaldehyde **84** was achieved taking advantage of Suzuki coupling reaction between the commercially available 4-iodobenzaldehyde **127** and (1-propyl-1*H*-pyrazol-4-yl)boronic acid **137** in presence of palladium(0) catalyst.

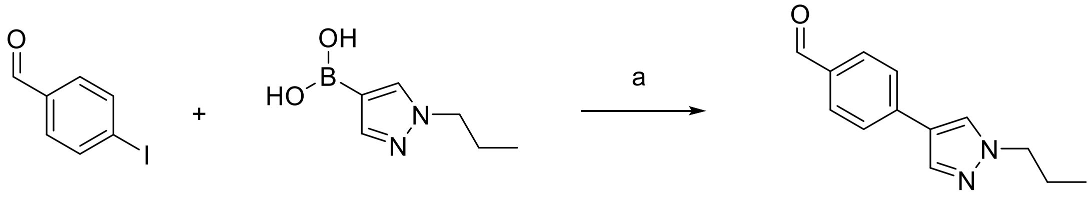

127 **137** **84**

Reagents and conditions: (a) i. Anhydrous sodium carbonate (3.0 equiv); ii. DMF, H_2O, rt, 15 minutes; iii. Tetrakis(triphenylphosphine)palladium(0) (0.15 equiv), 110 °C, 18 h.

4-(1-Propyl-1H-pyrazol-4-yl)benzaldehyde (**84**). In a Schlenk tube 4-iodobenzaldehyde **127** (326 mg, 1.4 mmol), (1-propyl-1*H*-pyrazol-4-yl)boronic acid **137** (323 mg, 2.1 mmol) and anhydrous sodium carbonate (445 mg, 4.2 mmol) were added. The tube was deoxygenated with three cycles

vacuum/Ar, then anhydrous DMF (1.1 mL) and H_2O (300 µL) were added. The solution was stirred at rt, under Ar flux, for 15 minutes. Tetrakis(triphenylphosphine)palladium(0) (243 mg, 0.2 mmol) was thus added, the mixture was heated to 110 °C and stirred for 18 hours. The reaction mixture was diluted with DCM and washed with water (3x50 mL), the solvent was thus removed under reduced pressure. Purified by normal phase flash column chromatography (SiO_2 gold 24 g, 0-30% EtOAc/cyclohexane). Yield 238 mg, 97%. ^{1}H NMR (400 MHz, DMSO-d_6) δ 9.95 (s, 1H), 8.37 (s, 1H), 8.03 (s, 1H), 7.94 – 7.75 (m, 4H), 4.10 (t, J = 6.9 Hz, 2H), 1.83 (h, J = 7.2 Hz, 2H), 0.85 (t, J = 7.4 Hz, 3H). ^{13}C NMR (101 MHz, DMSO-d_6) δ 192.17, 138.95, 136.76, 133.78, 130.30, 128.33, 125.10, 120.48, 53.14, 23.05, 10.90. R_t 1.86 min (generic method). ESI-MS for $C_{13}H_{14}N_2O$: calculated 214.1, found m/z 215.5 $[M+H]^+$.

^{1}H-NMR spectrum, ^{13}C-NMR spectrum and HPLC-MS analysis of final compounds 4d-10d, 14d-15d, 18d-57d

^{1}H-NMR spectrum (400 MHz, DMSO-d_6) of **4d**

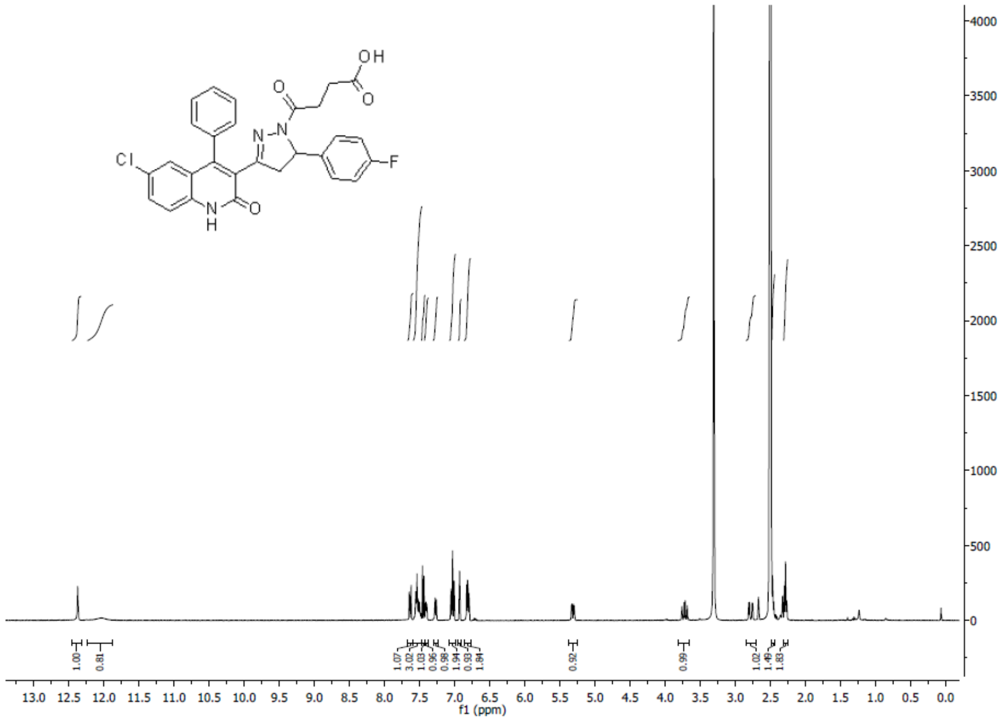

^{13}C-NMR spectrum (101 MHz, DMSO-d_6) of **4d**

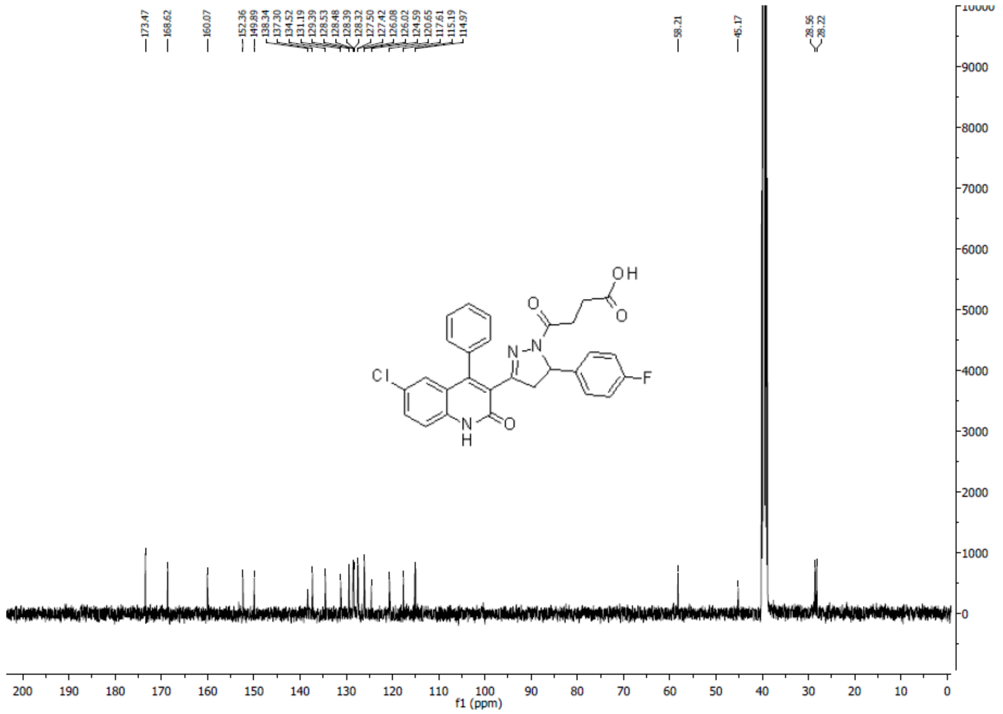

HPLC-MS analysis of **4d**

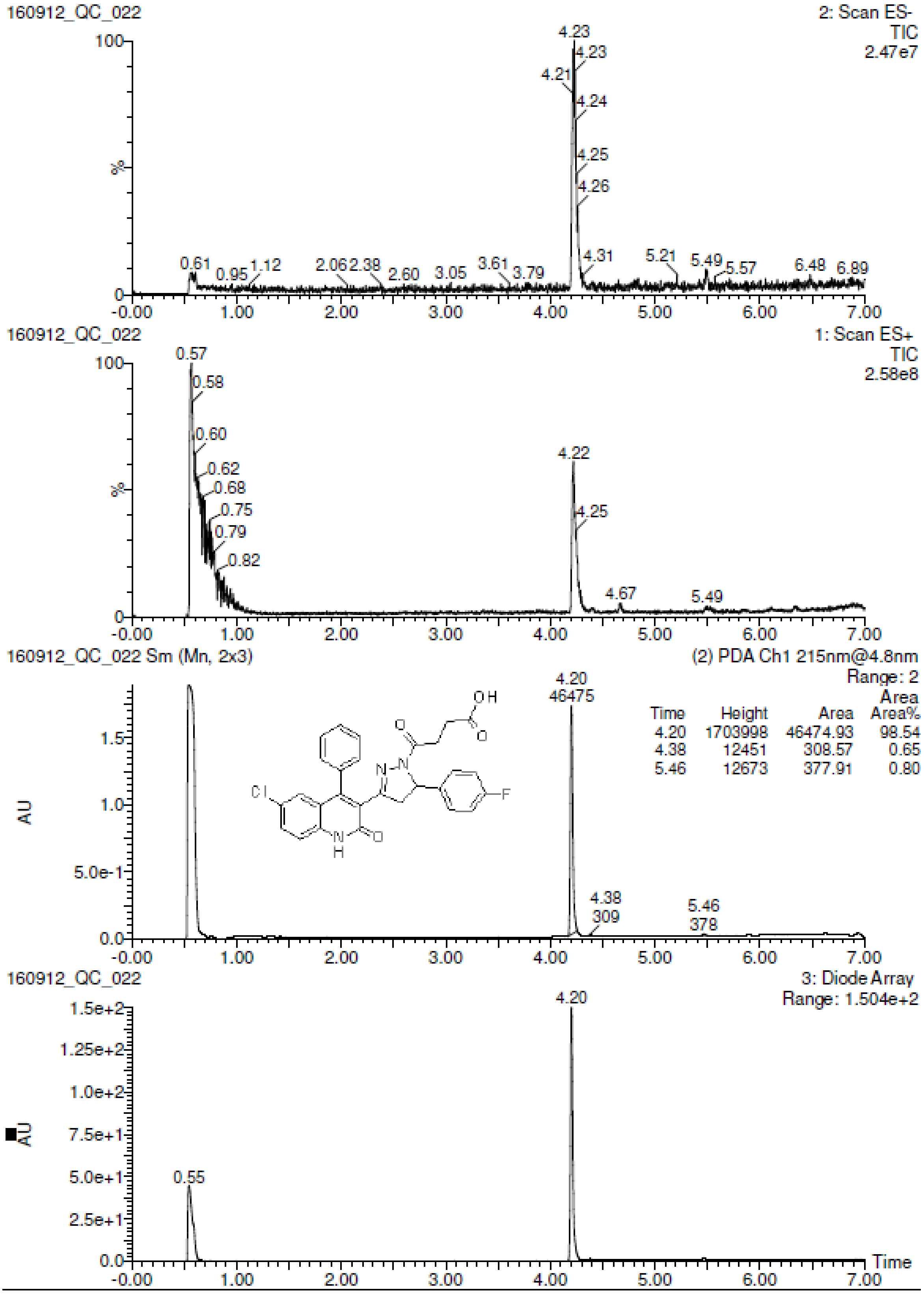

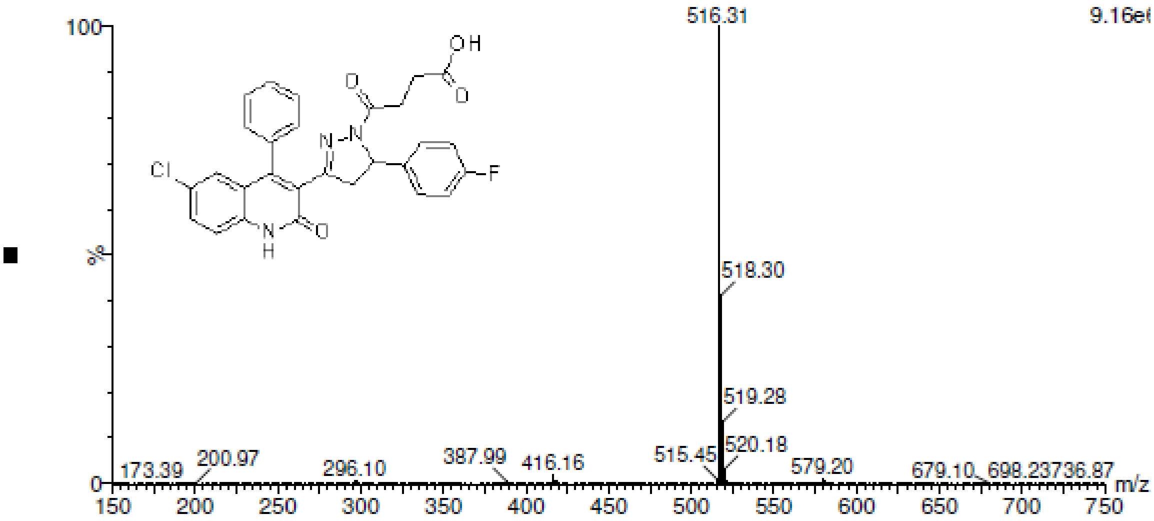

516.31
9.16e6
100
%
0
518.30
519.28
520.18
173.39
200.97
296.10
387.99
416.16
515.45
579.20
679.10
698.23
736.87
m/z
150 200 250 300 350 400 450 500 550 600 650 700 750
OH
O
O
N
N
Cl
N
H
O
F

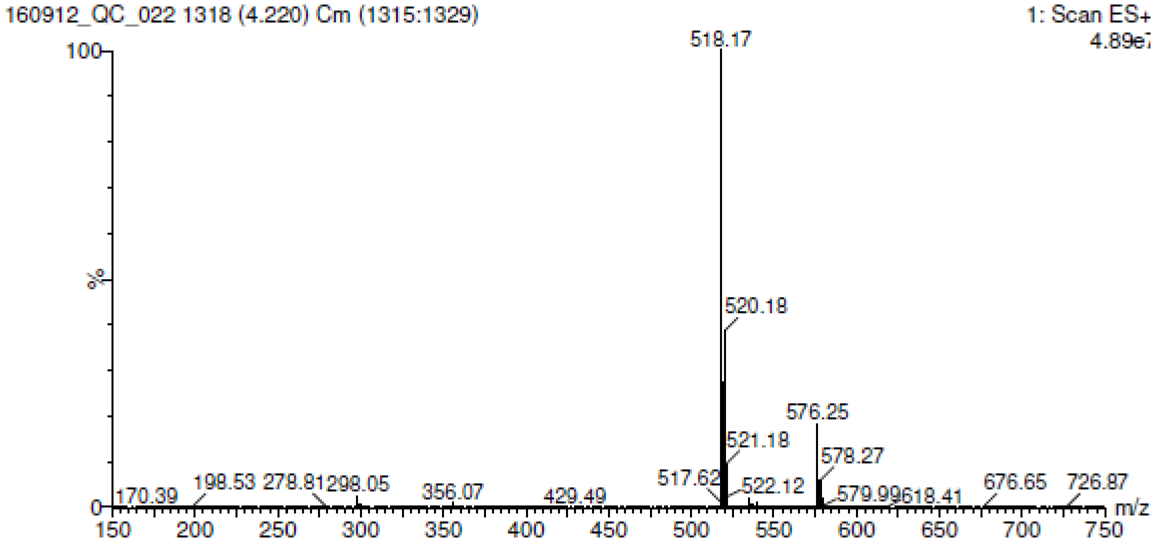

160912_QC_022 1318 (4.220) Cm (1315:1329)
1: Scan ES+
518.17
4.89e7
100
%
0
520.18
576.25
521.18
578.27
170.39
198.53
278.81
298.05
356.07
429.49
517.62
522.12
579.99
618.41
676.65
726.87
m/z
150 200 250 300 350 400 450 500 550 600 650 700 750

^{1}H-NMR spectrum (400 MHz, DMSO-d_6) of **5d**

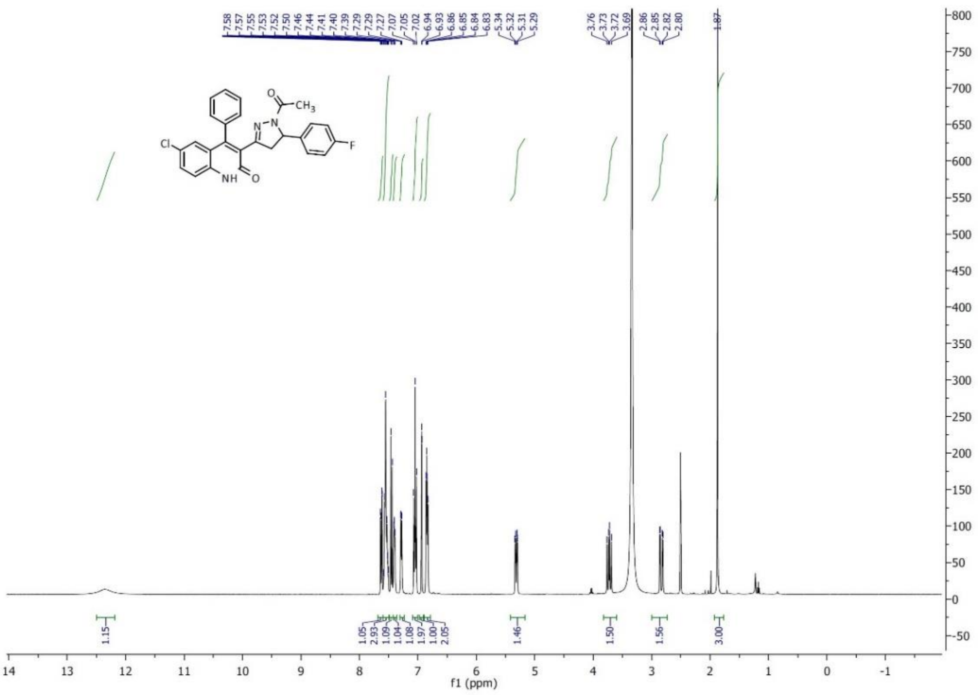

^{13}C-NMR spectrum (101 MHz, DMSO-d_6) of **5d**

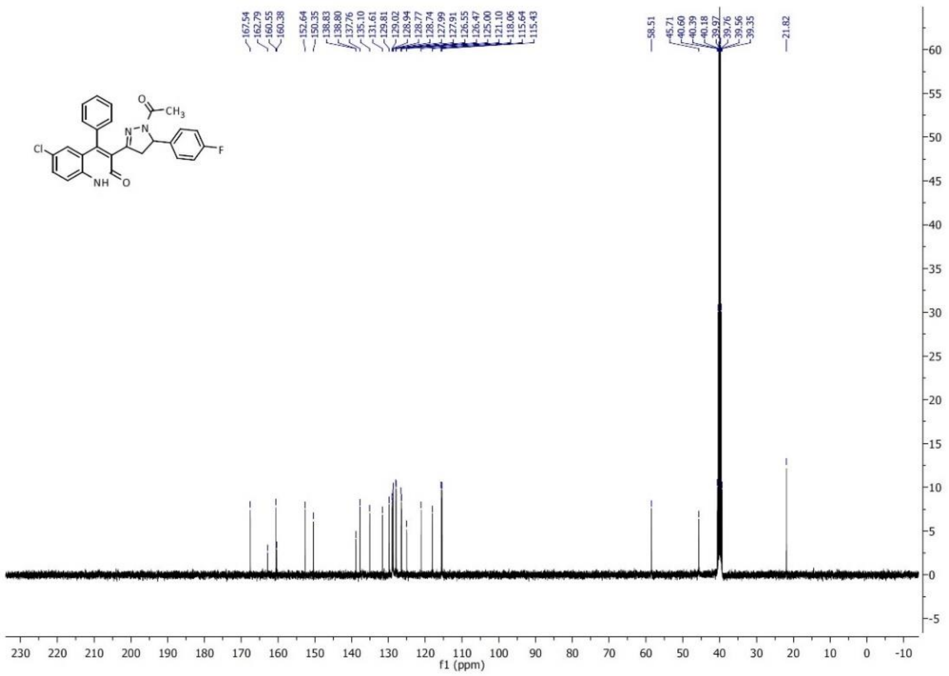

HPLC-MS analysis of **5d**

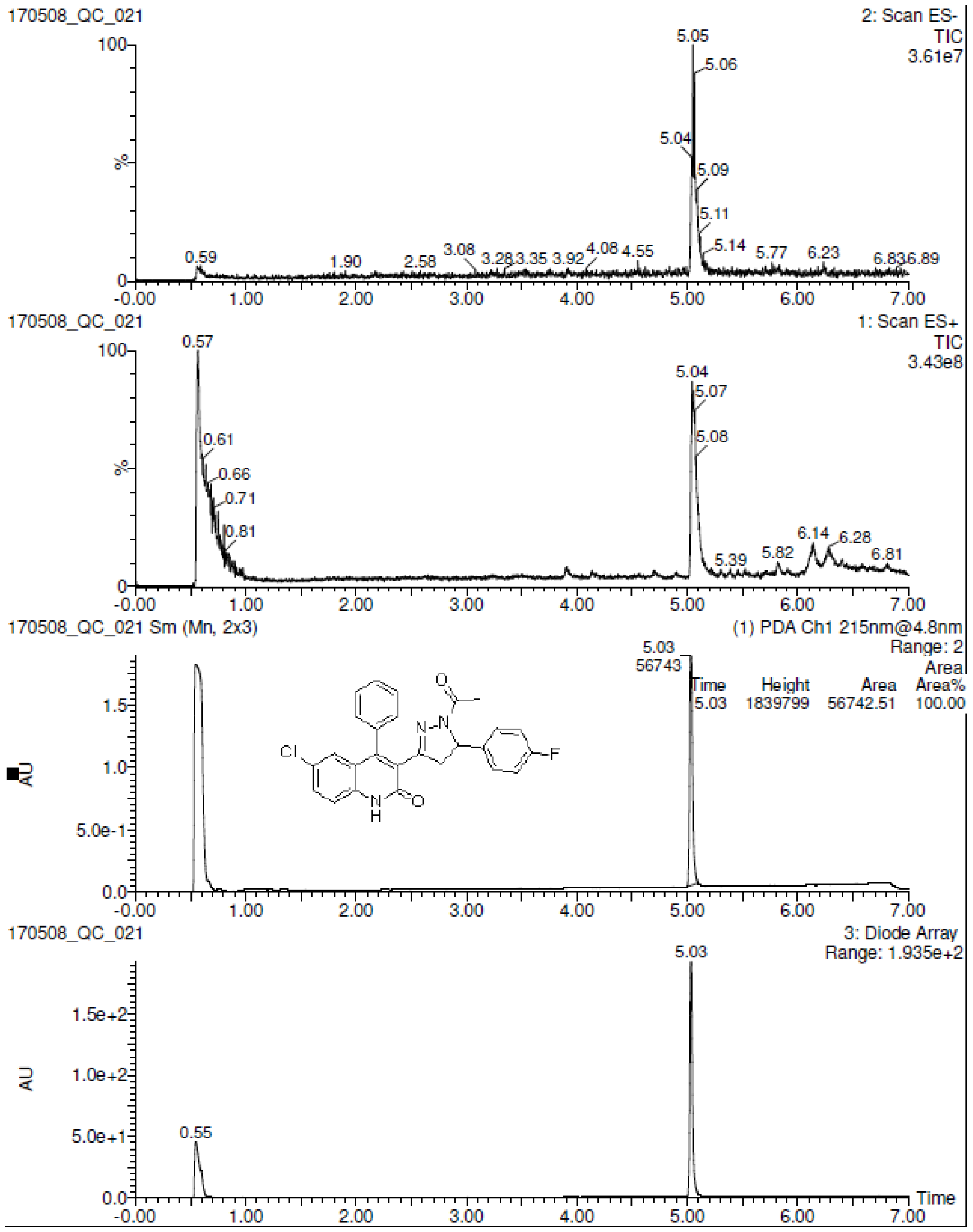

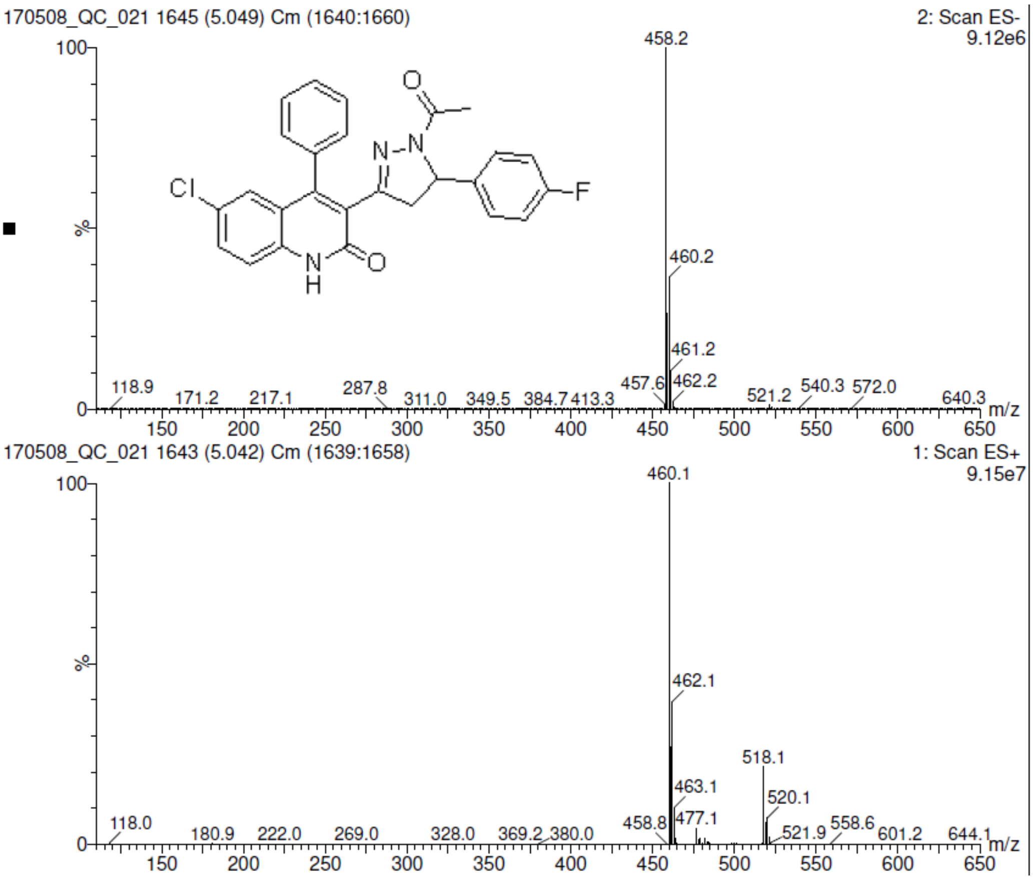

170508_QC_021 1645 (5.049) Cm (1640:1660)
2: Scan ES-
9.12e6
458.2
460.2
461.2
462.2
457.6
118.9
171.2
217.1
287.8
311.0
349.5
384.7
413.3
521.2
540.3
572.0
640.3
m/z
%
100
0
150
200
250
300
350
400
450
500
550
600
650
Cl
N
H
O
N
N
O
F
170508_QC_021 1643 (5.042) Cm (1639:1658)
1: Scan ES+
9.15e7
460.1
462.1
518.1
463.1
520.1
477.1
458.8
521.9
558.6
601.2
644.1
118.0
180.9
222.0
269.0
328.0
369.2
380.0

^{1}H-NMR spectrum (400 MHz, DMSO-d_6) of **6d**

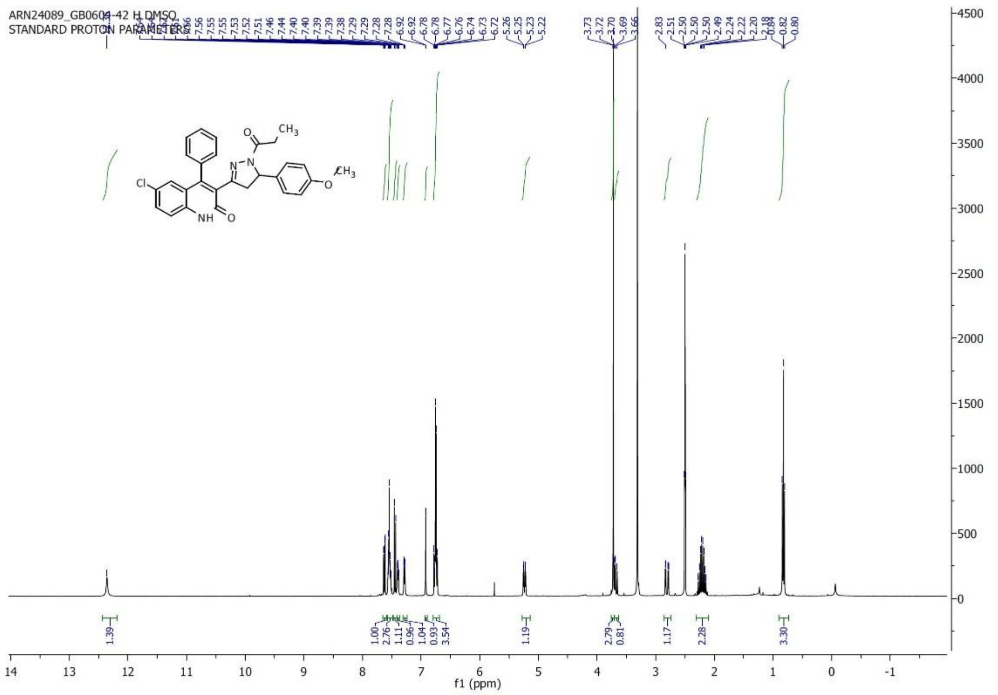

^{13}C-NMR spectrum (101 MHz, DMSO-d_6) of **6d**

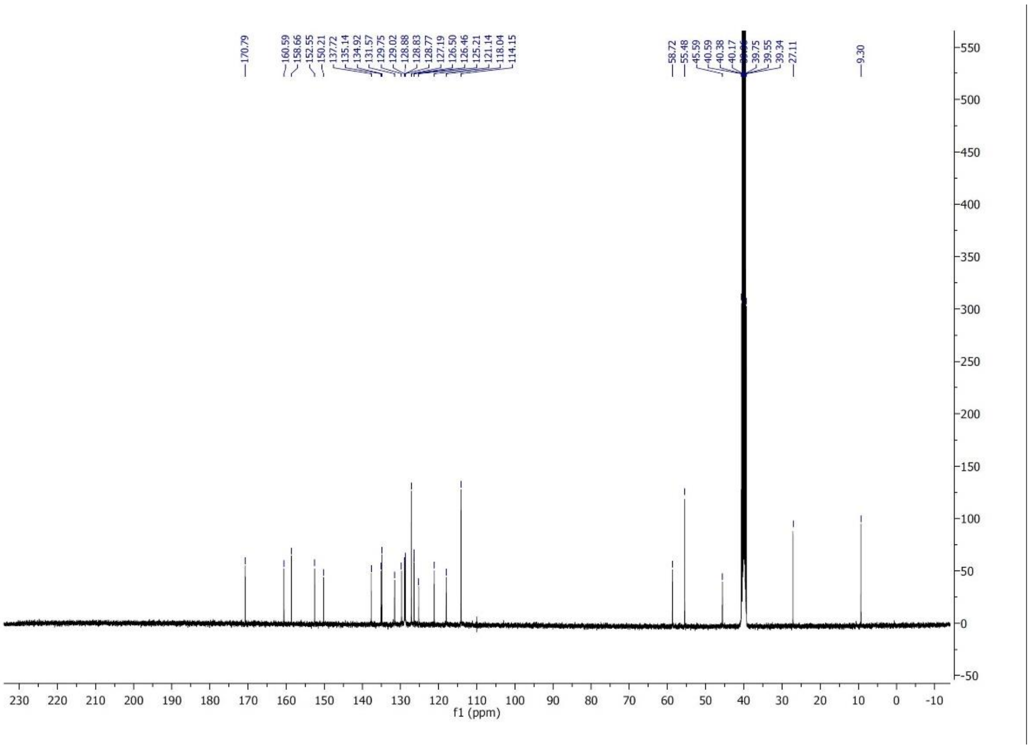

HPLC-MS analysis of **6d**

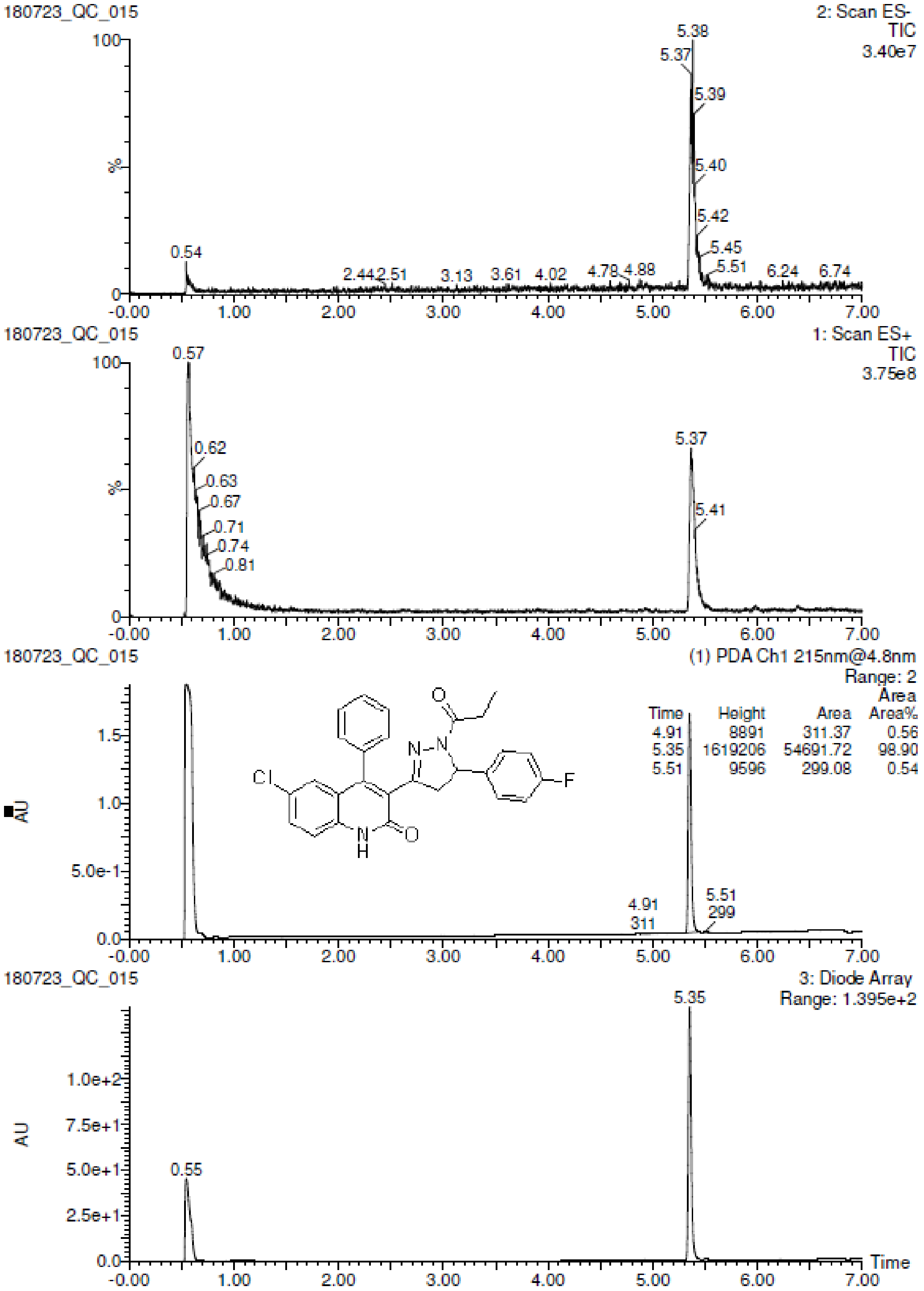

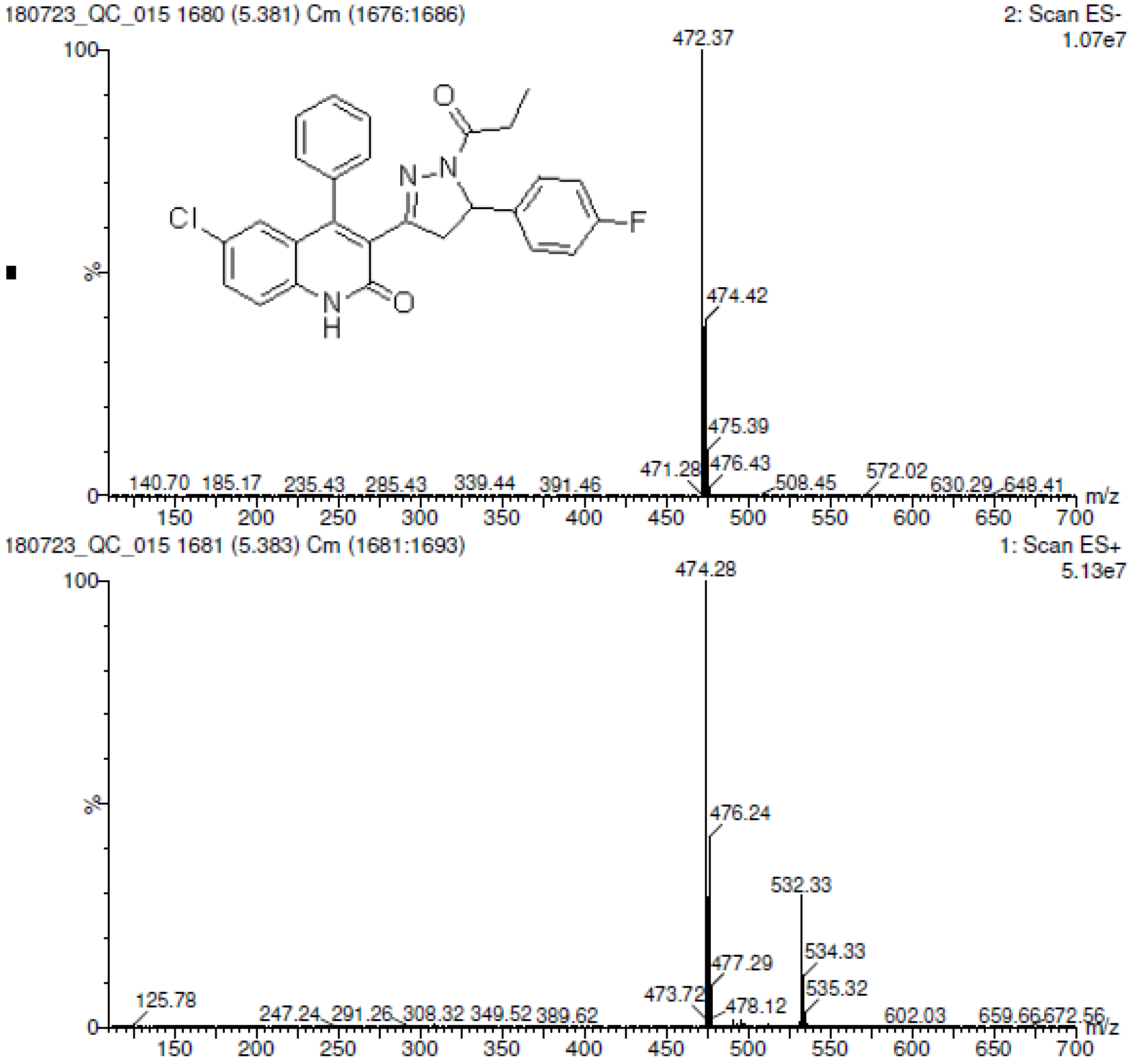

180723_QC_015 1680 (5.381) Cm (1676:1686)
2: Scan ES-
1.07e7
472.37
474.42
475.39
471.28
476.43
140.70 185.17 235.43 285.43 339.44 391.46
508.45
572.02
630.29 648.41
m/z
150 200 250 300 350 400 450 500 550 600 650 700
100
%
0
Cl
N
H
O
N
N
O
F
180723_QC_015 1681 (5.383) Cm (1681:1693)
1: Scan ES+
5.13e7
474.28
476.24
532.33
534.33
477.29
535.32
473.72
478.12
125.78
247.24 291.26 308.32 349.52 389.62
602.03
659.66 672.56
m/z

^{1}H-NMR spectrum (400 MHz, DMSO-d_6) of **7d**

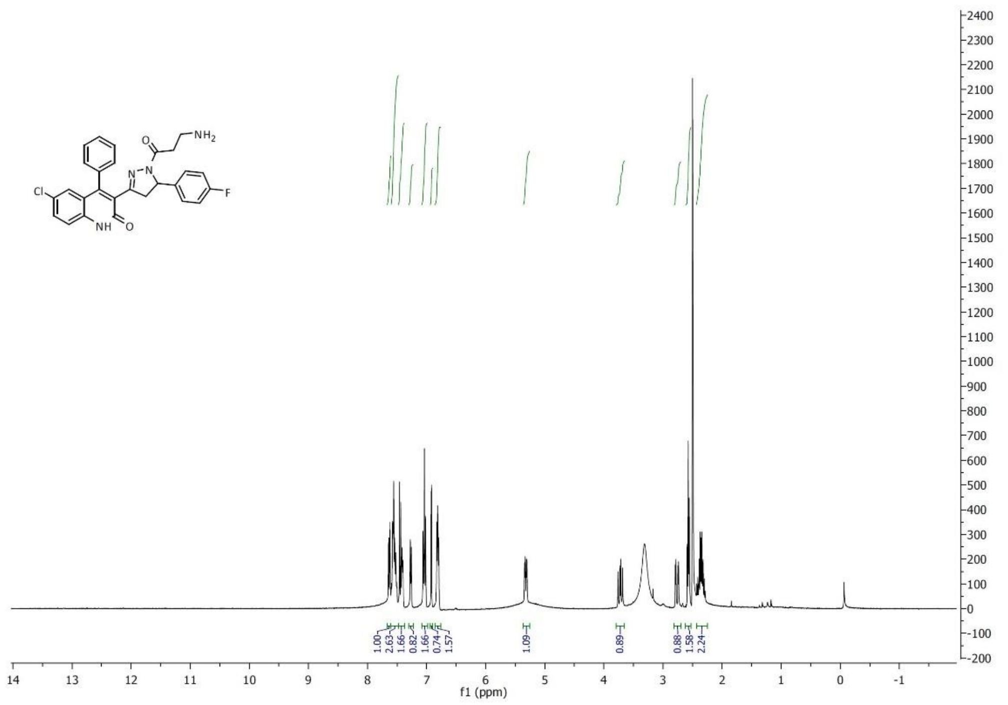

^{13}C-NMR spectrum (101 MHz, DMSO-d_6) of **7d**

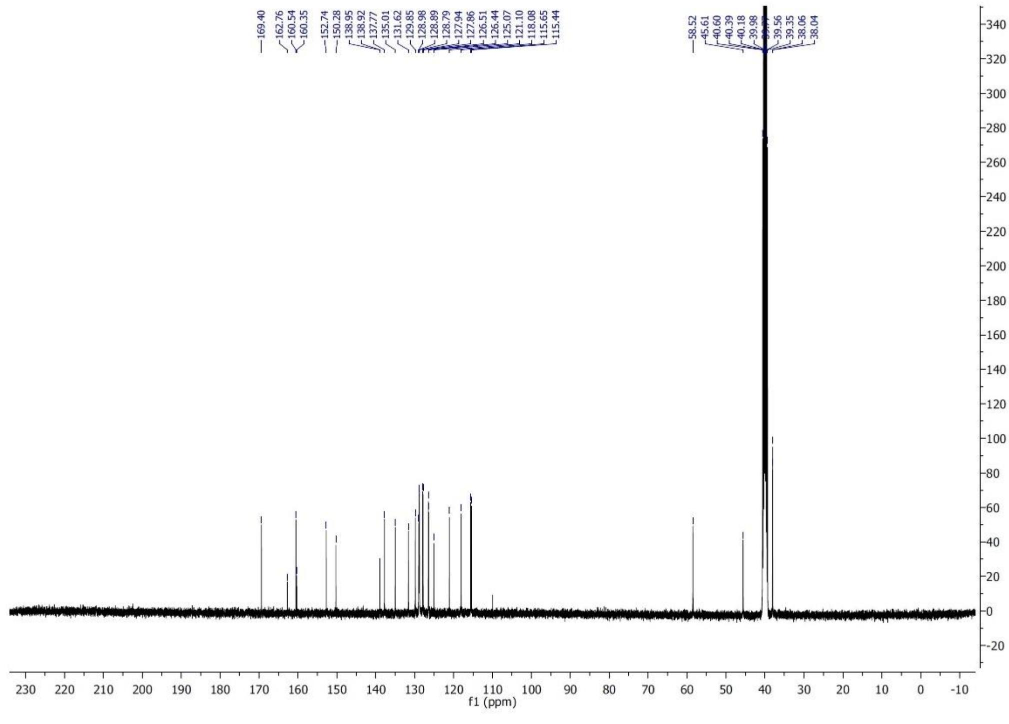

HPLC-MS analysis of **7d**

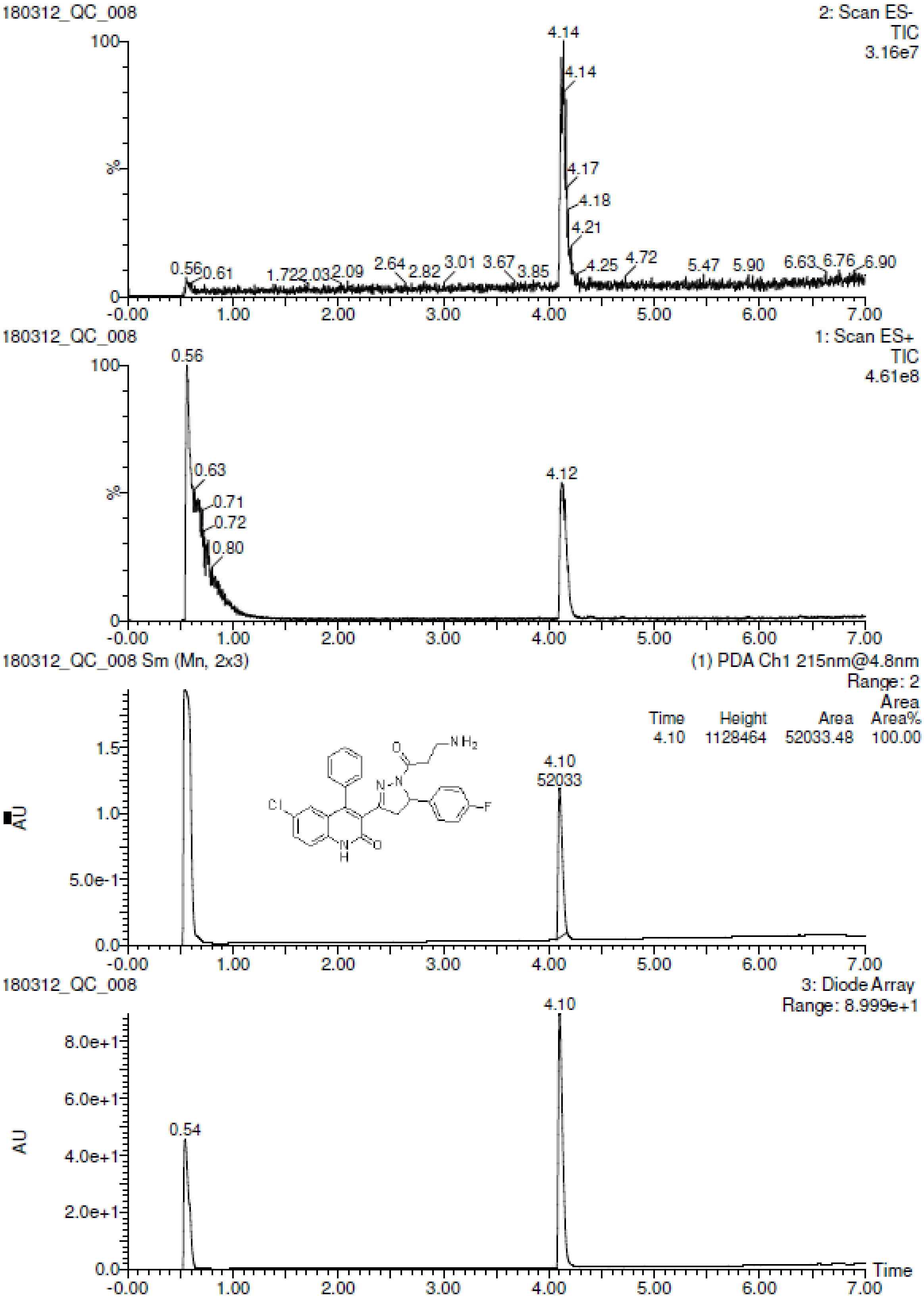

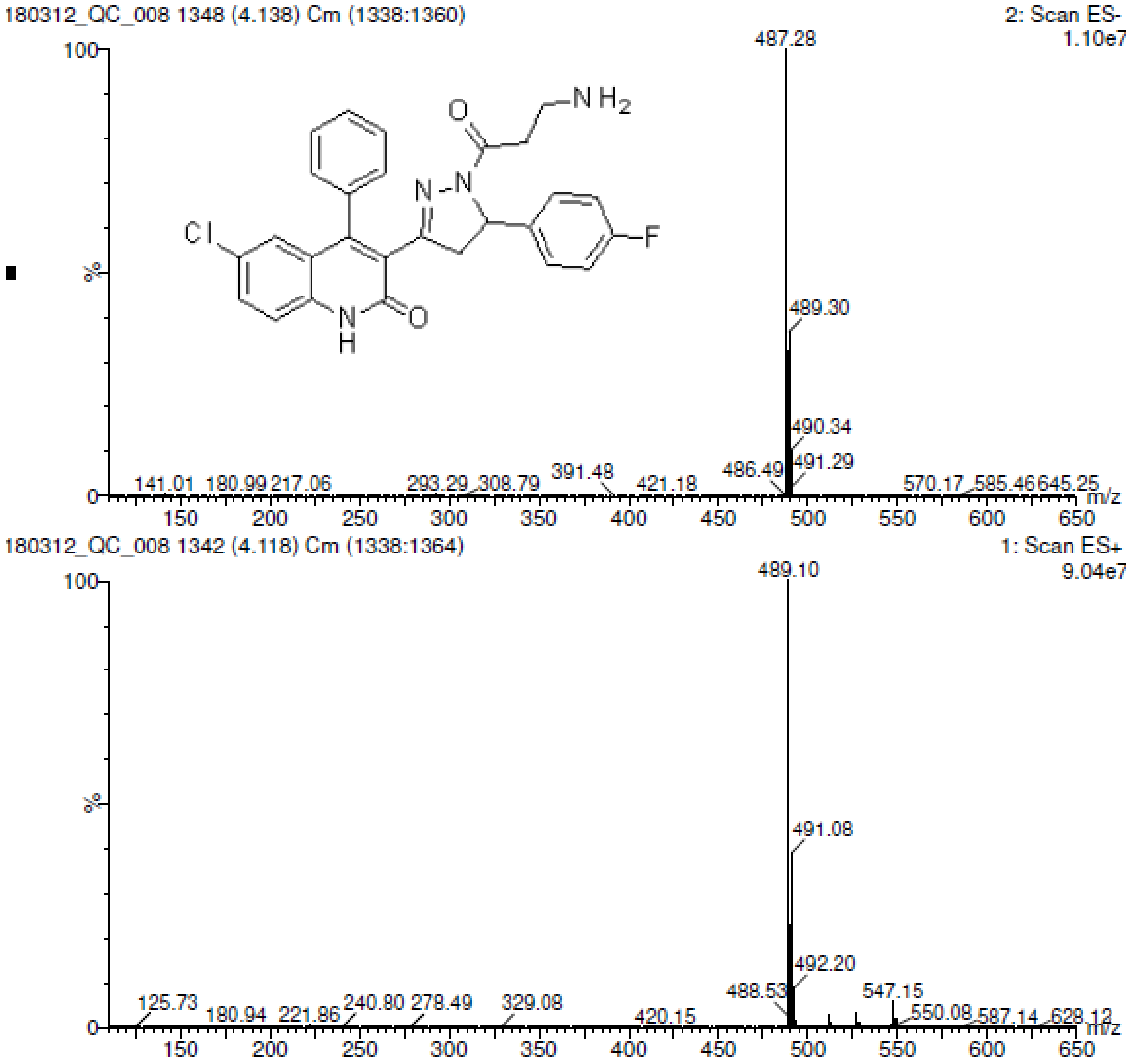

180312_QC_008 1348 (4.138) Cm (1338:1360)
2: Scan ES-
1.10e7
487.28
489.30
490.34
491.29
486.49
141.01 180.99 217.06
293.29 308.79
391.48
421.18
570.17 585.46 645.25
Cl
N
H
O
N
N
O
NH2
F
100
%
0
150 200 250 300 350 400 450 500 550 600 650
m/z
180312_QC_008 1342 (4.118) Cm (1338:1364)
1: Scan ES+
9.04e7
489.10
491.08
492.20
488.53
547.15
550.08 587.14 628.12
125.73 180.94 221.86 240.80 278.49 329.08
420.15

^{1}H-NMR spectrum (400 MHz, DMSO-d_6) of **8d**

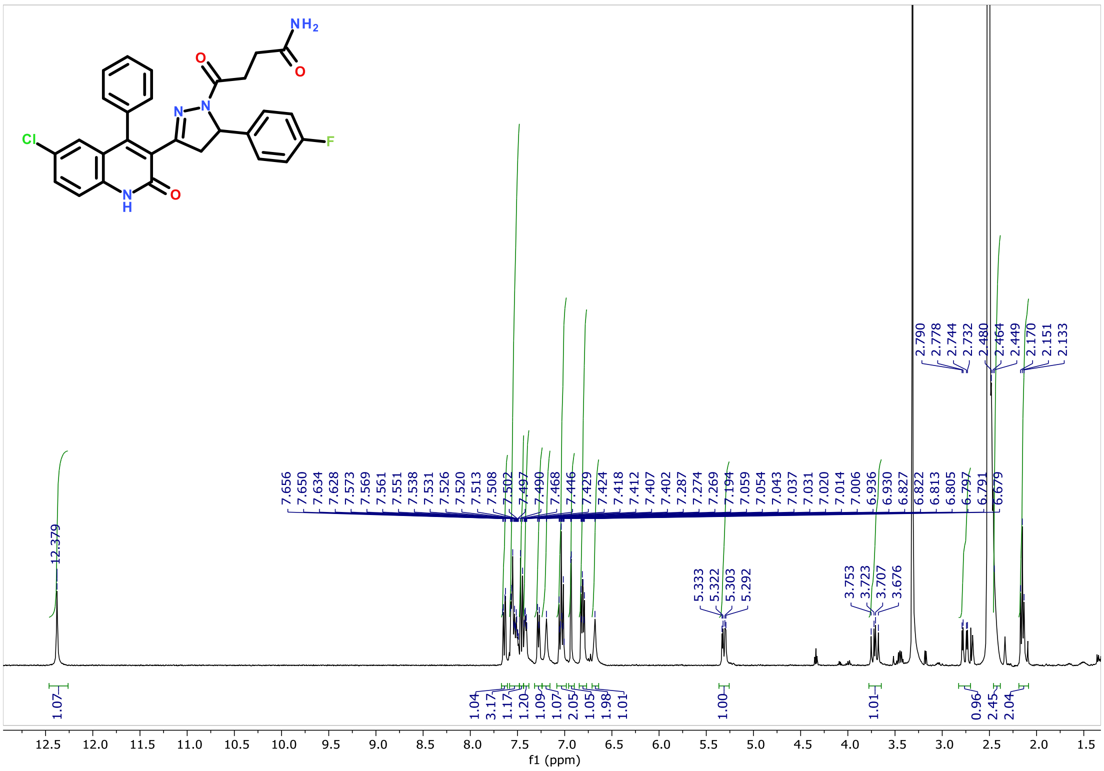

^{13}C-NMR spectrum (101 MHz, DMSO-d_6) of **8d**

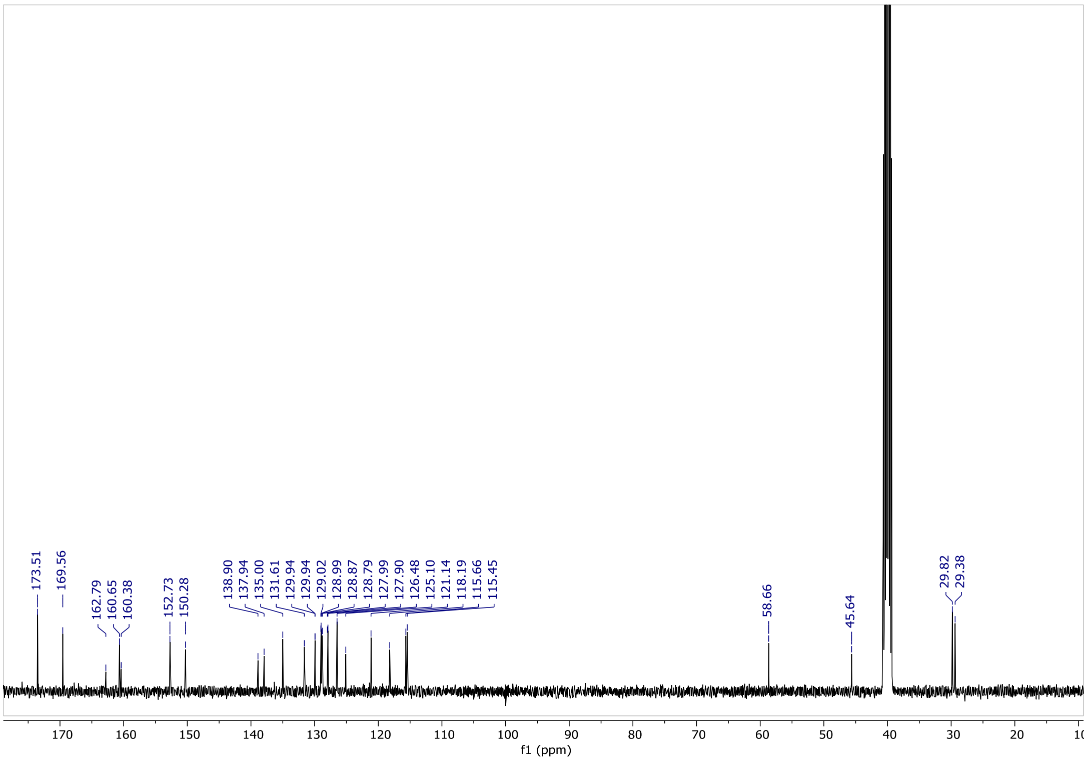

HPLC-MS analysis of **8d**

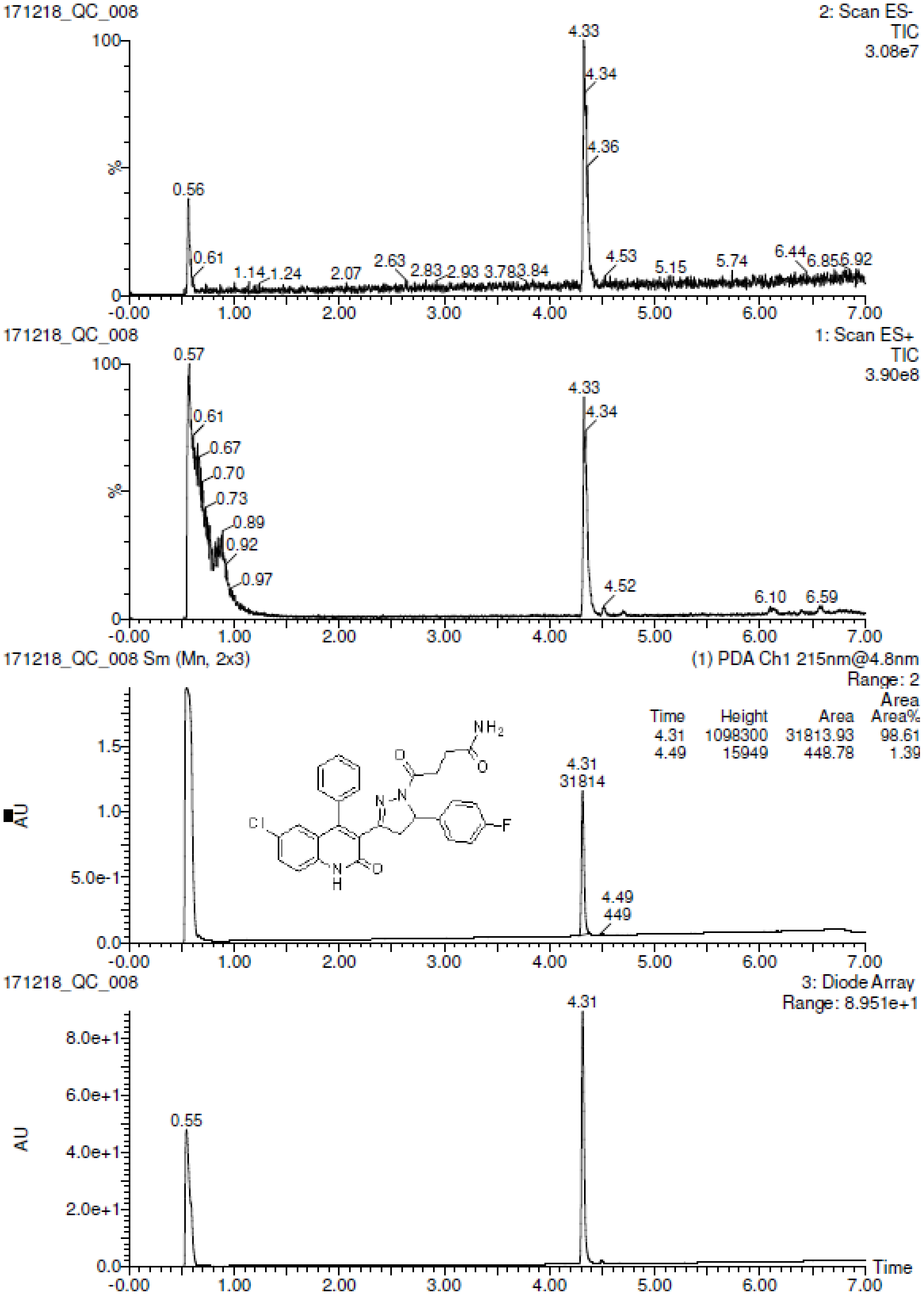

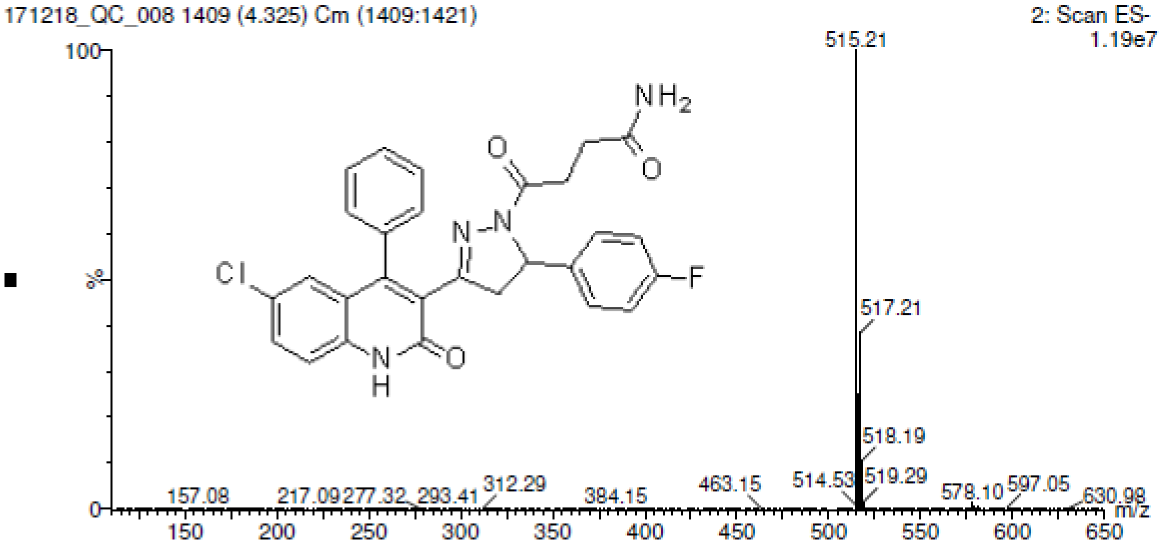
171218_QC_008 1409 (4.325) Cm (1409:1421)
2: Scan ES-
1.19e7
515.21
517.21
518.19
519.29
514.53
463.15
384.15
312.29
157.08
217.09
277.32
293.41
578.10
597.05
630.98
m/z
Cl
NH2
O
N
H
F

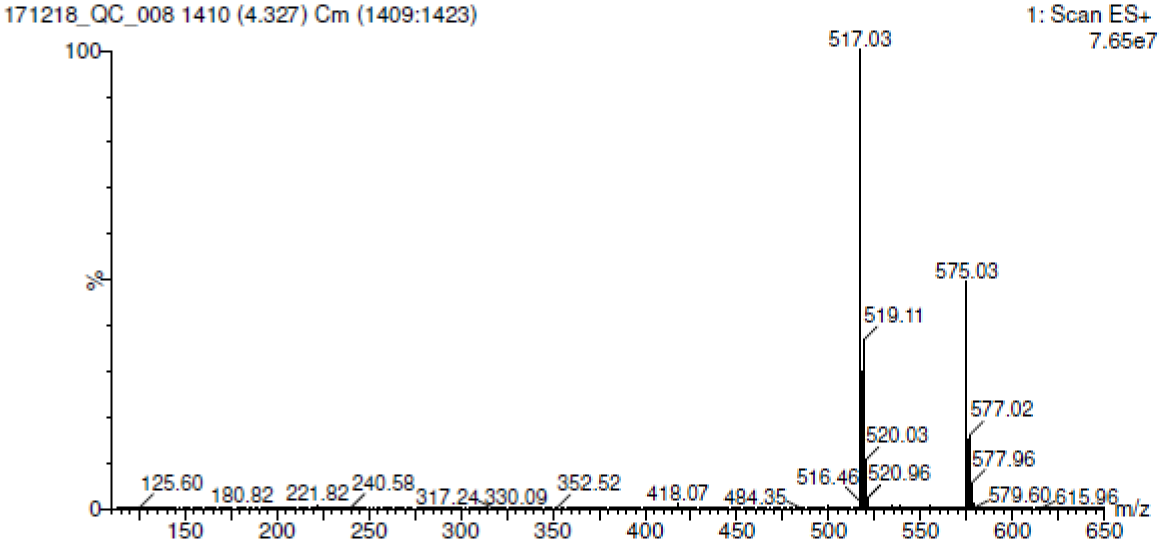
171218_QC_008 1410 (4.327) Cm (1409:1423)
1: Scan ES+
7.65e7
517.03
575.03
519.11
577.02
520.03
577.96
516.46
520.96
125.60
180.82
221.82
240.58
317.24
330.09
352.52
418.07
484.35
579.60
615.96
m/z

^{1}H-NMR spectrum (400 MHz, DMSO-d_6) of **9d**

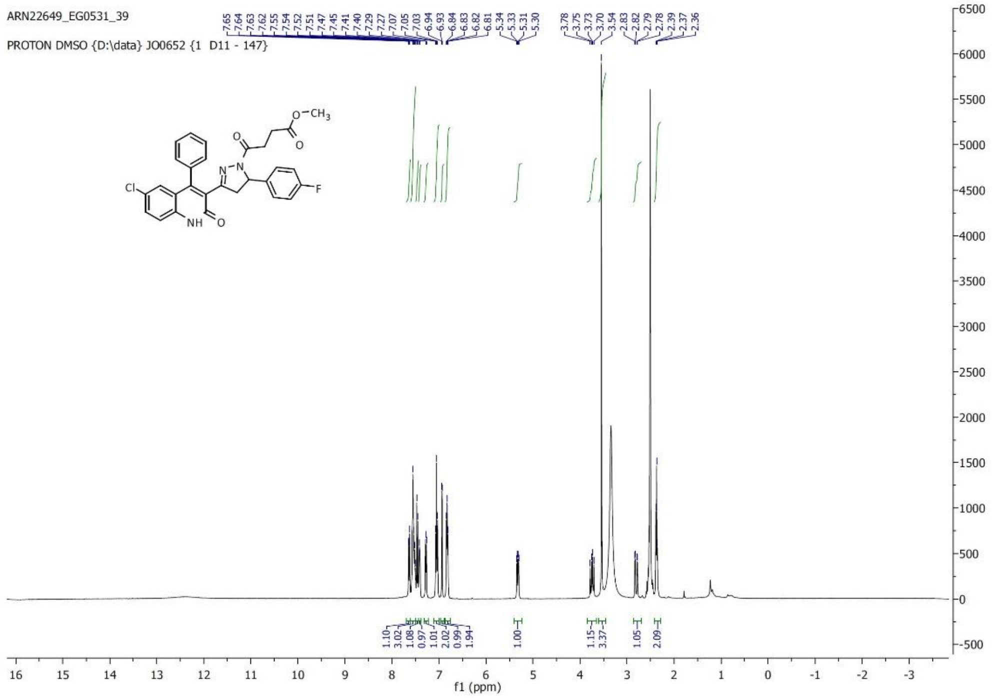

^{13}C-NMR spectrum (101 MHz, DMSO-d_6) of **9d**

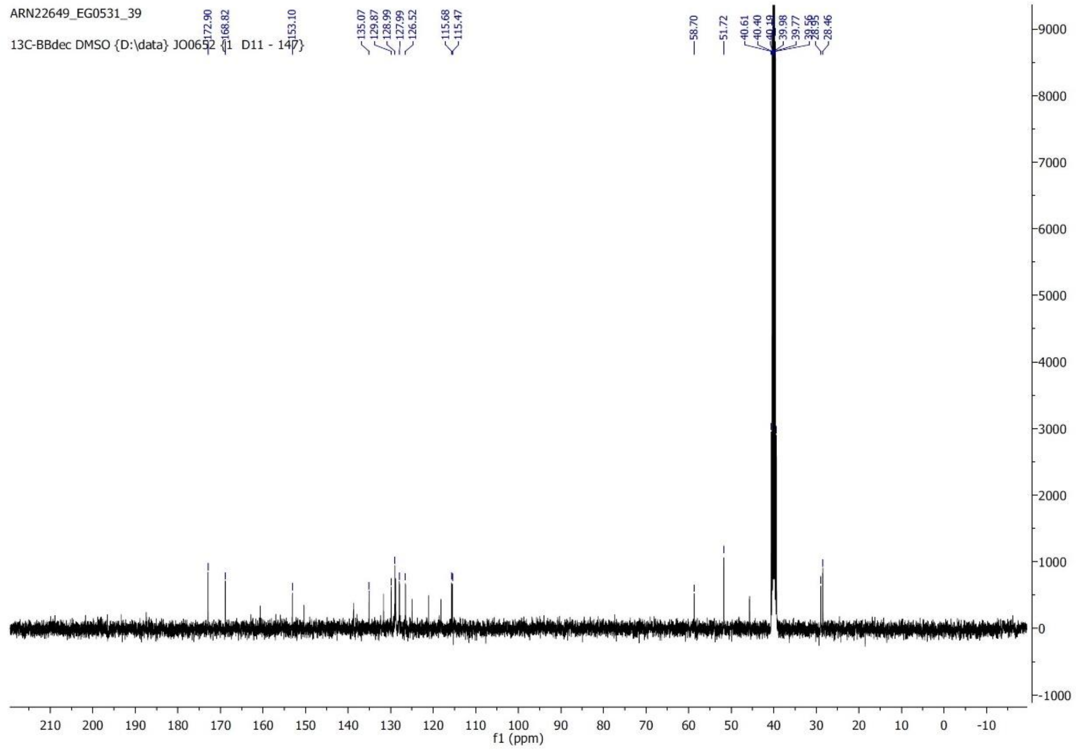

HPLC-MS analysis of **9d**

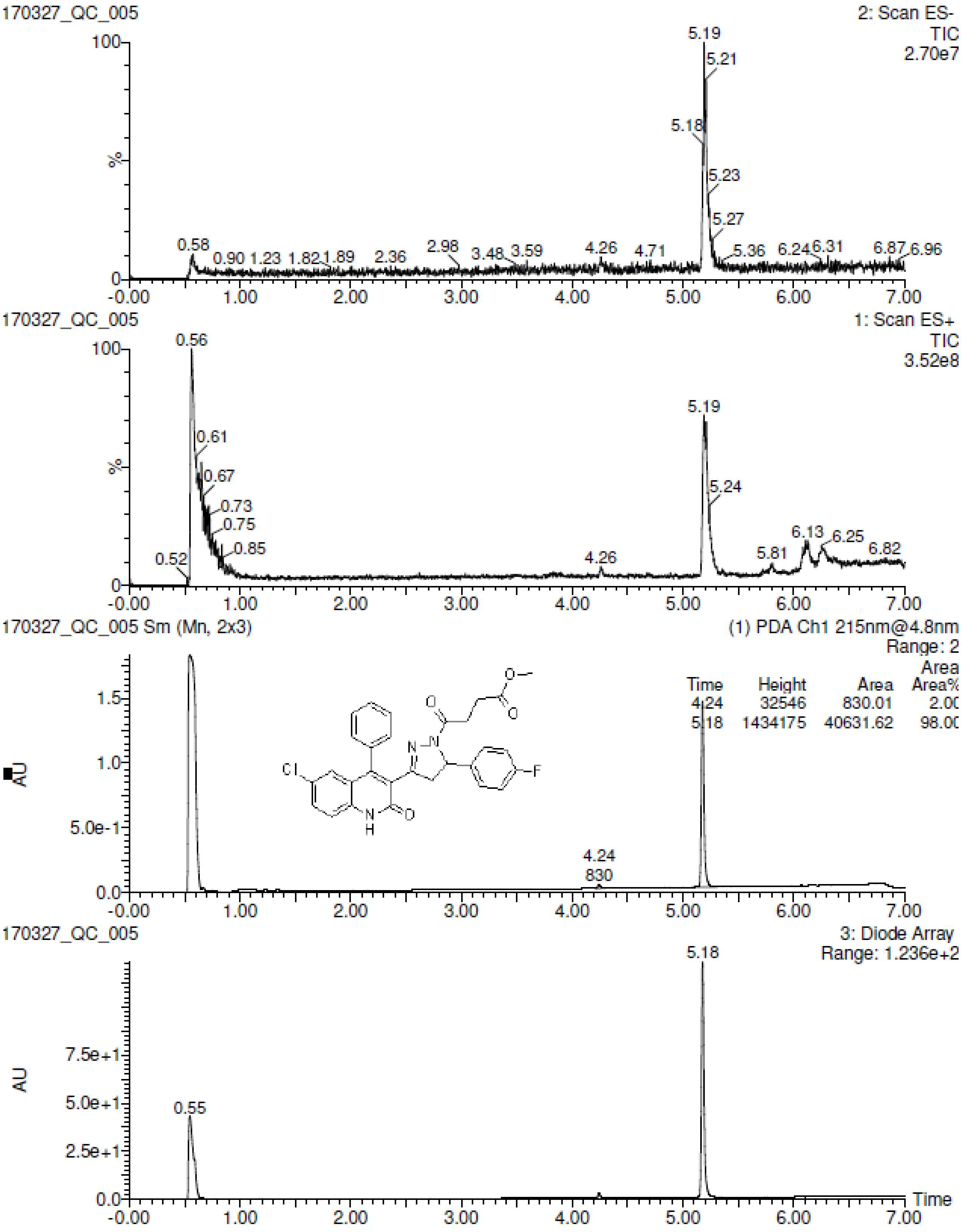

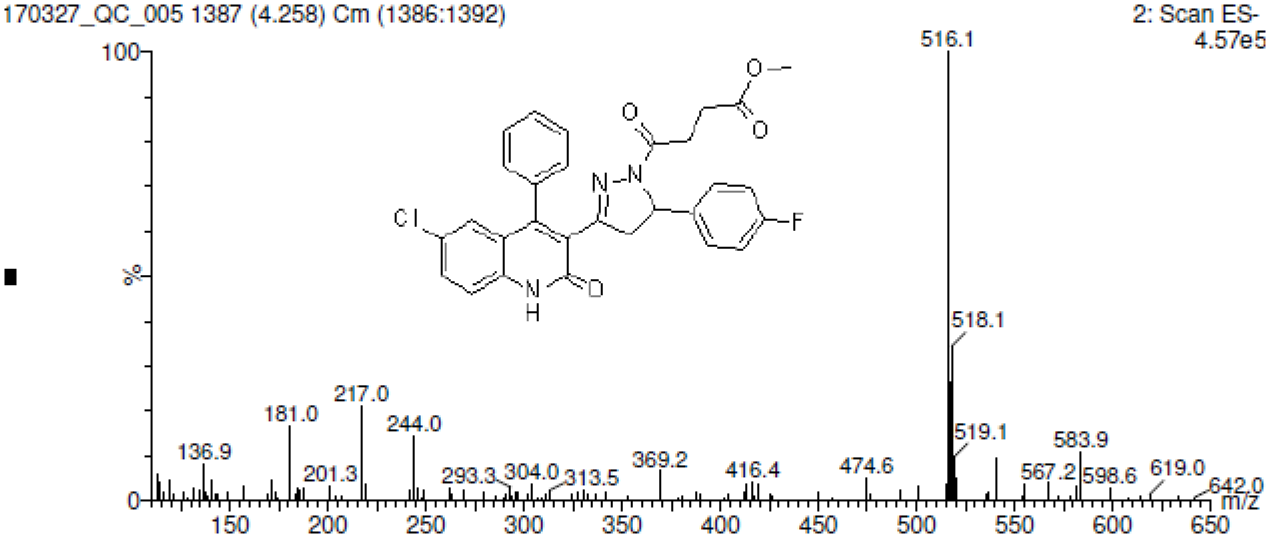
170327_QC_005 1387 (4.258) Cm (1386:1392)
2: Scan ES-
4.57e5
516.1
518.1
519.1
583.9
217.0
181.0
244.0
136.9
201.3
293.3
304.0
313.5
369.2
416.4
474.6
567.2
598.6
619.0
642.0
m/z
%
Cl
N
H
O
N
N
O
O
O
F

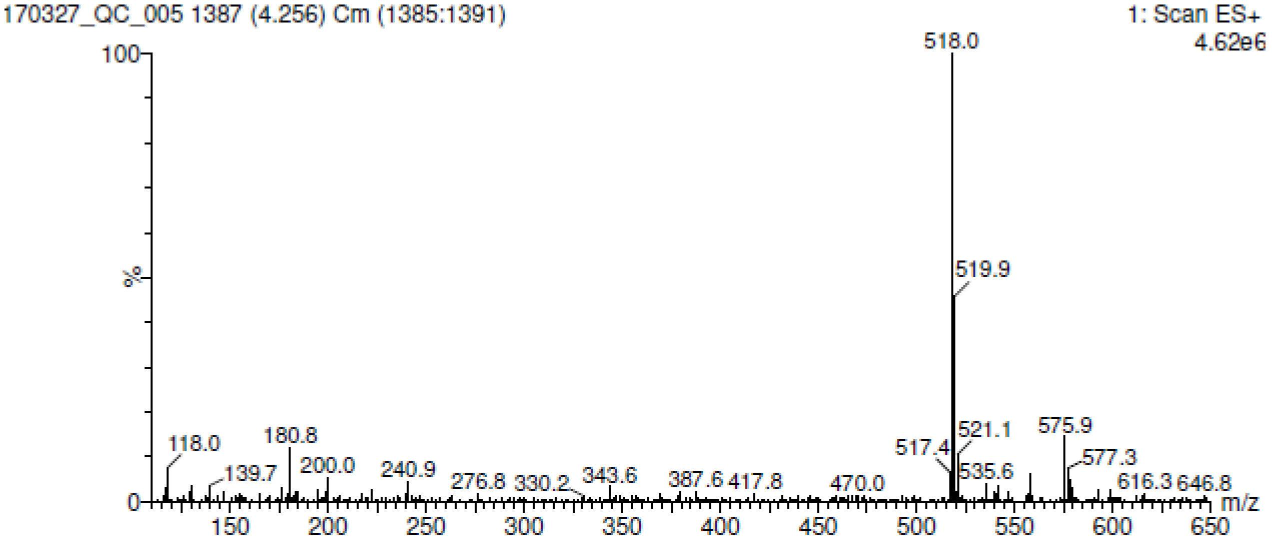
170327_QC_005 1387 (4.256) Cm (1385:1391)
1: Scan ES+
4.62e6
518.0
519.9
575.9
577.3
521.1
517.4
535.6
118.0
139.7
180.8
200.0
240.9
276.8
330.2
343.6
387.6
417.8
470.0
616.3
646.8
m/z
%

^{1}H-NMR spectrum (400 MHz, DMSO-d_6) of **10d**

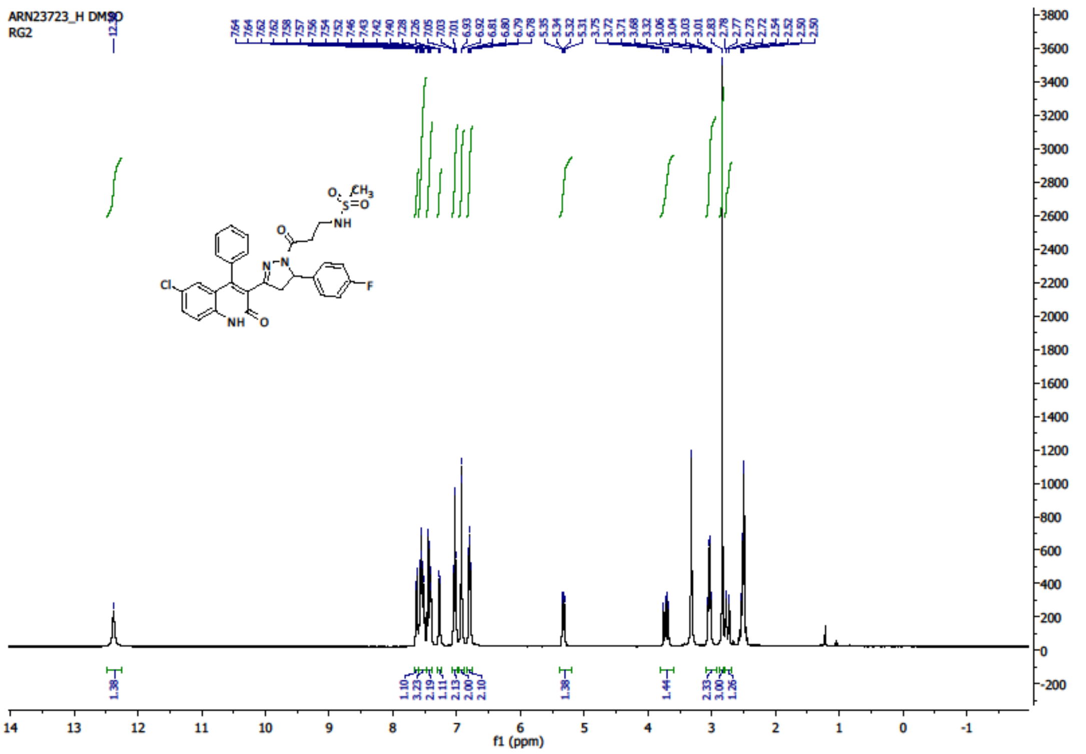

^{13}C-NMR spectrum (101 MHz, DMSO-d_6) of **10d**

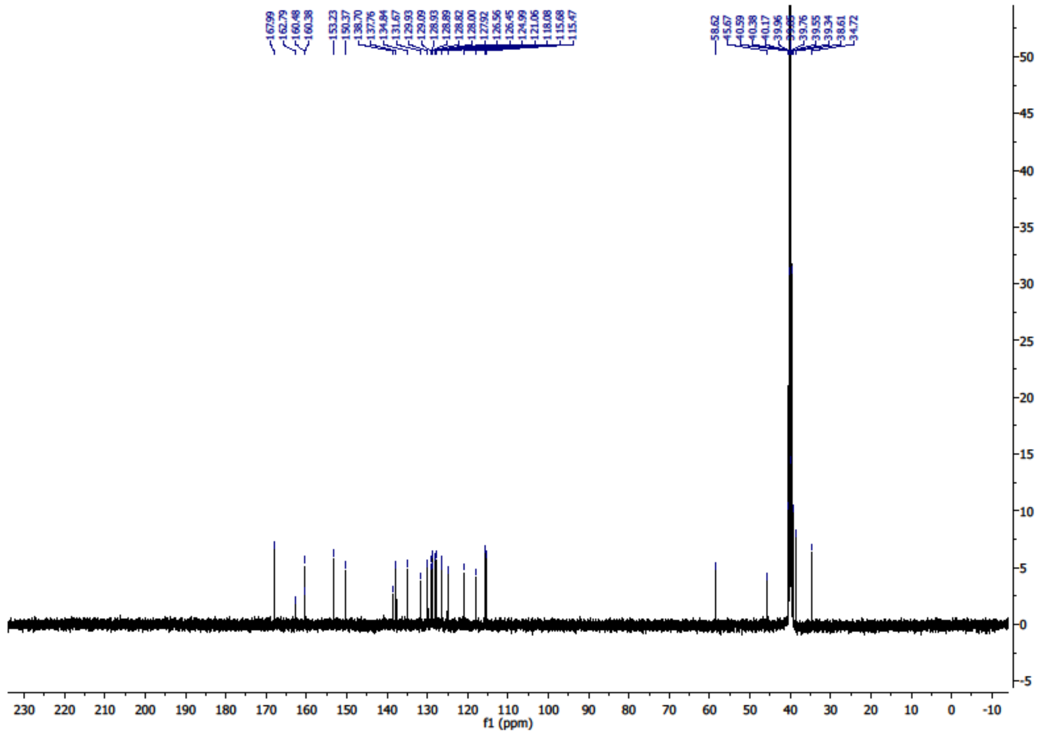

HPLC-MS analysis of **10d**

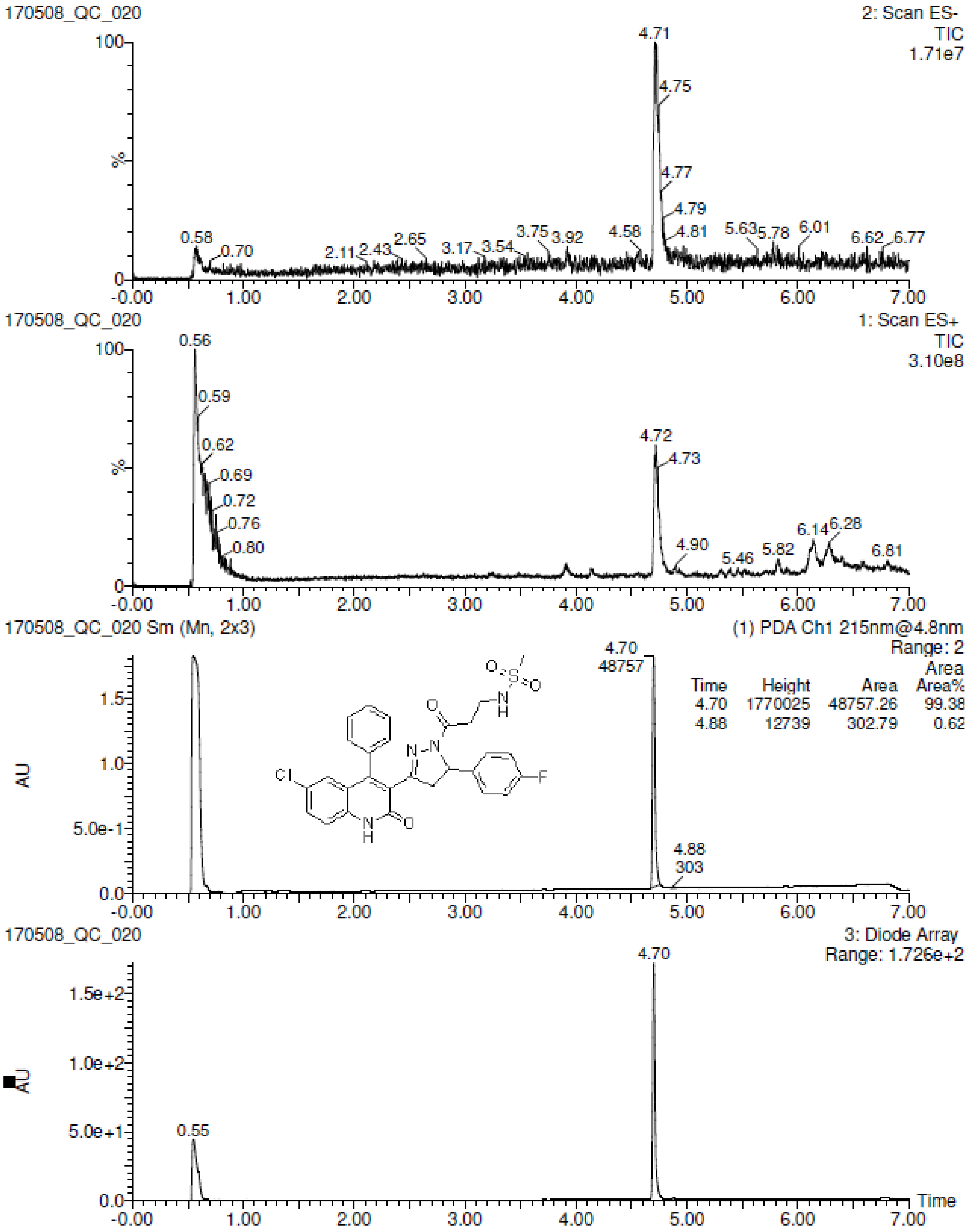

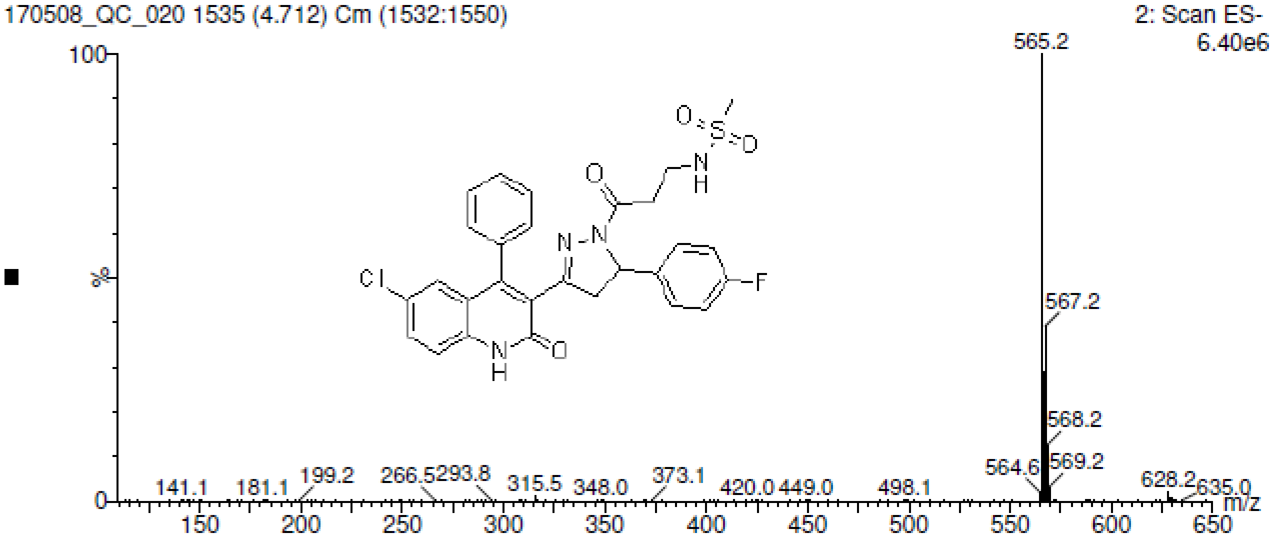

170508_QC_020 1535 (4.712) Cm (1532:1550)
2: Scan ES-
6.40e6
565.2
567.2
568.2
564.6
569.2
141.1
181.1
199.2
266.5
293.8
315.5
348.0
373.1
420.0
449.0
498.1
628.2
635.0
m/z
%

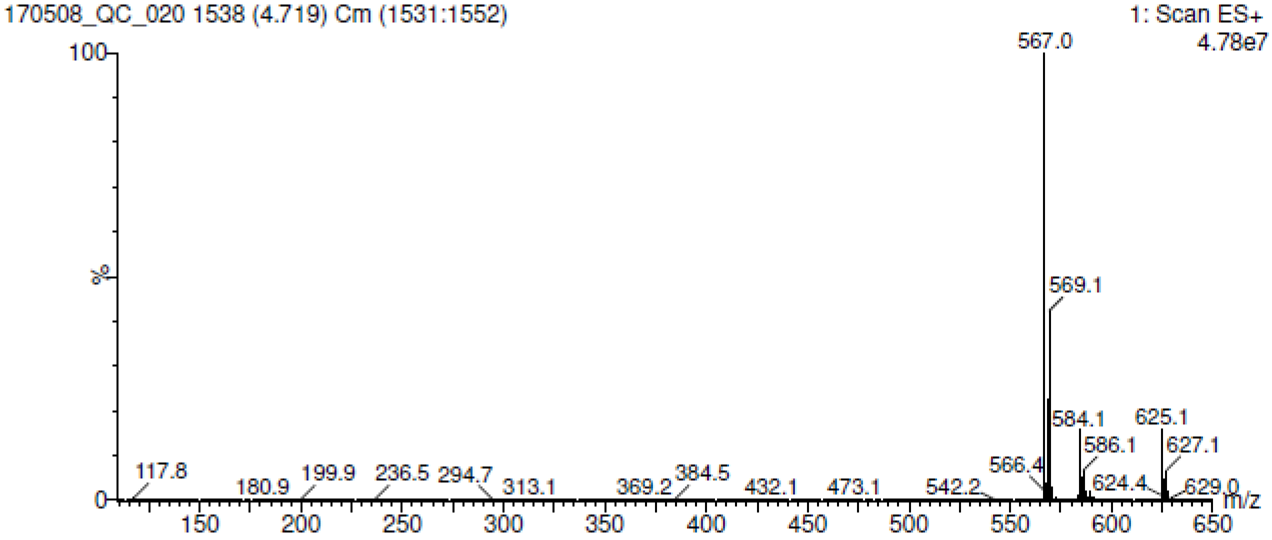

170508_QC_020 1538 (4.719) Cm (1531:1552)
1: Scan ES+
4.78e7
567.0
569.1
584.1
625.1
586.1
627.1
566.4
624.4
629.0
117.8
180.9
199.9
236.5
294.7
313.1
369.2
384.5
432.1
473.1
542.2
m/z
%

^{1}H-NMR spectrum (400 MHz, DMSO-d_6) of **14d**

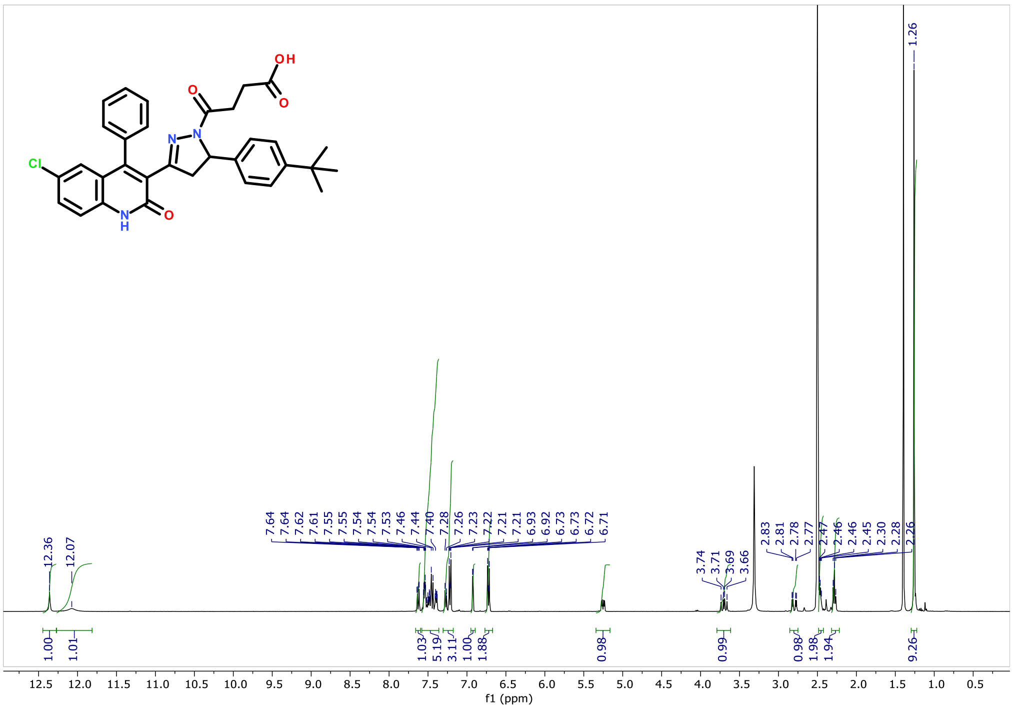

^{13}C-NMR spectrum (101 MHz, DMSO-d_6) of **14d**

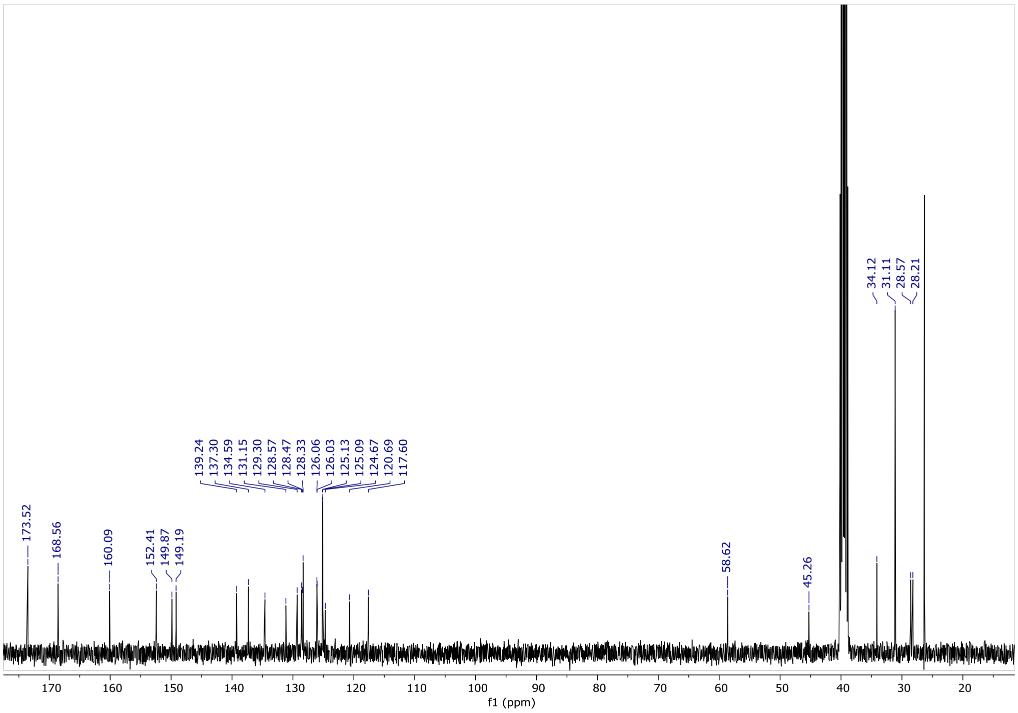

HPLC-MS analysis of **14d**

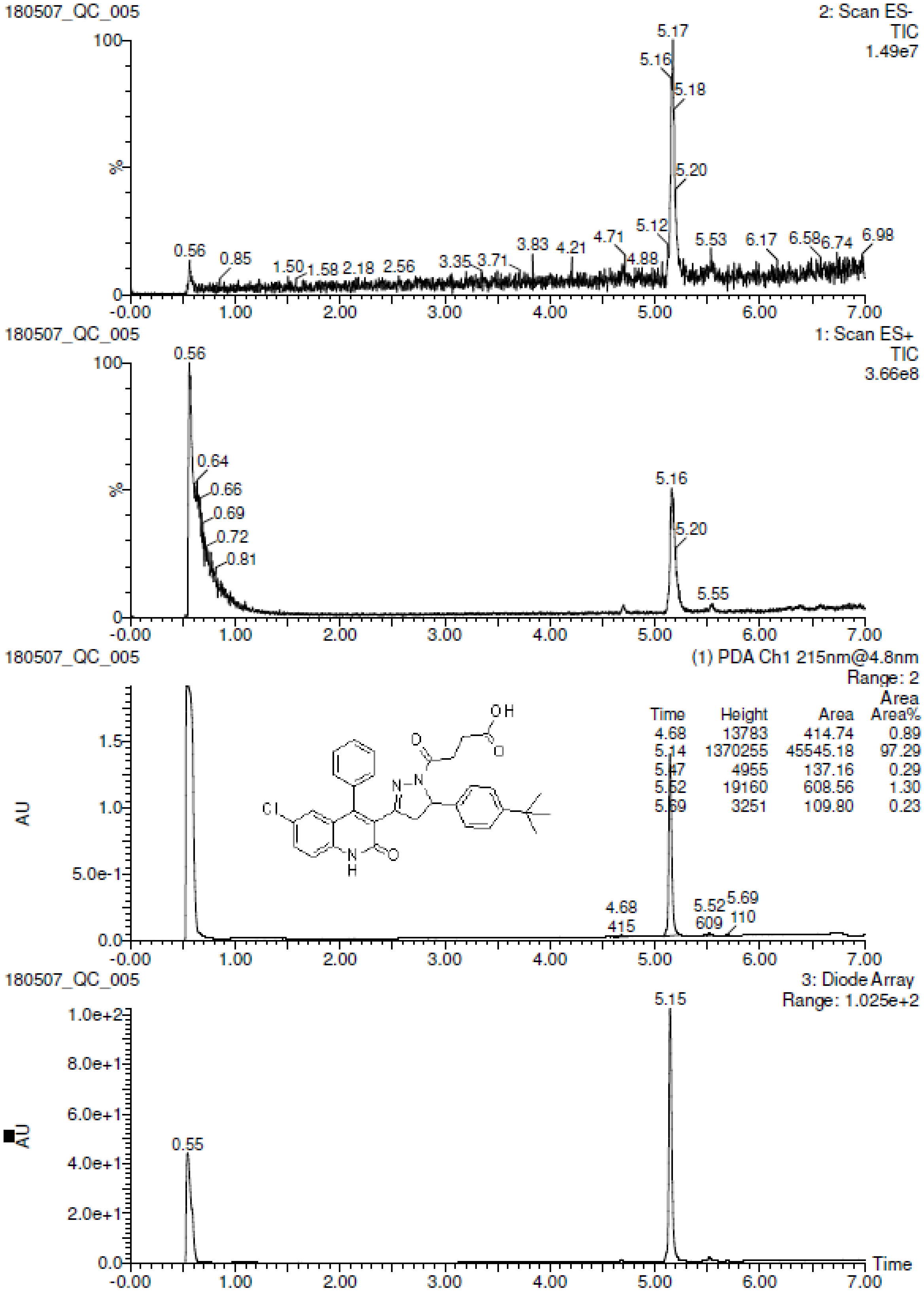

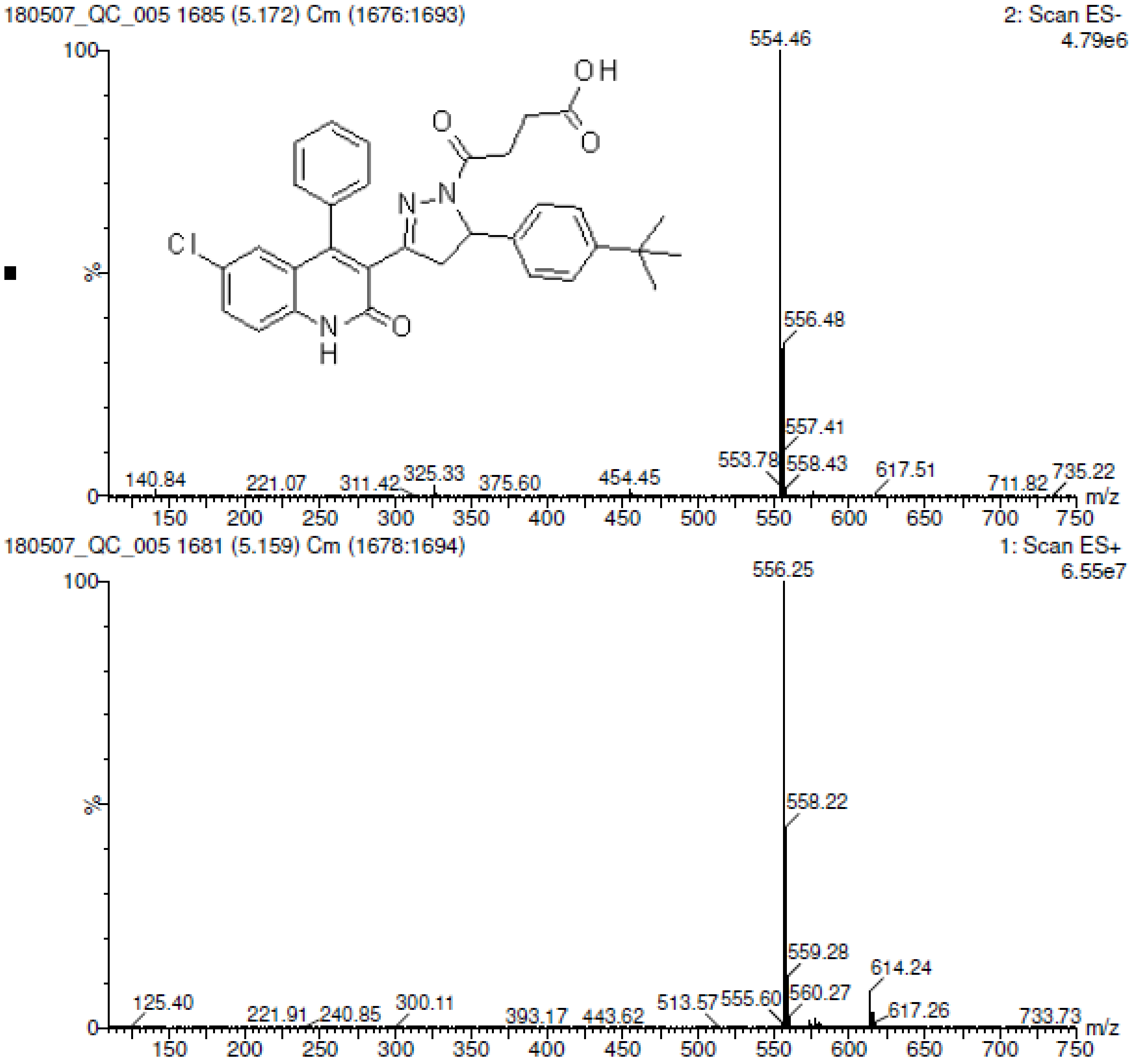
180507_QC_005 1685 (5.172) Cm (1676:1693)
2: Scan ES-
4.79e6
554.46
556.48
557.41
553.78
558.43
617.51
711.82
735.22
140.84
221.07
311.42
325.33
375.60
454.45
OH
O
O
N
N
Cl
N
H
O
%
100
0
150
200
250
300
350
400
450
500
550
600
650
700
750
m/z
180507_QC_005 1681 (5.159) Cm (1678:1694)
1: Scan ES+
6.55e7
556.25
558.22
559.28
614.24
560.27
617.26
555.60
513.57
443.62
393.17
300.11
221.91
240.85
125.40
733.73
%
100
0
150
200
250
300
350
400
450
500
550
600
650
700
750
m/z

^{1}H-NMR spectrum (400 MHz, DMSO-d_6) of **15d**

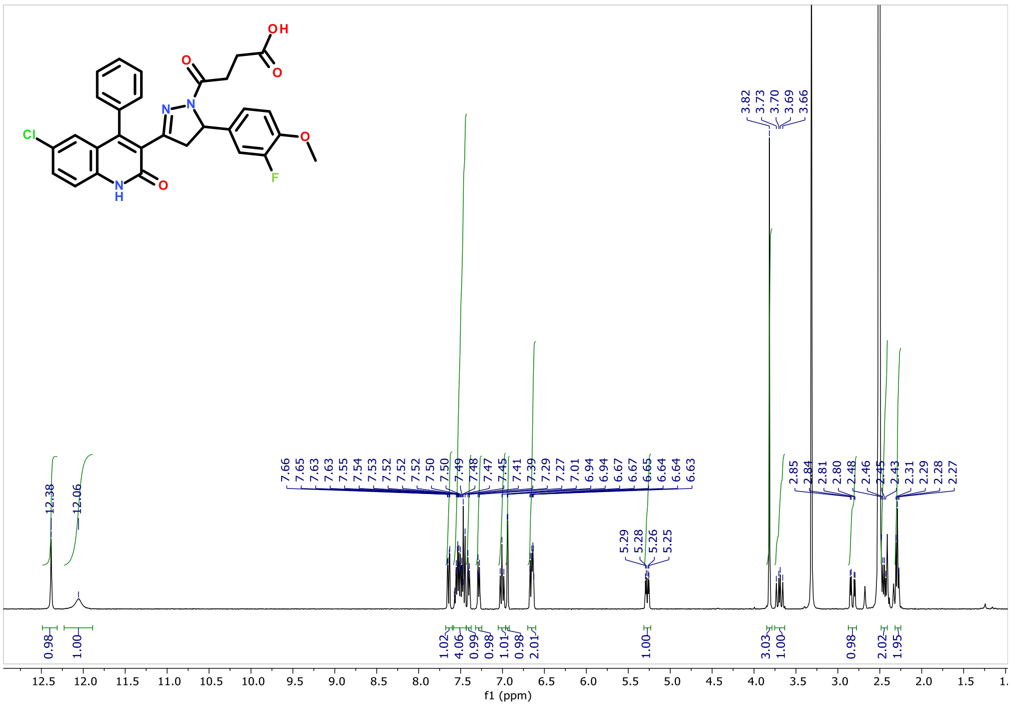

^{13}C-NMR spectrum (101 MHz, DMSO-d_6) of **15d**

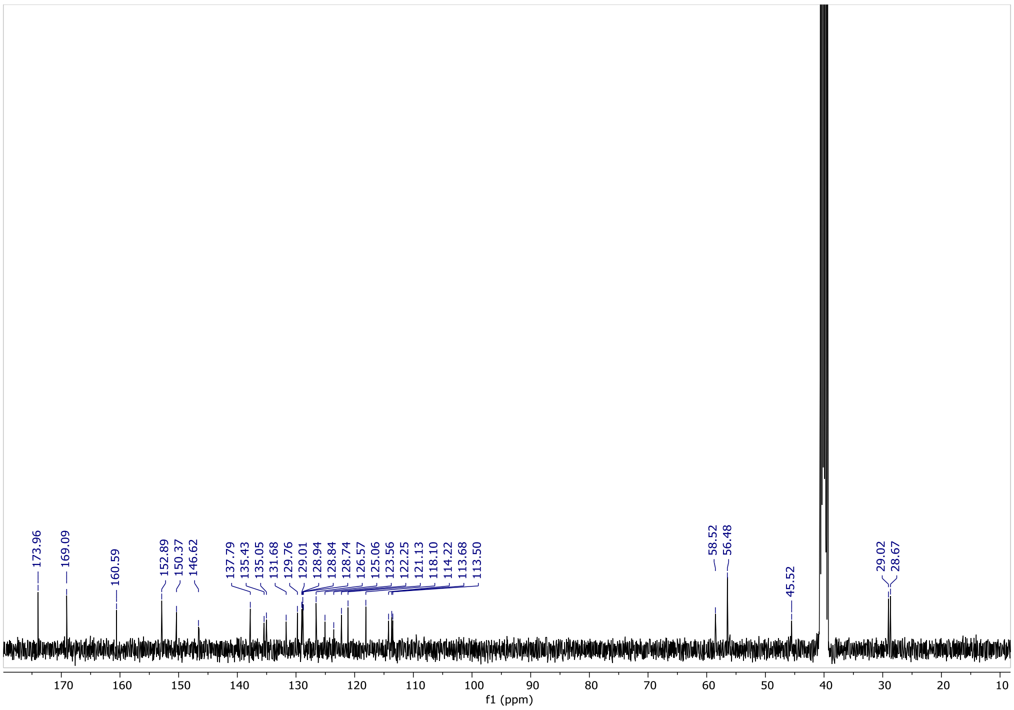

HPLC-MS analysis of **15d**

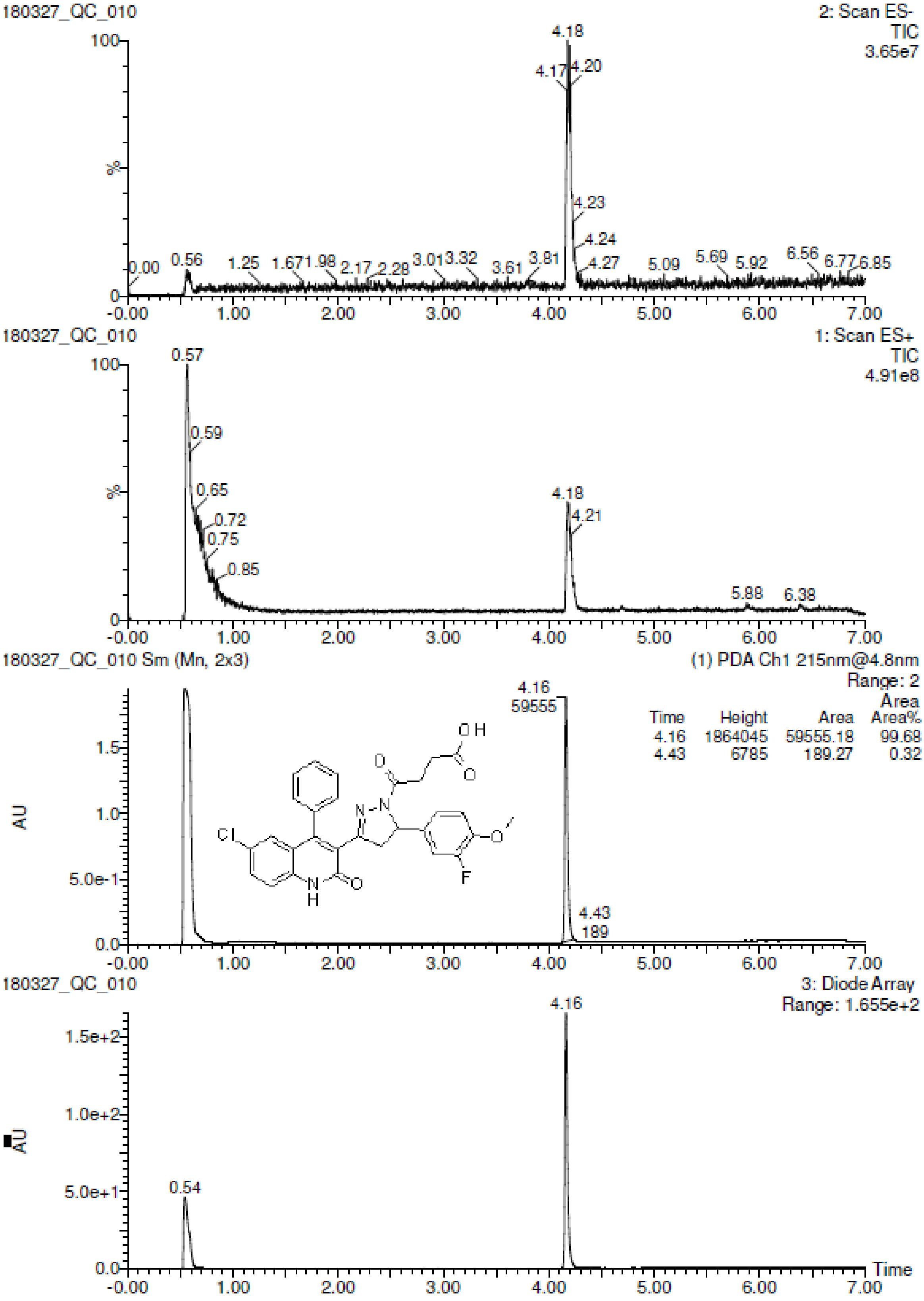

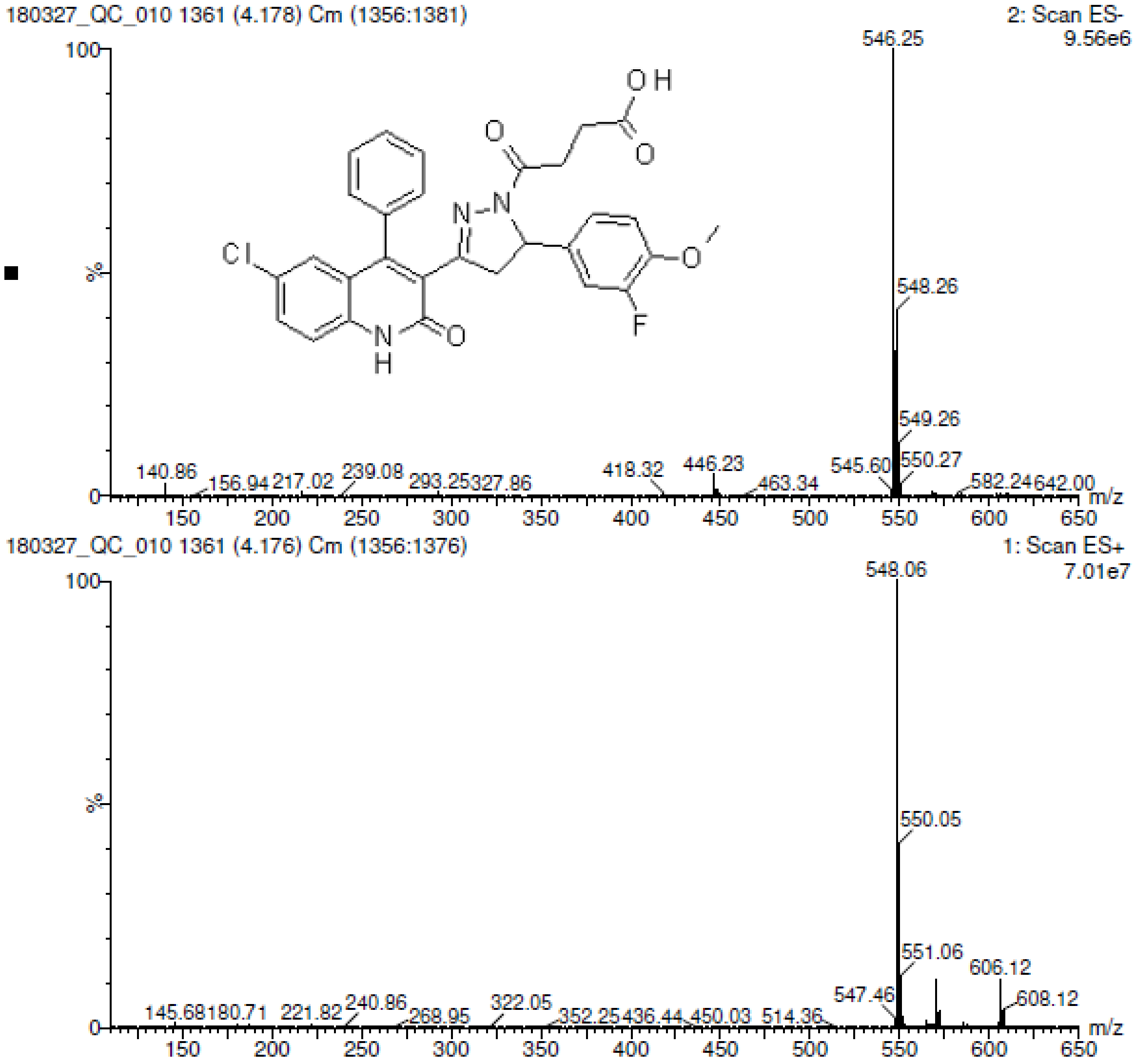

180327_QC_010 1361 (4.178) Cm (1356:1381)
2: Scan ES-
9.56e6
546.25
548.26
549.26
550.27
545.60
582.24
642.00
140.86
156.94
217.02
239.08
293.25
327.86
418.32
446.23
463.34
Cl
N
H
O
N
N
O
OH
O
F
O
%
m/z
180327_QC_010 1361 (4.176) Cm (1356:1376)
1: Scan ES+
7.01e7
548.06
550.05
551.06
606.12
608.12
547.46
145.68
180.71
221.82
240.86
268.95
322.05
352.25
436.44
450.03
514.36

^{1}H-NMR spectrum (400 MHz, DMSO-d_6) of **18d**

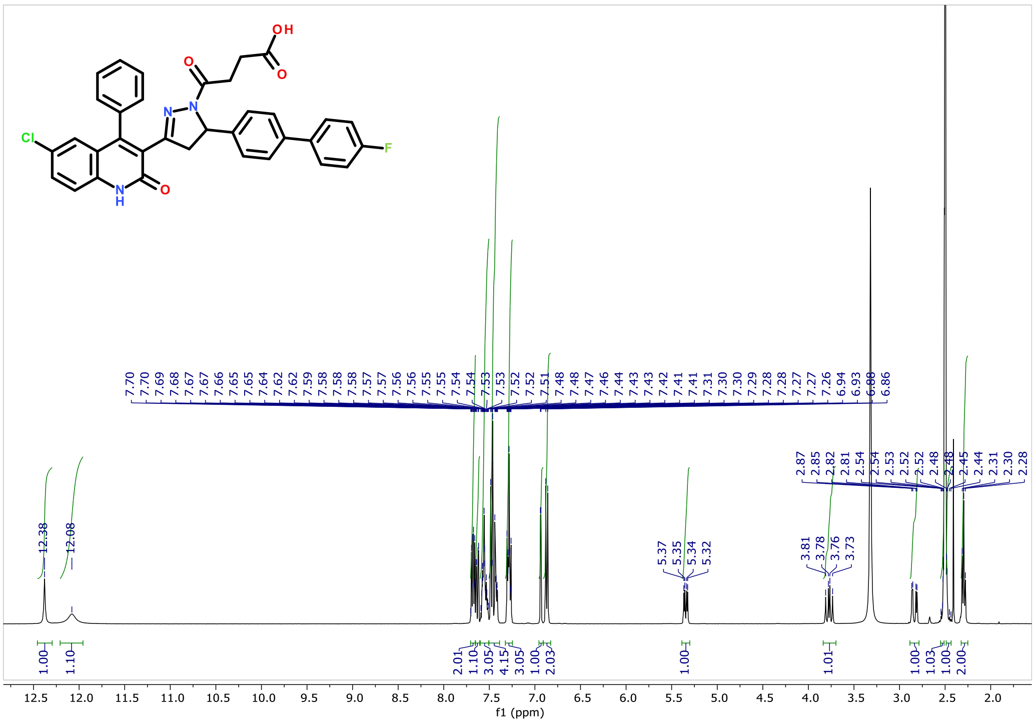

^{13}C-NMR spectrum (101 MHz, DMSO-d_6) of **18d**.

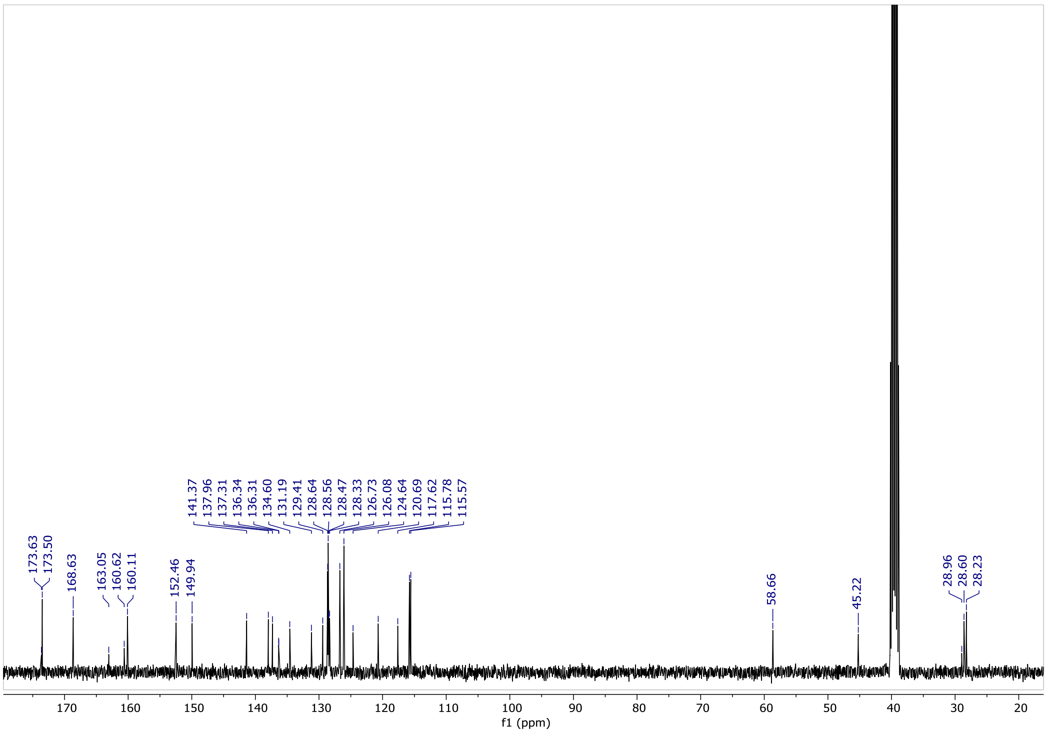

HPLC-MS analysis of **18d**

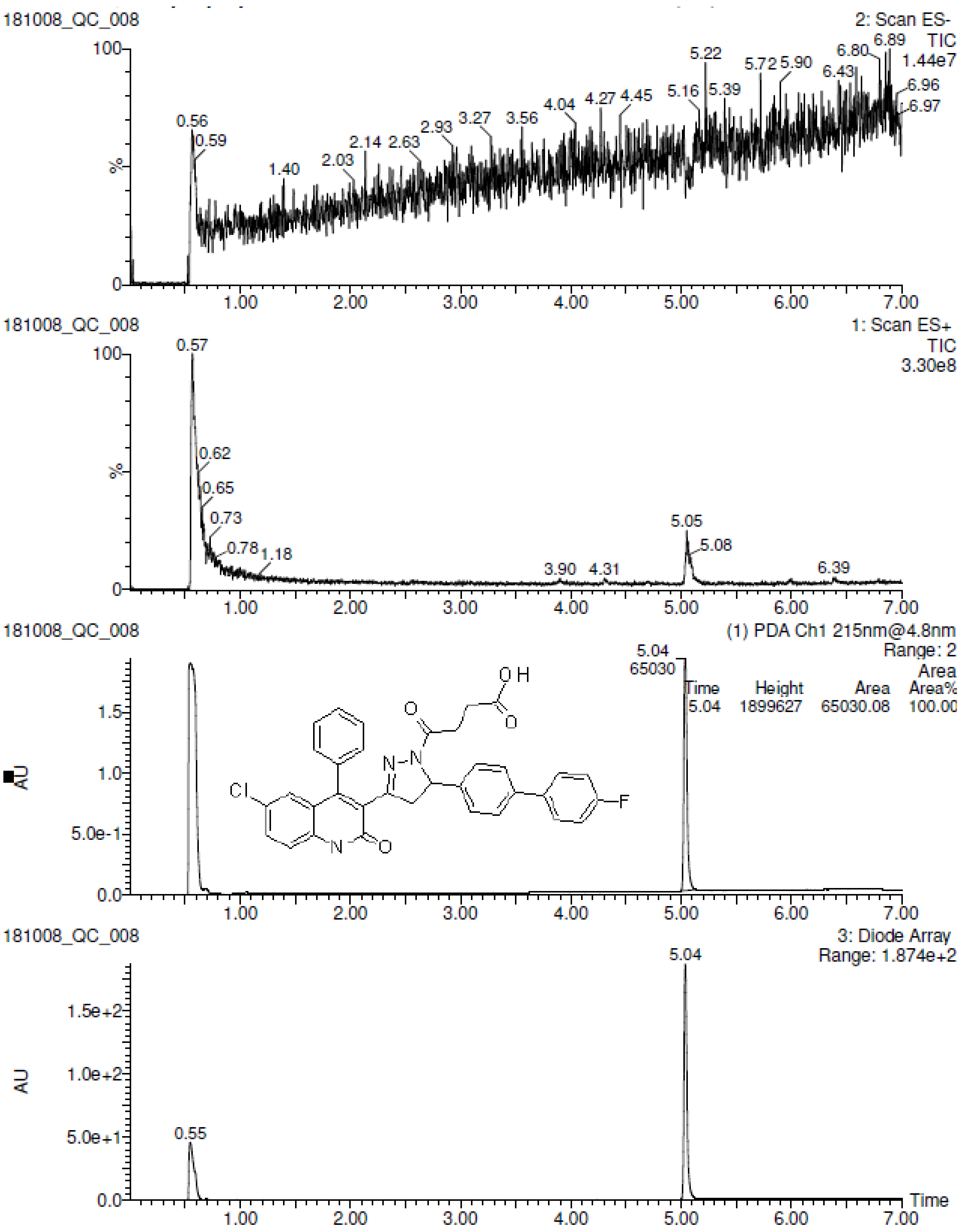

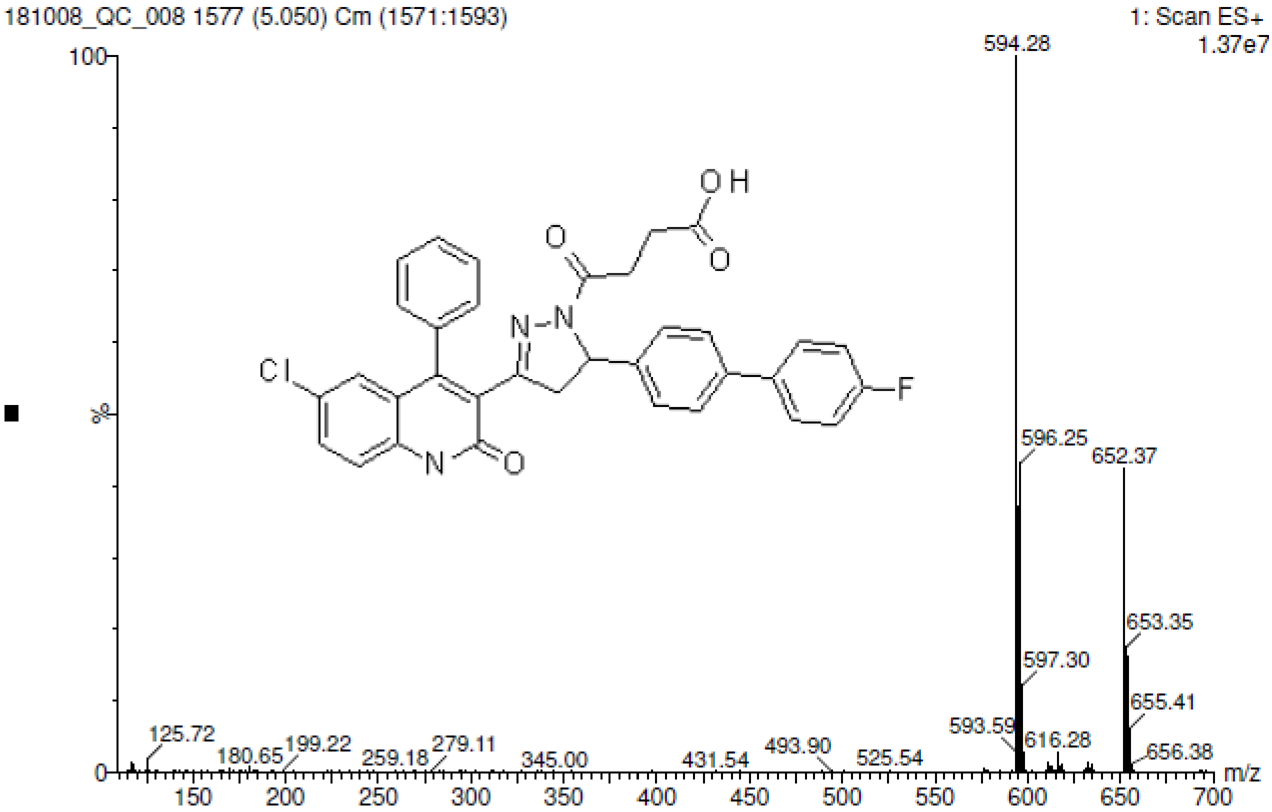
181008_QC_008 1577 (5.050) Cm (1571:1593)
1: Scan ES+
1.37e7
594.28
596.25
652.37
653.35
597.30
655.41
593.59
616.28
656.38
125.72
180.65
199.22
259.18
279.11
345.00
431.54
493.90
525.54
100
%
0
m/z
150
200
250
300
350
400
450
500
550
600
650
700
OH
O
O
N
N
Cl
F
N
O

^{1}H-NMR spectrum (400 MHz, DMSO-d_6) of **19d**

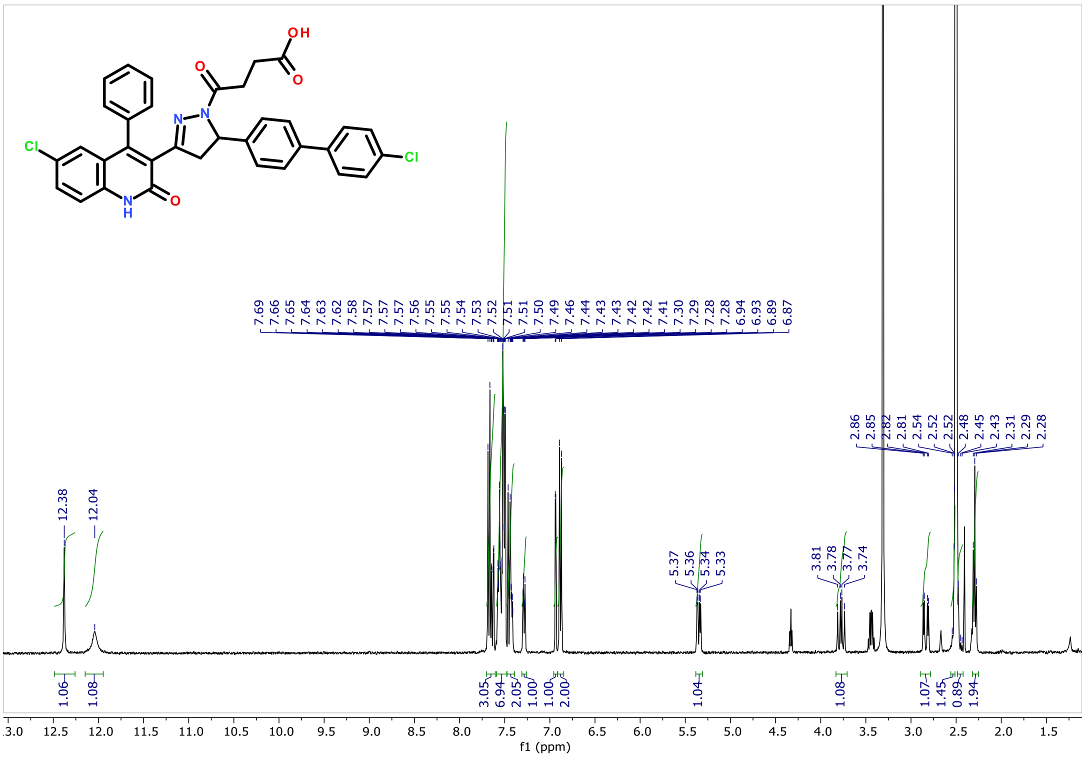

^{13}C-NMR spectrum (101 MHz, DMSO-d_6) of **19d**

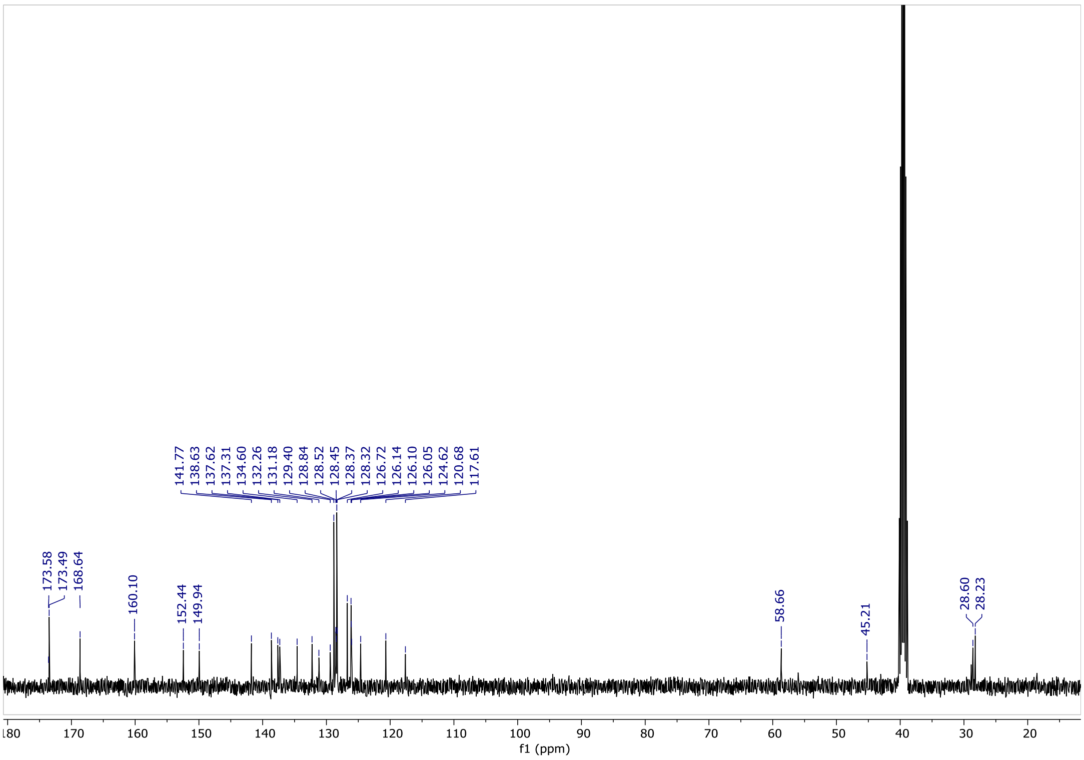

HPLC-MS analysis of **19d**

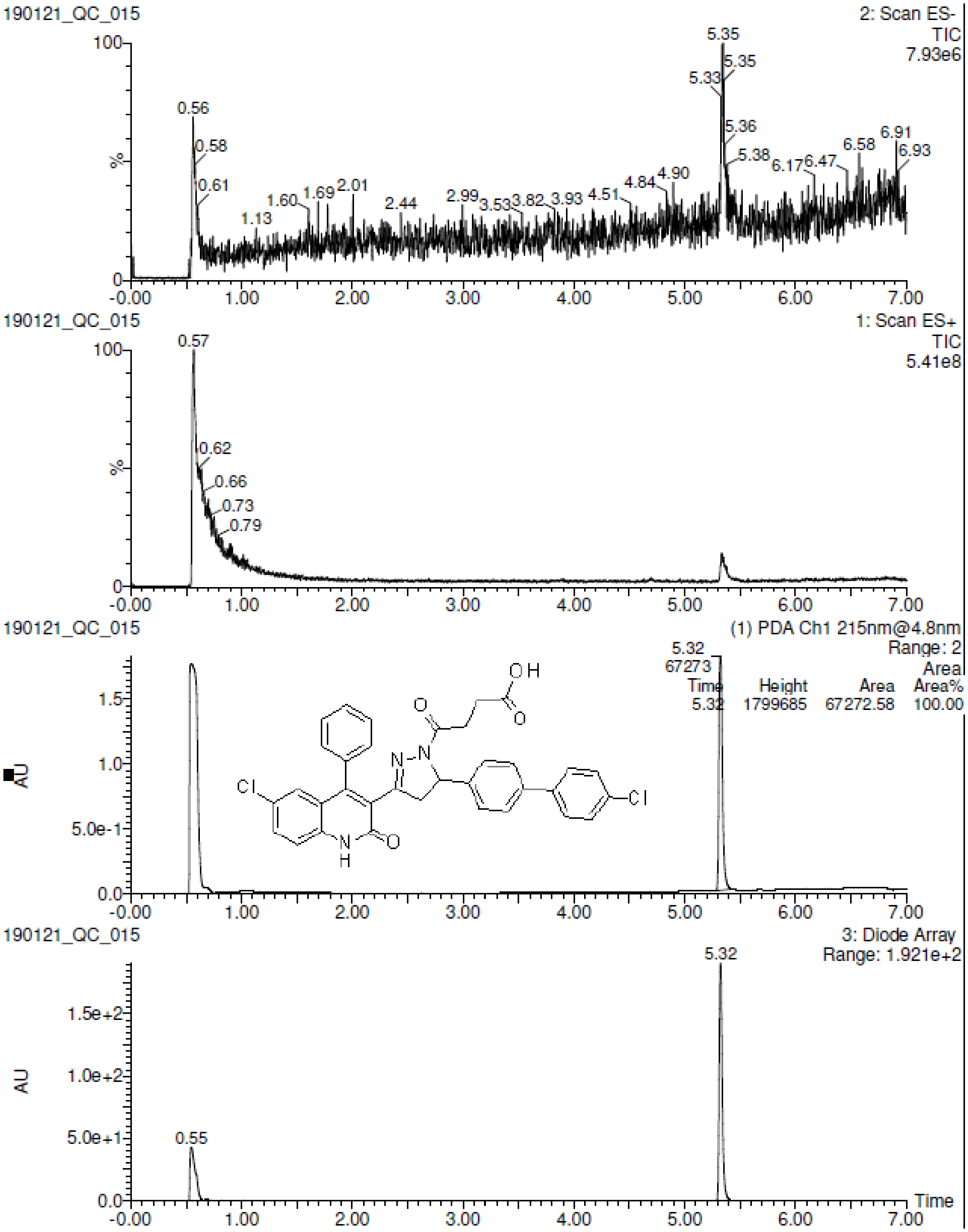

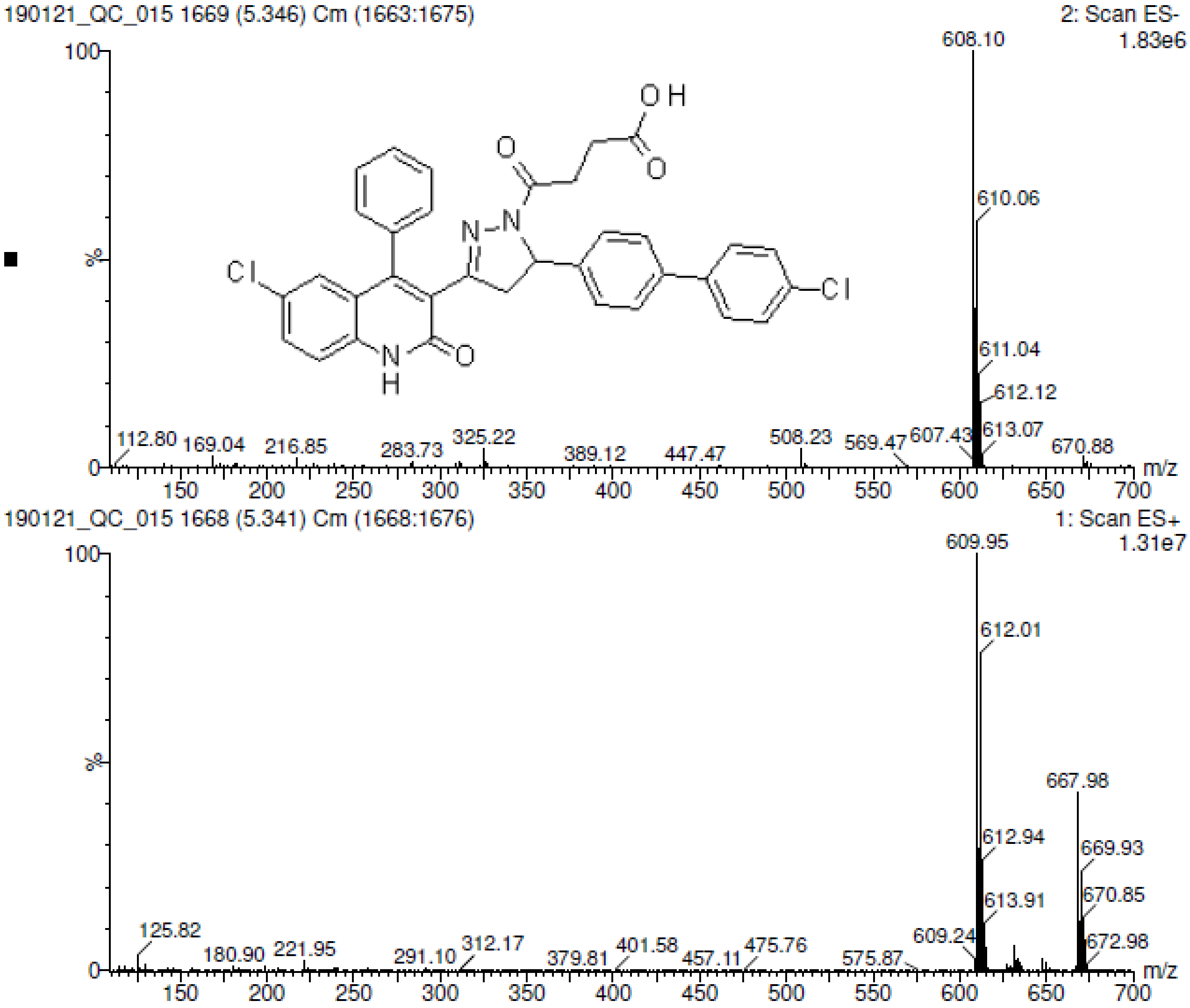
190121_QC_015 1669 (5.346) Cm (1663:1675)
2: Scan ES-
1.83e6
608.10
610.06
611.04
612.12
613.07
607.43
569.47
508.23
447.47
389.12
325.22
283.73
216.85
169.04
112.80
670.88
%
m/z
OH
O
O
N
N
Cl
Cl
N
H
O
190121_QC_015 1668 (5.341) Cm (1668:1676)
1: Scan ES+
1.31e7
609.95
612.01
612.94
613.91
609.24
667.98
669.93
670.85
672.98
575.87
475.76
457.11
401.58
379.81
312.17
291.10
221.95
180.90
125.82

^{1}H-NMR spectrum (400 MHz, DMSO-d_6) of **20d**

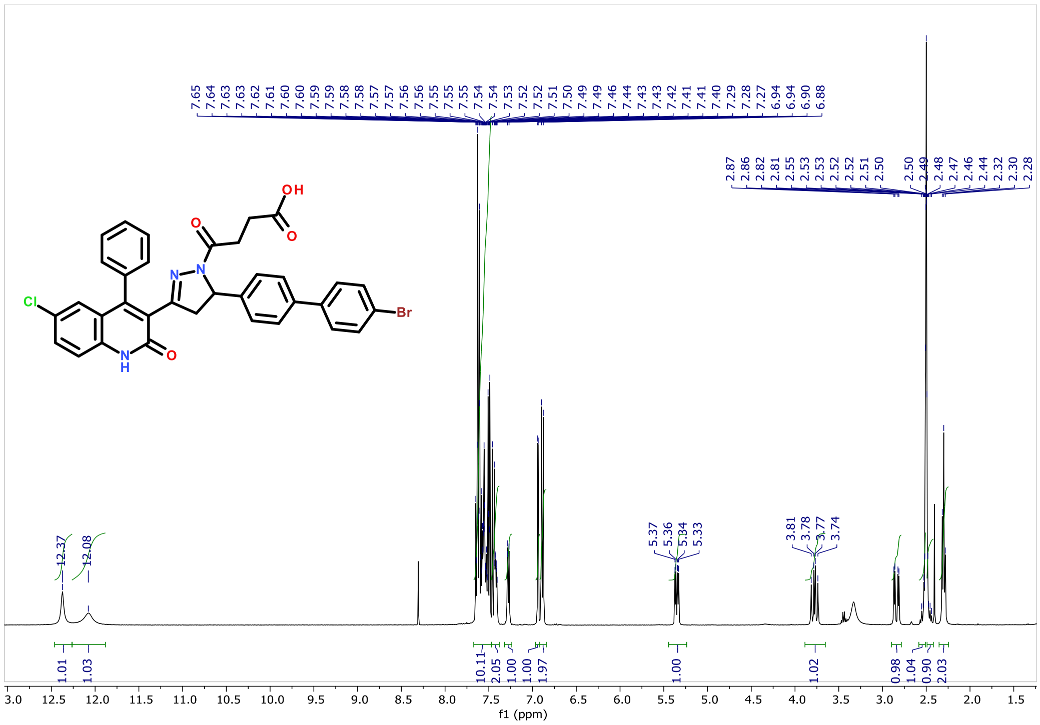

^{13}C-NMR spectrum (101 MHz, DMSO-d_6) of **20d**

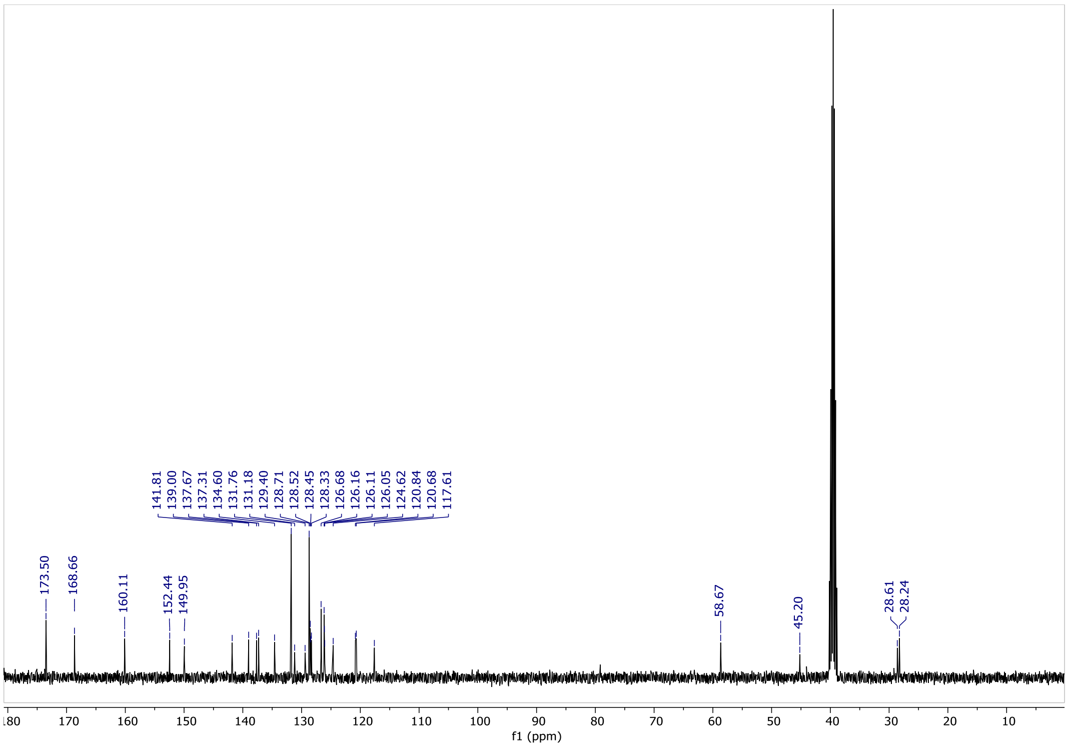

HPLC-MS analysis of **20d**

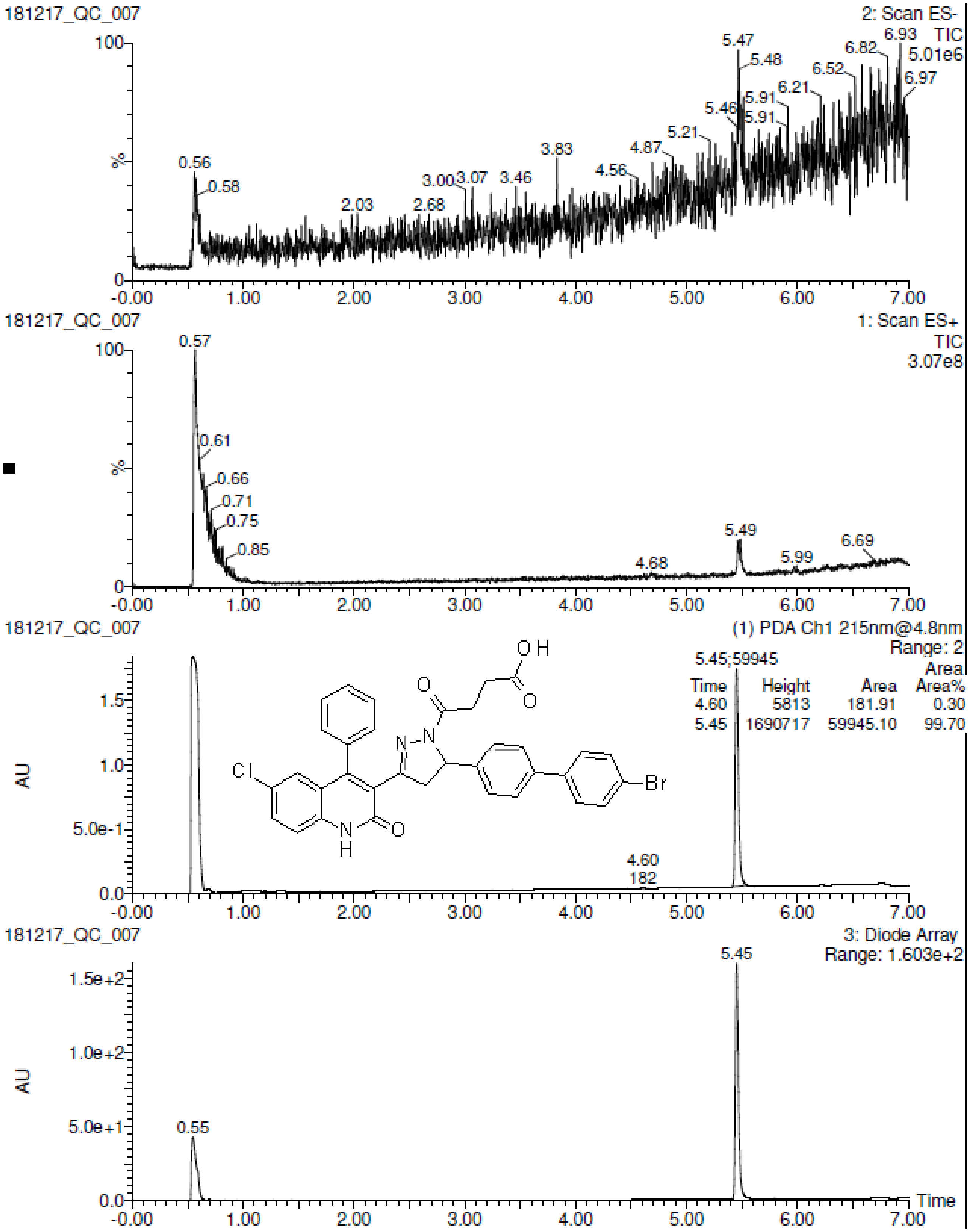

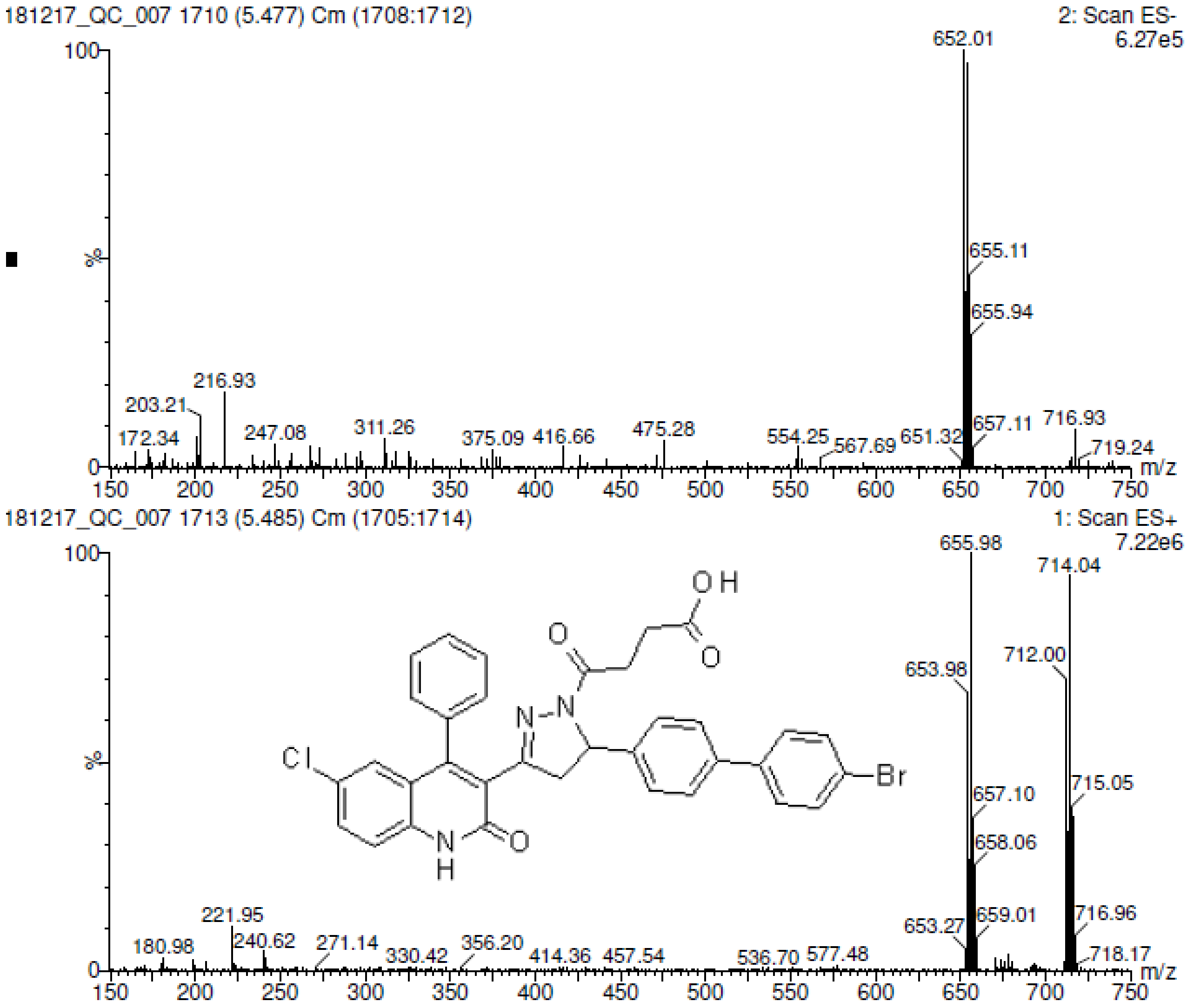
181217_QC_007 1710 (5.477) Cm (1708:1712)
2: Scan ES-
6.27e5
652.01
655.11
655.94
216.93
203.21
172.34
247.08
311.26
375.09
416.66
475.28
554.25
567.69
651.32
657.11
716.93
719.24
m/z
%
181217_QC_007 1713 (5.485) Cm (1705:1714)
1: Scan ES+
7.22e6
655.98
714.04
653.98
712.00
657.10
715.05
658.06
653.27
659.01
716.96
718.17
221.95
180.98
240.62
271.14
330.42
356.20
414.36
457.54
536.70
577.48
OH
O
O
N
N
Cl
Br
N
H
O

^{1}H-NMR spectrum (400 MHz, DMSO-d_6) of **21d**

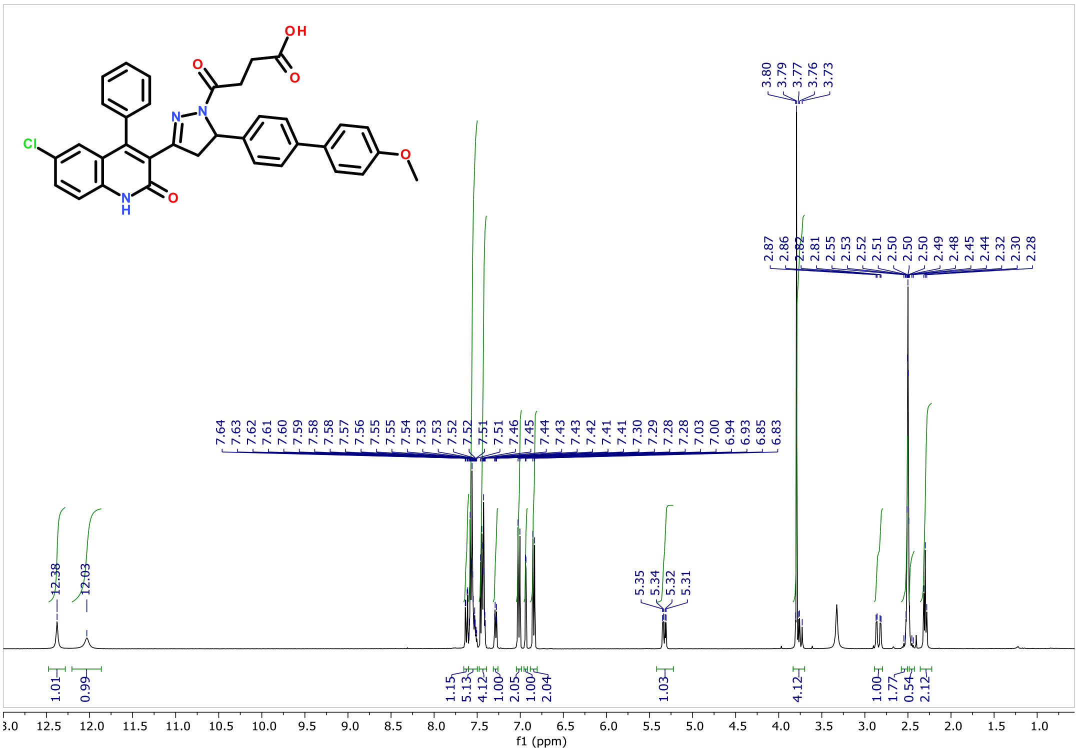

^{13}C-NMR spectrum (101 MHz, DMSO-d_6) of **21d**

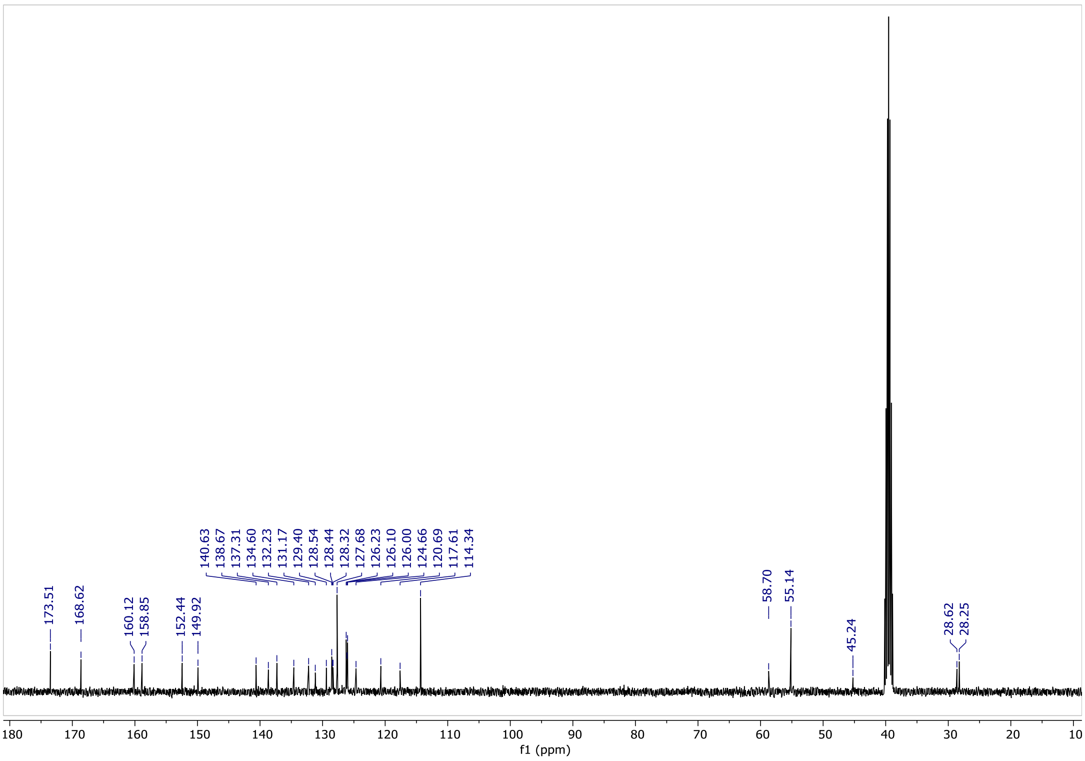

HPLC-MS analysis of **21d**

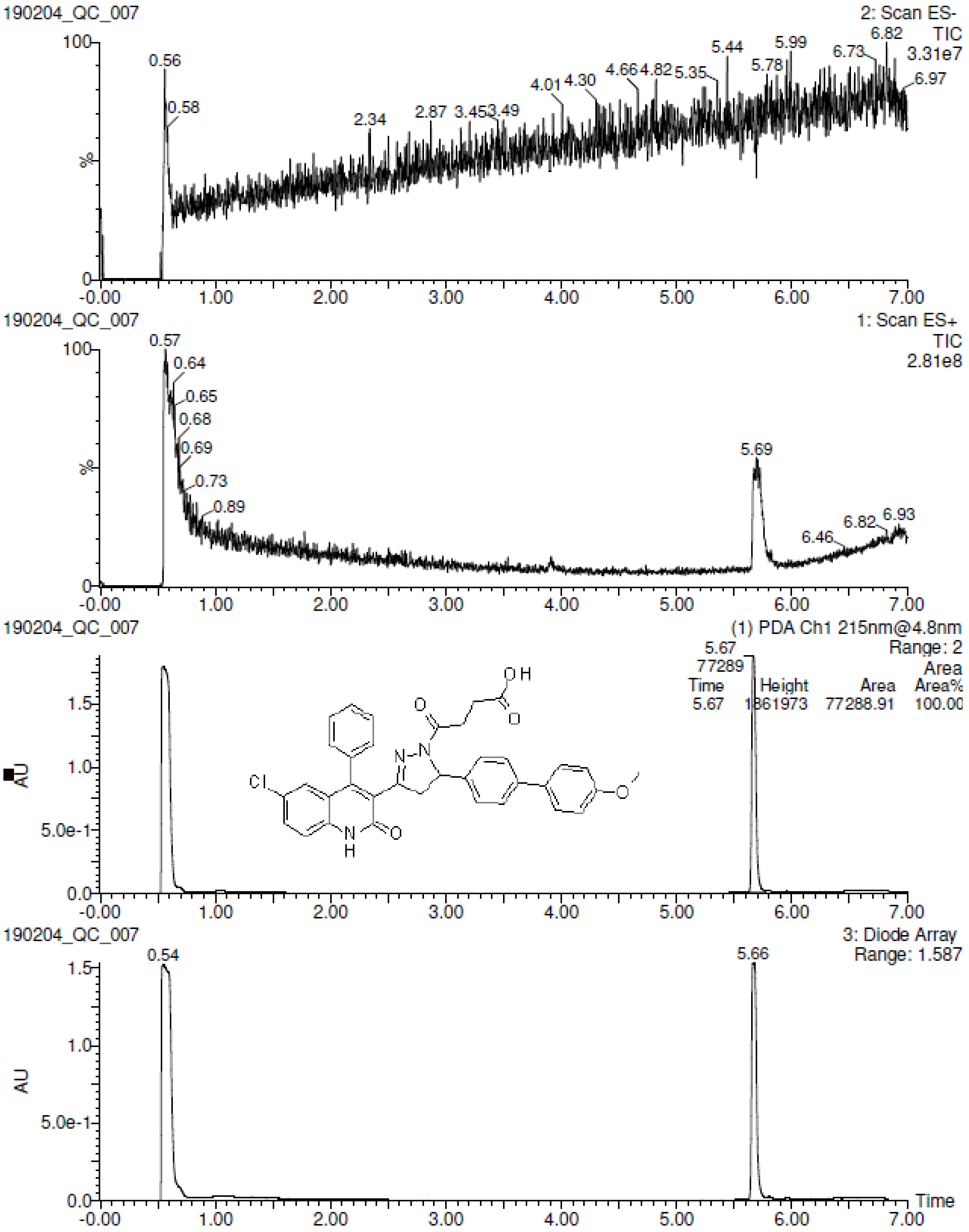

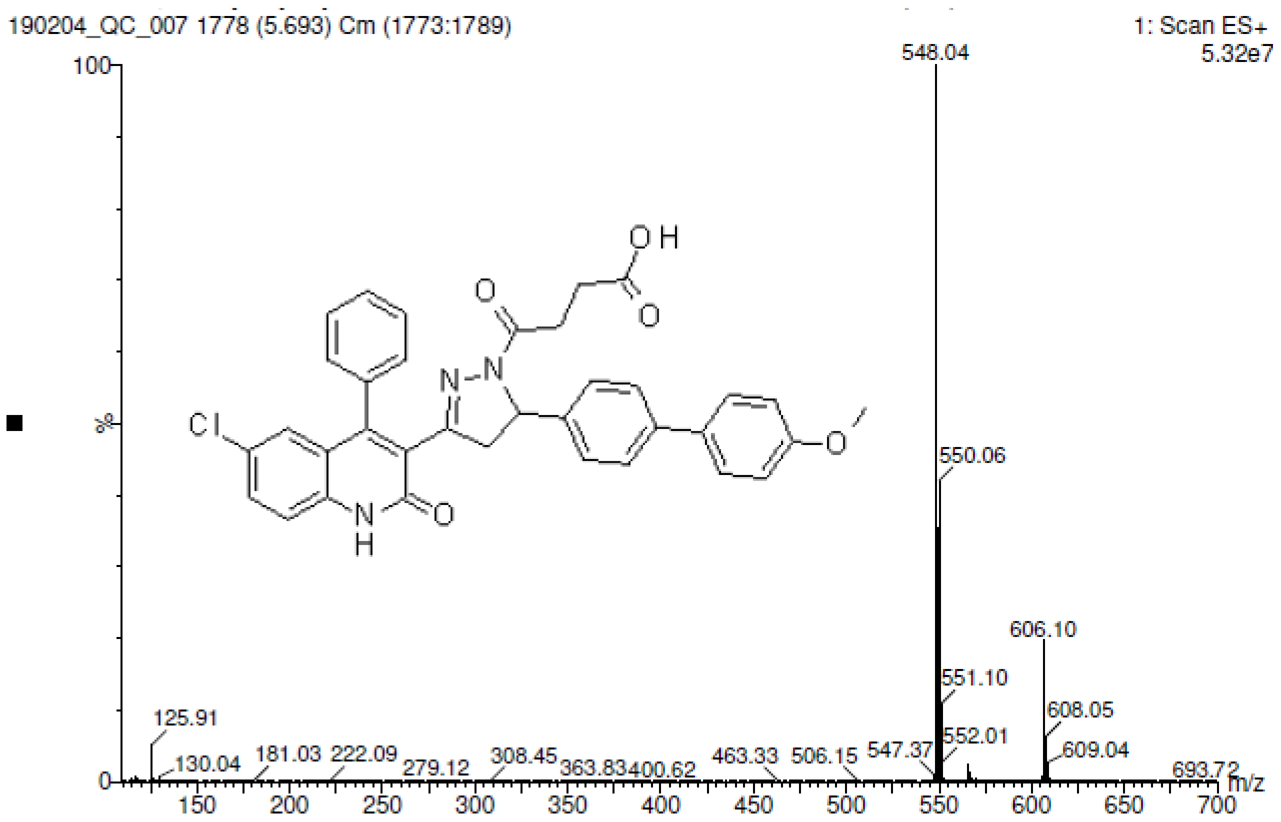

190204_QC_007 1778 (5.693) Cm (1773:1789)
1: Scan ES+
5.32e7
100
%
0
548.04
550.06
606.10
551.10
608.05
552.01
609.04
125.91
130.04
181.03
222.09
279.12
308.45
363.83
400.62
463.33
506.15
547.37
693.72
m/z
150
200
250
300
350
400
450
500
550
600
650
700
OH
O
O
N
N
Cl
N
H
O
O

^{1}H-NMR spectrum (400 MHz, DMSO-d_6) of **22d**

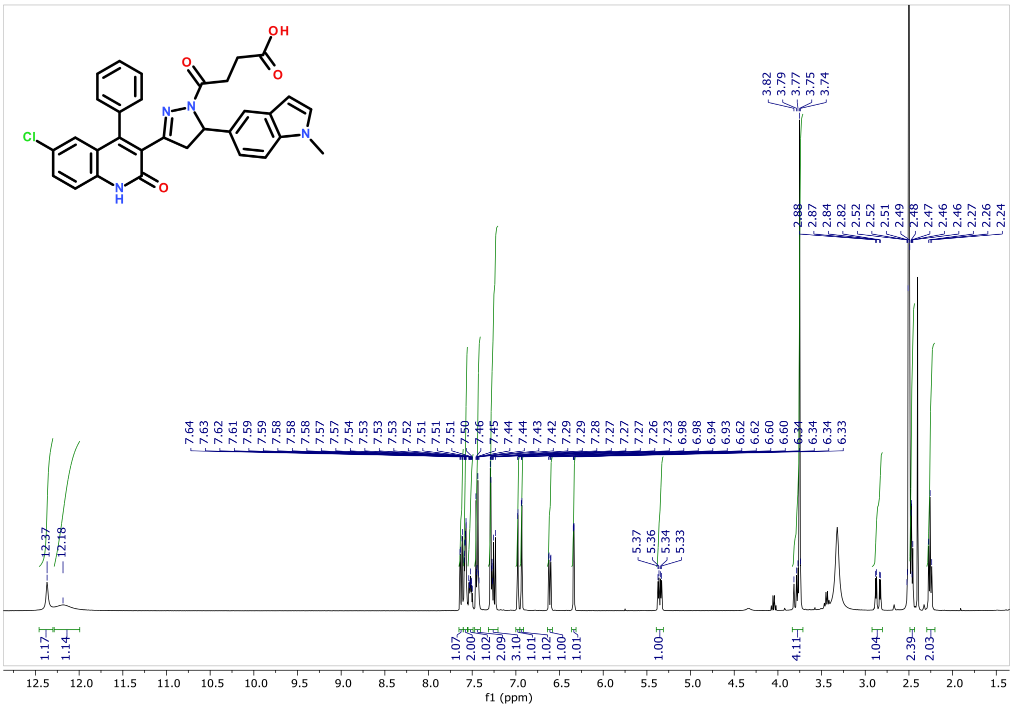

^{13}C-NMR spectrum (101 MHz, DMSO-d_6) of **22d**

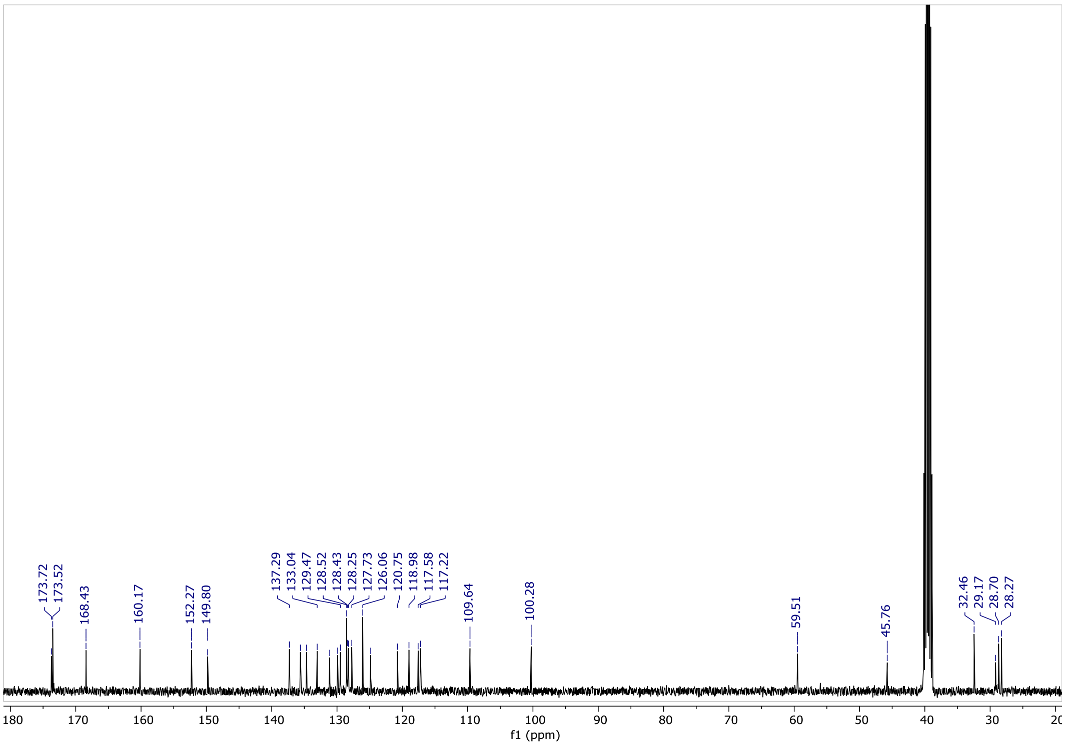

HPLC-MS analysis of **22d**

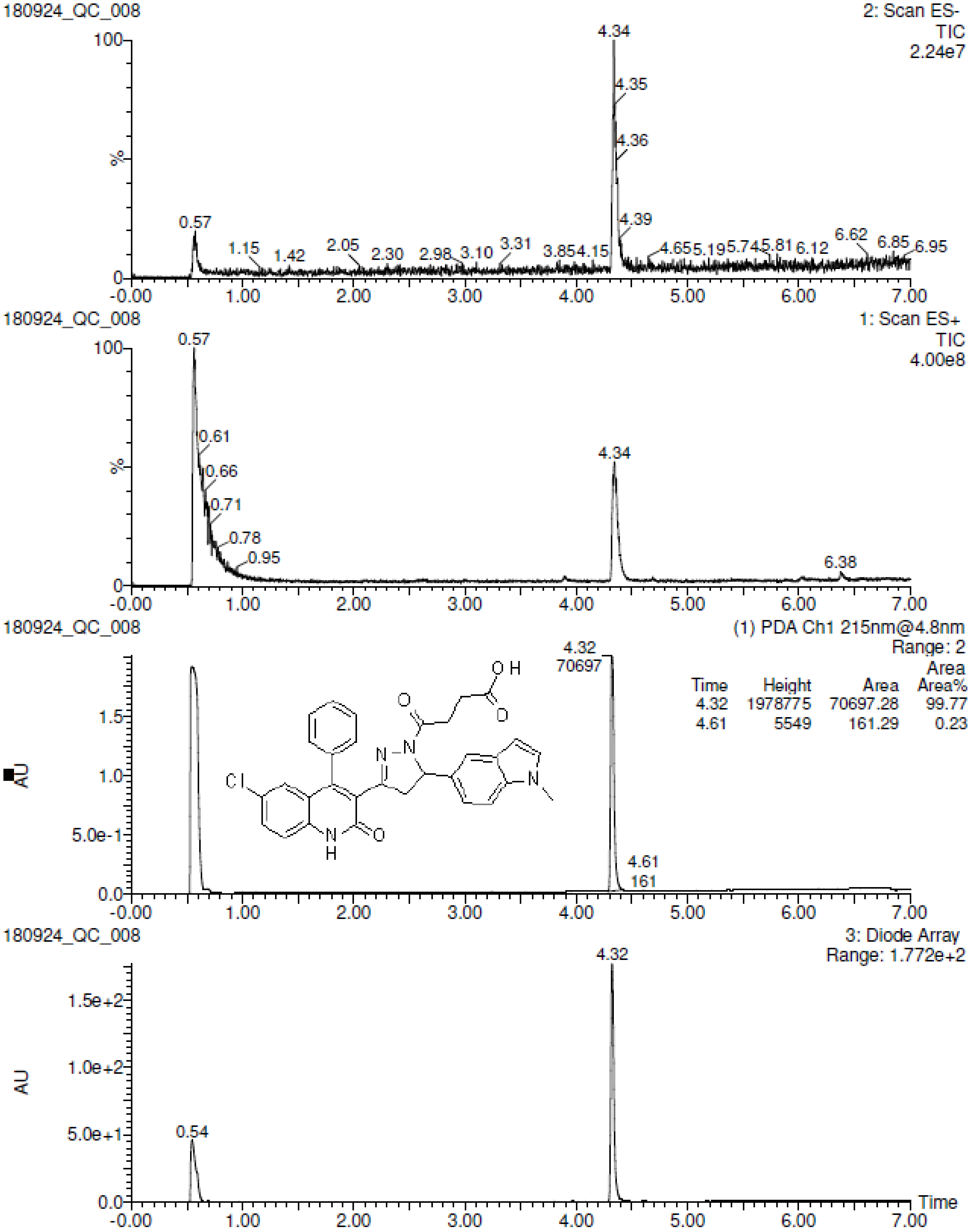

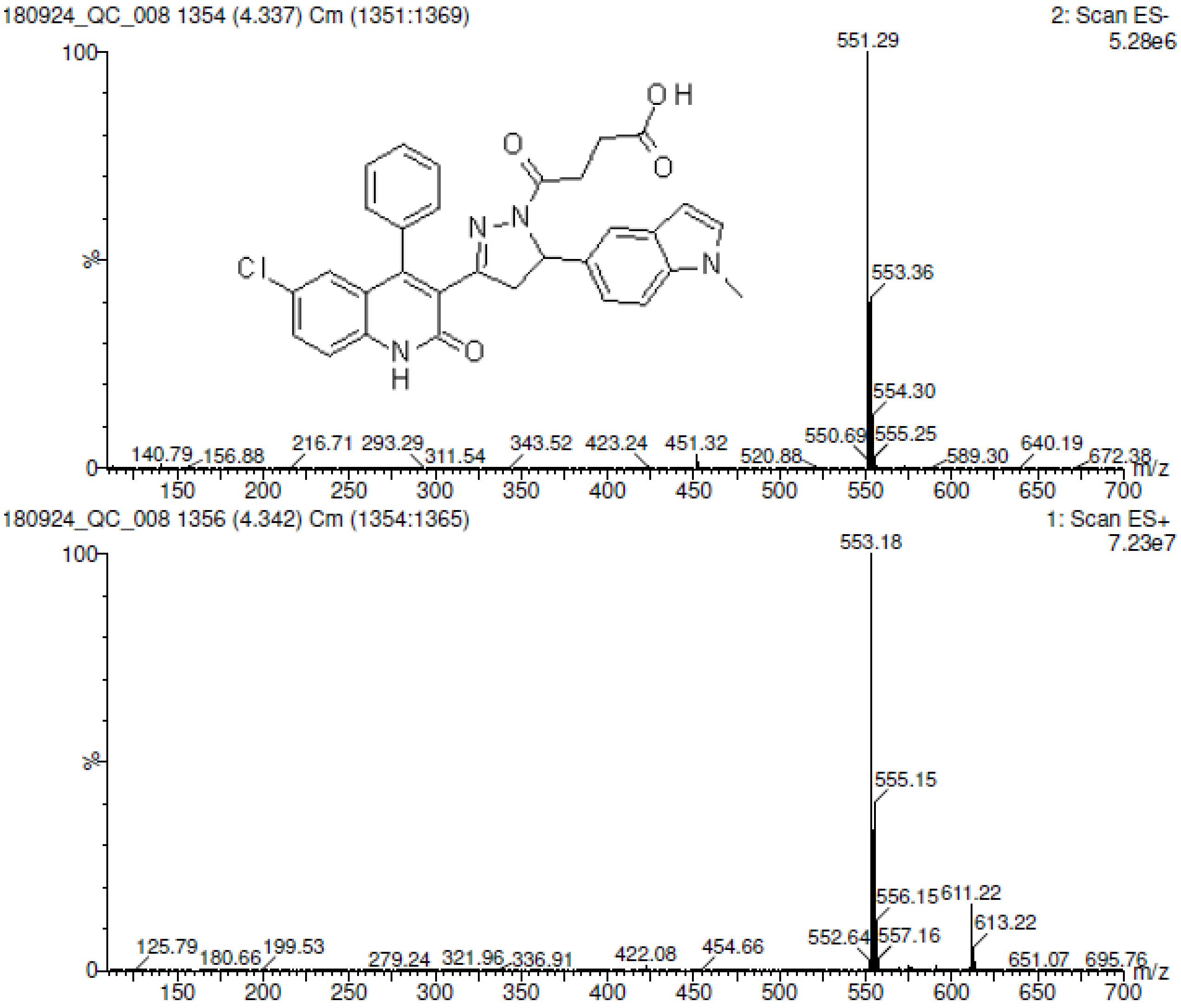

180924_QC_008 1354 (4.337) Cm (1351:1369)
2: Scan ES-
5.28e6
551.29
553.36
554.30
550.69
555.25
140.79
156.88
216.71
293.29
311.54
343.52
423.24
451.32
520.88
589.30
640.19
672.38
m/z
%
OH
O
O
N
N
Cl
N
H
O
N
180924_QC_008 1356 (4.342) Cm (1354:1365)
1: Scan ES+
7.23e7
553.18
555.15
556.15
611.22
613.22
552.64
557.16
125.79
180.66
199.53
279.24
321.96
336.91
422.08
454.66
651.07
695.76

^{1}H-NMR spectrum (400 MHz, DMSO-d_6) of **23d**

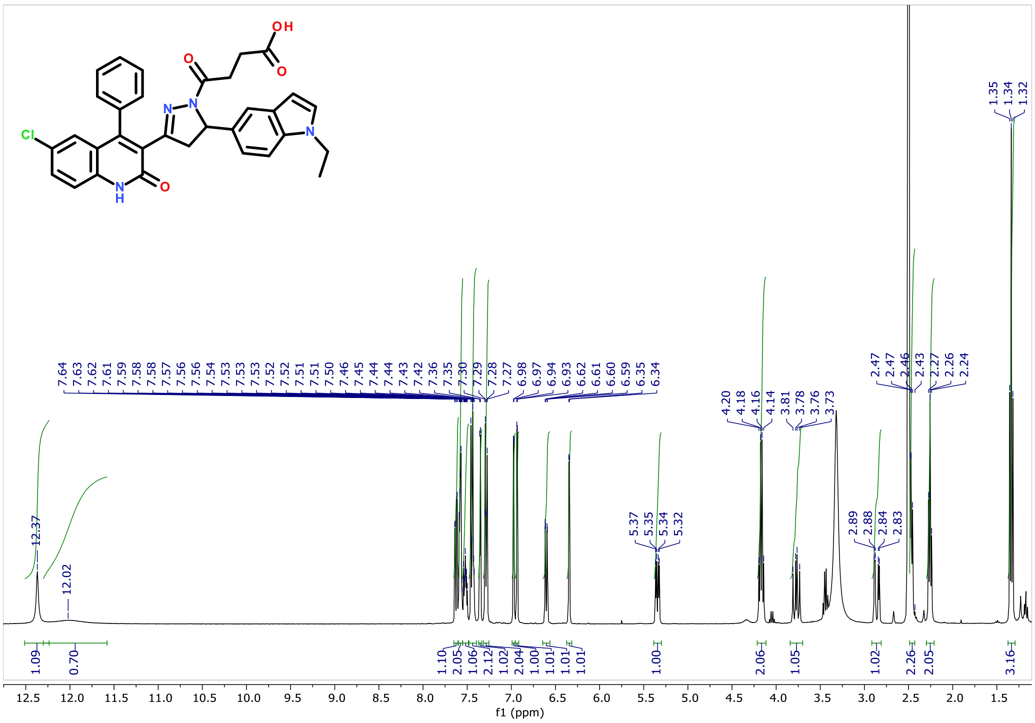

^{13}C-NMR spectrum (101 MHz, DMSO-d_6) of **23d**

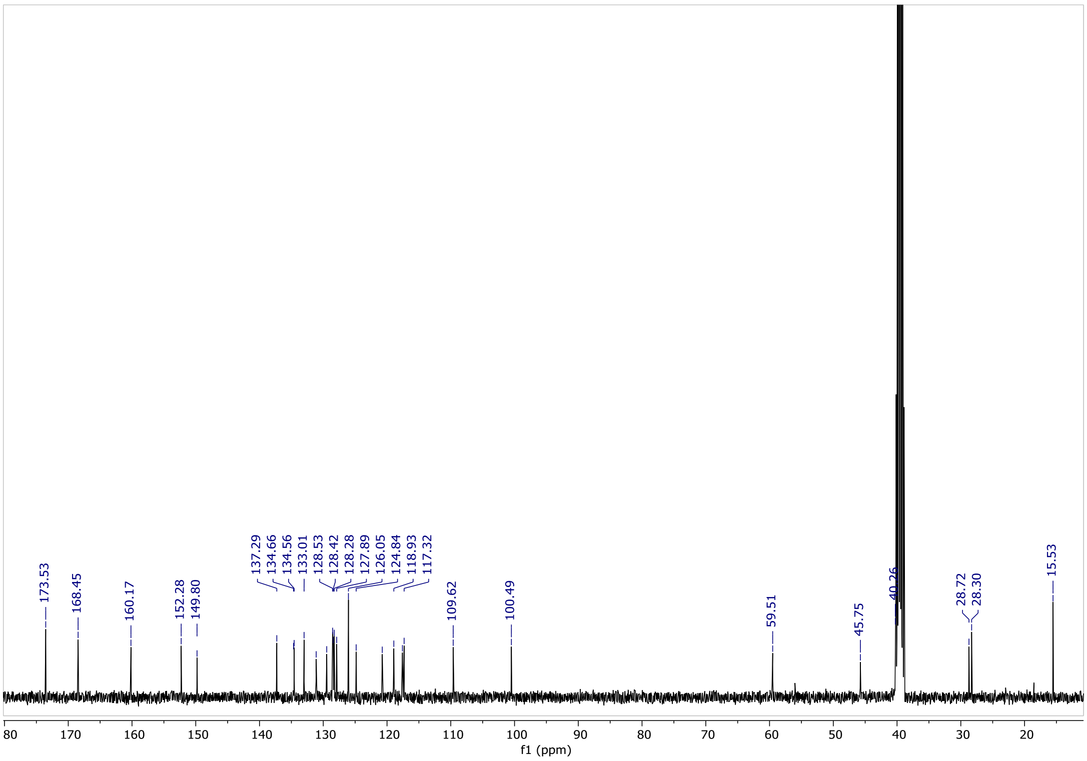

HPLC-MS analysis of **23d**

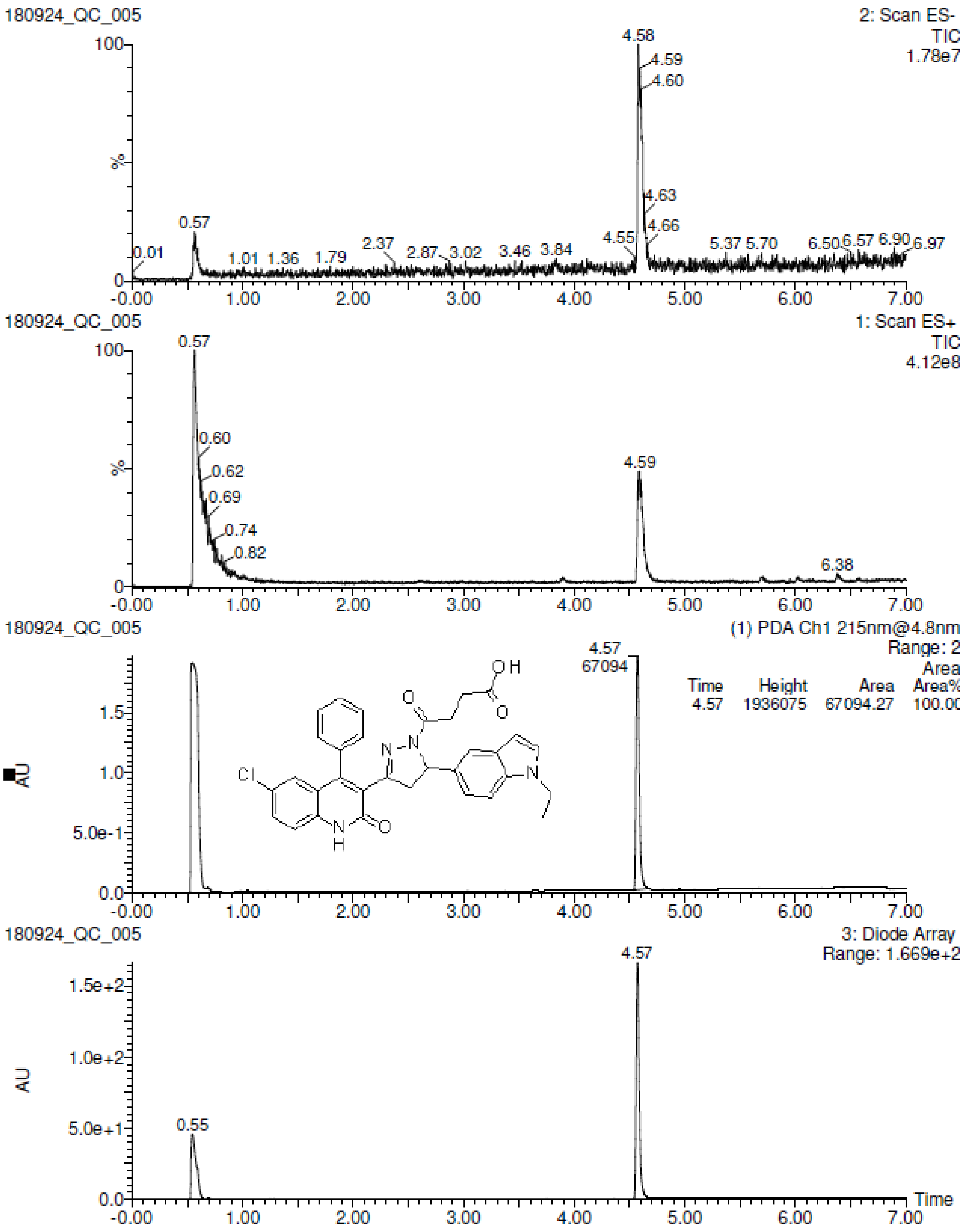

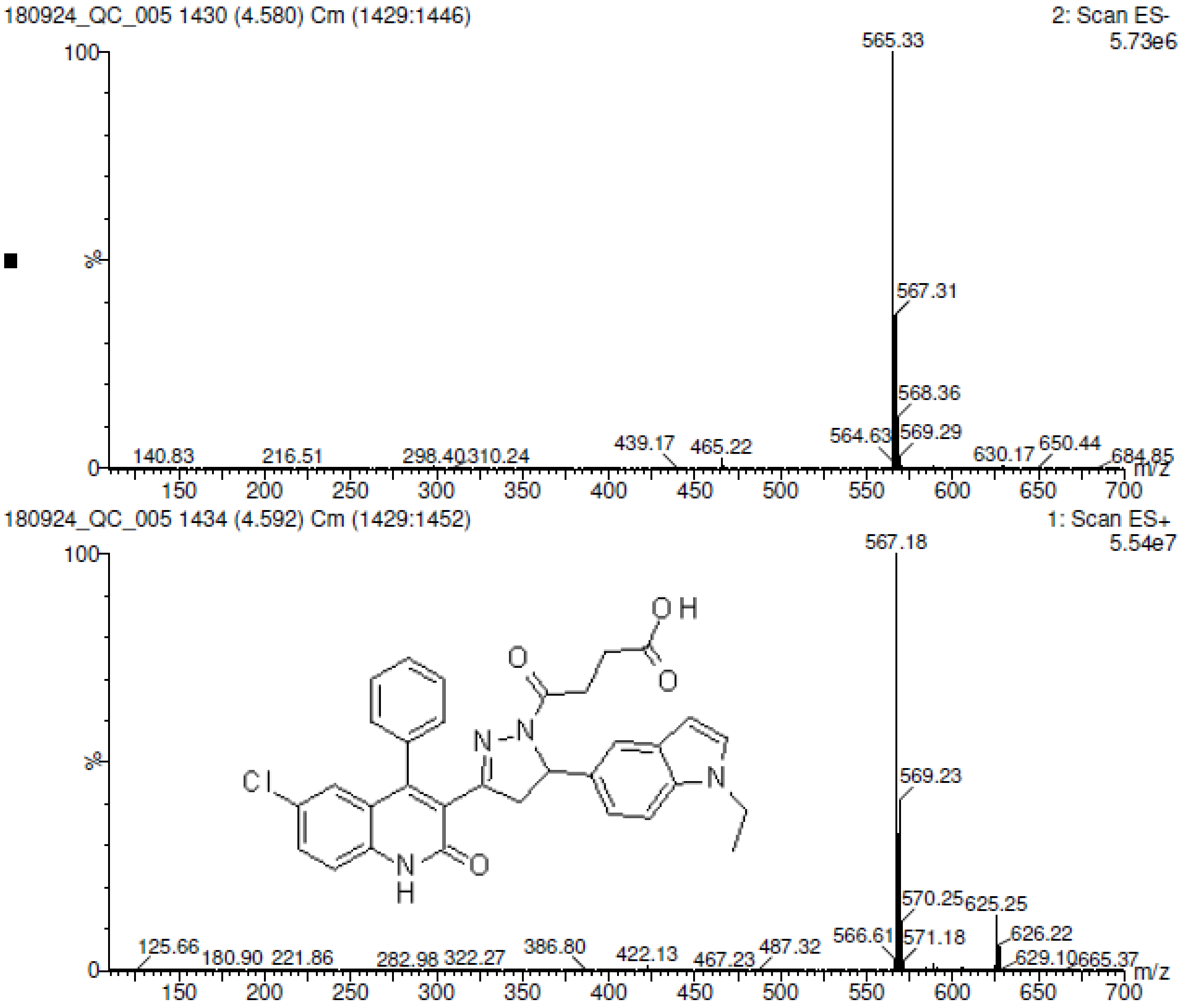
180924_QC_005 1430 (4.580) Cm (1429:1446)
2: Scan ES-
5.73e6
565.33
567.31
568.36
569.29
564.63
140.83
216.51
298.40
310.24
439.17
465.22
630.17
650.44
684.85
m/z
%
180924_QC_005 1434 (4.592) Cm (1429:1452)
1: Scan ES+
5.54e7
567.18
569.23
570.25
625.25
626.22
571.18
566.61
629.10
665.37
125.66
180.90
221.86
282.98
322.27
386.80
422.13
467.23
487.32
Cl
N
H
O
OH

^{1}H-NMR spectrum (400 MHz, DMSO-d_6) of **24d**

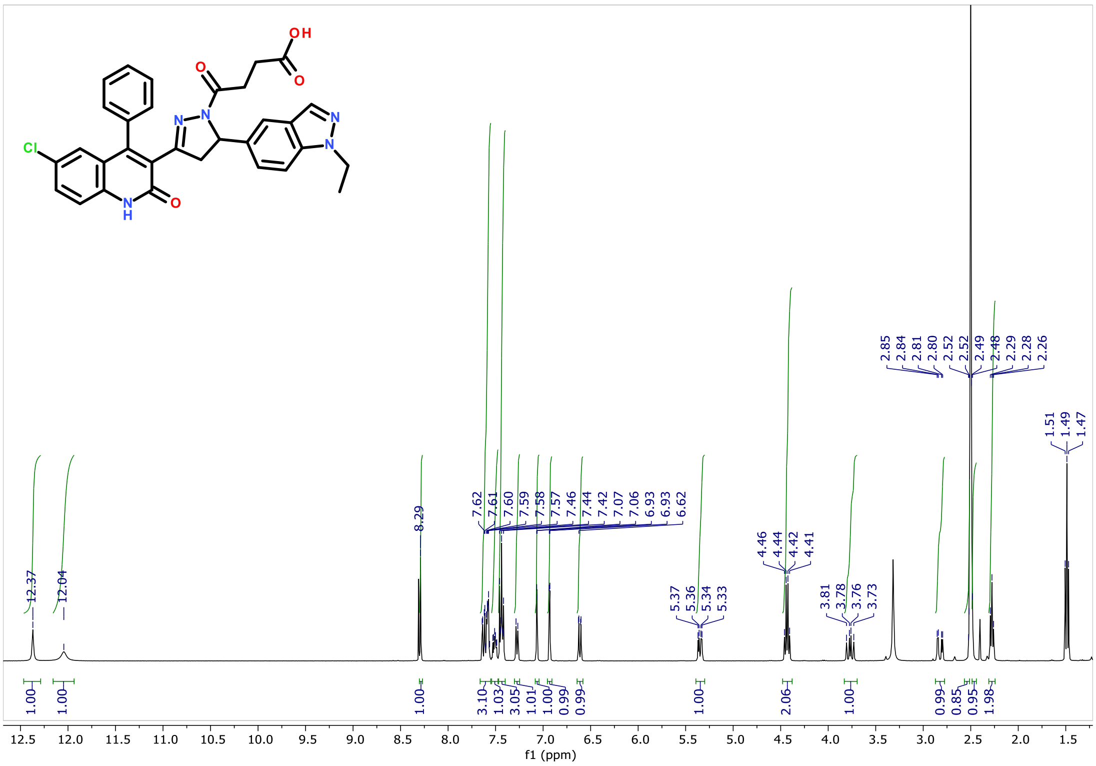

^{13}C-NMR spectrum (101 MHz, DMSO-d_6) of **24d**

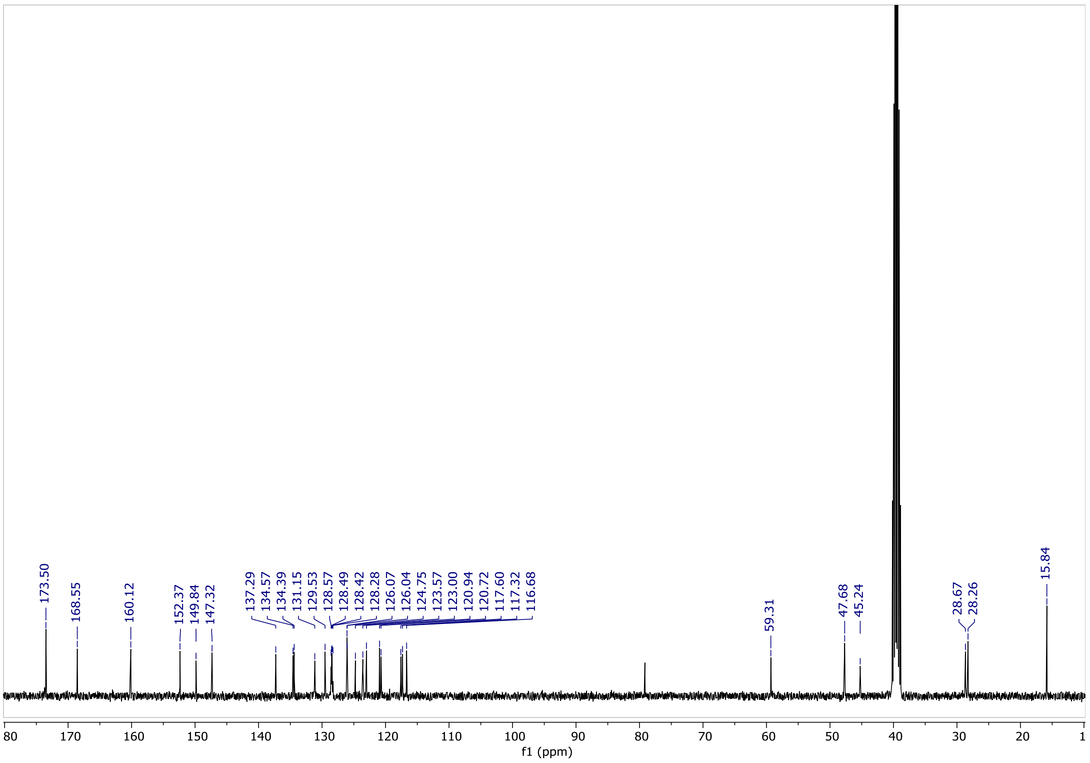

HPLC-MS analysis of **24d**

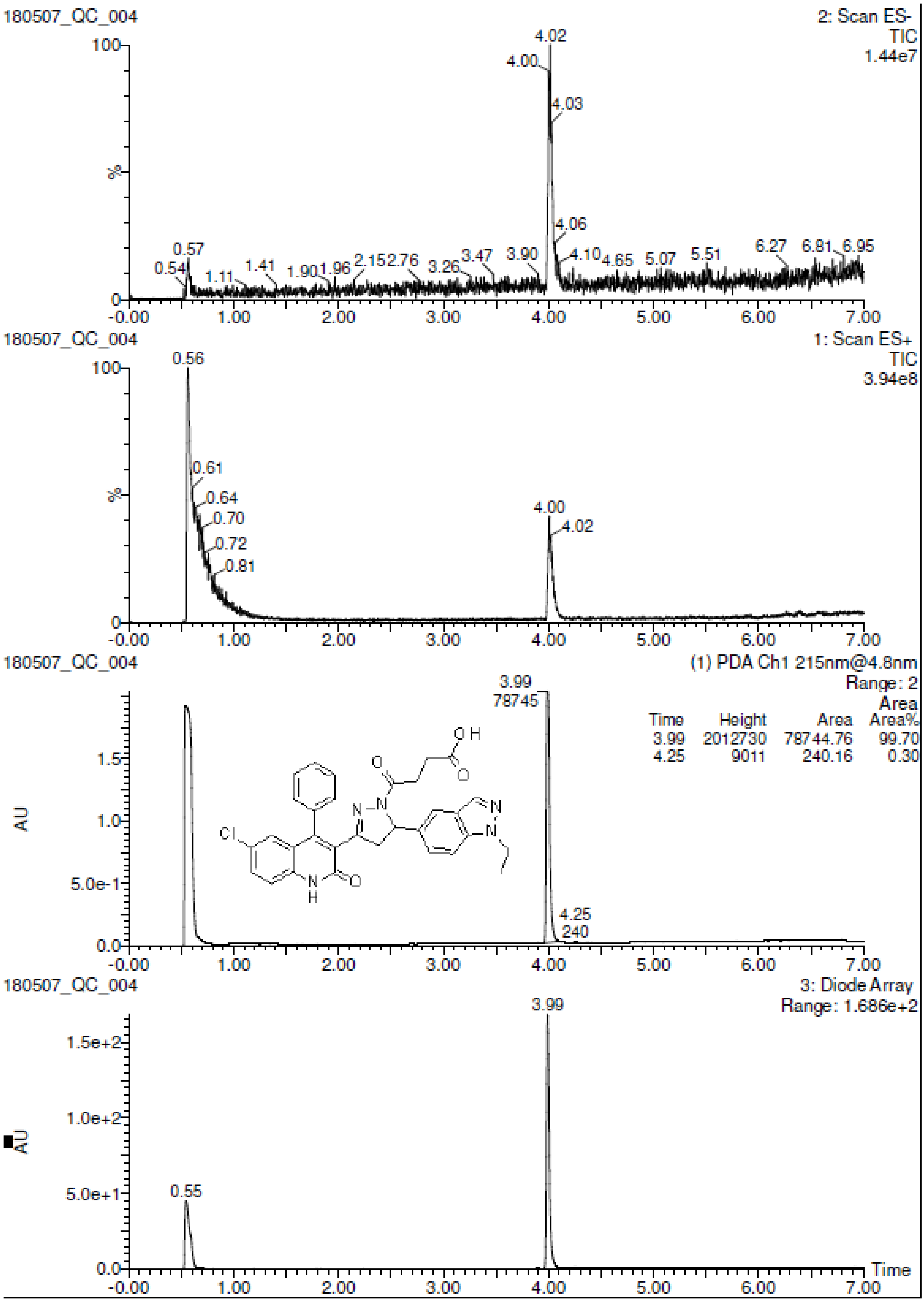

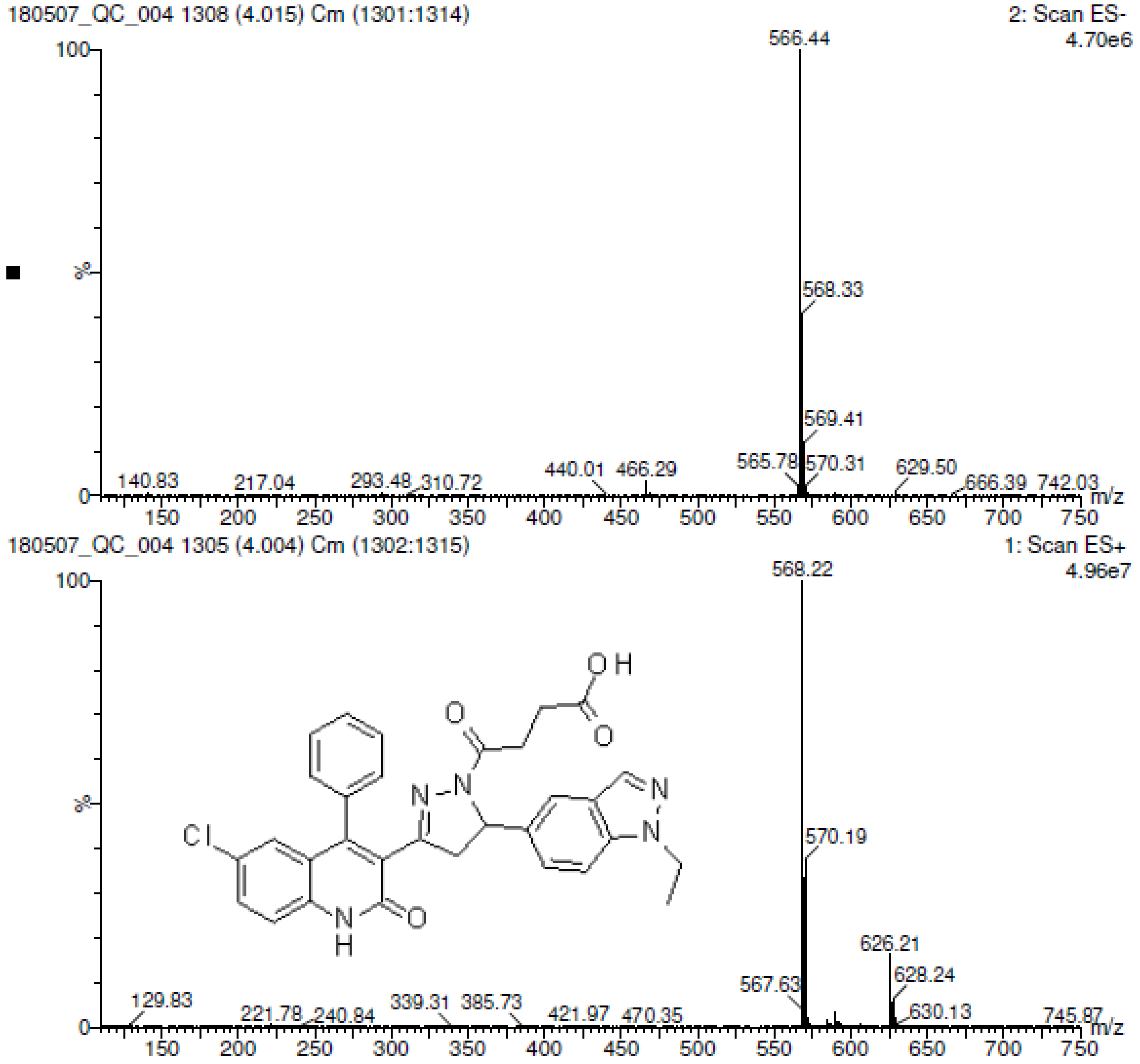

180507_QC_004 1308 (4.015) Cm (1301:1314)
2: Scan ES-
4.70e6
566.44
568.33
569.41
140.83
217.04
293.48
310.72
440.01
466.29
565.78
570.31
629.50
666.39
742.03
m/z
%
180507_QC_004 1305 (4.004) Cm (1302:1315)
1: Scan ES+
4.96e7
568.22
570.19
626.21
628.24
567.63
630.13
129.83
221.78
240.84
339.31
385.73
421.97
470.35
745.87

^{1}H-NMR spectrum (400 MHz, DMSO-d_6) of **25d**

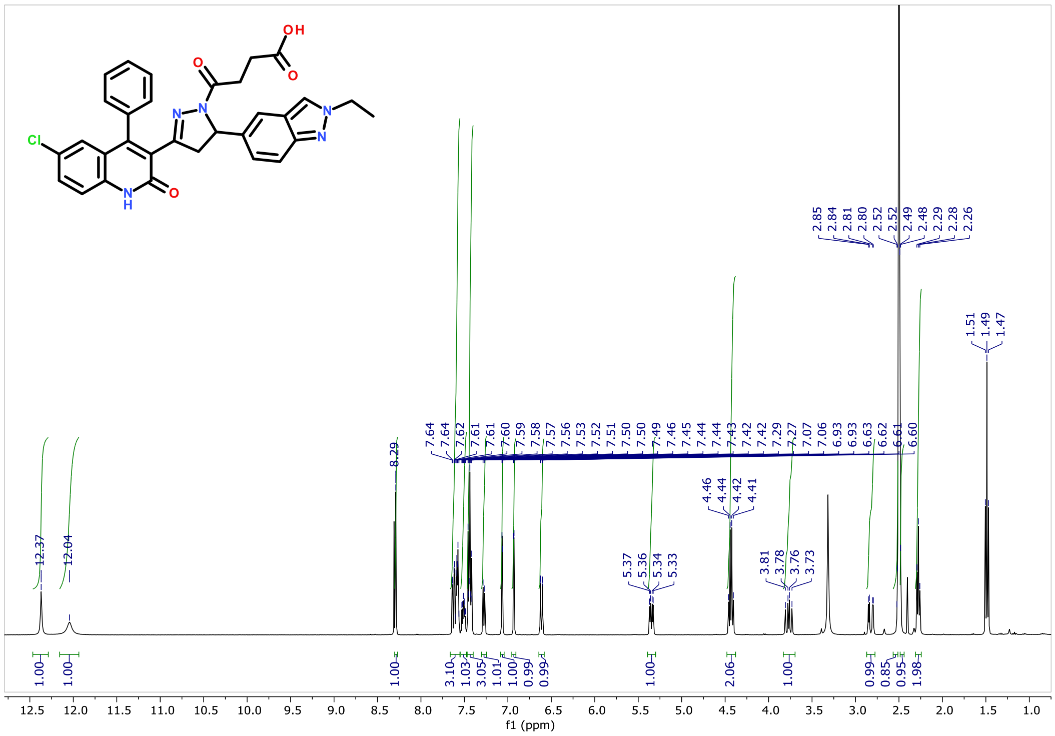

^{13}C-NMR spectrum (101 MHz, DMSO-d_6) of **25d**

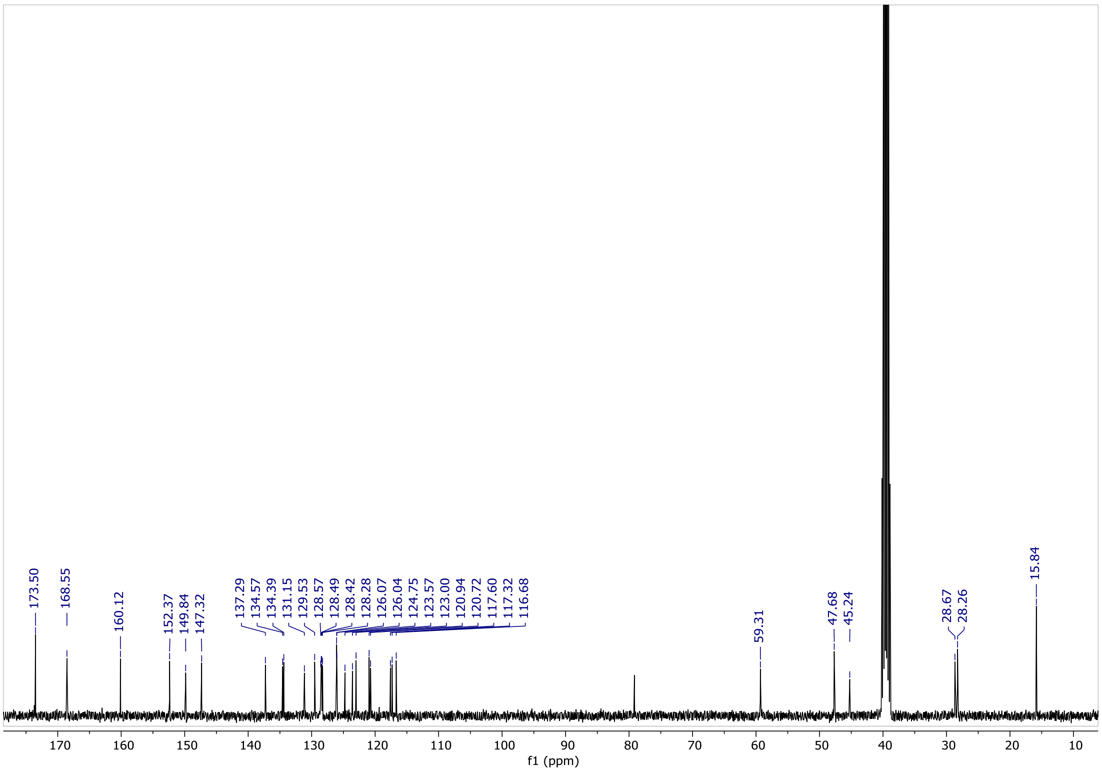

HPLC-MS analysis of **25d**

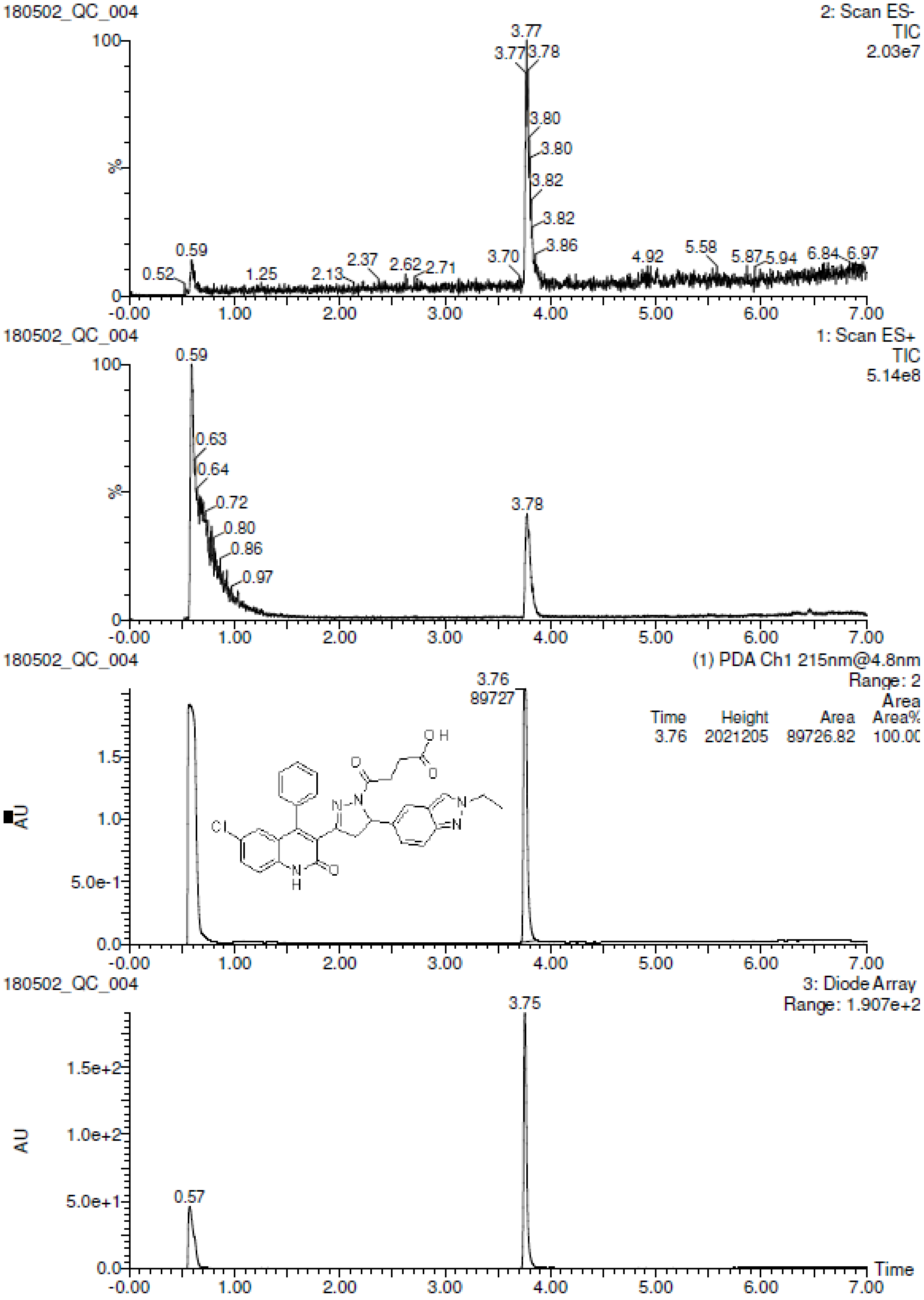

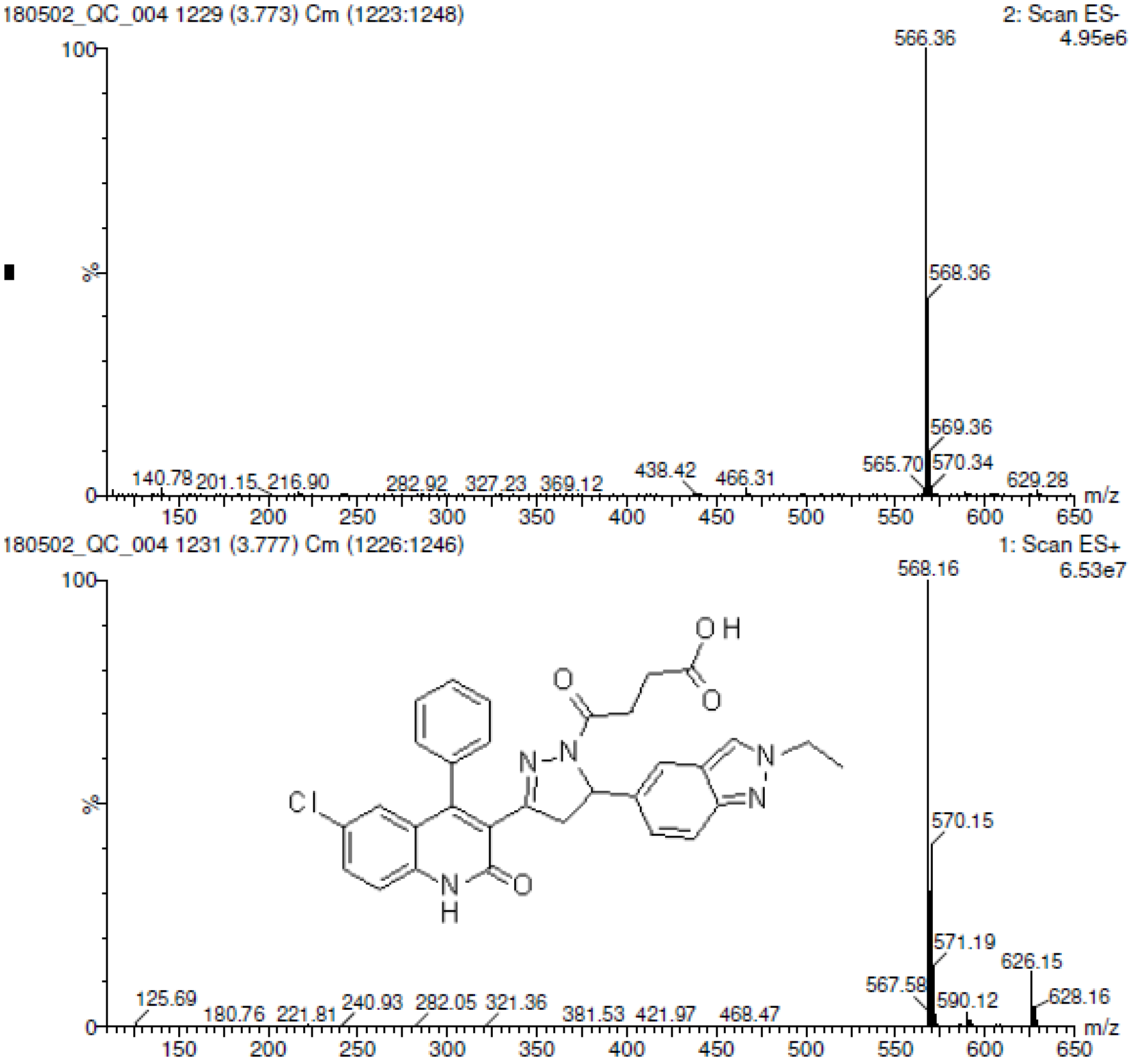
180502_QC_004 1229 (3.773) Cm (1223:1248)
2: Scan ES-
4.95e6
566.36
568.36
569.36
565.70
570.34
629.28
140.78
201.15
216.90
282.92
327.23
369.12
438.42
466.31
100
%
0
150
200
250
300
350
400
450
500
550
600
650
m/z
180502_QC_004 1231 (3.777) Cm (1226:1246)
1: Scan ES+
6.53e7
568.16
570.15
571.19
626.15
628.16
567.58
590.12
125.69
180.76
221.81
240.93
282.05
321.36
381.53
421.97
468.47
100
%
0
150
200
250
300
350
400
450
500
550
600
650
m/z
OH
O
O
N-N
N
N
Cl
N
H
O

^{1}H-NMR spectrum (400 MHz, DMSO-d_6) of **26d**

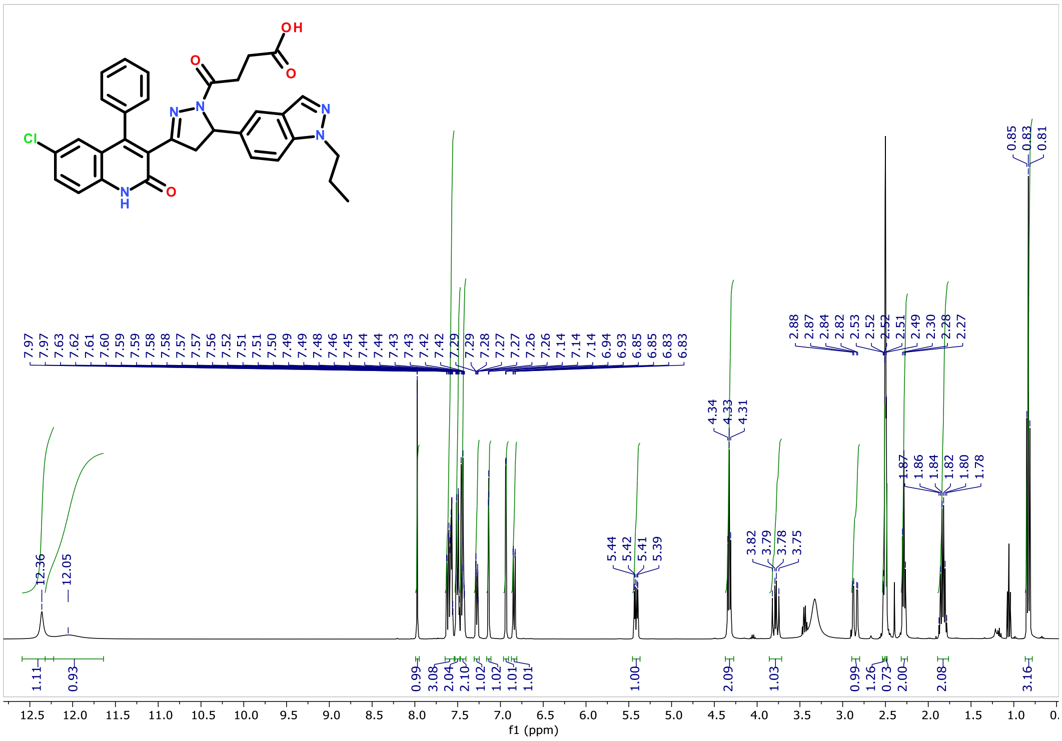

^{13}C-NMR spectrum (101 MHz, DMSO-d_6) of **26d**

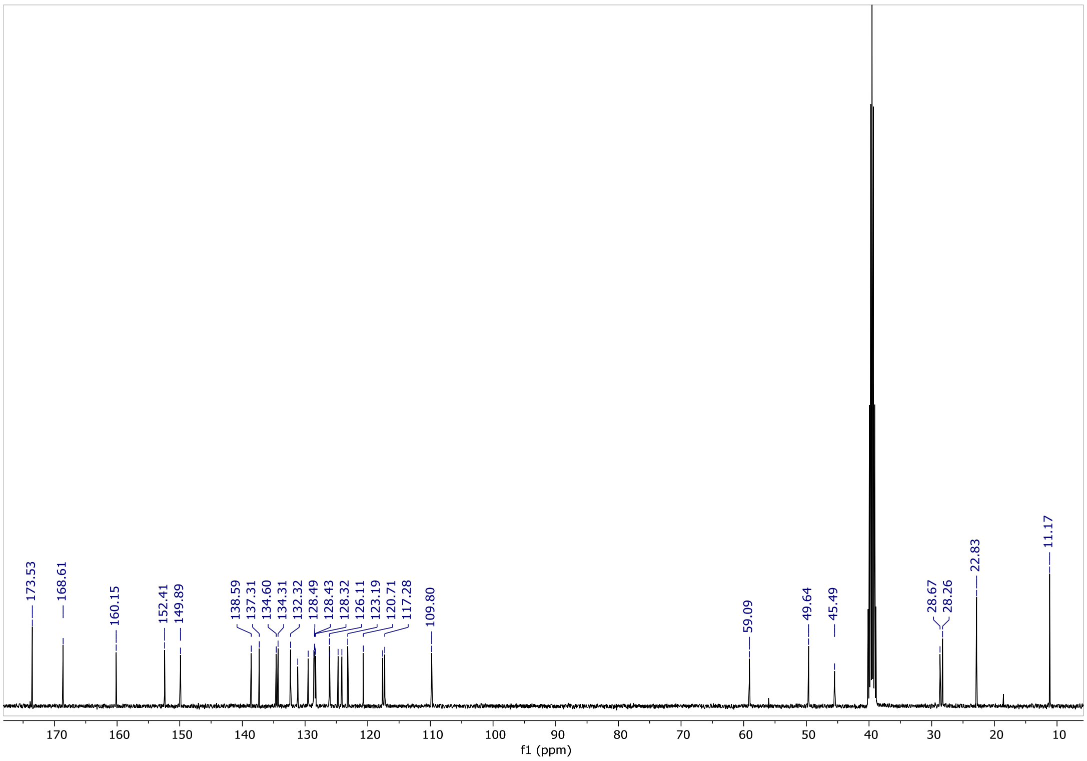

^{1}H-NMR spectrum (400 MHz, DMSO-d_6) of **27d**

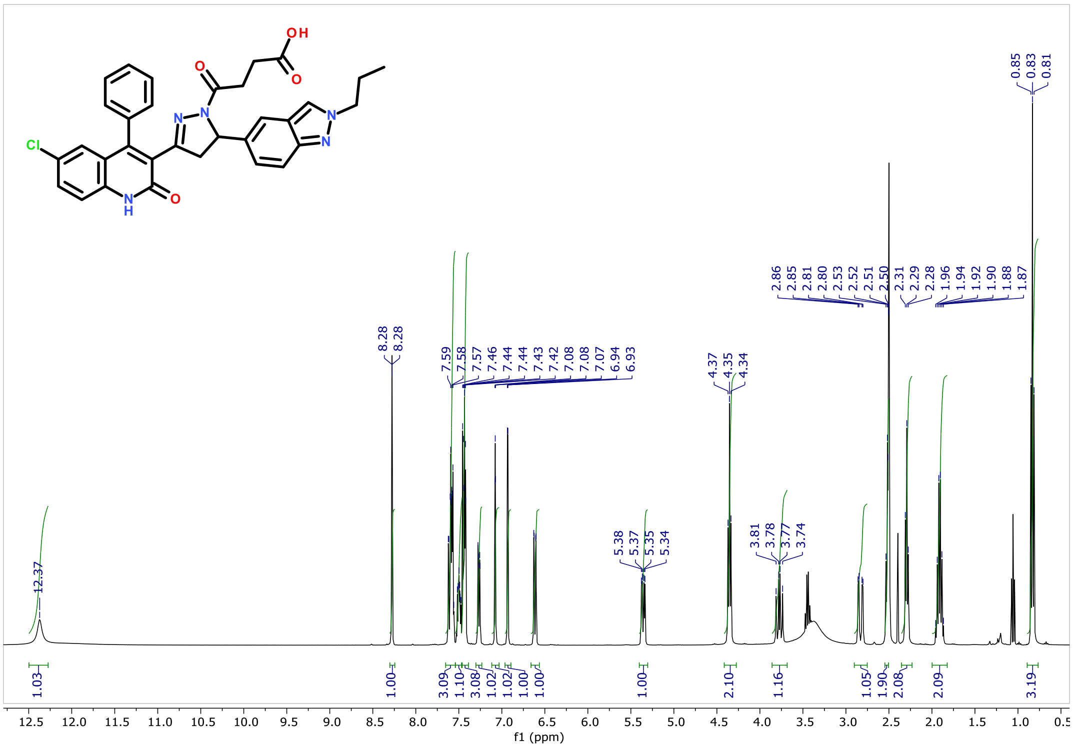

^{13}C-NMR spectrum (101 MHz, DMSO-d_6) of **27d**

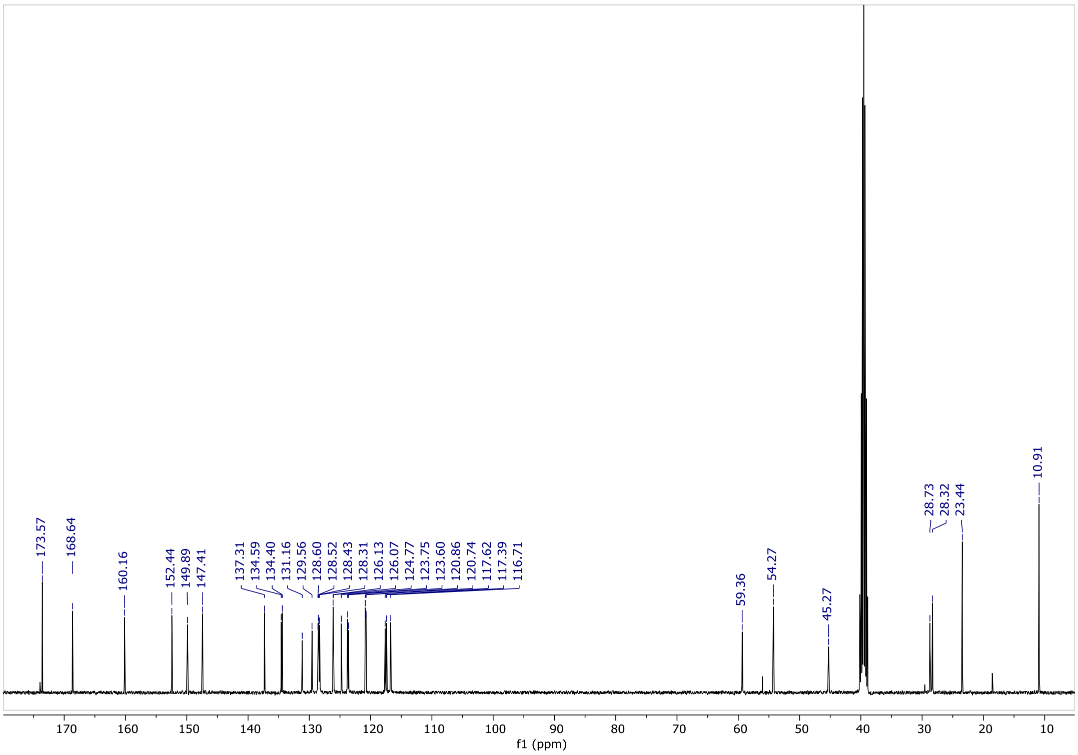

^{1}H-NMR spectrum (400 MHz, DMSO-d_6) of **28d**

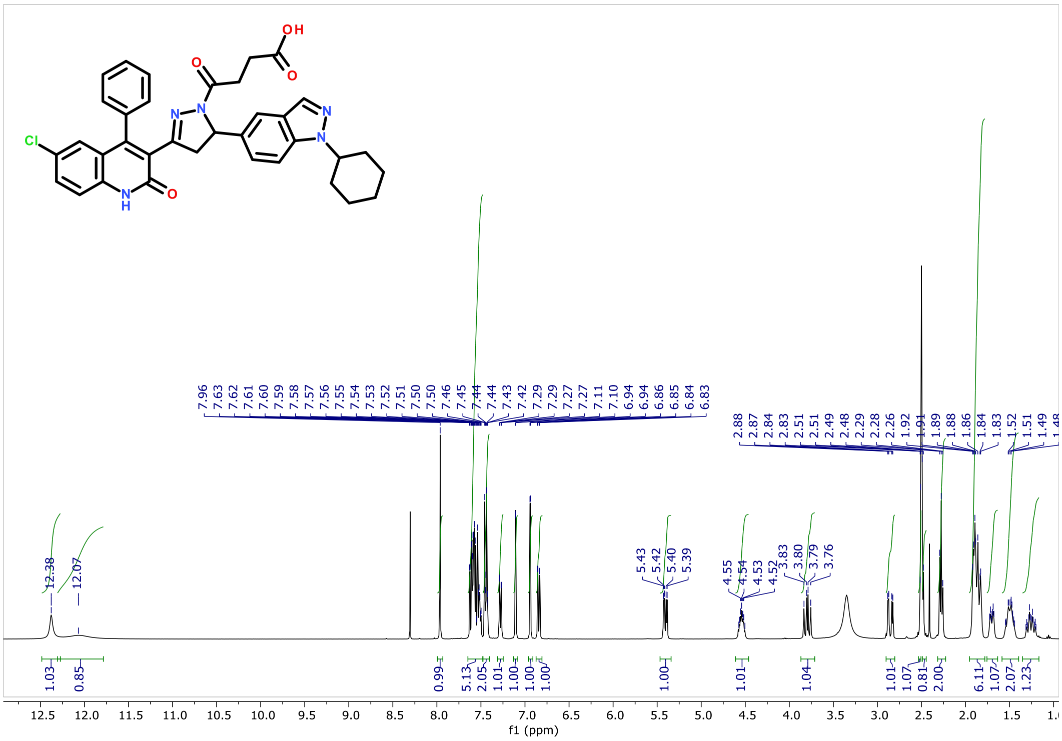

^{13}C-NMR spectrum (101 MHz, DMSO-d_6) of **28d**

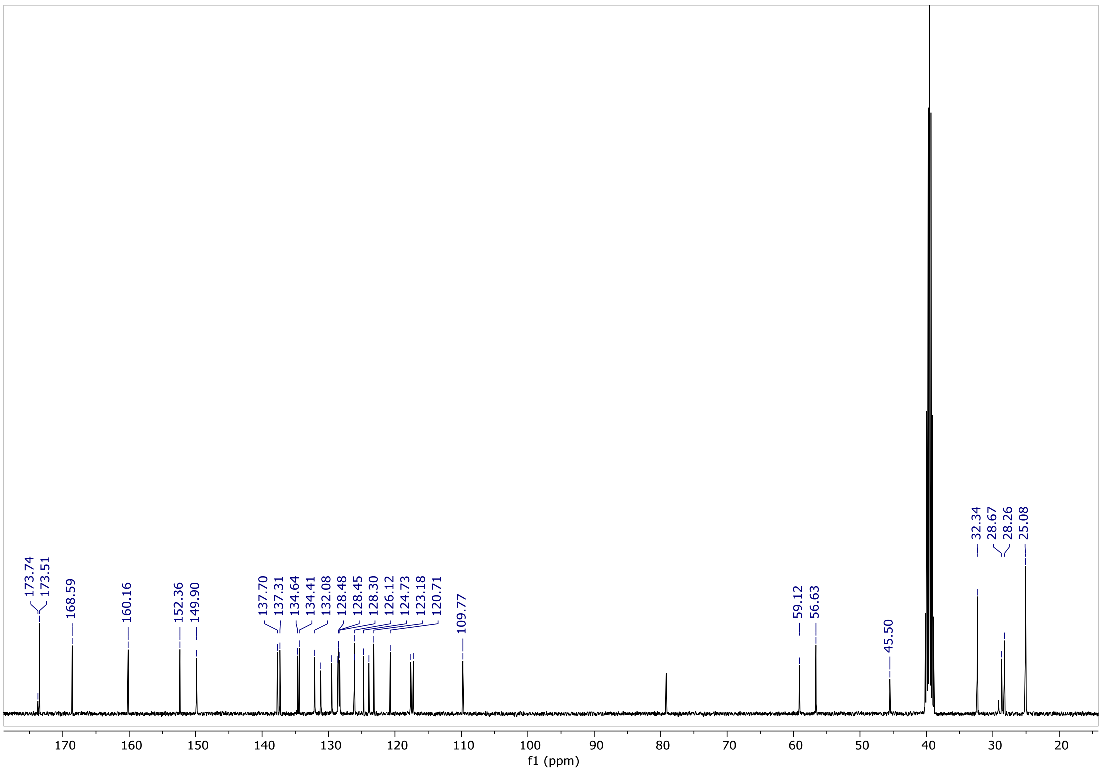

^{1}H-NMR spectrum (400 MHz, DMSO-d_6) of **29d**

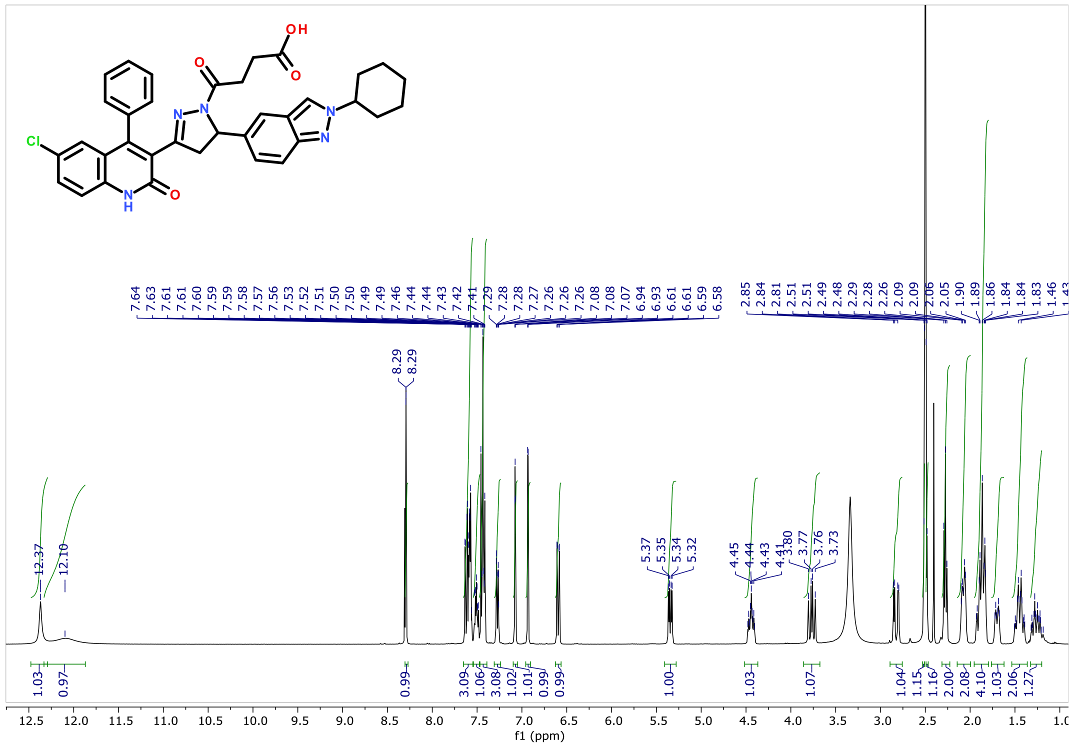

^{13}C-NMR spectrum (101 MHz, DMSO-d_6) of **29d**

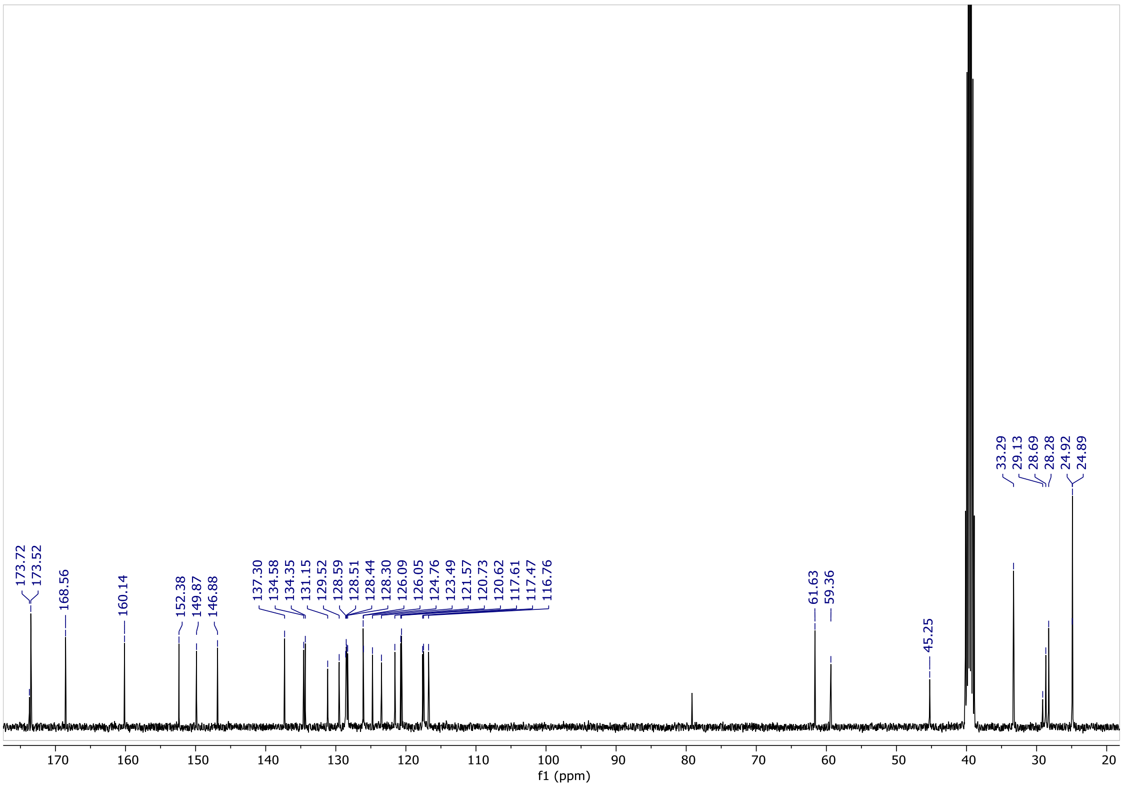

^{1}H-NMR spectrum (400 MHz, DMSO-d_6) of **30d**

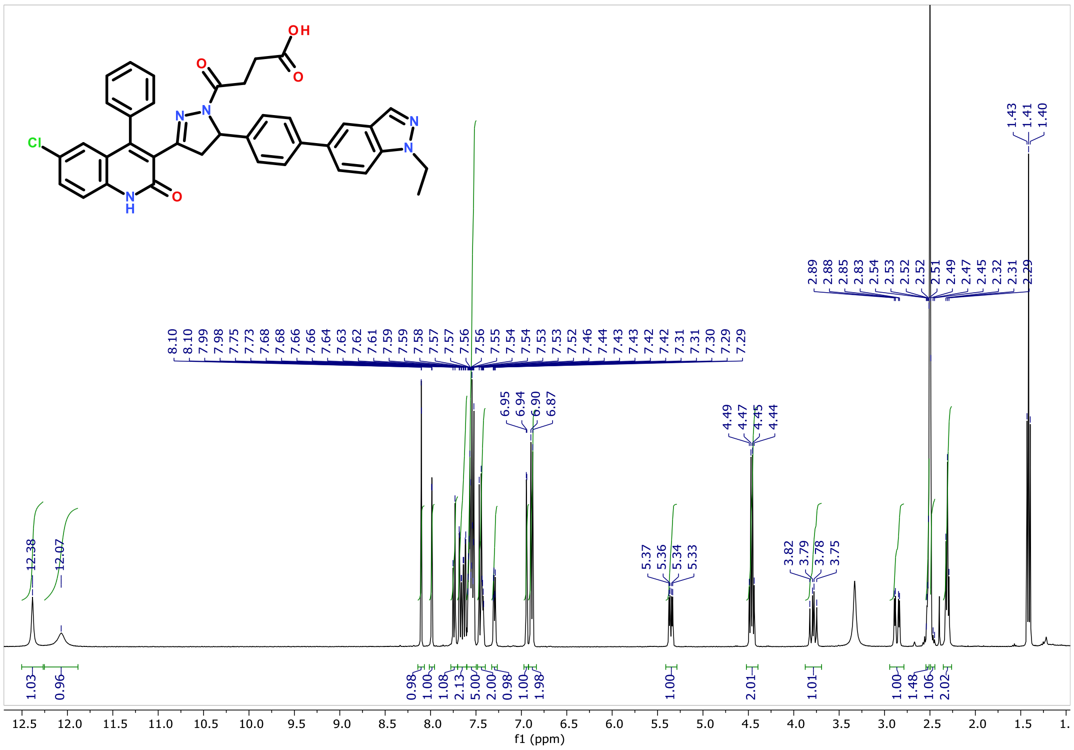

^{13}C-NMR spectrum (101 MHz, DMSO-d_6) of **30d**

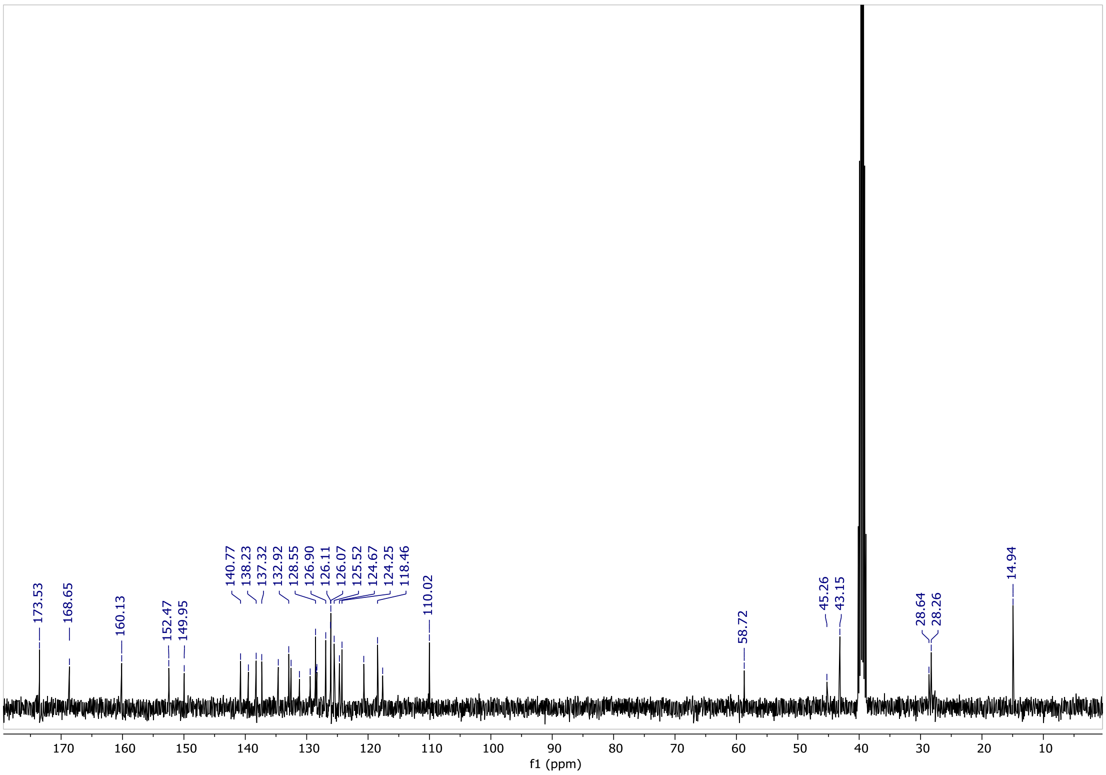

^{1}H-NMR spectrum (400 MHz, DMSO-d_6) of **31d**

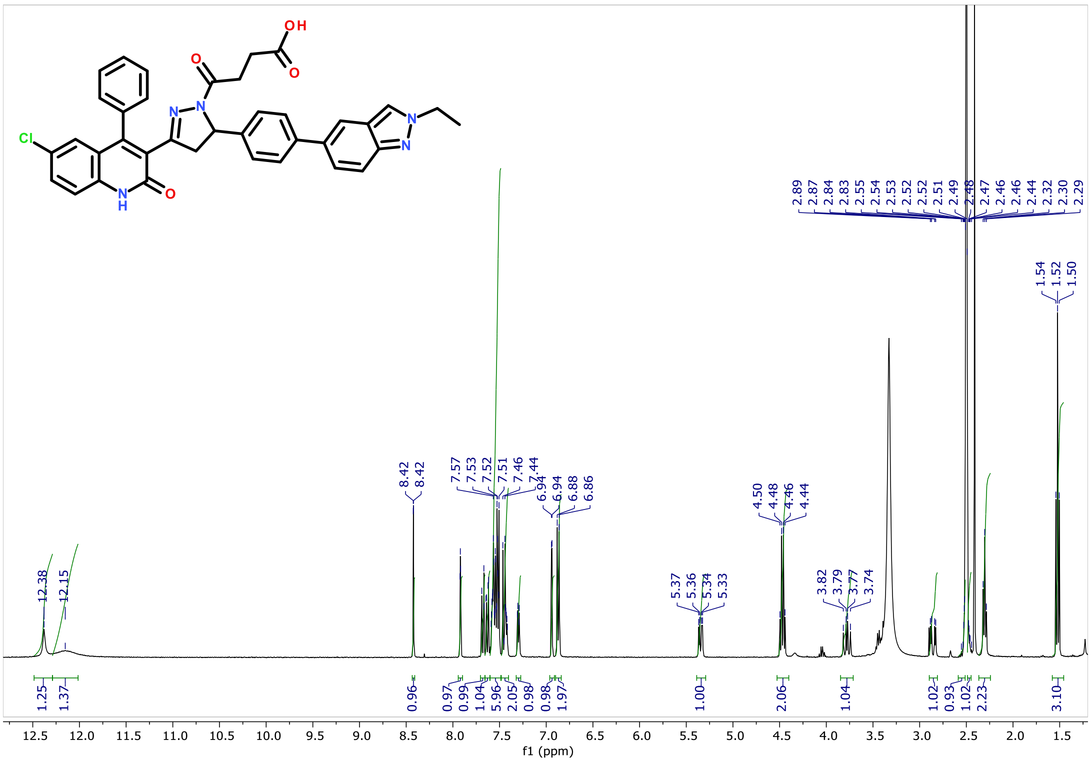

^{13}C-NMR spectrum (101 MHz, DMSO-d_6) of **31d**

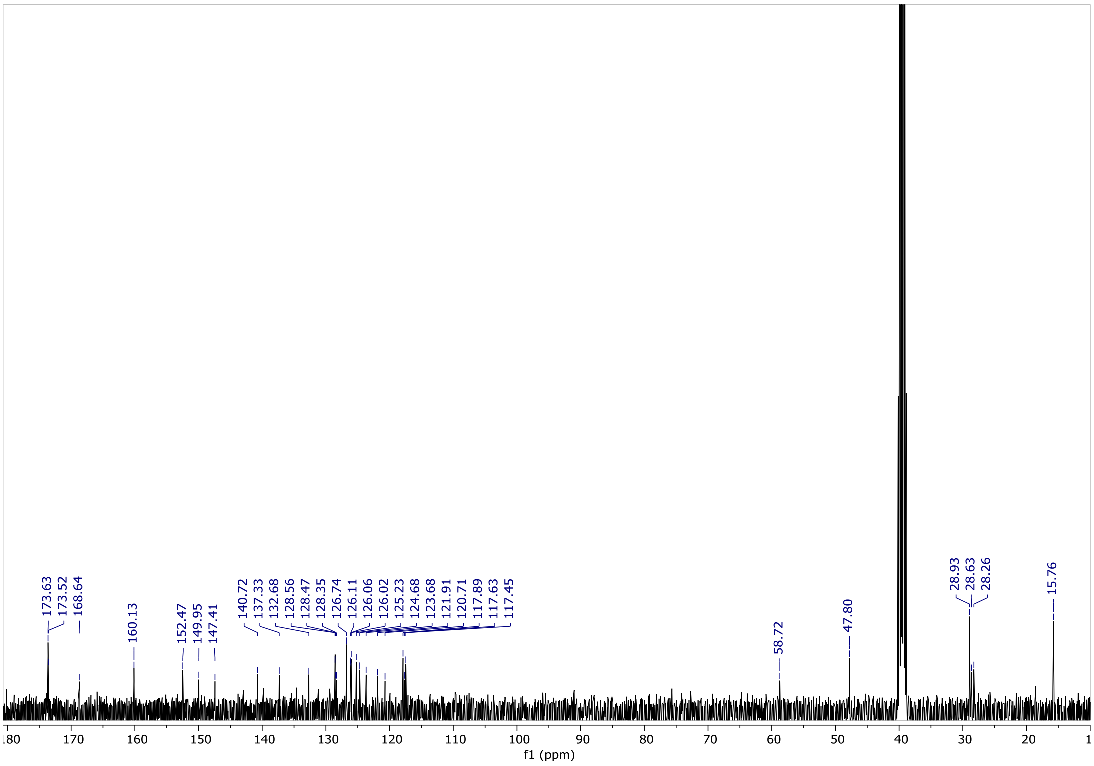

^{1}H-NMR spectrum (400 MHz, DMSO-d_6) of **32d**

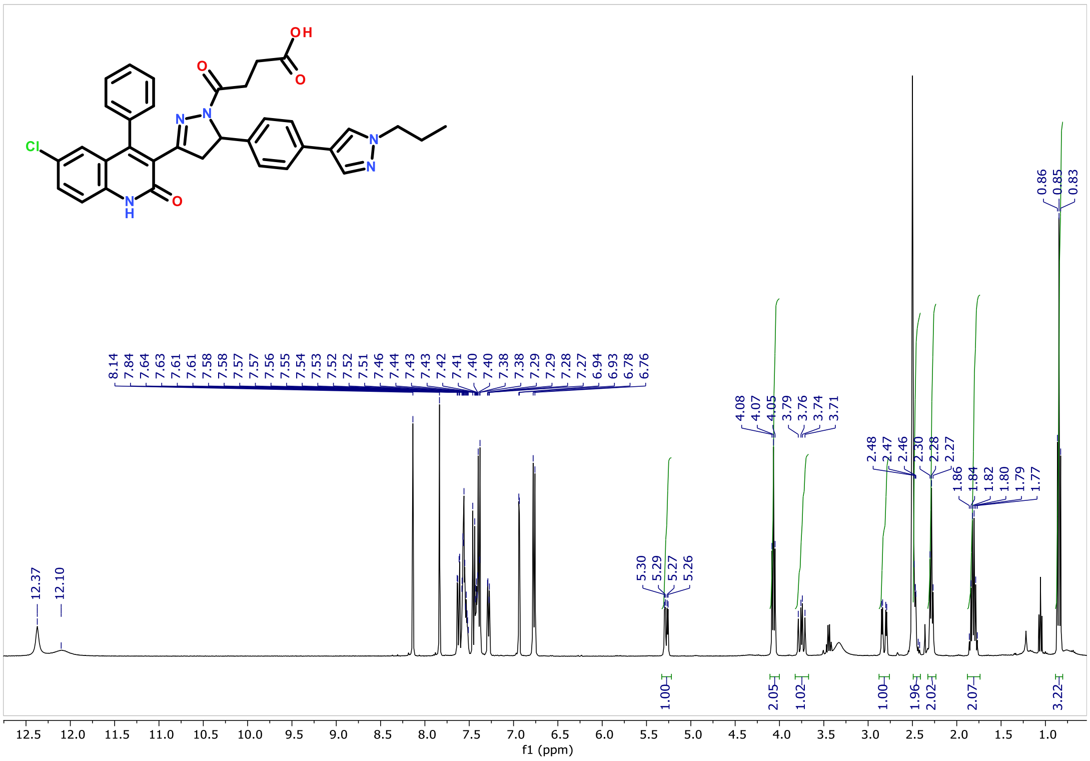

^{13}C-NMR spectrum (101 MHz, DMSO-d_6) of **32d**

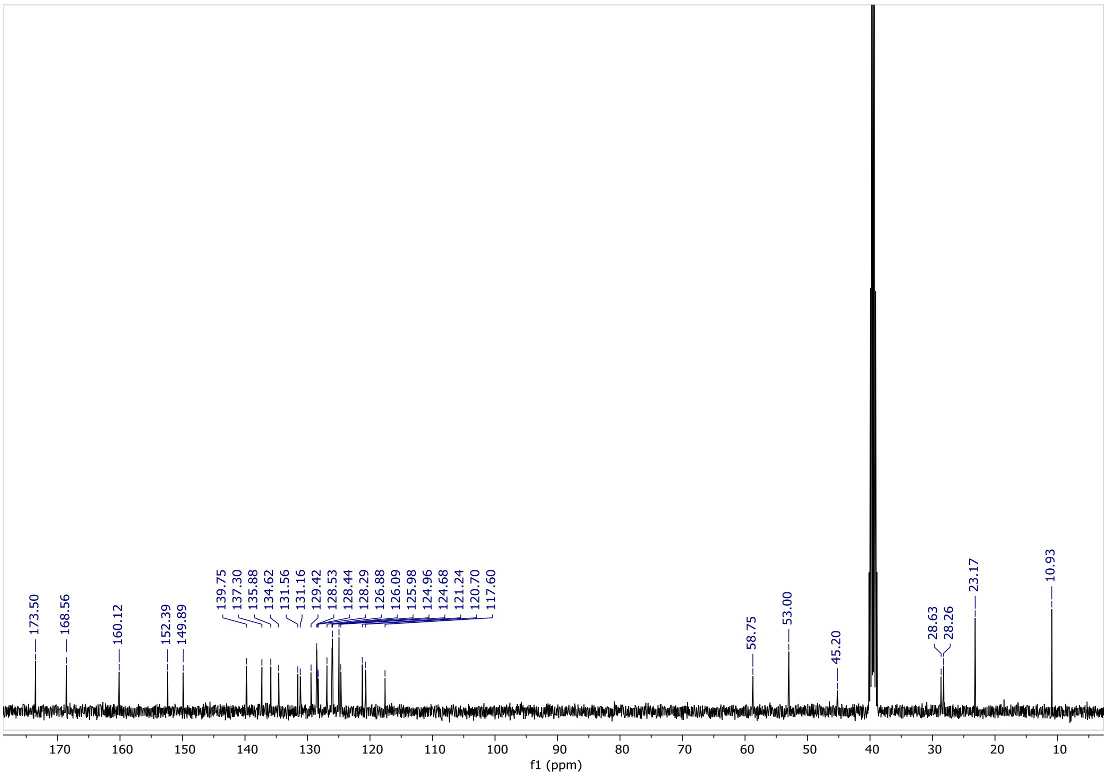

^{1}H-NMR spectrum (400 MHz, DMSO-d_6) of **33d**

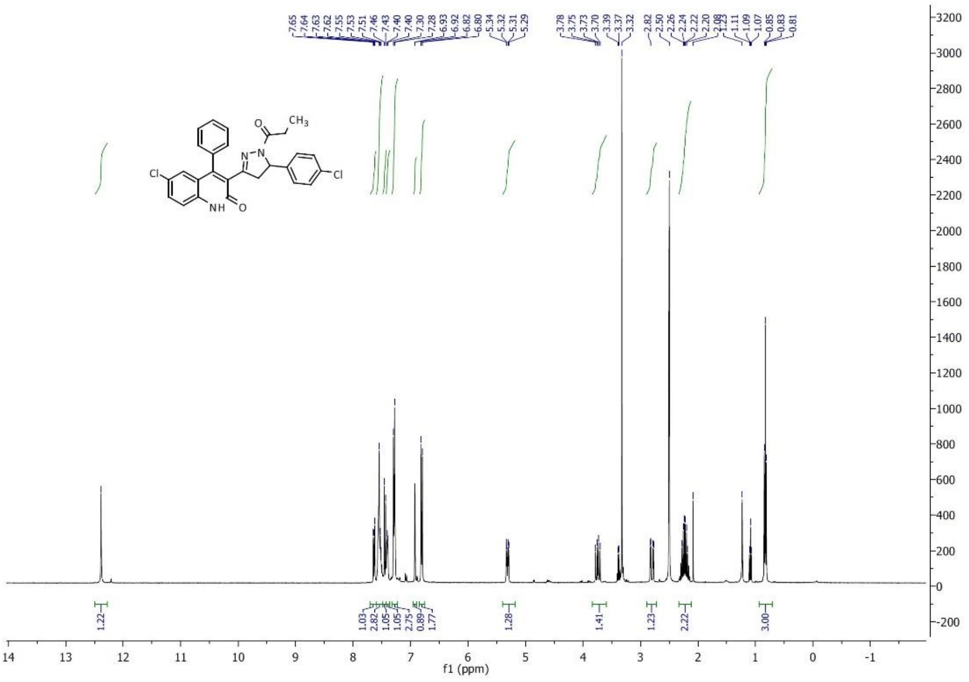

^{13}C-NMR spectrum (101 MHz, DMSO-d_6) of **33d**

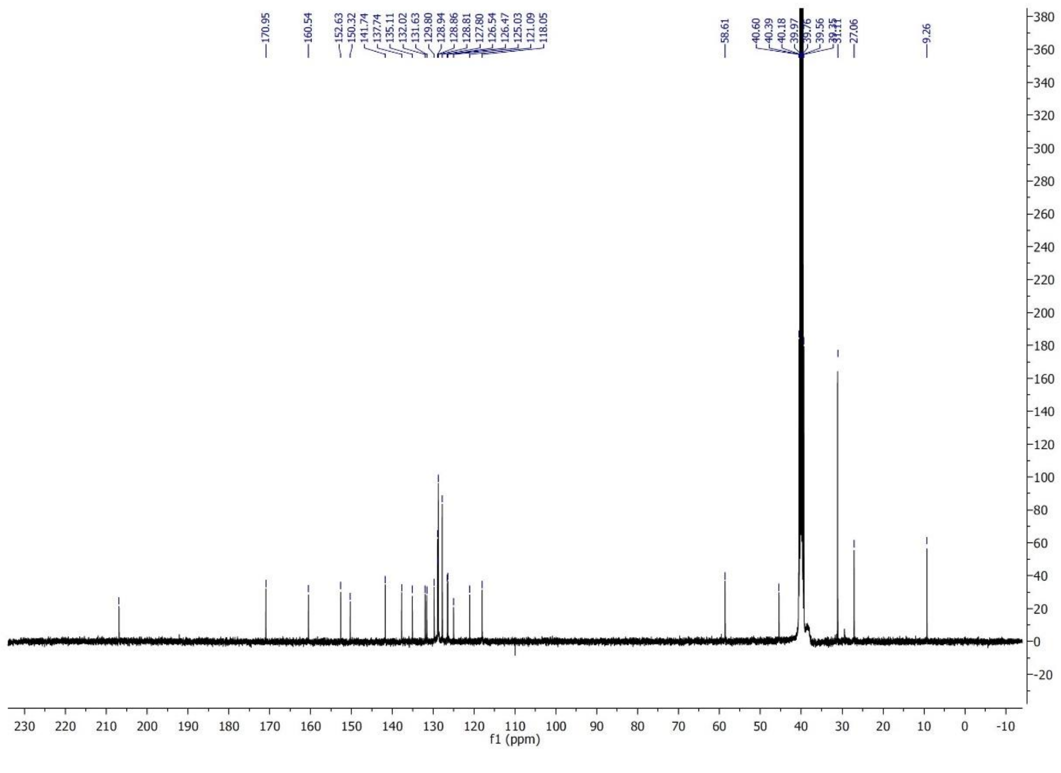

^{1}H-NMR spectrum (400 MHz, DMSO-d_6) of **34d**

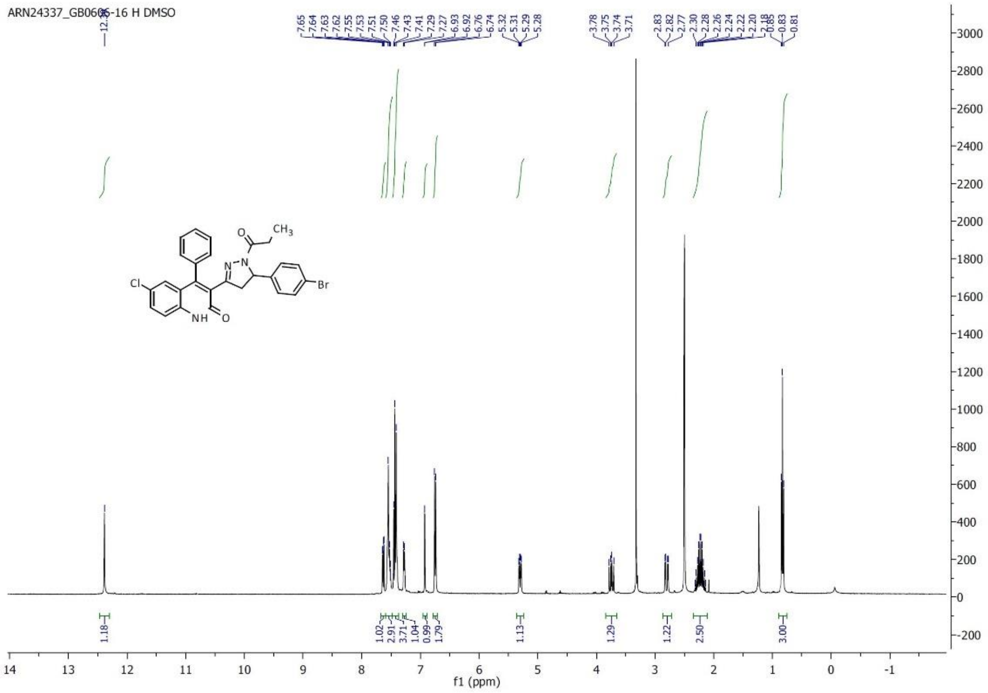

^{13}C-NMR spectrum (101 MHz, DMSO-d_6) of **34d**

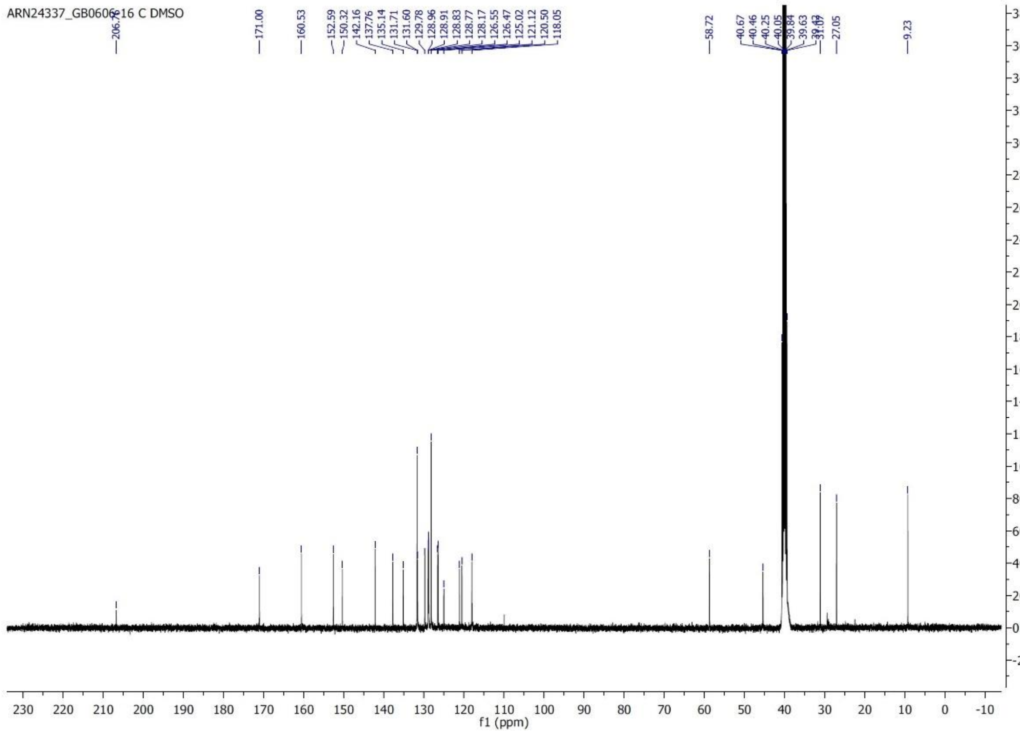

^{1}H-NMR spectrum (400 MHz, DMSO-d_6) of **35d**

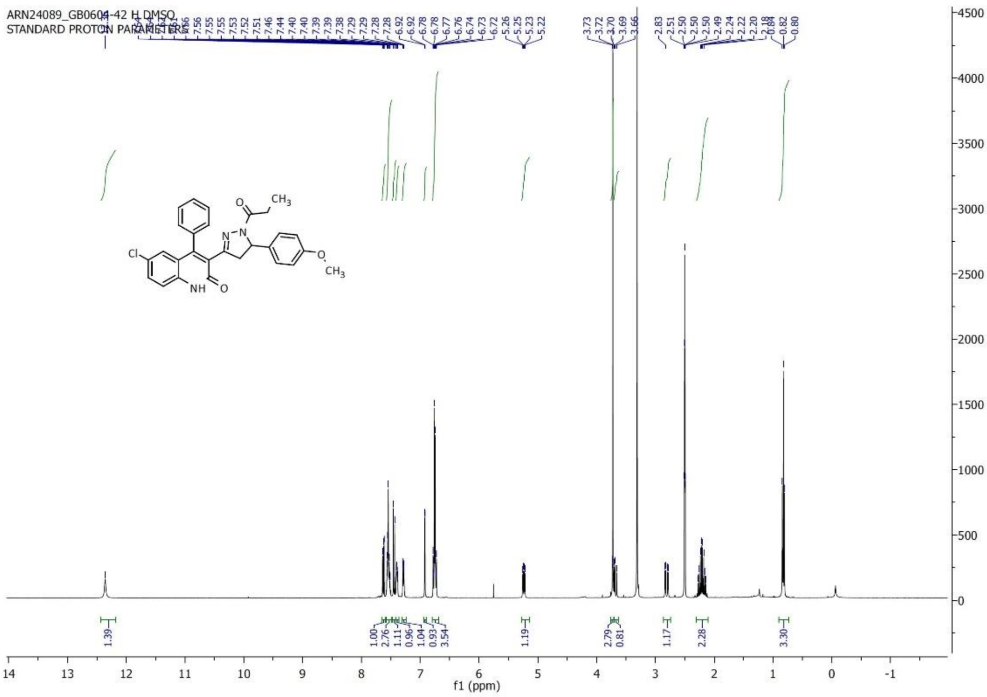

^{13}C-NMR spectrum (101 MHz, DMSO-d_6) of **35d**

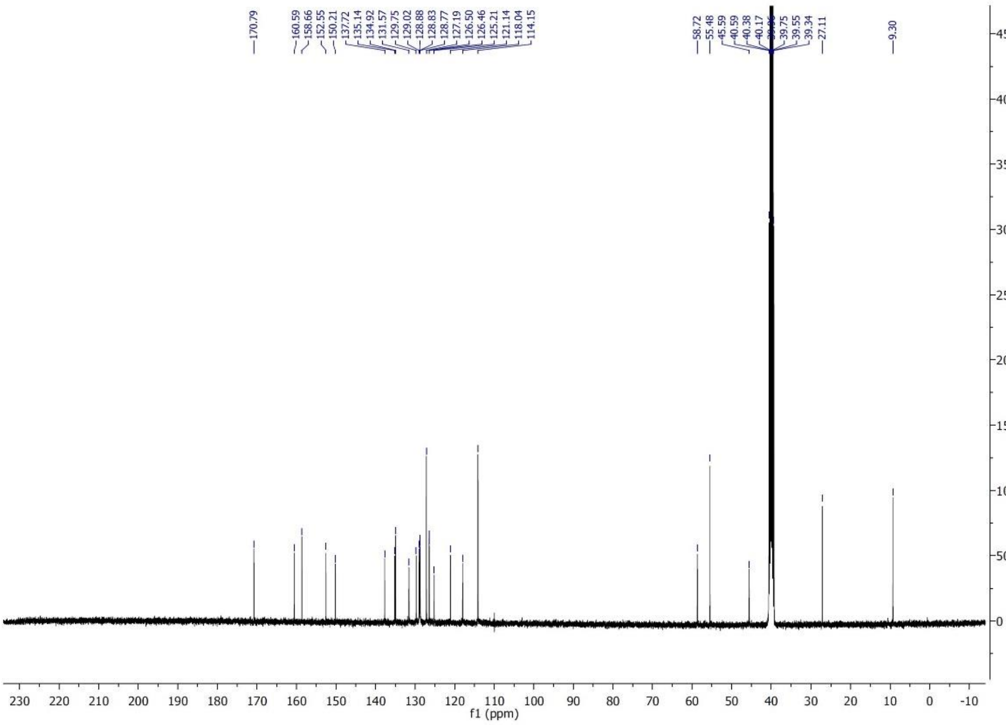

HPLC-MS analysis of **35d**

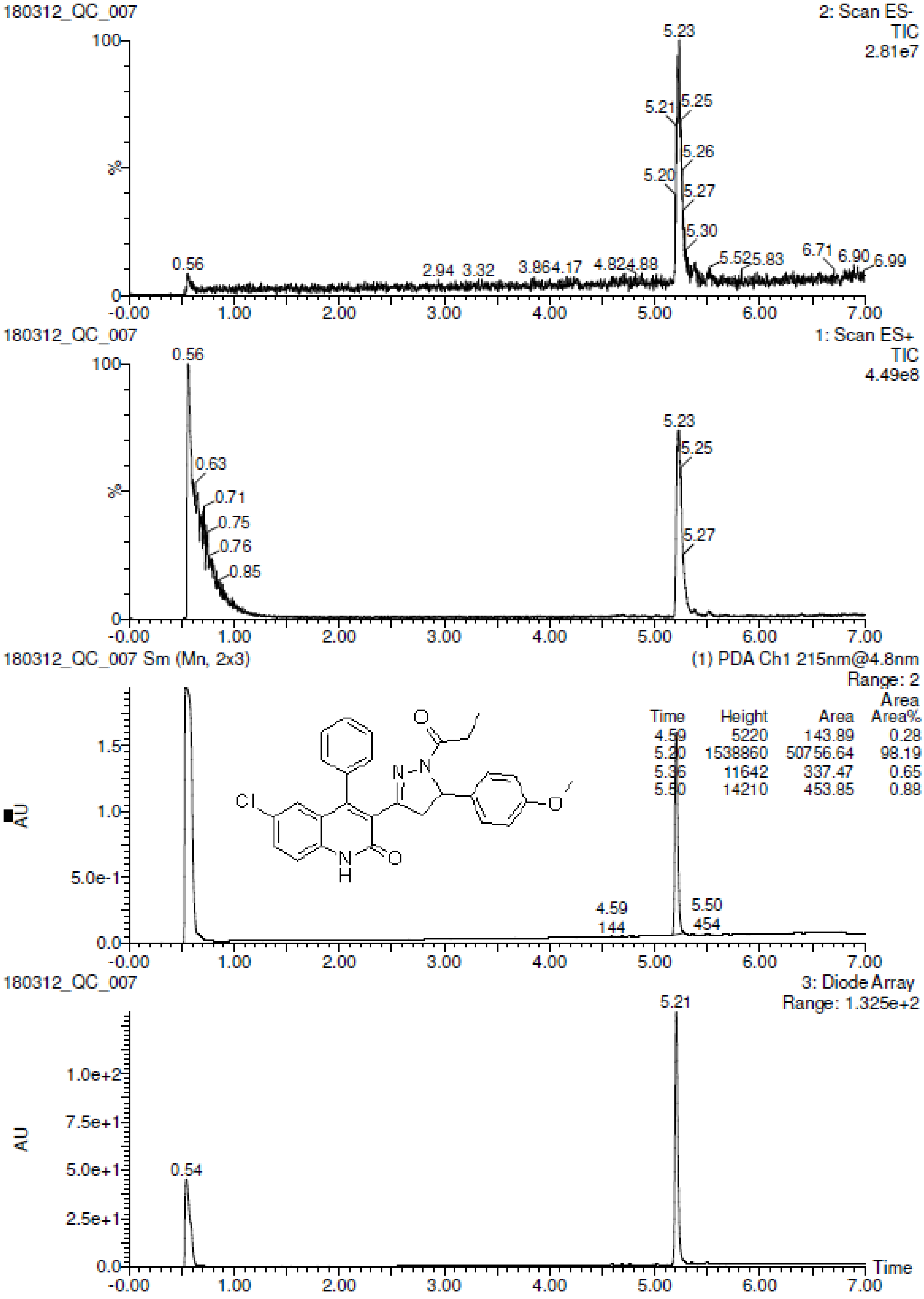

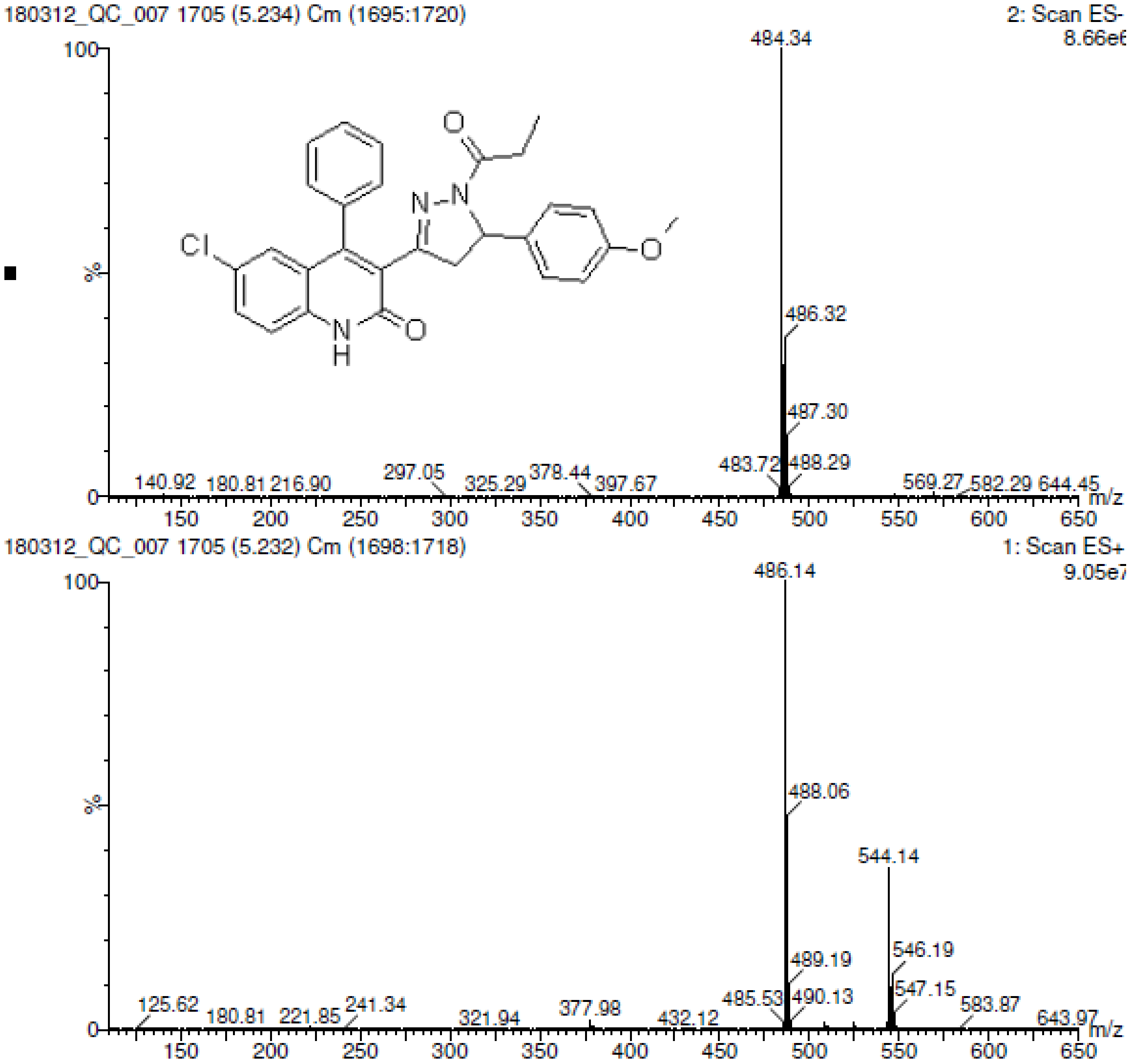

180312_QC_007 1705 (5.234) Cm (1695:1720)
2: Scan ES-
8.66e6
484.34
486.32
487.30
483.72
488.29
140.92
180.81
216.90
297.05
325.29
378.44
397.67
569.27
582.29
644.45
m/z
180312_QC_007 1705 (5.232) Cm (1698:1718)
1: Scan ES+
9.05e7
486.14
488.06
544.14
489.19
546.19
547.15
485.53
490.13
125.62
180.81
221.85
241.34
321.94
377.98
432.12
583.87
643.97
m/z

^{1}H-NMR spectrum (400 MHz, DMSO-d_6) of **36d**

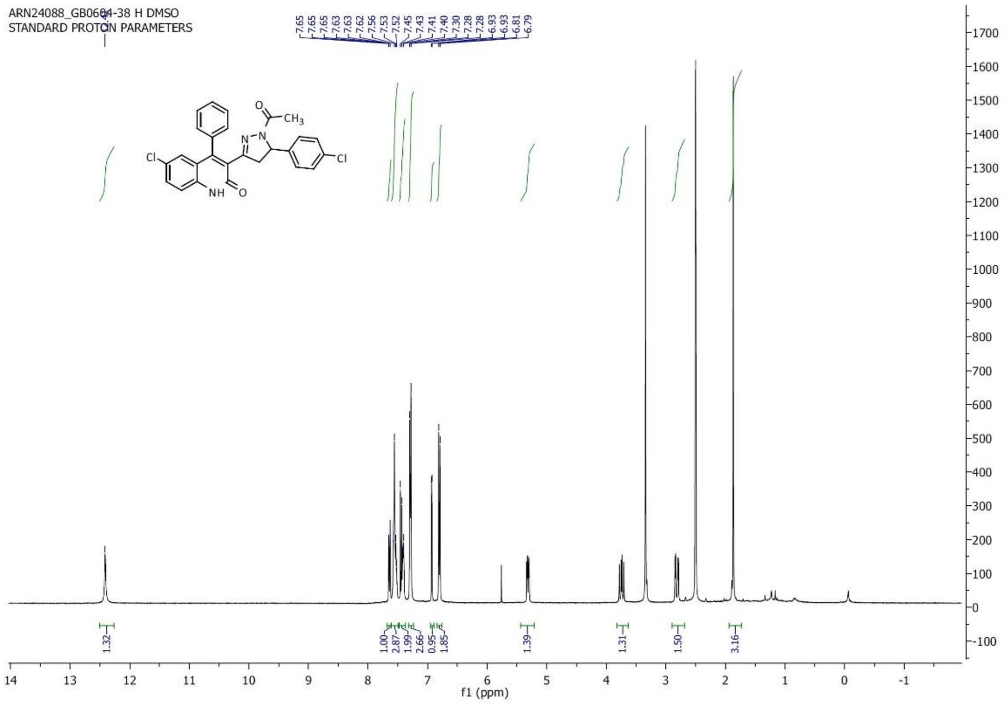

^{13}C-NMR spectrum (101 MHz, DMSO-d_6) of **36d**

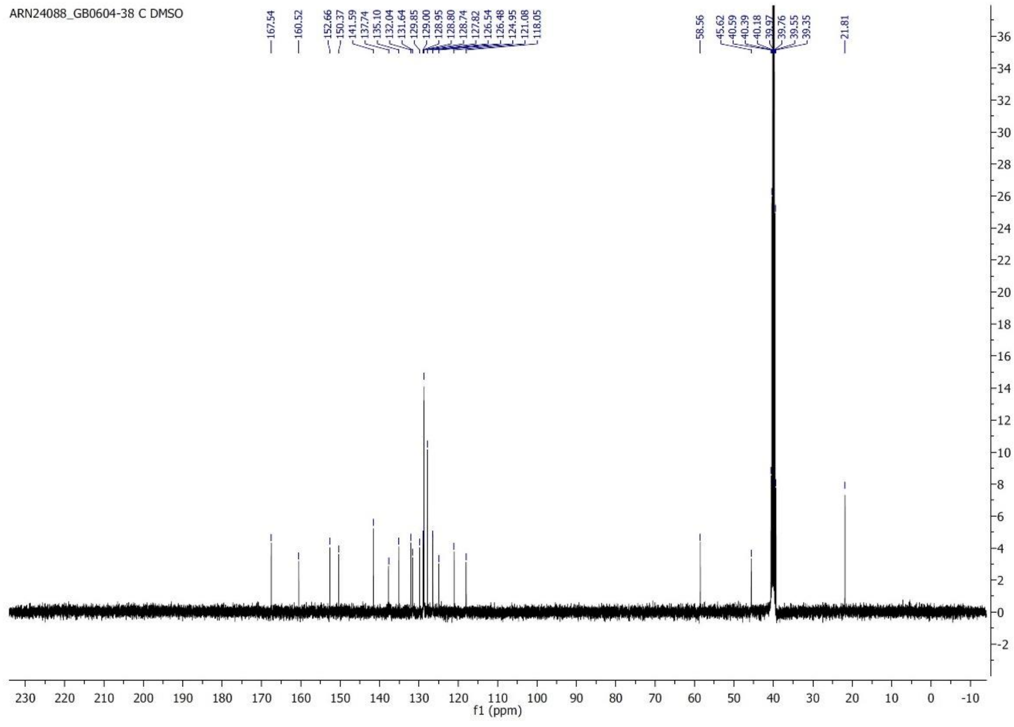

^{1}H-NMR spectrum (400 MHz, DMSO-d_6) of **37d**

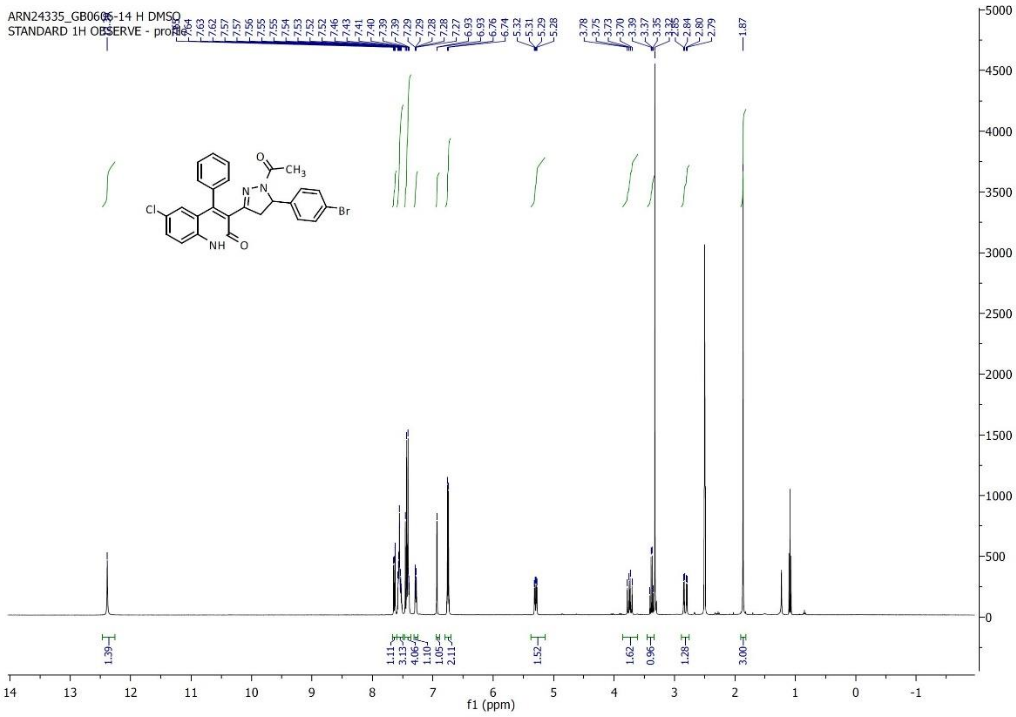

^{13}C-NMR spectrum (101 MHz, DMSO-d_6) of **37d**

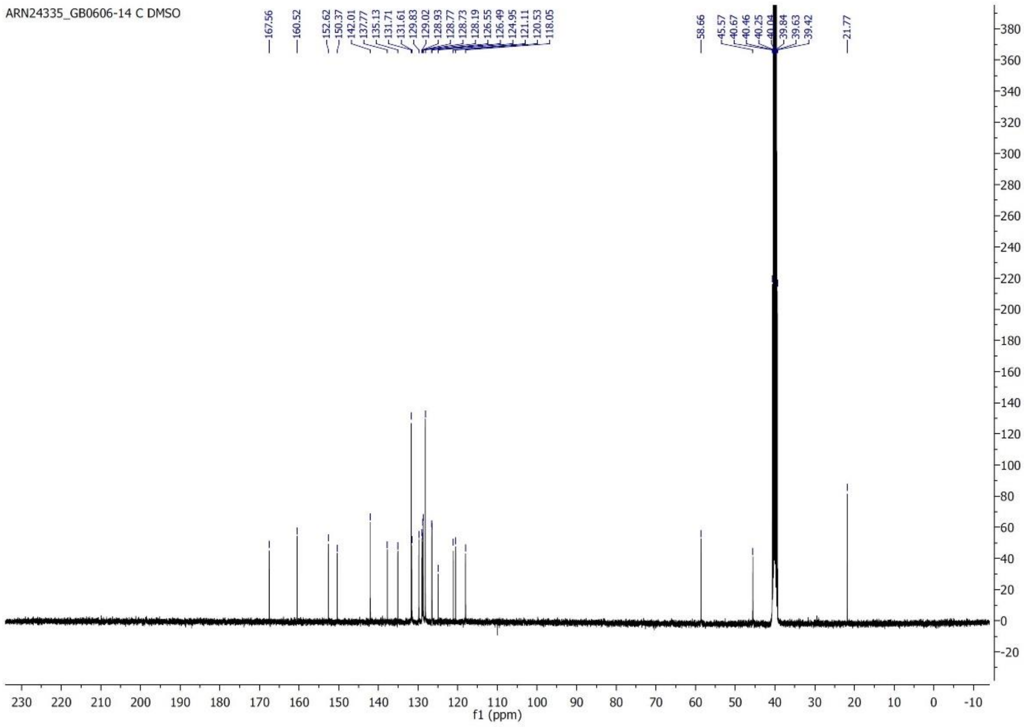

^{1}H-NMR spectrum (400 MHz, DMSO-d_6) of **38d**

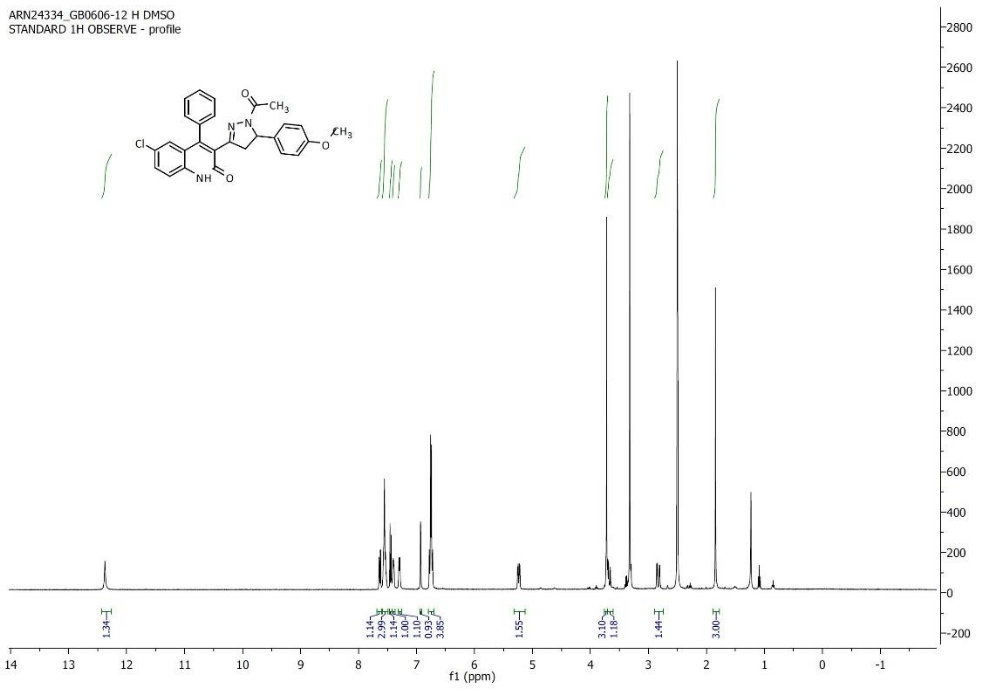

^{13}C-NMR spectrum (101 MHz, DMSO-d_6) of **38d**

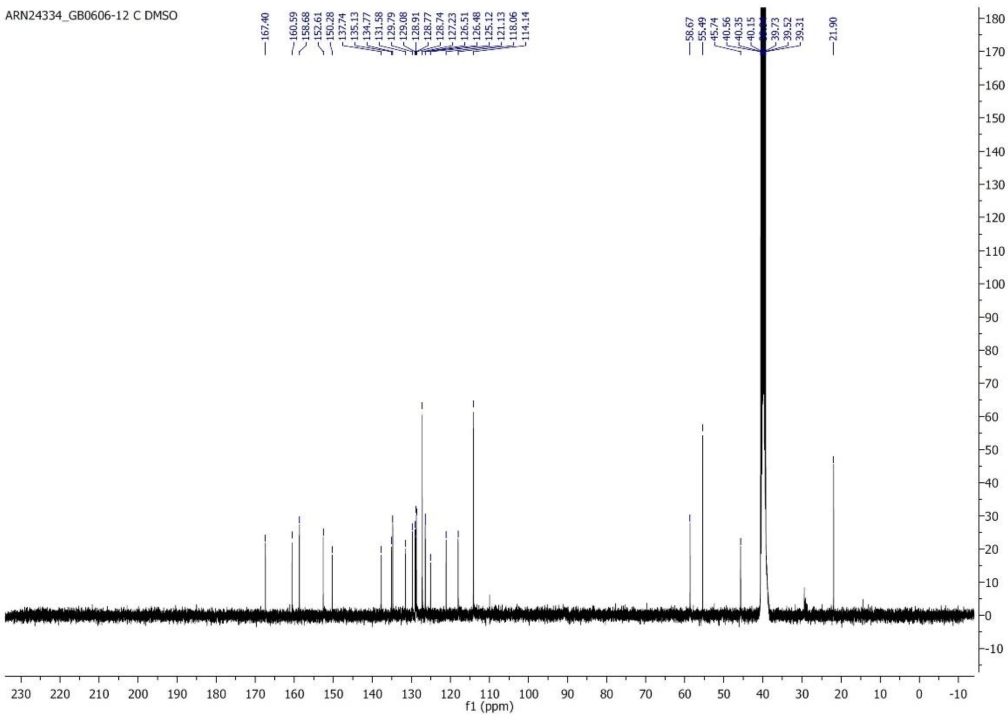

^{1}H-NMR spectrum (400 MHz, DMSO-d_6) of **39d**

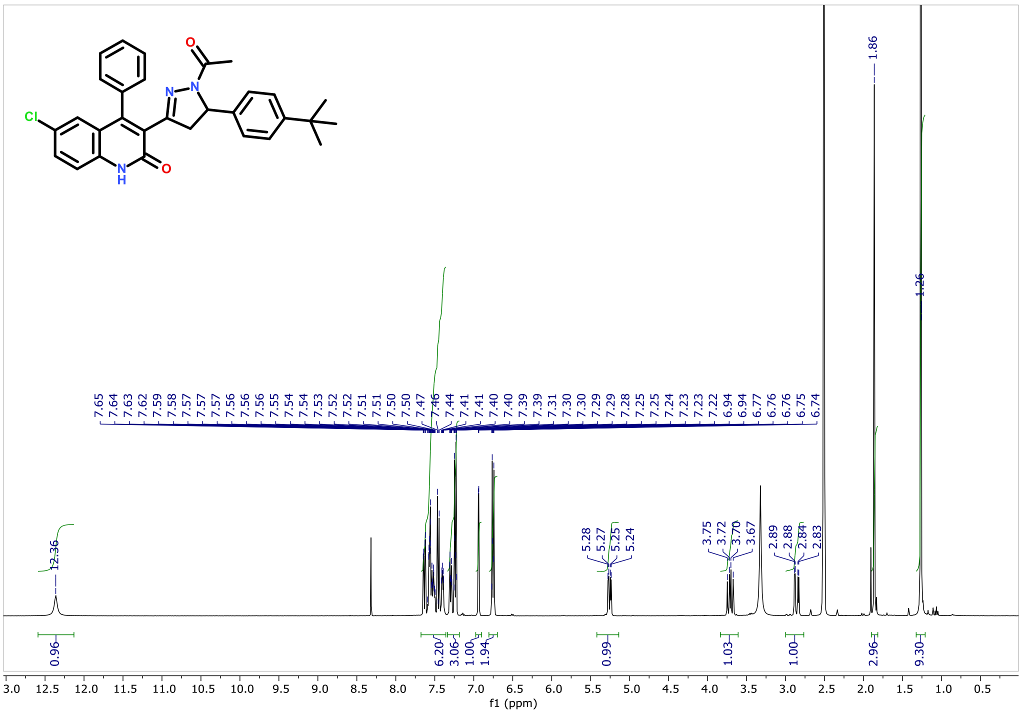

^{13}C-NMR spectrum (101 MHz, DMSO-d_6) of **39d**

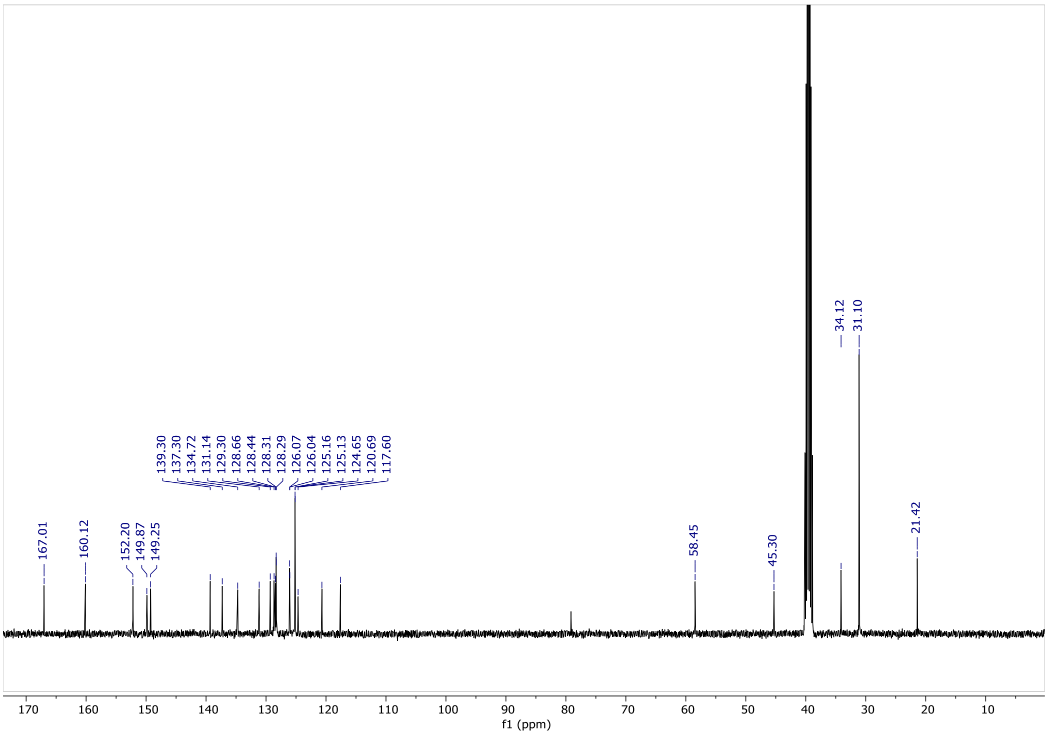

^{1}H-NMR spectrum (400 MHz, DMSO-d_6) of **40d**

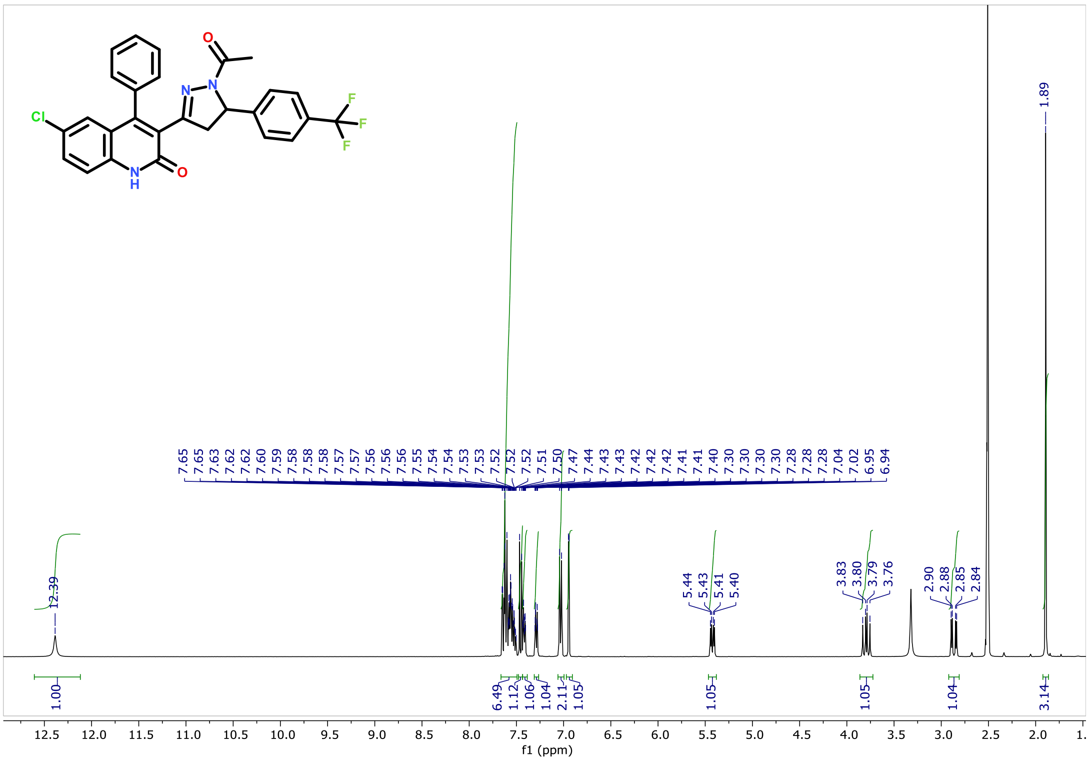

^{13}C-NMR spectrum (101 MHz, DMSO-d_6) of **40d**

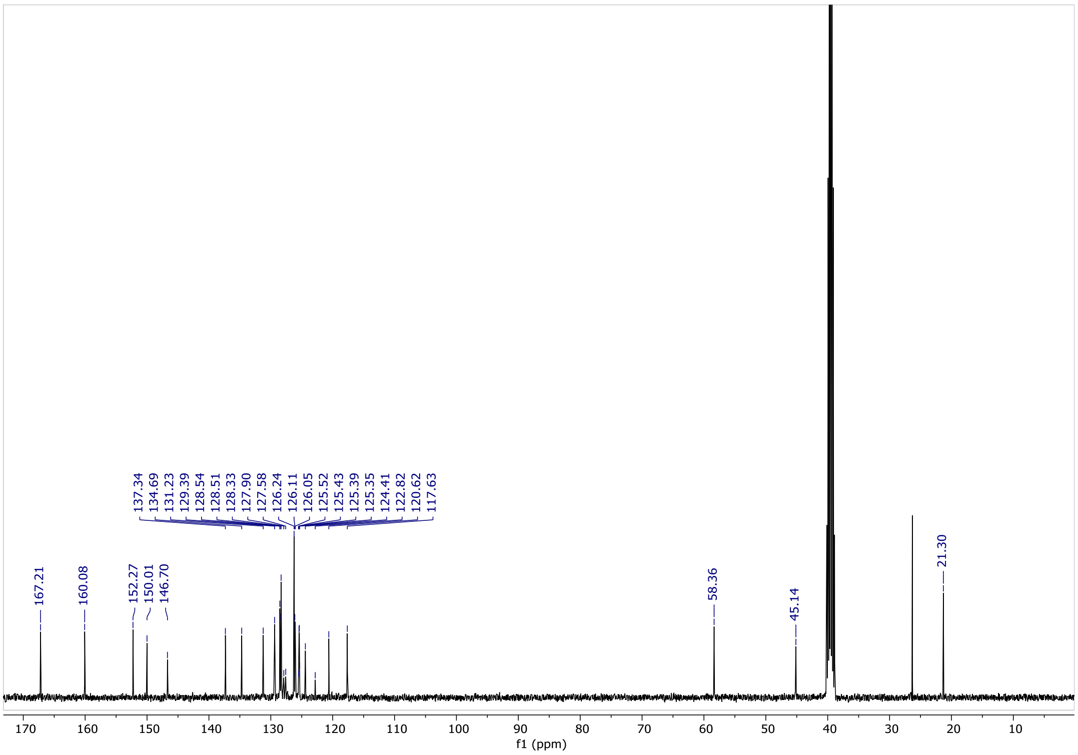

^{1}H-NMR spectrum (400 MHz, DMSO-d_6) of **41d**

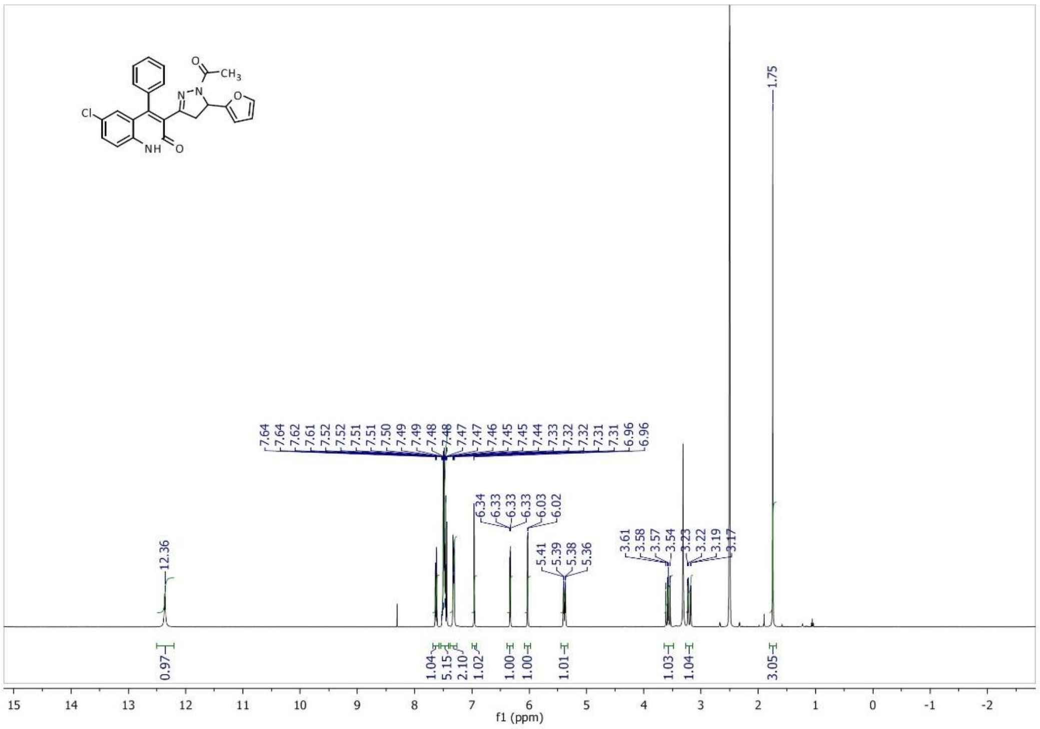

^{13}C-NMR spectrum (101 MHz, DMSO-d_6) of **41d**

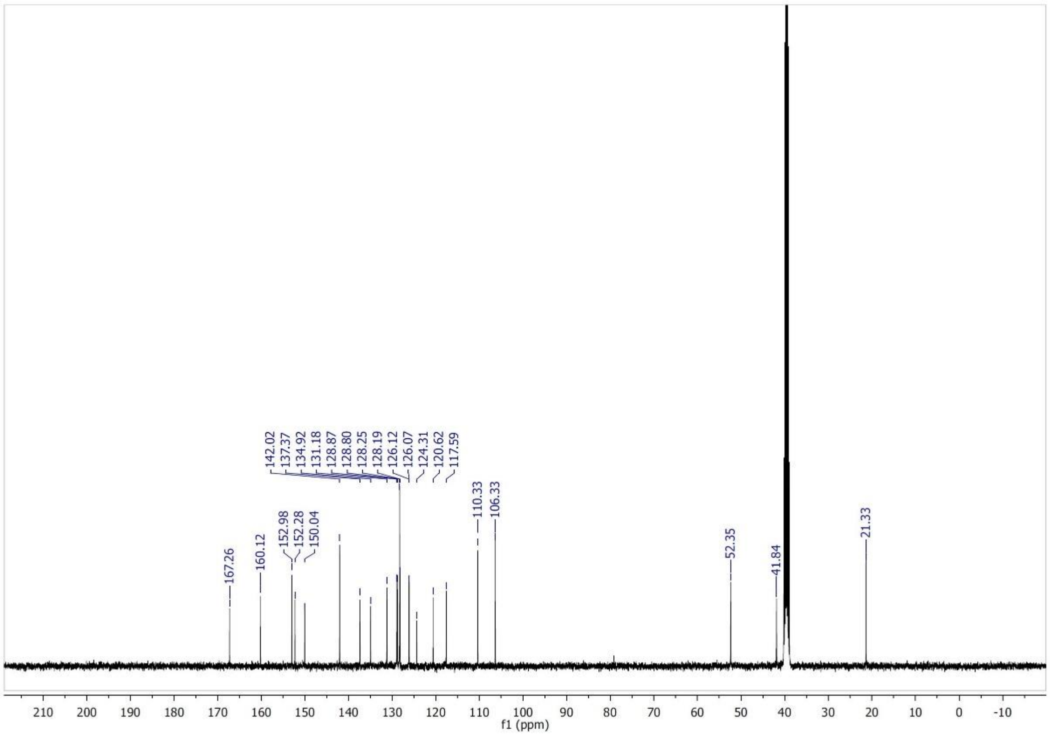

^{1}H-NMR spectrum (400 MHz, DMSO-d_6) of **42d**

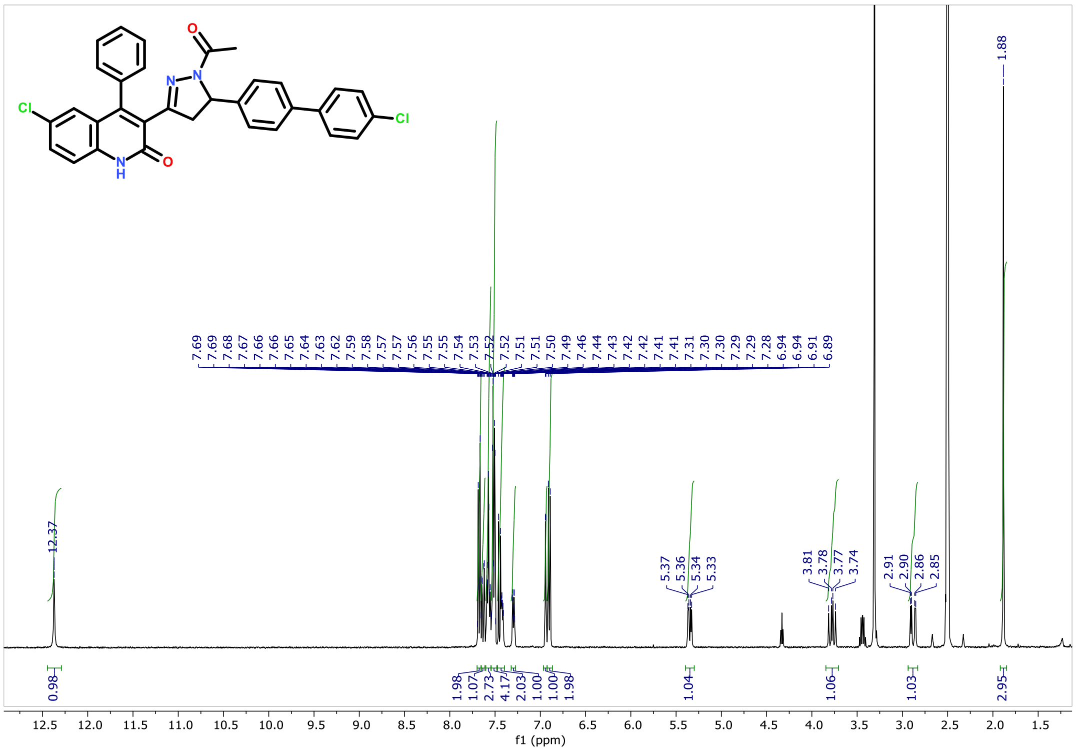

^{13}C-NMR spectrum (101 MHz, DMSO-d_6) of **42d**

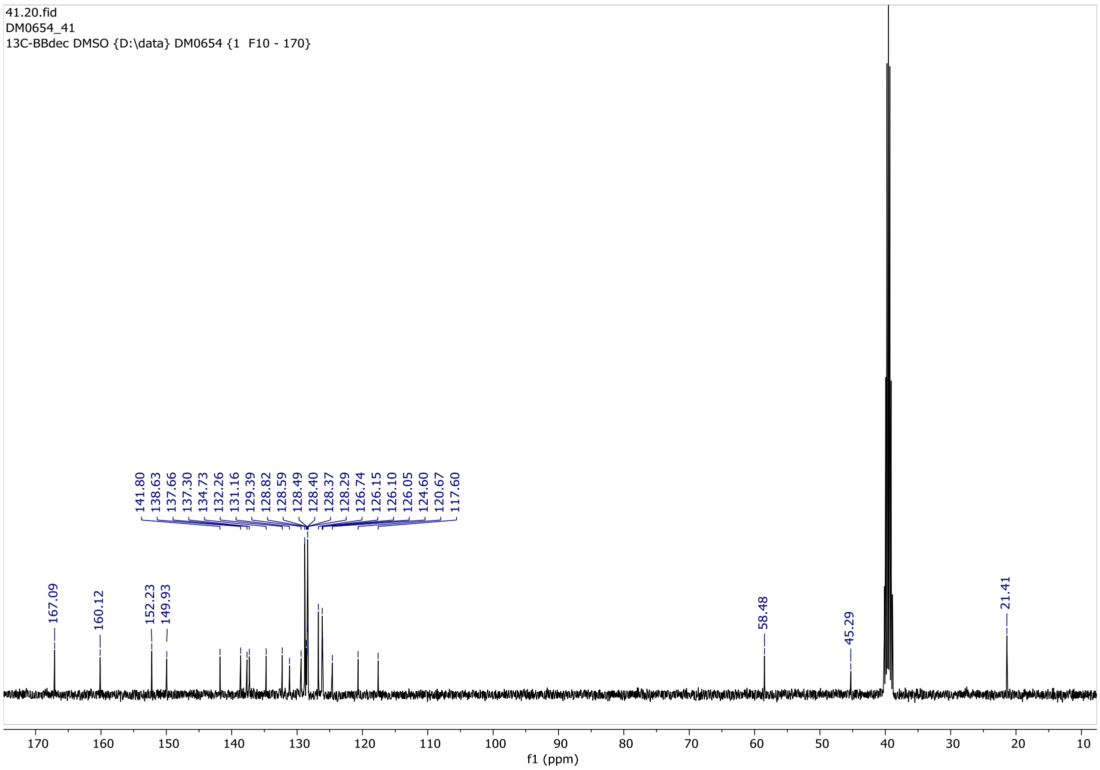

^{1}H-NMR spectrum (400 MHz, DMSO-d_6) of **43d**

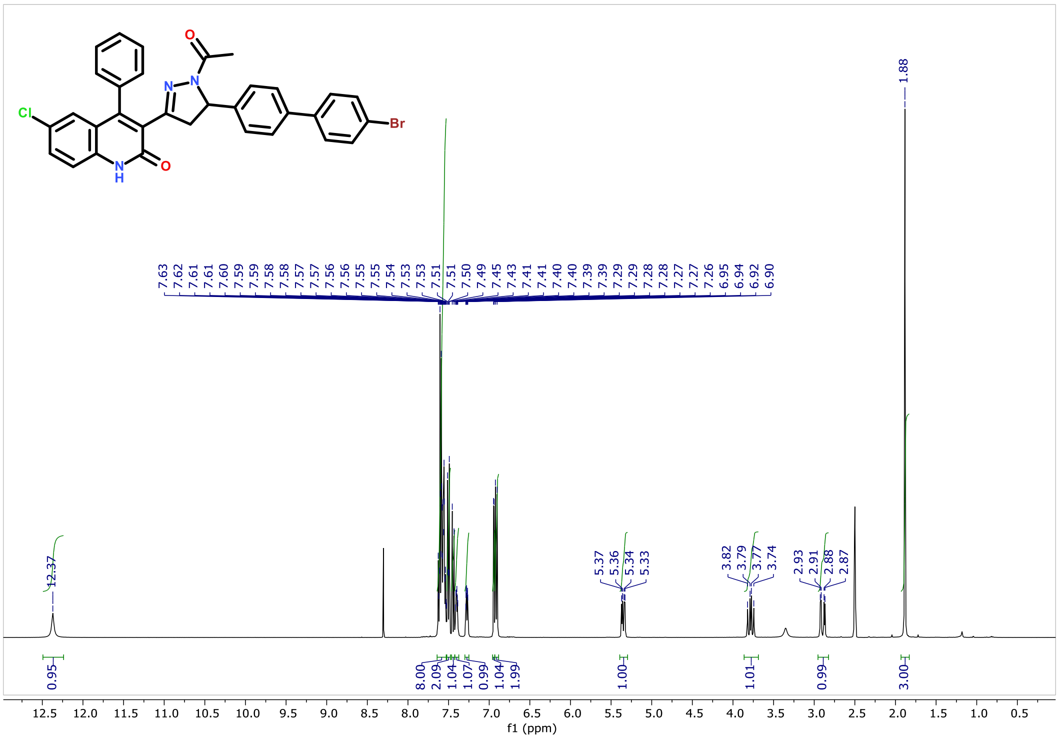

^{13}C-NMR spectrum (101 MHz, DMSO-d_6) of **43d**

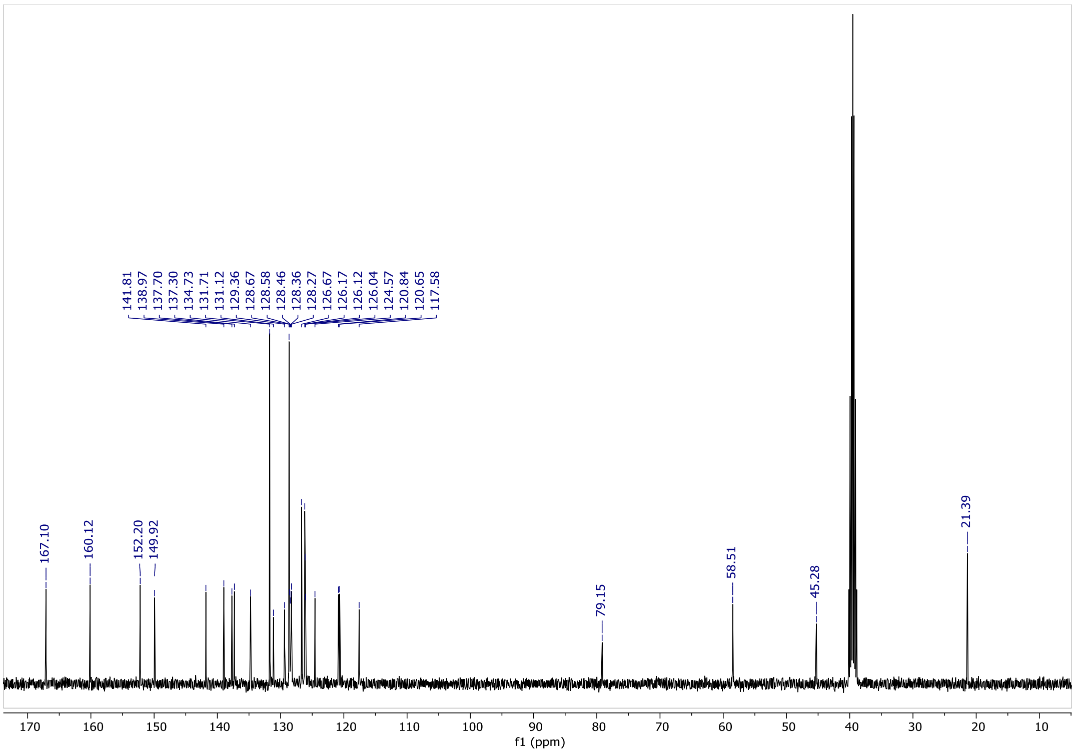

^{1}H-NMR spectrum (400 MHz, DMSO-d_6) of **44d**

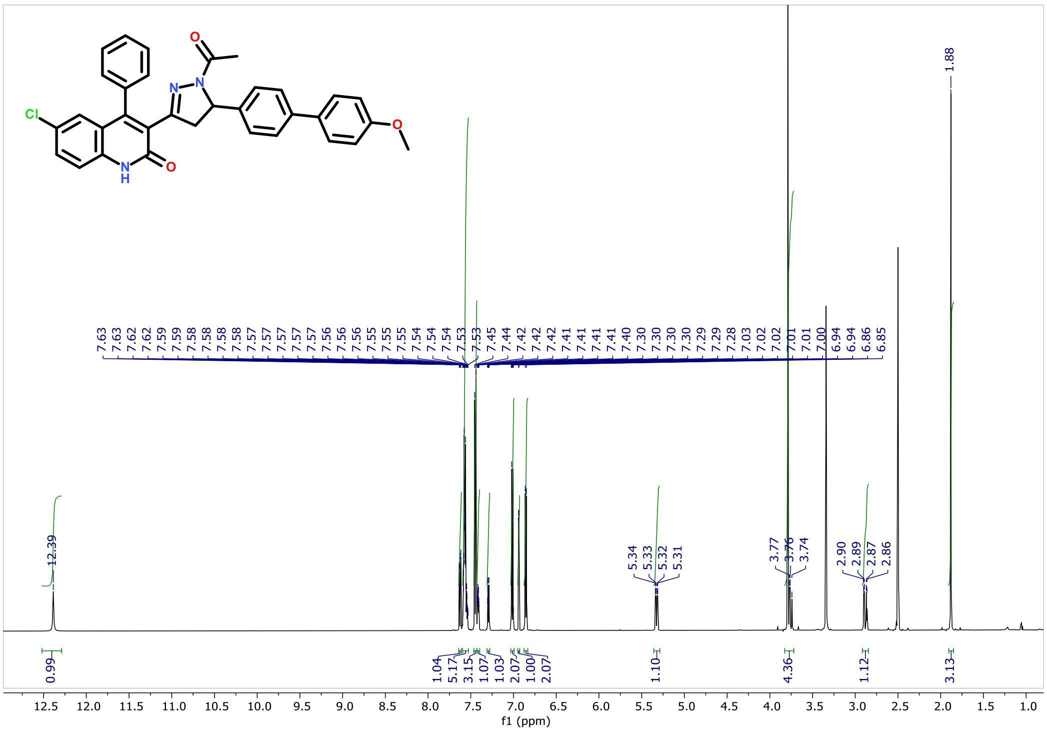

^{13}C-NMR spectrum (101 MHz, DMSO-d_6) of **44d**

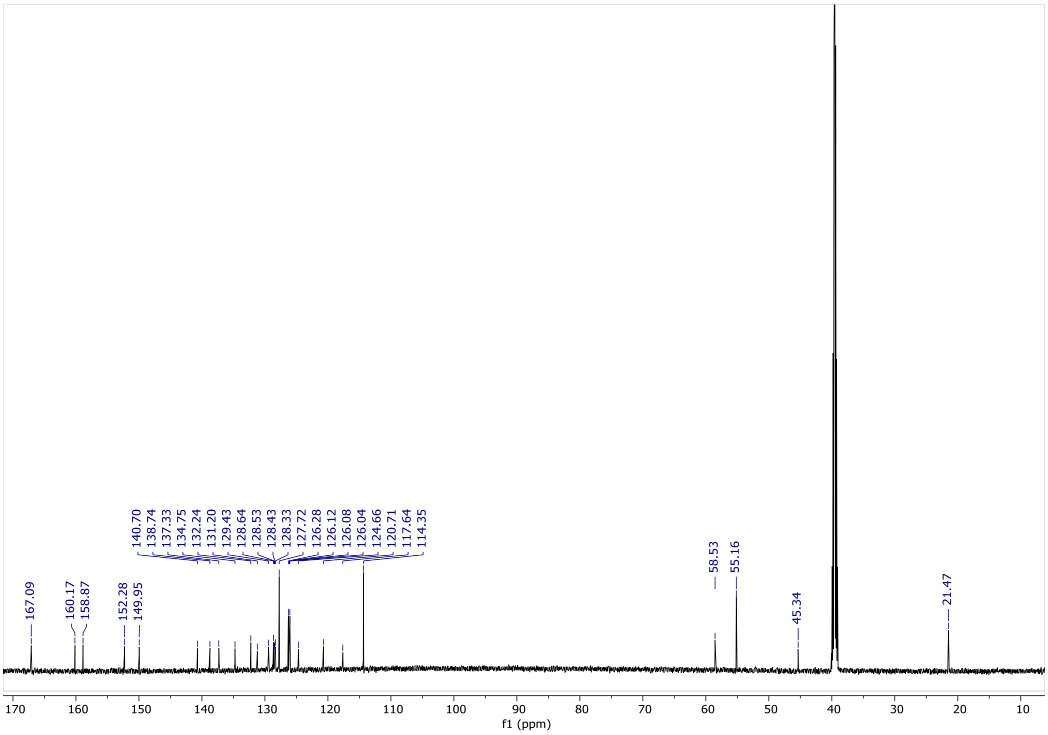

^{1}H-NMR spectrum (400 MHz, DMSO-d_6) of **45d**

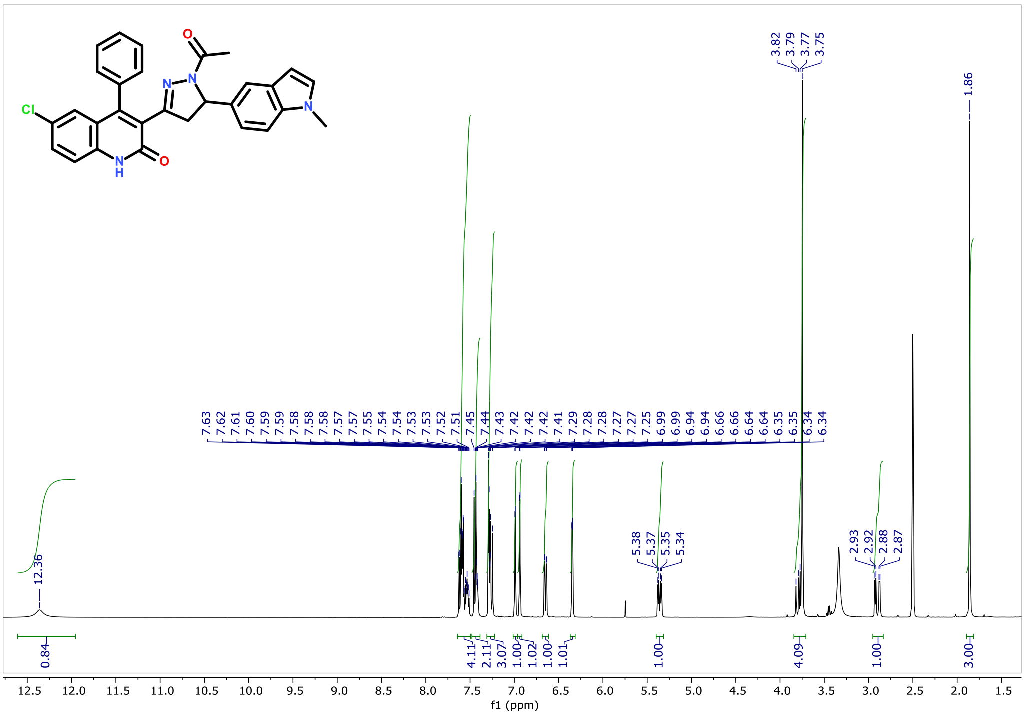

^{13}C-NMR spectrum (101 MHz, DMSO-d_6) of **45d**

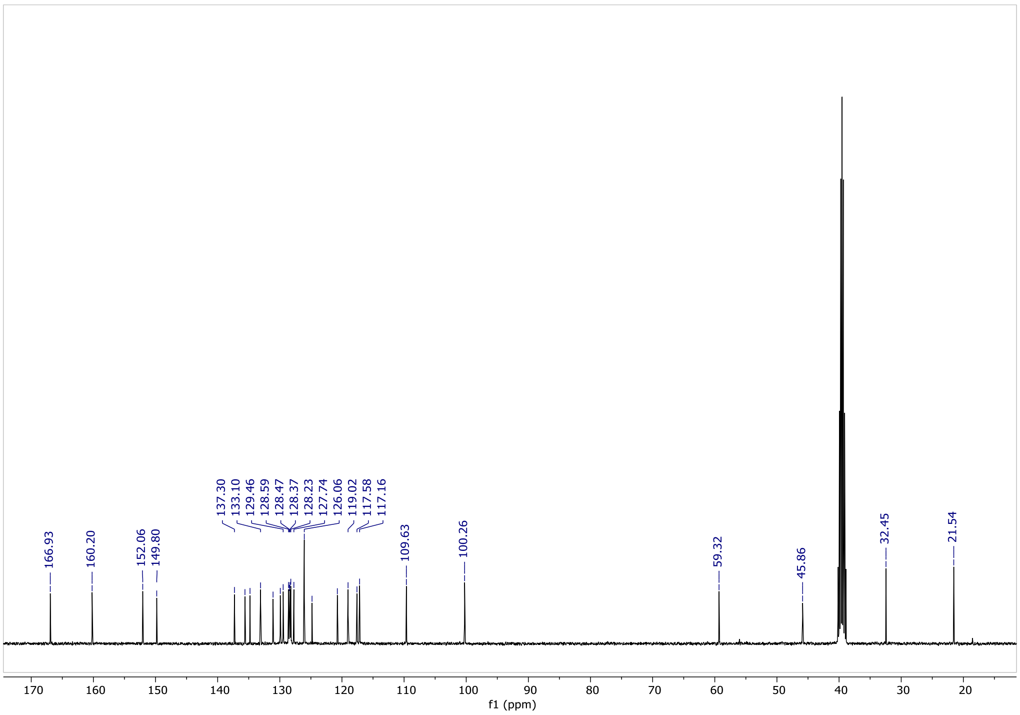

^{1}H-NMR spectrum (400 MHz, DMSO-d_6) of **46d**

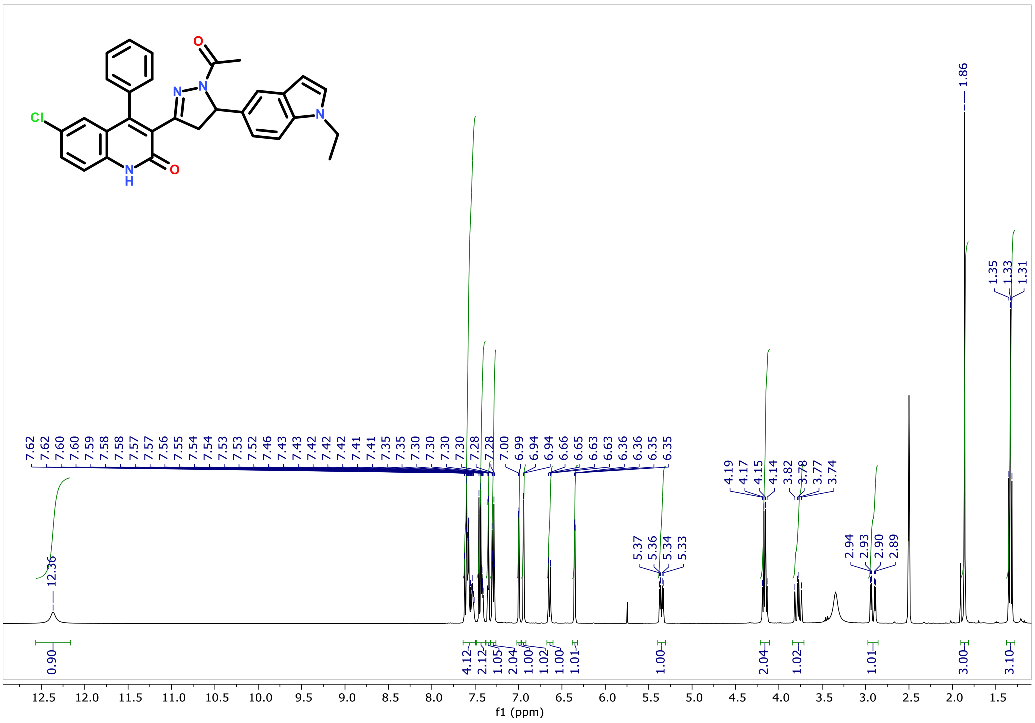

^{13}C-NMR spectrum (101 MHz, DMSO-d_6) of **46d**

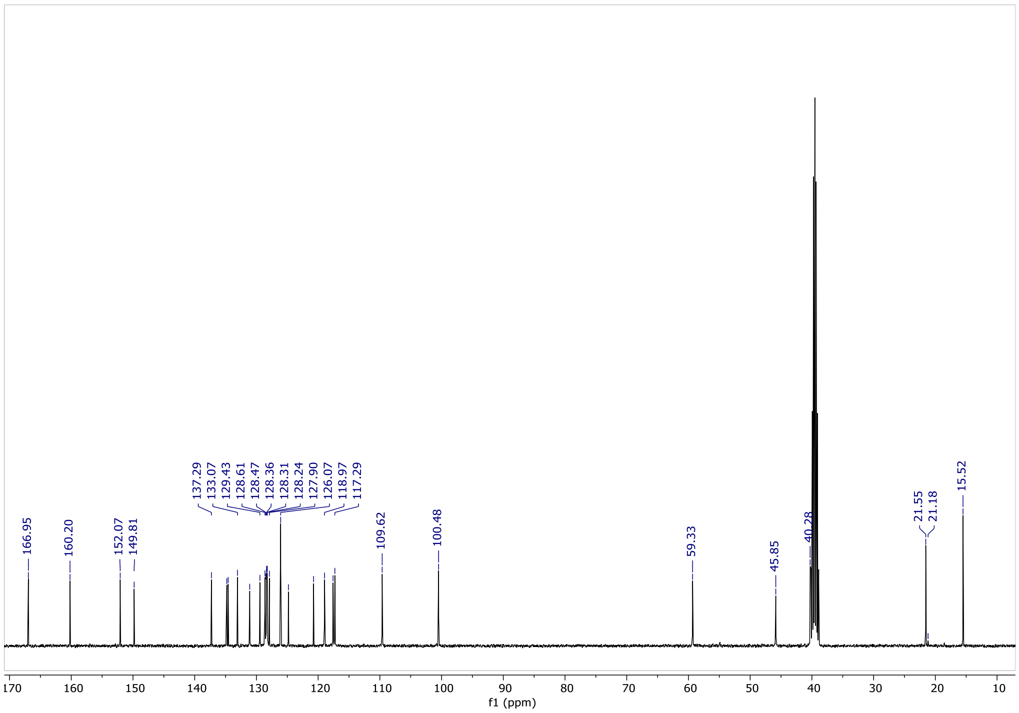

^{1}H-NMR spectrum (400 MHz, DMSO-d_6) of **47d**

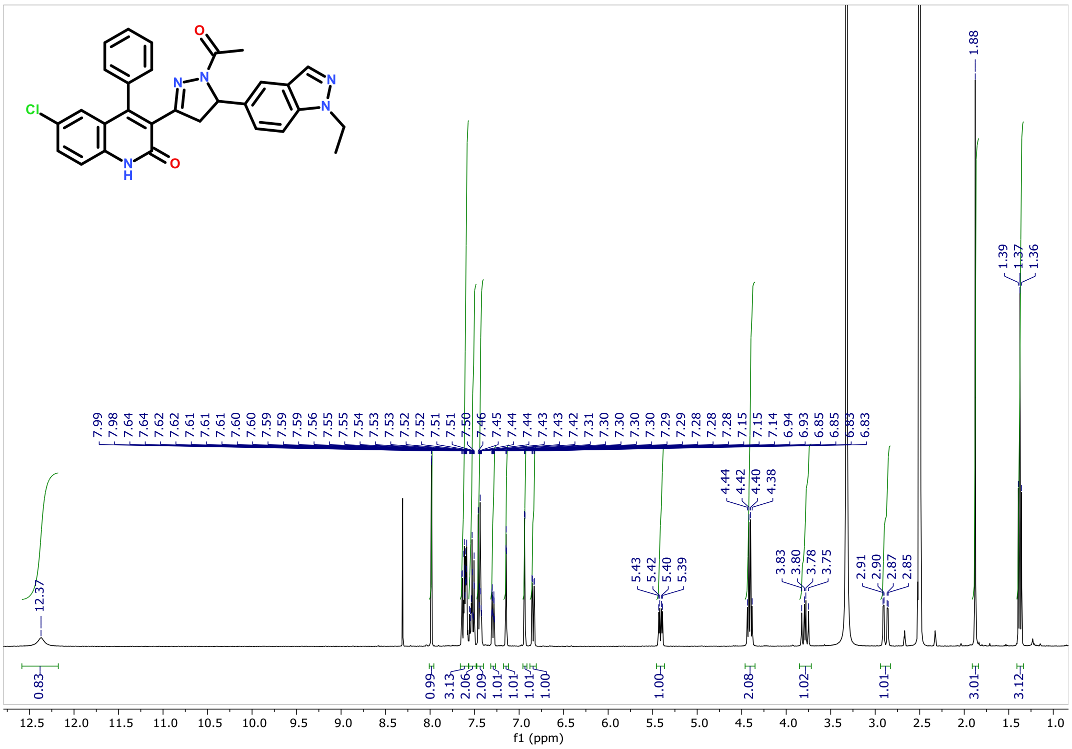

^{13}C-NMR spectrum (101 MHz, DMSO-d_6) of **47d**

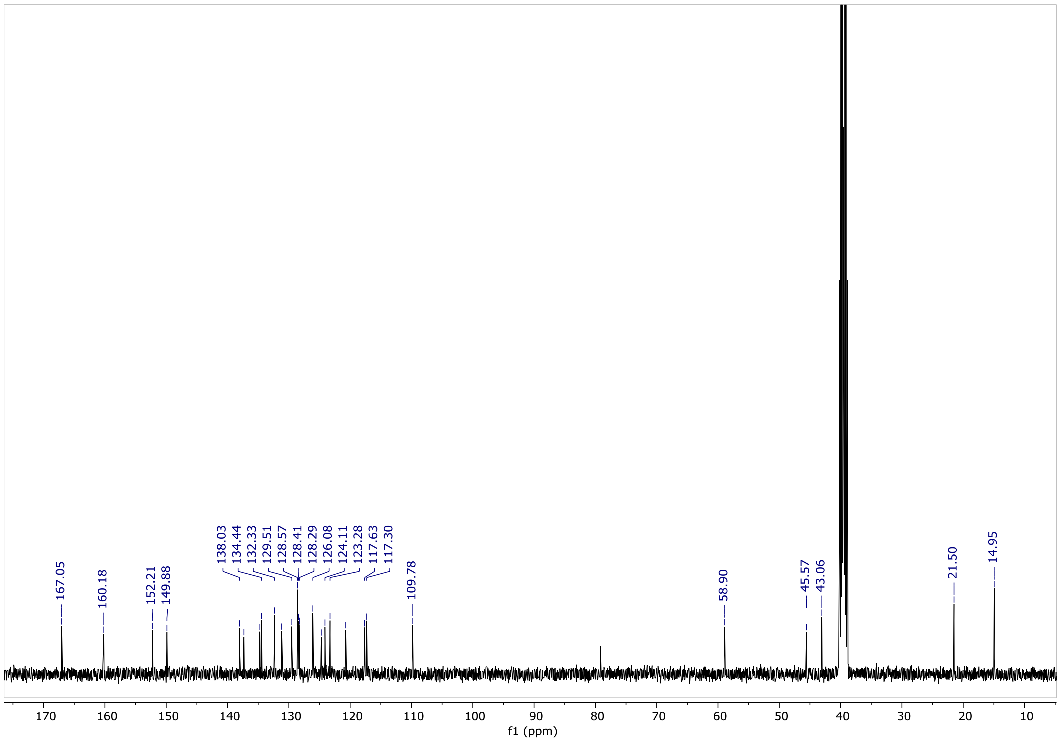

^{1}H-NMR spectrum (400 MHz, DMSO-d_6) of **48d**

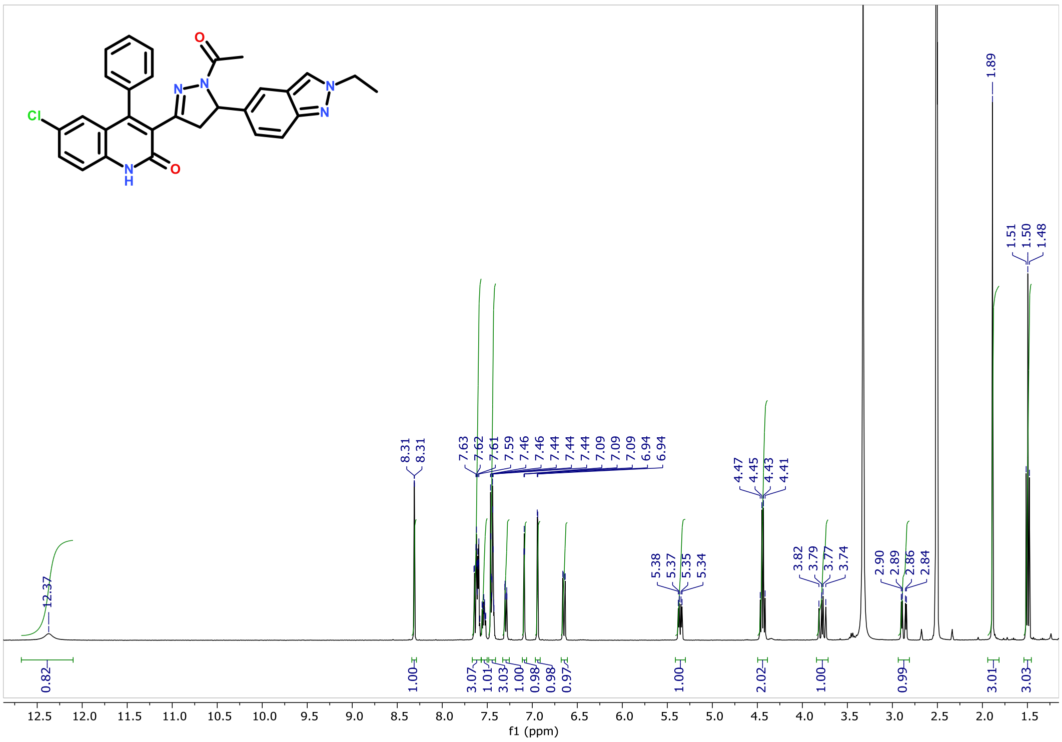

^{13}C-NMR spectrum (101 MHz, DMSO-d_6) of **48d**

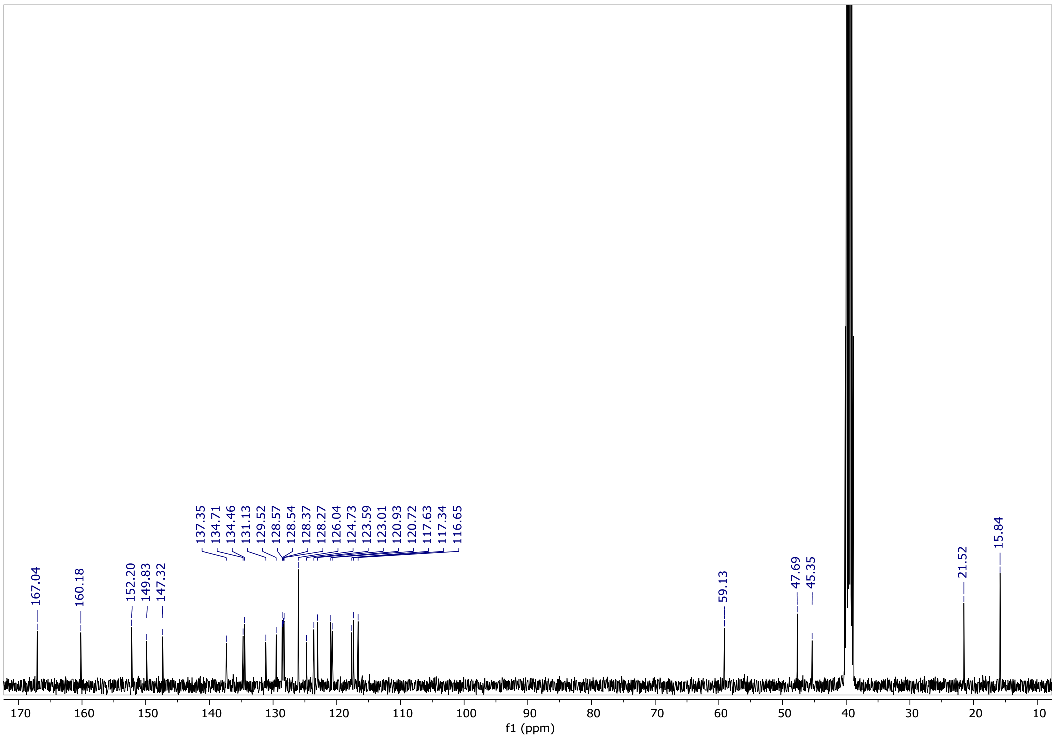

^{1}H-NMR spectrum (400 MHz, DMSO-d_6) of **49d**

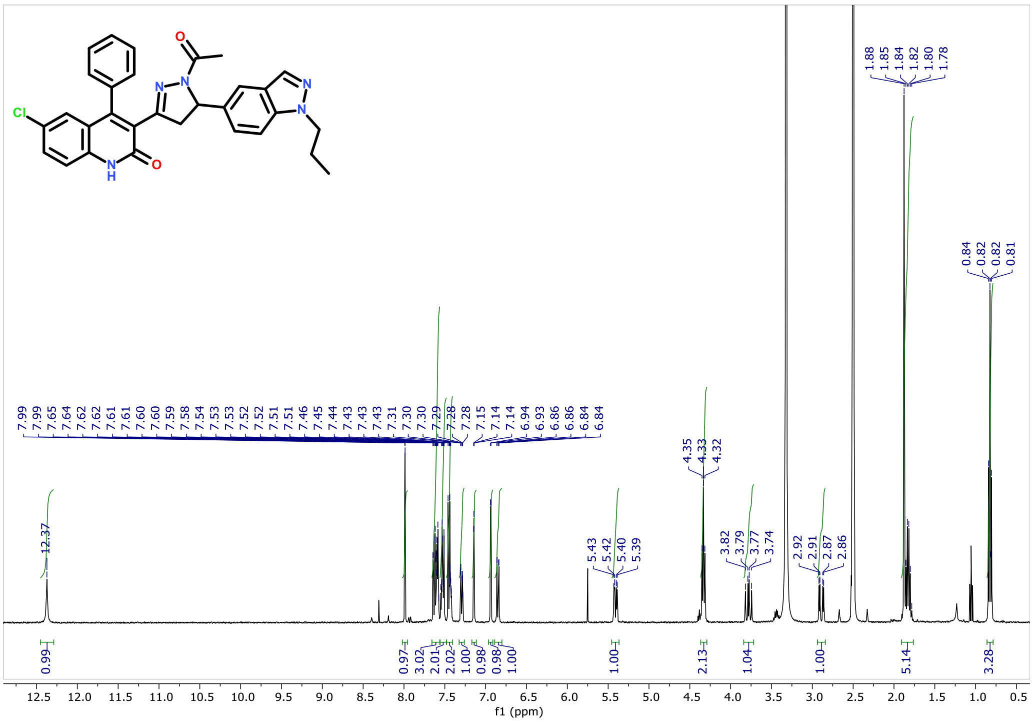

^{13}C-NMR spectrum (101 MHz, DMSO-d_6) of **49d**

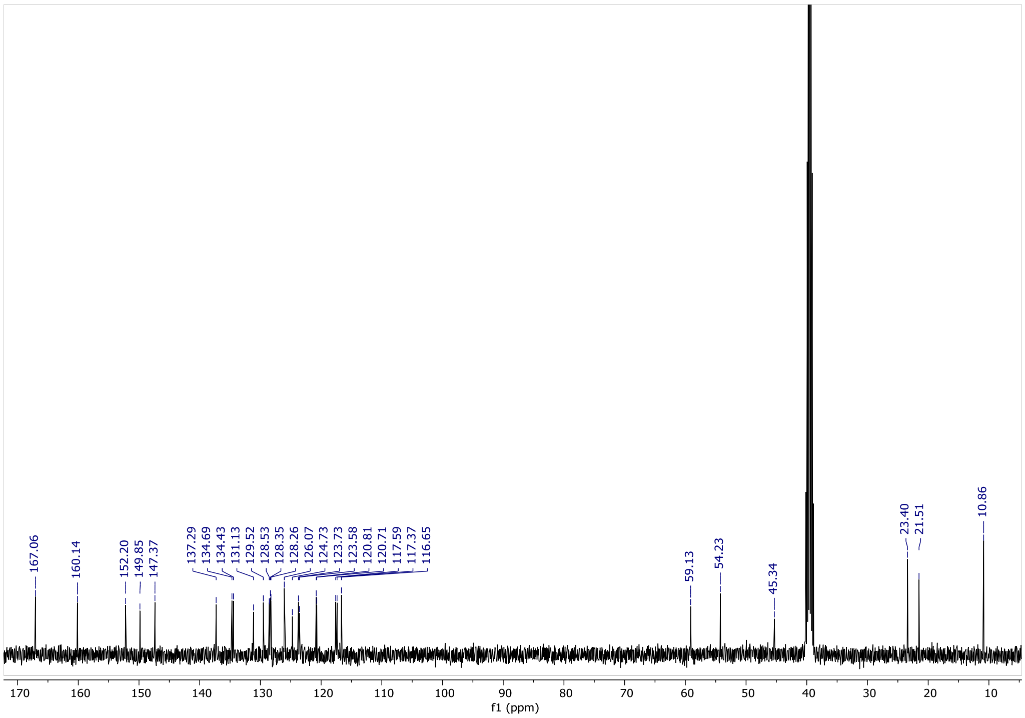

HPLC-MS analysis of **49d**

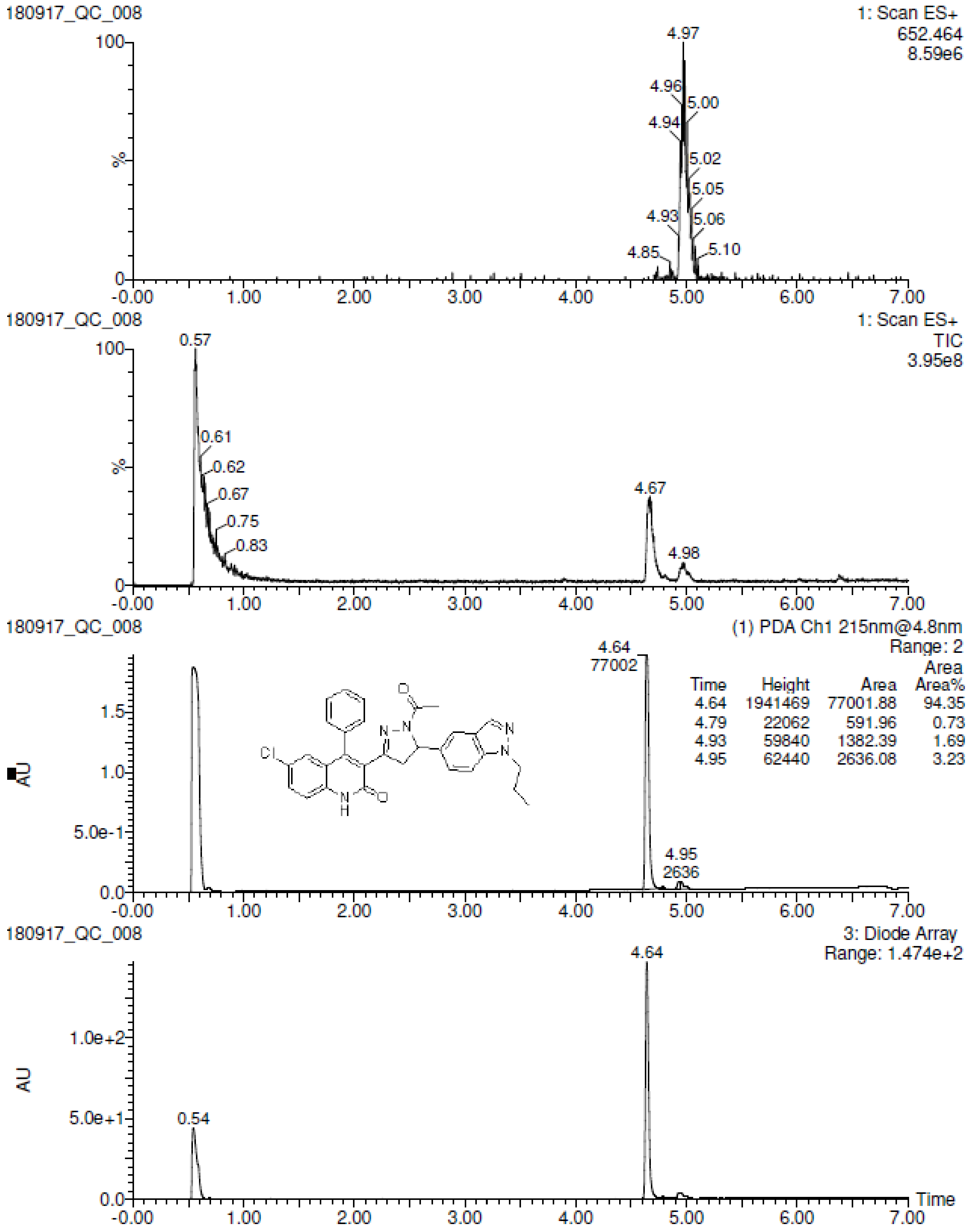

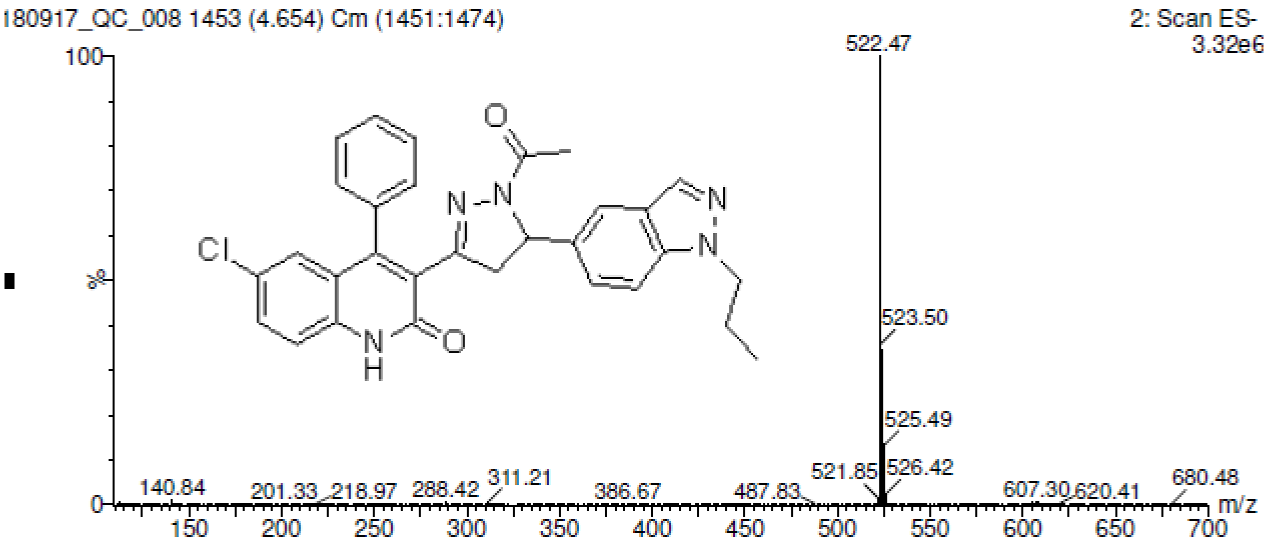
180917_QC_008 1453 (4.654) Cm (1451:1474)
2: Scan ES-
3.32e6
522.47
523.50
525.49
521.85
526.42
140.84
201.33
218.97
288.42
311.21
386.67
487.83
607.30
620.41
680.48
Cl
N
H
O
N
N
O
N
N
%
100
0
150
200
250
300
350
400
450
500
550
600
650
700
m/z

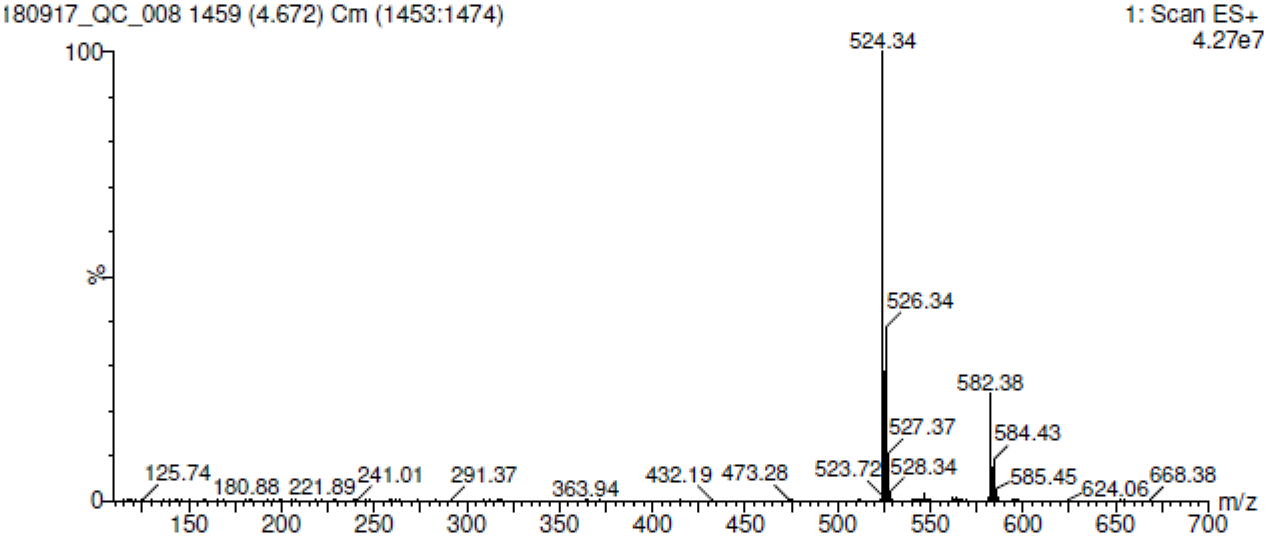
180917_QC_008 1459 (4.672) Cm (1453:1474)
1: Scan ES+
4.27e7
524.34
526.34
582.38
527.37
584.43
523.72
528.34
585.45
125.74
180.88
221.89
241.01
291.37
363.94
432.19
473.28
624.06
668.38
%
100
0
150
200
250
300
350
400
450
500
550
600
650
700
m/z

^{1}H-NMR spectrum (400 MHz, DMSO-d_6) of **50d**

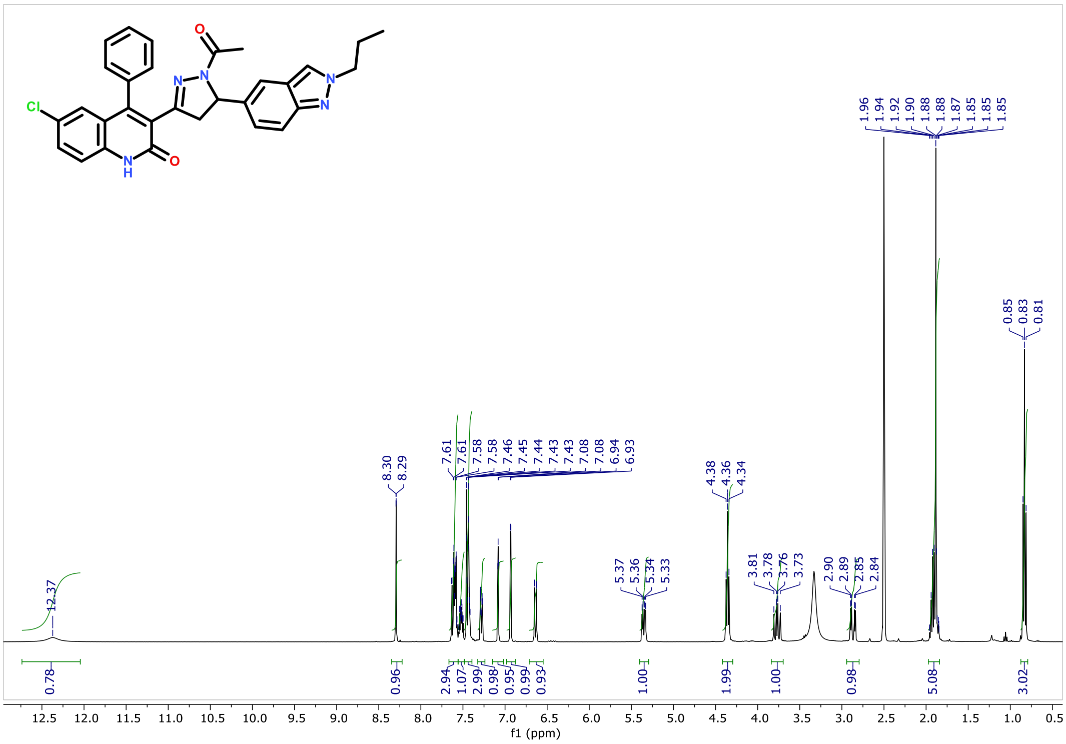

^{13}C-NMR spectrum (101 MHz, DMSO-d_6) of **50d**

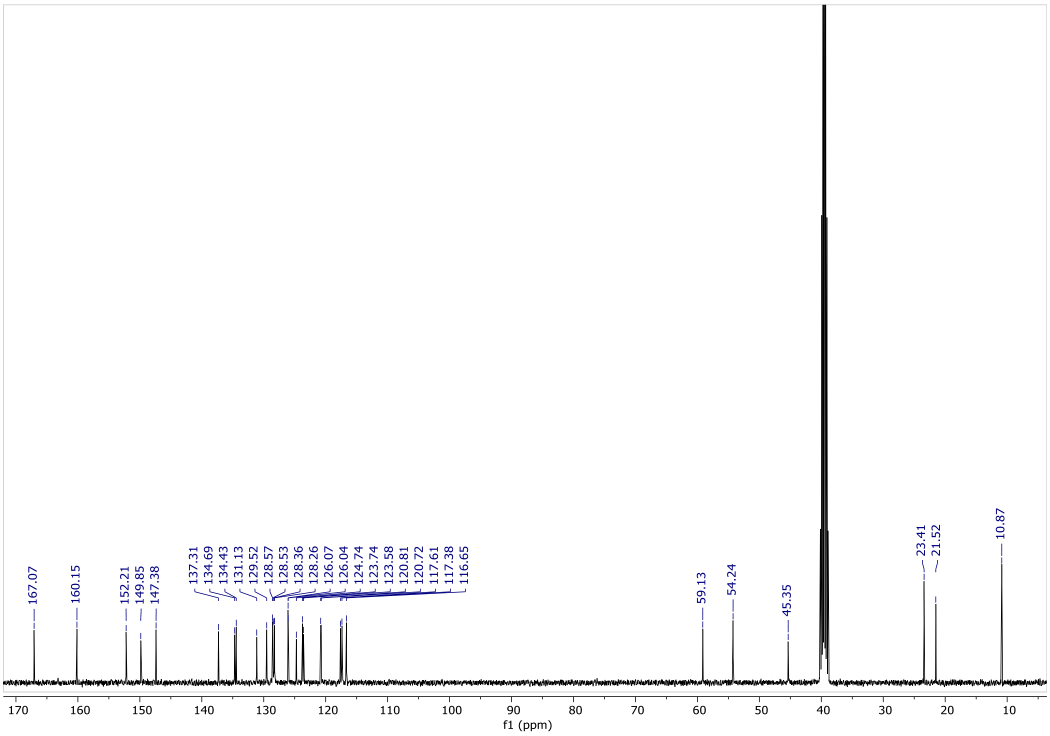

^{1}H-NMR spectrum (400 MHz, DMSO-d_6) of **51d**

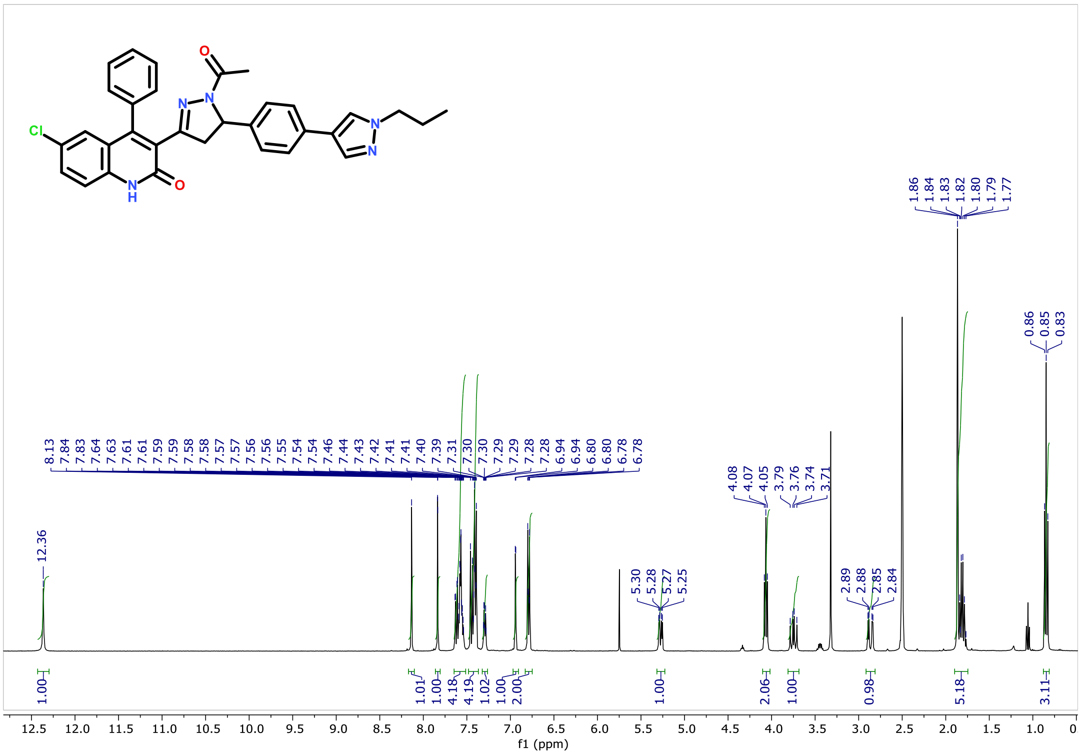

^{13}C-NMR spectrum (101 MHz, DMSO-d_6) of **51d**

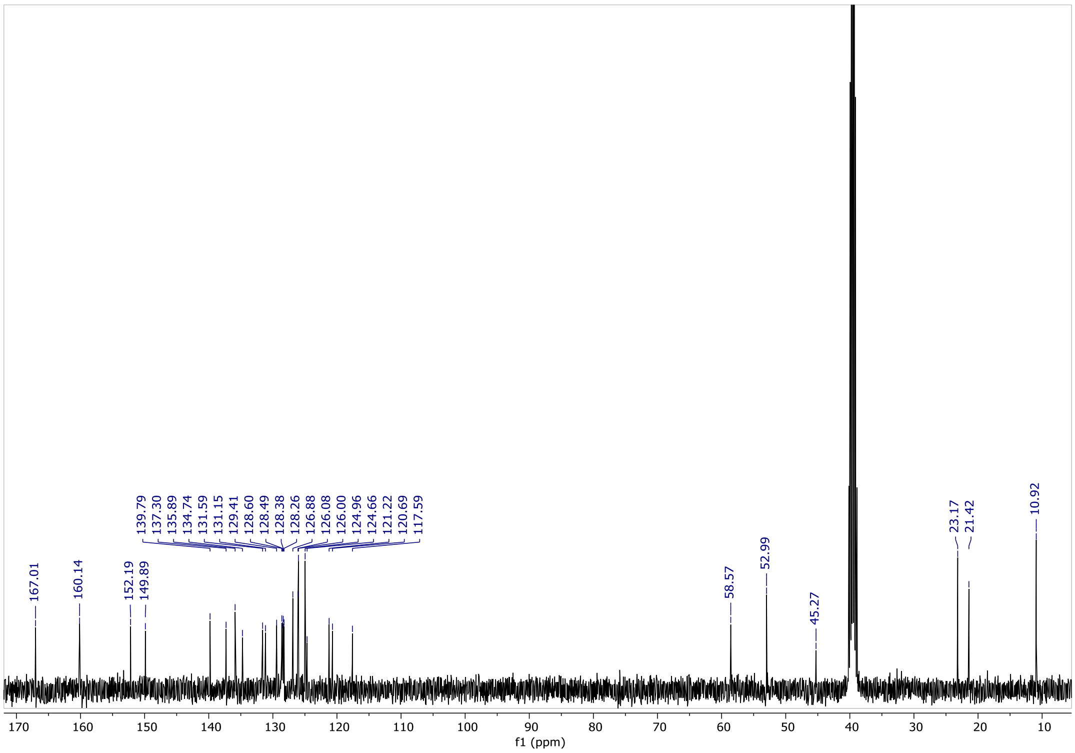

^{1}H-NMR spectrum (400 MHz, DMSO-d_6) of **52d**

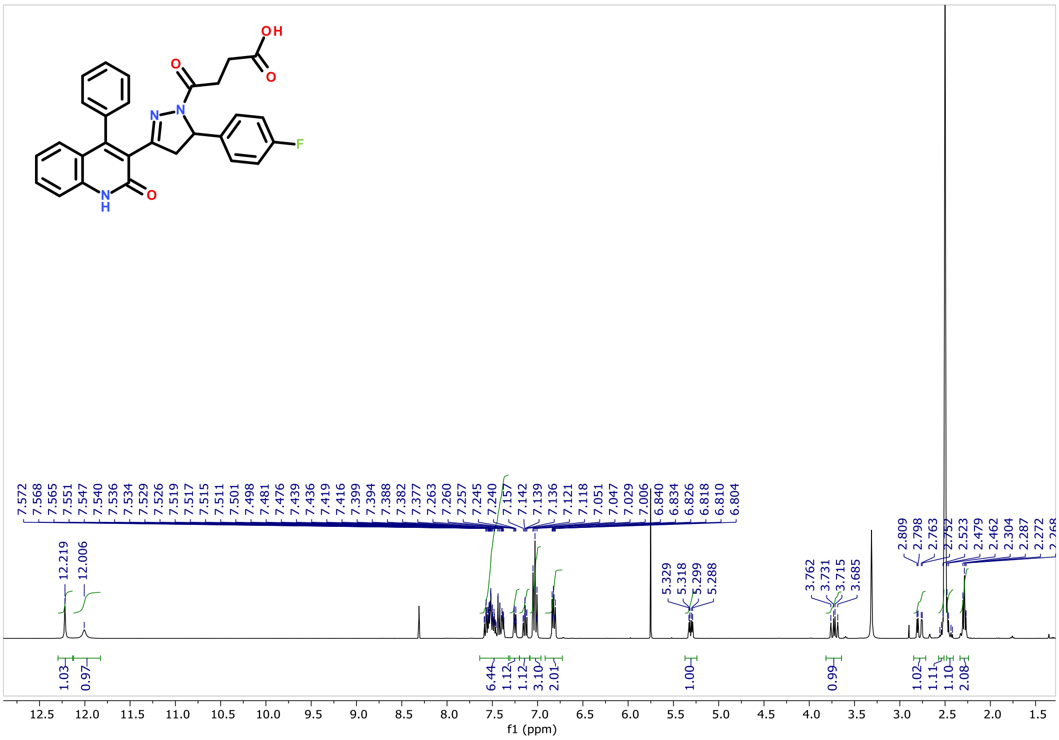

^{13}C-NMR spectrum (101 MHz, DMSO-d_6) of **52d**

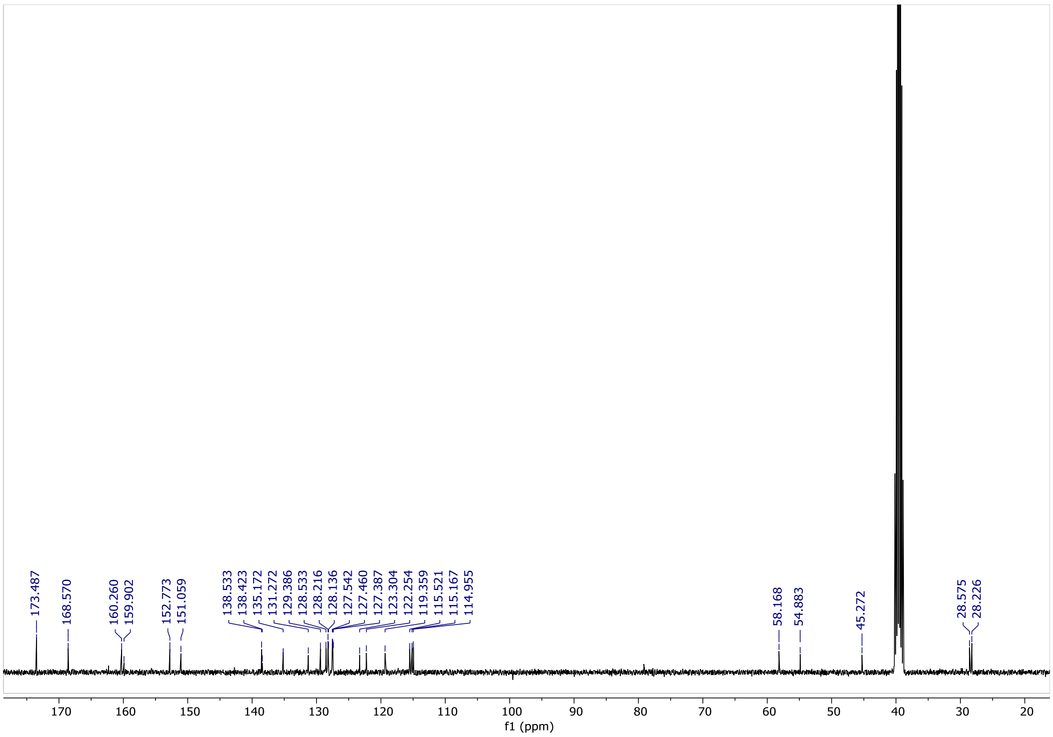

^{1}H-NMR spectrum (400 MHz, DMSO-d_6) of **53d**

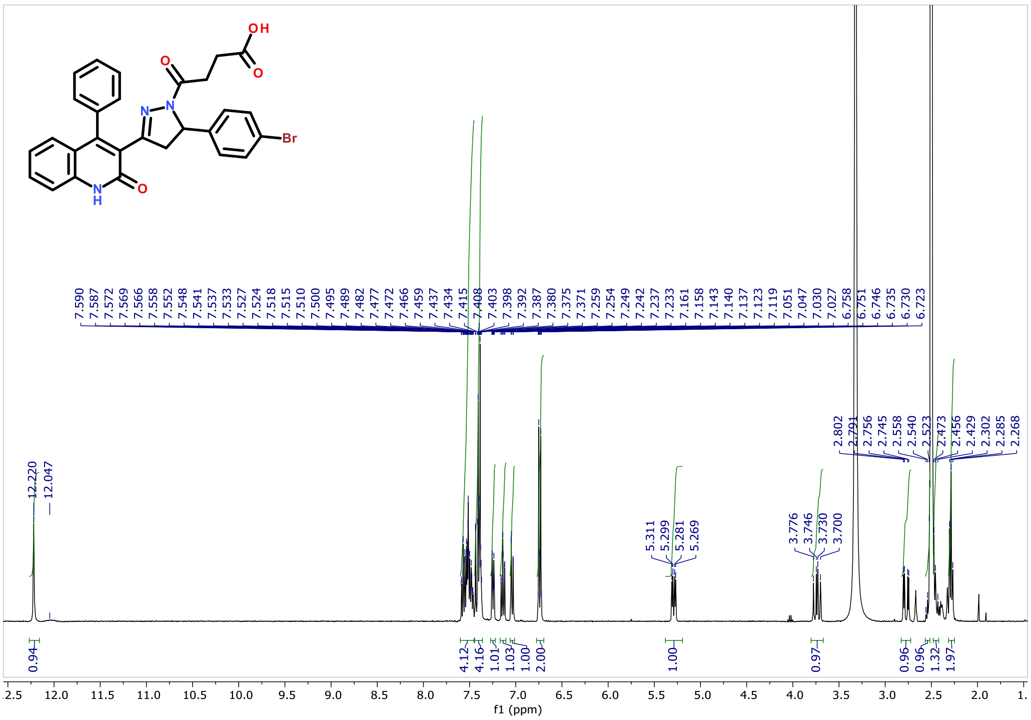

^{13}C-NMR spectrum (101 MHz, DMSO-d_6) of **53d**

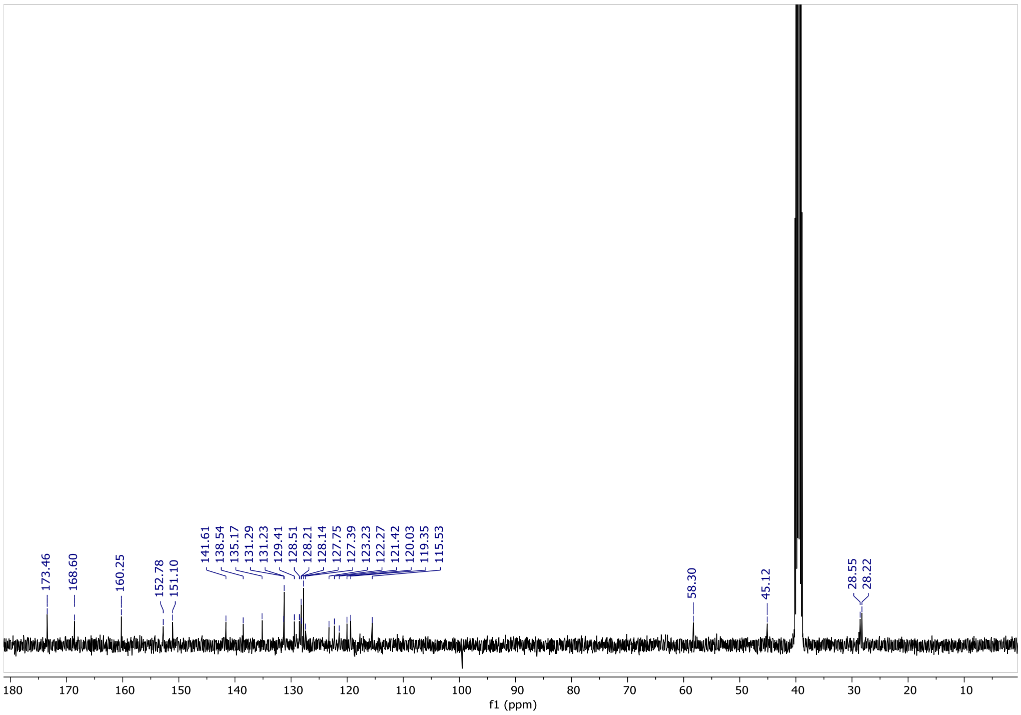

^{1}H-NMR spectrum (400 MHz, DMSO-d_6) of **54d**

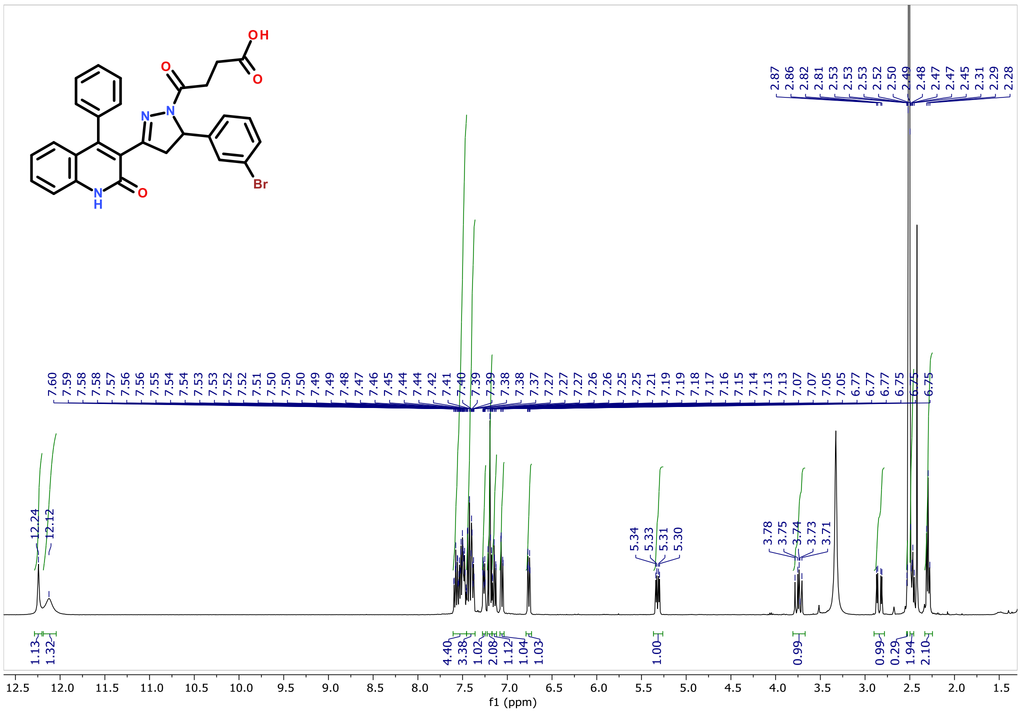

^{13}C-NMR spectrum (101 MHz, DMSO-d_6) of **54d**

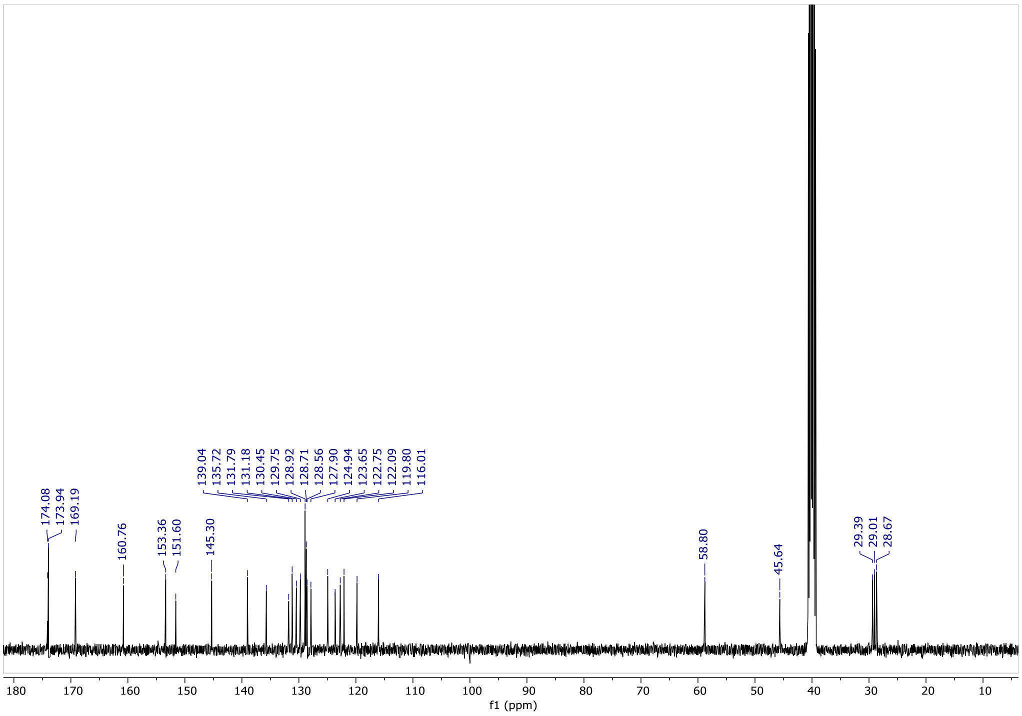

^{1}H-NMR spectrum (400 MHz, DMSO-d_6) of **55d**

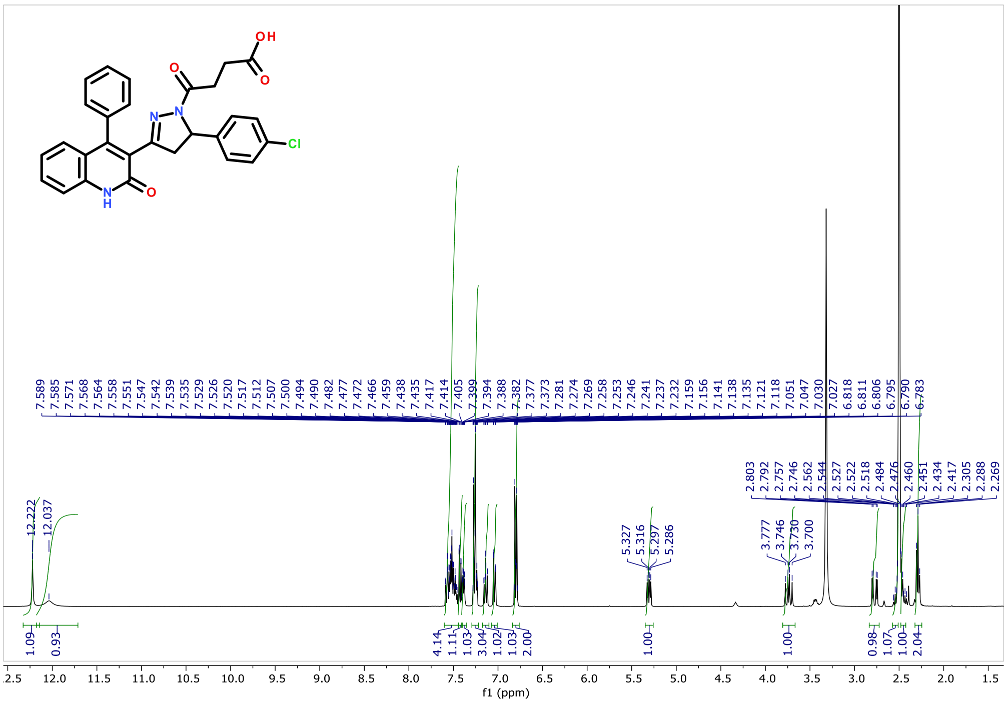

^{13}C-NMR spectrum (101 MHz, DMSO-d_6) of **55d**

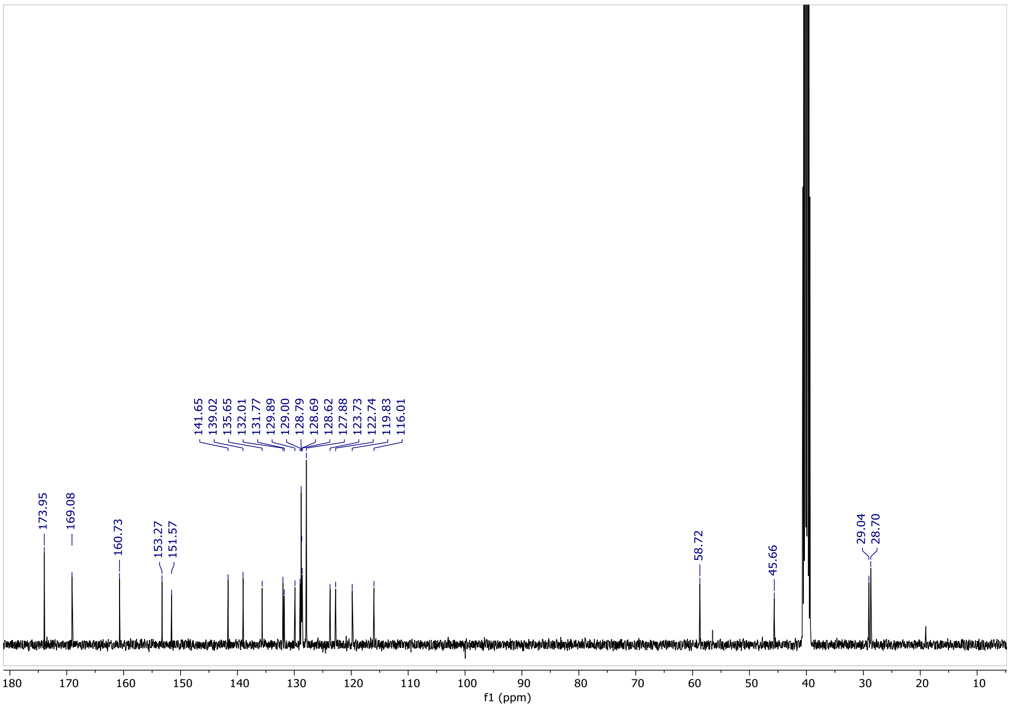

^{1}H-NMR spectrum (400 MHz, DMSO-d_6) of **56d**

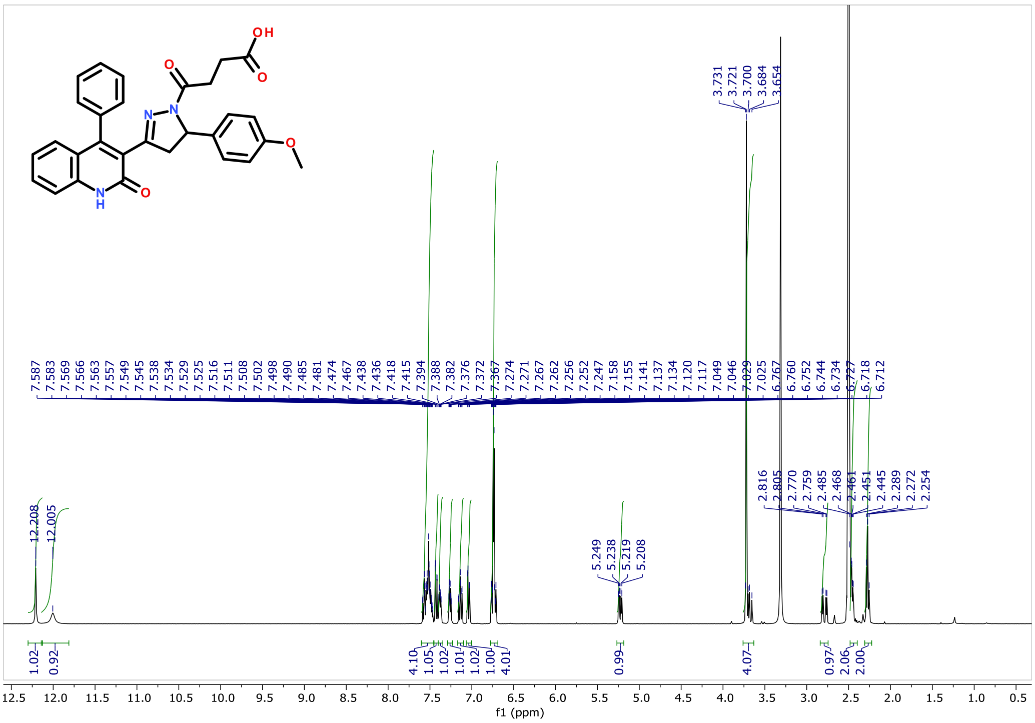

^{13}C-NMR spectrum (101 MHz, DMSO-d_6) of **56d**

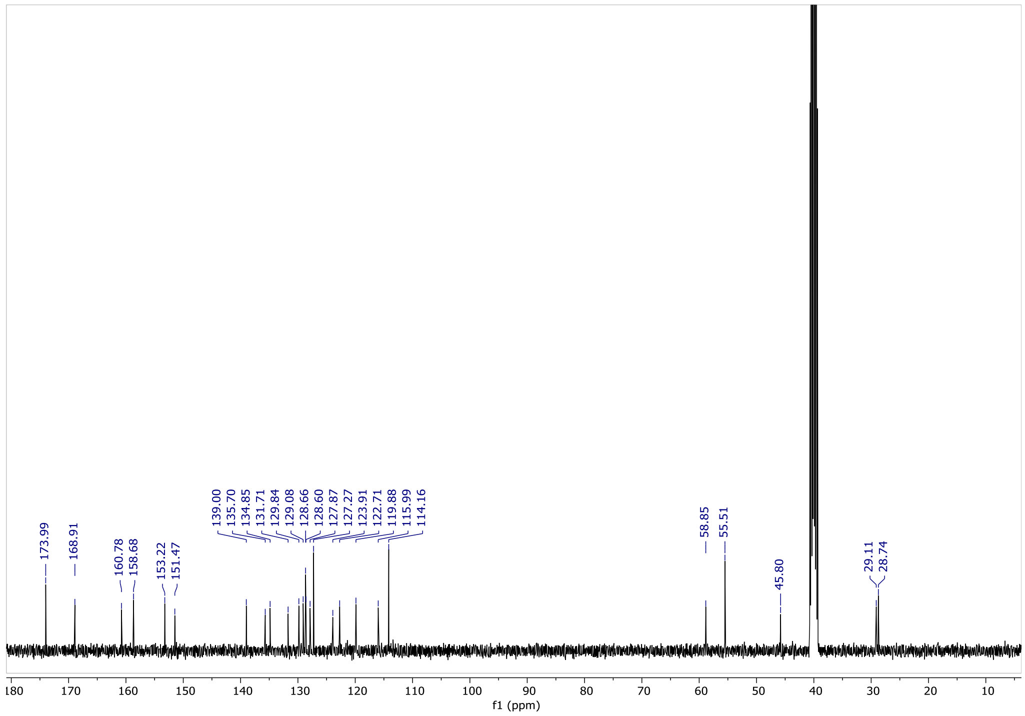

^{1}H-NMR spectrum (400 MHz, DMSO-d_6) of **57d**

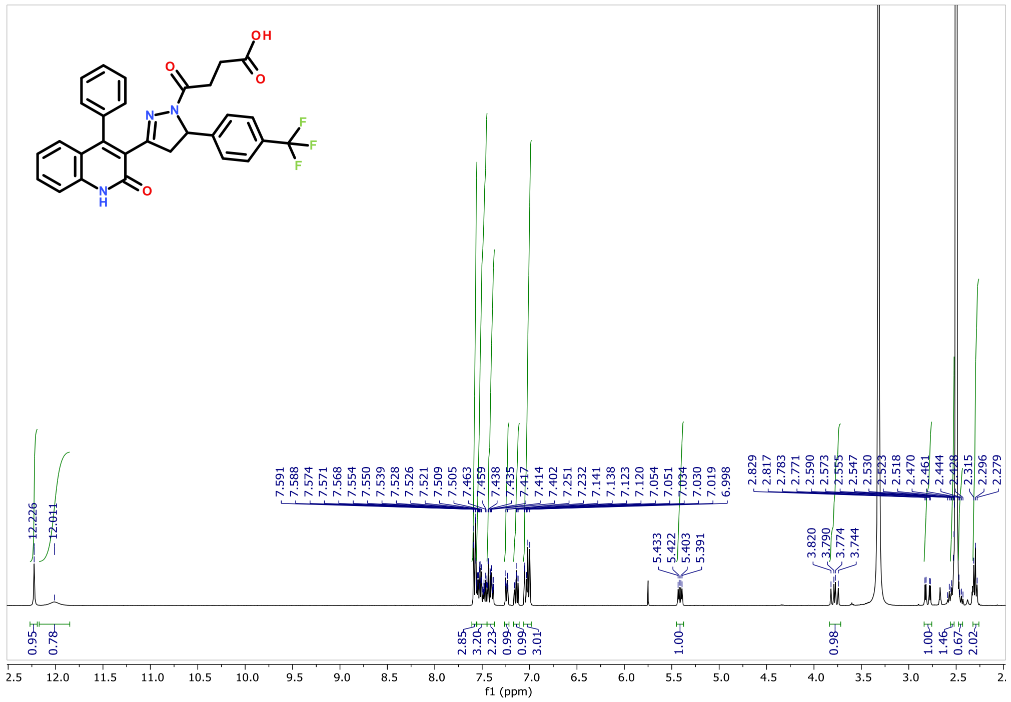

^{13}C-NMR spectrum (101 MHz, DMSO-d_6) of **57d**

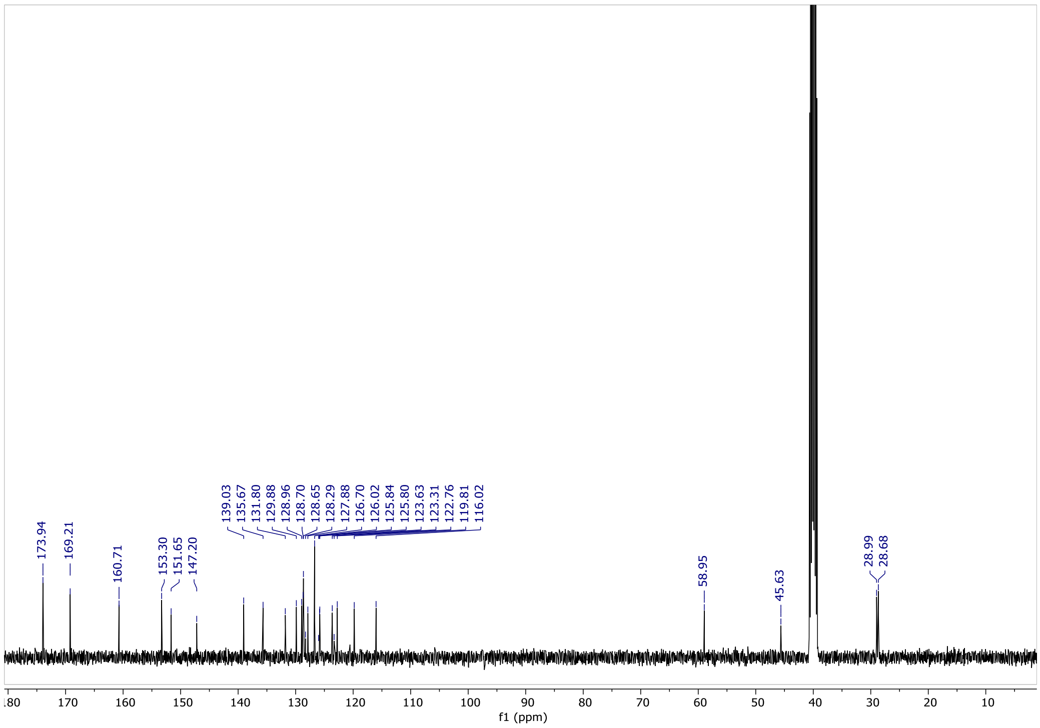

HPLC-MS analysis of **57d**

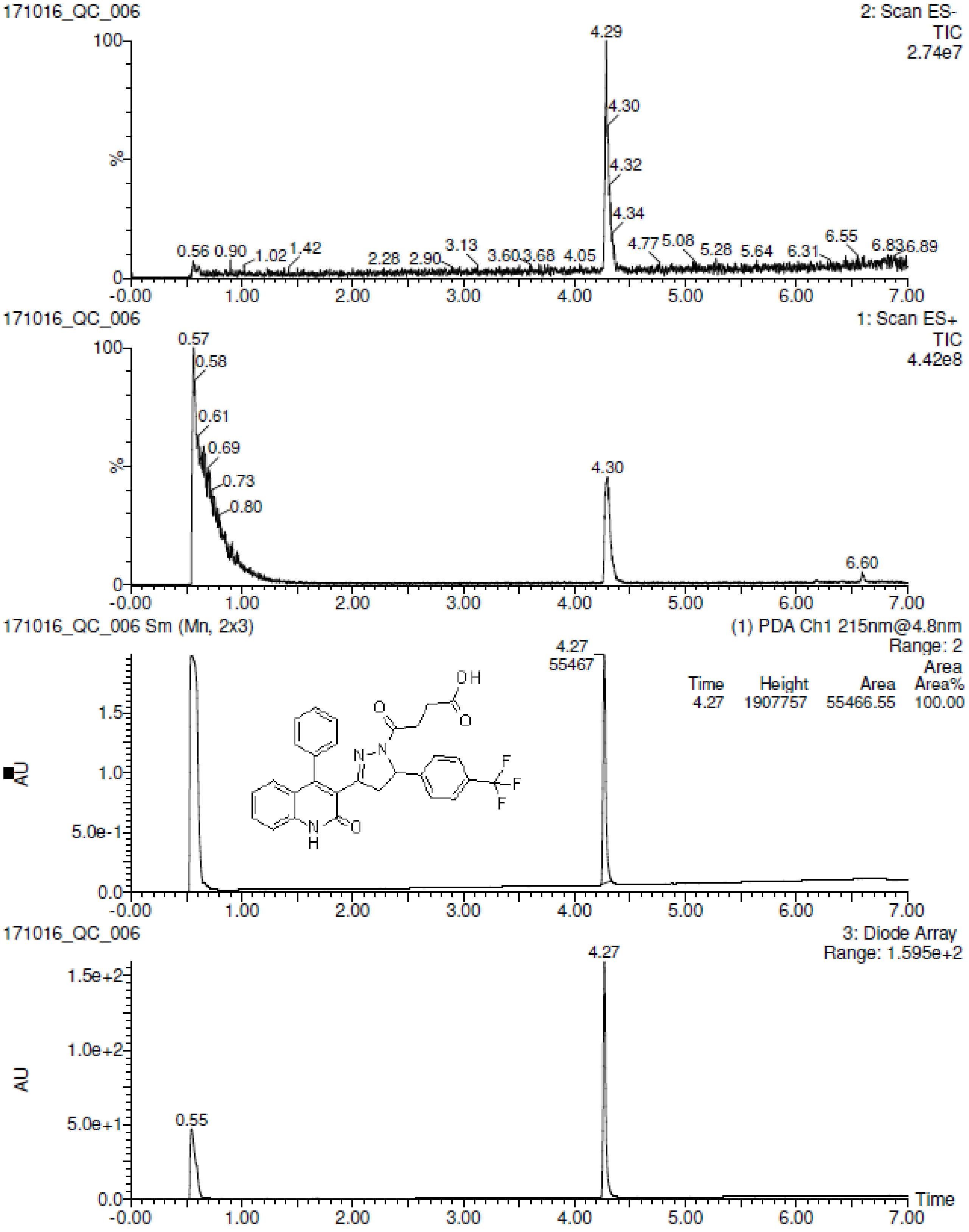

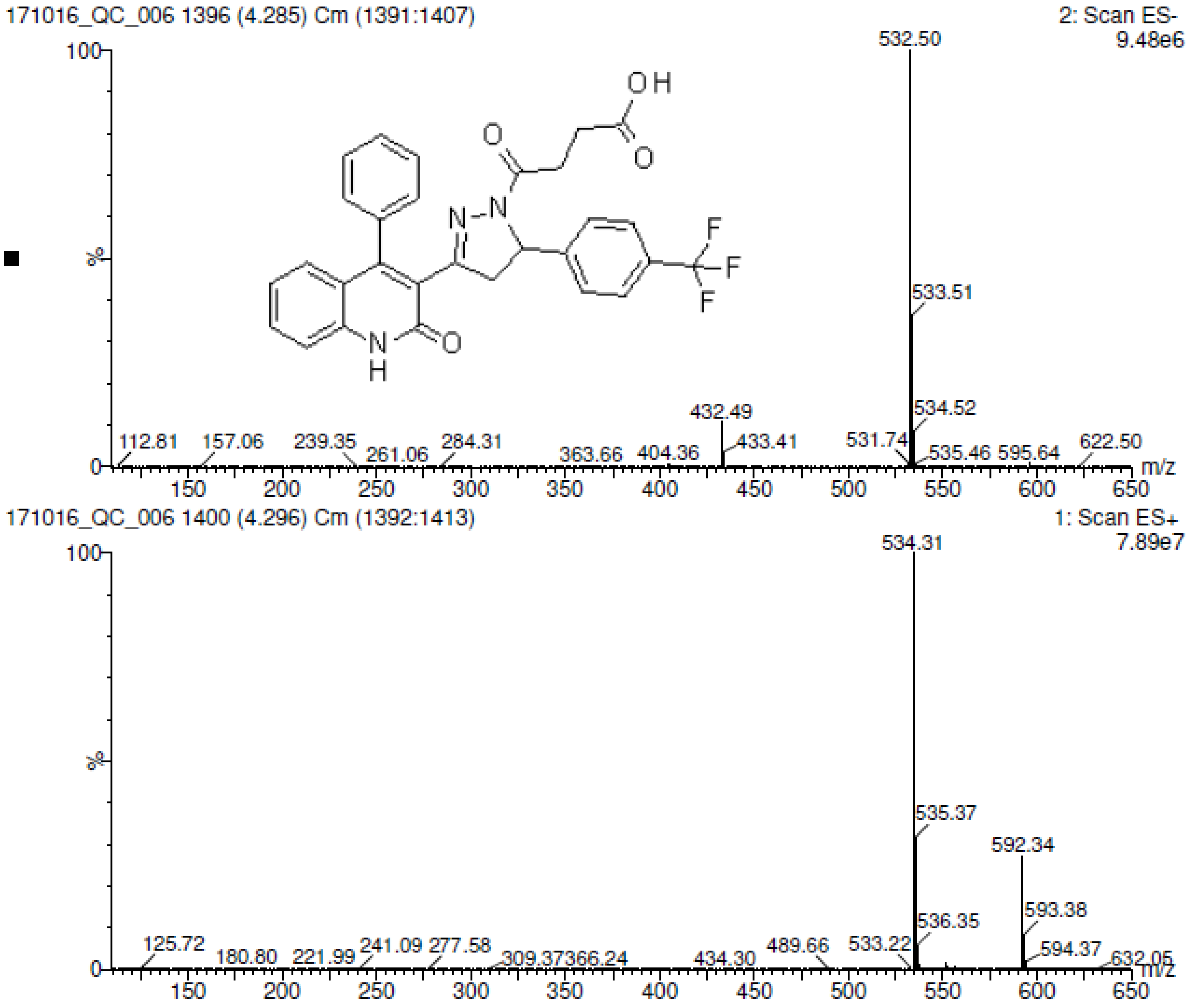

171016_QC_006 1396 (4.285) Cm (1391:1407)
2: Scan ES-
9.48e6
532.50
533.51
534.52
432.49
433.41
531.74
535.46
595.64
622.50
112.81
157.06
239.35
261.06
284.31
363.66
404.36
m/z
171016_QC_006 1400 (4.296) Cm (1392:1413)
1: Scan ES+
7.89e7
534.31
535.37
592.34
593.38
536.35
594.37
632.05
125.72
180.80
221.99
241.09
277.58
309.37
366.24
434.30
489.66
533.22
